# EZH2 is a key prognostic marker and therapeutic target in aggressive and proliferative hepatoblastoma

**DOI:** 10.1186/s12943-025-02474-9

**Published:** 2026-02-23

**Authors:** Fatma Zohra Khoubai, Alexia Calovoulos, Hélène Guillorit, Juan Carrillo-Reixach, Alvaro del Rio-Alvarez, Pierre Klein, Marina Simon Coma, Christophe Avignon, Benoit Rousseau, Lea Mora Charrot, Sandrine Fedou, Jean-William Dupuy, Laura Royo, Montse Domingo-Sàbat, Nadine Thézé, Géraldine Siegfried, Abdel-Majid Khatib, Stefano Cairo, Emilie Indersie, Buddhi Prakash Jain, Véronique Trézéguet, Etienne Gontier, François Lamoureux, Catherine Guettier, Charlotte Mussini, Martin Hagedorn, Carolina Armengol, Christophe F. Grosset

**Affiliations:** 1 Univ. Bordeaux, INSERM, Biotherapy of Genetic Diseases, Inflammatory Disorders and Cancer, BMGIC, U1035, Bordeaux, F-33000 France; 2 Univ. Bordeaux, INSERM, Bordeaux Institute of Oncology, BRIC, U1312, Bordeaux, F-33000 France; 3 Univ. Bordeaux, CNRS, INSERM, Bordeaux Imaging Centre, BIC, UMS 3420, UMS 3420, US 4, Bordeaux, F-33000 France; 4 Childhood Liver Oncology Group, Germans Trias i Pujol Research Institute (IGTP), Badalona, Spain; 5 Networking Biomedical Research Centre (CIBER) in Hepatic and Digestive Diseases, Barcelona, Spain; 6 Department of Pathology, Bicêtre University Hospital, Assistance Publique- Hôpitaux de Paris, Le Kremlin-Bicêtre, F-94275 France; 7 Univ. Bordeaux, Animalerie A2, Département STS, Service commun des Animaleries, Bordeaux, F-33000 France; 8 Univ. Bordeaux, Xenofish platform, F-33000 Bordeaux, France; 9 Univ. Bordeaux, Bordeaux Proteome, F-33000 Bordeaux, France; 10 XenTech SAS, Genopole Campus, 1-3 impasse Alexis Trinquet, Évry, F-91000 France; 11 Champions Oncology, Inc, Baltimore, MD USA; 12 Istituto di Ricerca Pediatrica, Padova, Italy; 13 Department of Zoology, Mahatma Gandhi Central University, Motihari, Bihar India; 14 Nantes Université, Inserm UMR 1307, CNRS UMR 6075, CRCI2NA, Team 9 CHILD, Nantes, F-44000 France; 15 INSERM, MIRCADE team, U1312, Université de Bordeaux, 146, Rue Léo Saignat, Bordeaux, F-33000 France

**Keywords:** Hepatoblastoma, EZH2, GSK126, DUSP9, DUSP5, HMG-Co reductase, Statin

## Abstract

**Background:**

EZH2 is a histone methyltransferase and a key component of polycomb repressive complex 2 (PRC2). It plays a critical role in genome remodeling, gene regulation and acts through PRC2-dependent and independent mechanisms, which comprise methylation of histone and non-histone substrates, and transcriptional activation through different transcriptional complexes. EZH2 is involved in many cancers, but its role in hepatoblastoma is poorly understood.

**Methods:**

Potential correlation between *EZH2* mRNA expression and clinical parameters was analyzed by computational and histological approaches using seven published transcriptomic datasets and tissue samples from patients with hepatoblastoma. EZH2 molecular function was deciphered using molecular approaches, gain- and loss-of-function genetic tools, proteomics, immunohistochemistry, pharmacological drugs, 2D cell- and spheroid-based assays, and four different animal models.

**Results:**

Our data show that *EZH2* mRNA expression correlated with poor prognostic markers such as tumor proliferation, and patients’ death and shorter survival. EZH2 protein potentiated hepatoblastoma cell proliferation, migration, survival and cisplatin resistance through its histone methyltransferase activity by repressing *DUSP5*, and transcriptionally inducing *DUSP9* and *HMGCR*. In vivo EZH2 sustained tumor cell proliferation, and tumor development and angiogenesis. The EZH2 inhibitor GSK126 synergized with HMG-CoA reductase inhibitor statins to eradicate hepatoblastoma cells in vitro and block tumor development in mice. This combination was also very effective on various hepatoblastoma and non-hepatoblastoma tumor cell lines.

**Conclusion:**

Collectively, our data showed that the protein EZH2 promotes hepatoblastoma development, partly through its histone methyltransferase activity, by differentially modulating the expression of *DUSP5*, *DUSP9* and *HMGCR* genes and by supporting the MAPK/ERK pathway in hepatoblastoma cells already displaying high Wnt signal activity. EZH2 inhibitors triggered lipid synthesis in hepatoblastoma cells and synergized with cholesterol-lowering statins to block hepatoblastoma development in vitro and in vivo. Therefore, we demonstrate the key role of EZH2 in proliferative hepatoblastoma and the therapeutic benefit of combining EZH2 inhibitor and statin to treat patients with cancer.

**Supplementary Information:**

The online version contains supplementary material available at 10.1186/s12943-025-02474-9.

## Introduction

Hepatoblastoma (HB) is the most common form of pediatric liver cancer [1]. Its diagnosis is based on radiologic staging with the PRETEXT system, histopathology and serum alpha-fetoprotein (AFP) levels, and the first-line treatment associates cisplatin-based chemotherapy (CT) and surgery [1, 2]. This treatment is effective for four-fifths of patients, but is less satisfactory for children presenting high-risk tumors who are treated with high-dose drug regimens that may induce several secondary pathologies [1–4]. Thus, new agents are needed to address the issues of cisplatin resistance, tumor recurrence and long-term adverse effects in these young patients.

HB is molecularly characterized by the abnormal activation of the Wnt/β-catenin (Wnt) pathway, mostly due to activating mutations in Wnt pathway-related genes [5–10]. In 2008, Cairo, Armengol et al. [6] proposed to distinguish two HB subtypes by a 16-gene signature: a standard-risk C1 group and a more aggressive C2 group. Ten years later, a new transcriptomic investigation performed by our team led to the definition of three independent groups of HB referred to as C1, C2A and C2B [10], further confirmed by additional studies [11–13]. Complementary investigations also revealed the involvement of other oncogenic pathways in HB including epigenetic deregulations, ubiquitination processes and MAPK/ERK pathways [5, 7, 8, 14–17].

EZH2 is a histone methyltransferase and a key component of polycomb repressive complex 2 (PRC2), which catalyzes the trimethylation of histone H3 lysine 27 (H3K27me3) [18]. It plays a critical role in gene regulation and acts through PRC2-dependent and independent mechanisms, which comprise methylation on histone and non-histone targets and transcriptional co-regulation [19, 20]. In cancer cells, *EZH2* deregulations cause PRC2 dysfunction, accumulation of suppressive mark H3K27me3, and thus transcriptome alterations [18]. While the role of EZH2 and its use as a therapeutic target in HB have been briefly reported [21–24], its oncogenic function remains elusive. Notably, the direct interaction between EZH2 and β-catenin proteins in HB was recently confirmed [19, 24].

In this work, we sought to investigate the role of EZH2 in HB. We first interrogated transcriptomic datasets from open-source databases [6, 8, 10–12, 25, 26] and performed thorough bioinformatics studies. We complemented our investigation using RNA interference, HB-derived cells ectopically expressing wild-type (WT) or methyl transferase-dead EZH2, in vitro cell-based assays using HB cells cultured as monolayer or spheroids and chick embryos. Next, we sought to identify new genes regulated by EZH2 and playing a key role in the cancerous, proliferative and chemoresistance potential of HB cells by proteomics and bioinformatics. The role of some of these genes was investigated in HB using the same experimental approaches as described for EZH2. We then evaluated the efficiency of a new combination of two drugs to eradicate HB cells in vitro and in mice and assessed its global efficacy in vitro on a set of non-HB cell lines. Overall, our work aimed to clarify the pathophysiological role of *EZH2* in HB, to evaluate its promise as a therapeutic target, and to propose a new treatment option for patients presenting with a high-risk tumor or a recurrent tumor.

## Materials and methods

### Transcriptomics and bioinformatic analyses

Transcriptomic data and datasets (Supplementary Table S1) supporting the conclusions of this article are included within the publications [6, 8, 10–12, 25, 26] and available in the NCBI’s Gene Expression Omnibus (https://www.ncbi.nlm.nih.gov/geo) [27] and R2: Genomics analysis and visualization platform (https://r2.amc.nl) repositories.

Gene Set Enrichment Analysis (GSEA) [28] was performed using the 32 HB samples from the Carrillo-Reixach et al.. RNAseq data [11]. Specifically, the analysis was performed using specific signaling pathways of the Molecular Signatures Database (MSigDB) related to the genes of interest and categorizing the tumor samples according to their low/high expression of genes of interest according to tumor expression median. GSEA was performed using the complete gene expression dataset, and data were analyzed under the following conditions: number of permutations = 1000, enrichment statistic = weighted, Metric for ranking genes = Signal2Noise. To evaluate statistical differences, we used the signal2noise ratio as a statistic to compare specific phenotypes with specific conditions for number of permutations (*n* = 1000) and enrichment statistic (Method = weighted).

See Supplementary Information for additional material, experimental procedures and data.

## Results

### EZH2 upregulation is associated with proliferative HB and poor prognostic biomarkers

**Fig. 1:**
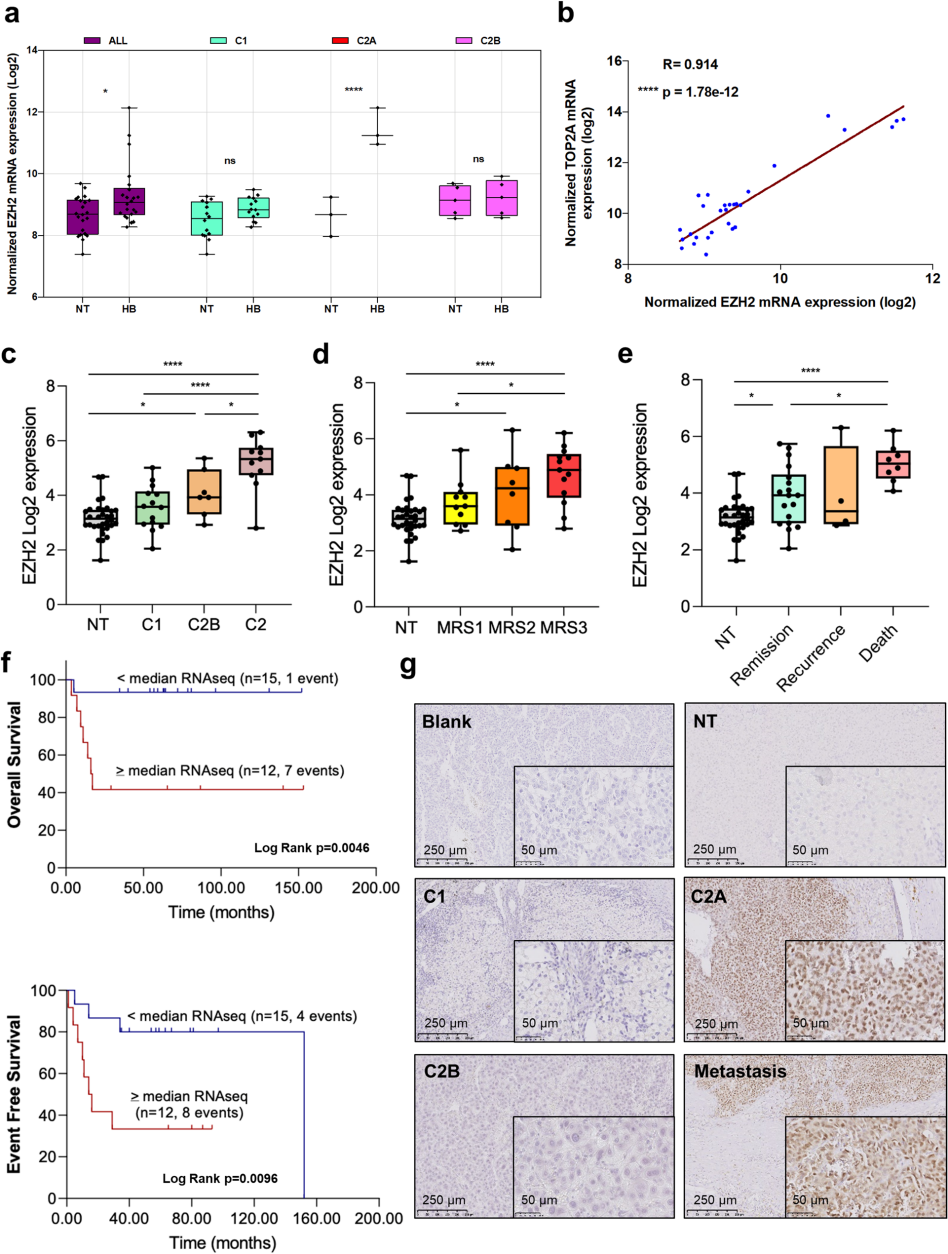
Expression of *EZH2* mRNA and protein in HB and correlative study. (**a**) Expression of *EZH2* transcript in C1, C2A and C2B subgroups and non-tumoral (NT, in this figure and the following) samples from Raymond’s dataset (gse104766 [10]) (Wilcoxon matched pairs signed rank test). (**b**) Graph recapitulates the two-tailed Pearson R correlation between *EZH2* and *TOP2A* transcripts (gse104766 [10]),. (**c-e**) Expression levels of *EZH2* mRNA in NT (*n* = 32), C1 (*n* = 14), C2B (*n* = 7) and C2 tumors (*n* = 11) (**c**, gse133039 [11]),, in NT (*n* = 32), MRS-1 (*n* = 11), MRS-2 (*n* = 8) and MRS-3 (*n* = 13) (**d**, gse133039 [11]), or in NT (*n* = 32), patients in remission (*n* = 18), in recurrence (*n* = 4) and those deceased (*n* = 8) (**e**, gse133039 [11]),. One-way ANOVA test; Tukey post-test. (**f**) Overall survival (top) and event-free survival (bottom) Kaplan Meier plots for patients with follow-up of more than 2 years. Patients were categorized as high or low *EZH2* mRNA expression according to the median of the tumoral RNAseq gene expression data. (**g**) Immunoshistochemical stains of HB patient tissues classed as NT, C1, C2A, C2B [10] and metastasis. Five representative samples, one from each subgroup, were stained using an antibody against EZH2. For this figure and the following: the R, p-value and scale bar are as shown in the corresponding image and graph, respectively. Blank, control stain with no primary antibody. ns, not significant; *p < 0.05; ****p < 0.0001

**Fig. 2:**
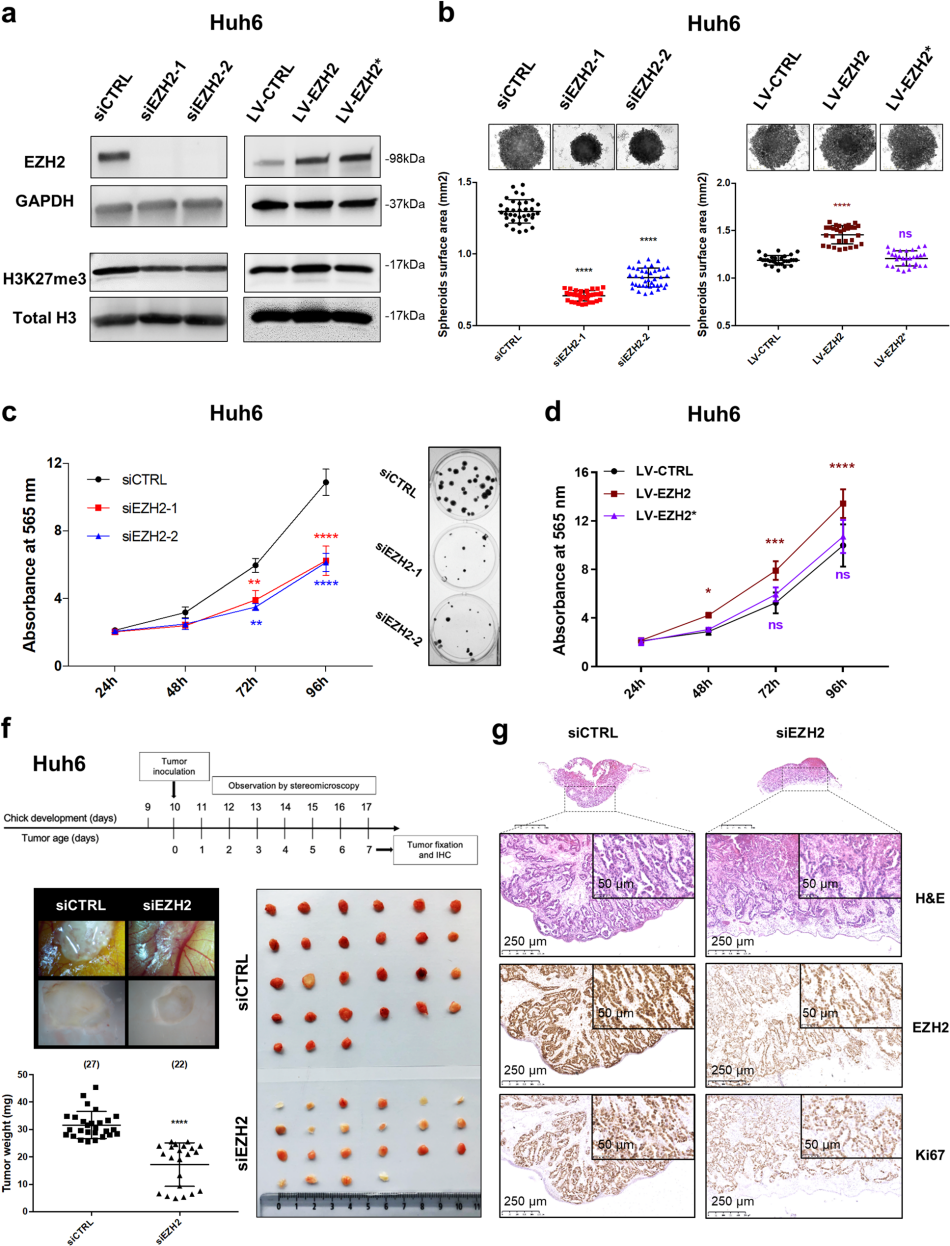
Role of EZH2 and its methyl transferase activity in HB. (**a**) Left blots: level of EZH2 and H3K27me3 proteins in control (siCTRL) and *EZH2*-depleted Huh6 cells using siEZH2-1 or siEZH2-2 as indicated. Right blots: level of EZH2 and H3K27me3 proteins in Huh6 cells transduced by LV-CTRL (empty cassette), LV-EZH2 (wild-type EZH2) or LV-EZH2* (H698A-mutant EZH2) lentiviruses (in this figure and the following). Representative blots of three independent experiments or more are shown in cropped images (loading control: GAPDH or total histone H3 protein as indicated). (**b**) Top panels: phase contrast micrographs of representative 96-h-old spheroids deriving from siCTRL and EZH2-depleted Huh6 cells (left) or from CTRL-, EZH2- or EZH2*-expressing Huh6 cells (right). Bottom panels: graphs presenting spheroid surface area in mm2 for each cellular model as described in top panels (*n* = 4, One way-ANOVA, ****p < 0.0001; Sidak’s multiple comparisons post-test). (**c-d**) Growth (Absorbance at 565 nm) of siCTRL *versus EZH2*-depleted Huh6 cells with a representative clonogenic test shown on the right (**c**) or of CTRL-, EZH2- or EZH2*-expressing Huh6 cells (**d**) (*n* = 3, Two way-ANOVA, ****p < 0.0001; Sidak’s multiple comparisons post-test). (**f**-**g**) CAM assay after implantation of siCTRL or *EZH2*-depleted Huh6 cells using siEZH2-1. (**f**) Top panel: timeline of the whole experiment. Middle left panel: representative stereomicroscopic images of 7-day-old tumors (top) and representative images of extracted and fixed tumors (bottom). Bottom left panel: weight in mg of 7-day-old tumors. The number of eggs analyzed per group is shown in brackets above the corresponding group of values. Horizontal line and whiskers represent mean ± SD (Wilcoxon matched-pairs signed rank test). Right bottom panel: Image of 7-day extracted tumors [siCTRL (top) and siEZH2 (bottom)]. (**g**) Immunoshistochemical stains of Huh6-derived CAM tumors. Representative siCTRL (left) and EZH2-depleted (right) tumors were stained using hematoxylin-eosin (H&S) or antibodies against EZH2 and Ki67. *p < 0.05; **p < 0.01; ***p < 0.001; ****p < 0.0001

Since the role of EZH2 in HB is not completely understood, we first analyzed the expression of *EZH2* transcript in HB by considering the C1/C2A/C2B tumor classification [10]. As shown in Fig. 1a, *EZH2* mRNA was increased in HB compared to NT samples and this expression was specifically associated with the proliferative and topoisomerase 2-alpha (TOP2A)-positive C2A group [10]. In line with this result, a strong positive correlation was found between *EZH2* and *TOP2A* mRNAs in HB samples from the same cohort (Fig. 1b). To confirm these data, we analyzed *EZH2* and *TOP2A* expressions in six additional published transcriptomic datasets (Supplementary Table S1). *EZH2* expression was significantly increased in HB, and it correlated strongly with *TOP2A* expression in all datasets (Supplementary Fig. S1-2 and Table S2) suggesting a close relationship between *EZH2* expression and the proliferative HB subtype. To evaluate the potential link between *EZH2* expression and tumor aggressiveness, additional correlative analyses were performed. *EZH2* mRNA upregulation strongly correlated with several poor prognostic factors including event-free and overall survival, the C2 group defined by Cairo, Armengol et al. [6] and by Carrillo-Reixach et al. [11], the intermediate- and high-risk groups with Molecular Risk Stratification (MRS) 2 and 3, the high 14q32 group and the death of patients (Fig. 2c-f, Supplementary Fig. S3) [11]. In line with our previous studies [10, 14], the MAPK pathway and the apoptotic hallmark gene sets were negatively enriched in tumors with low *EZH2* expression, while the G2M checkpoint hallmark and the Fanconi anemia pathway reactome gene sets were positively enriched in tumors with high *EZH2* expression (Supplementary Fig. S4a). Indeed, we previously reported an activation of the MAPK pathway in HB [14] and the key role of the Fanconi anemia pathway in proliferative TOP2A-positive HB C2A subgroup [10]. Moreover, *EZH2* mRNA was increased at all stages of tumor development as defined by Sumazin et al. [12], independently of the PRETEXT system, and more specifically in embryonal, mixed and small cell histological subtypes, in recurrent tumors and in deceased patients (Supplementary Fig. S4b-f). Finally, immunohistochemical analysis showed that EZH2 protein was detected in the nucleus of HB cells in C2A tumors and a diaphragmatic metastasis, but not in NT, C1 and C2B tumors (Fig. 1g and Supplementary Fig. S5).

Altogether, our data support a close relationship between *EZH2* upregulation, tumor proliferation and poor prognostic markers in HB.

### EZH2 and its methyltransferase activity participate in HB carcinogenesis and cisplatin resistance

To decipher the role of *EZH2* in proliferative HB and the molecular mechanism involved, we combined the use of HB C2A-derived Huh6 and HepG2 cell lines [10], RNA interference technology and infectious lentiviruses encoding the WT EZH2 or the methyl transferase-dead H689A mutated variant (referred to as EZH2* in our work) [29]. As shown in Fig. 2a and Supplementary Figures S6a-b, EZH2-1 and EZH2-2 siRNAs very efficiently silenced *EZH2* mRNA and protein levels in both cell lines. As a consequence, the H3K27me3 marks were lowered. On the contrary, the ectopic expression of WT EZH2 increased the H3K27me3 marks in Huh6 and HepG2 cells, while EZH2* had no effect compared to control (Fig. 2a and Supplementary Fig. S6a). Following *EZH2* depletion, the size of HB-derived spheroids was negatively affected, the growth and the clonogenic capacity of HB cells were reduced, the migration of Huh6 cells was inhibited and HB cells cultured either in the 2D or 3D condition entered senescence with no sign of apoptosis, as illustrated by an increase in P21 and β-galactosidase positive staining, and the lack of dead (red-positive) cells and caspase-3/7 activation (Fig. 2b-c and Supplementary Fig. S6c-d, S7 to S9). In contrast, ectopic expression of WT EZH2 increased the growth of HB cells as a monolayer or spheroids, while the EZH2* mutant had no effect (Fig. 2b and d, Supplementary Fig. S6c and e).

Since cisplatin is used as first-line treatment for HB patients, we also evaluated the role of *EZH2* in response to cisplatin. As shown in Supplementary Figure S10a-c, *EZH2* silencing slightly but significantly increased the sensitivity of HB cells to cisplatin cultured either as monolayer or spheroids. In contrast, forced expression of WT EZH2 increased the resistance of HB cells, while EZH2* had no effect (Supplementary Fig. S10d). At the molecular level, EZH2 loss reduced ERK phosphorylation, while WT EZH2 had the opposite effect and no change was observed with EZH2* (Supplementary Figure S11). These results support a link between *EZH2* expression and active MAPK pathway signaling in HB [14]. Finally, using the in vivo chick chorioallantoic membrane (CAM) assay and immunohistochemical analysis [30, 31], we found that following HB cell implantation, *EZH2* depletion led to a significant reduction in tumor weight, vascularization and bleeding, as well as in Ki67 biomarker level (Fig. 2f-g, Supplementary Fig. S12).

Together, these data demonstrated the pro-proliferative, pro-migratory, pro-angiogenic, pro-tumoral and anti-senescent function of *EZH2* in HB cells, and the key role of its methyl transferase activity in tumor cell growth and survival, cisplatin resistance [32], MAPK signaling and HB development.

## DUSP9 is a key prognostic marker in HB and is positively regulated by EZH2

**Fig. 3:**
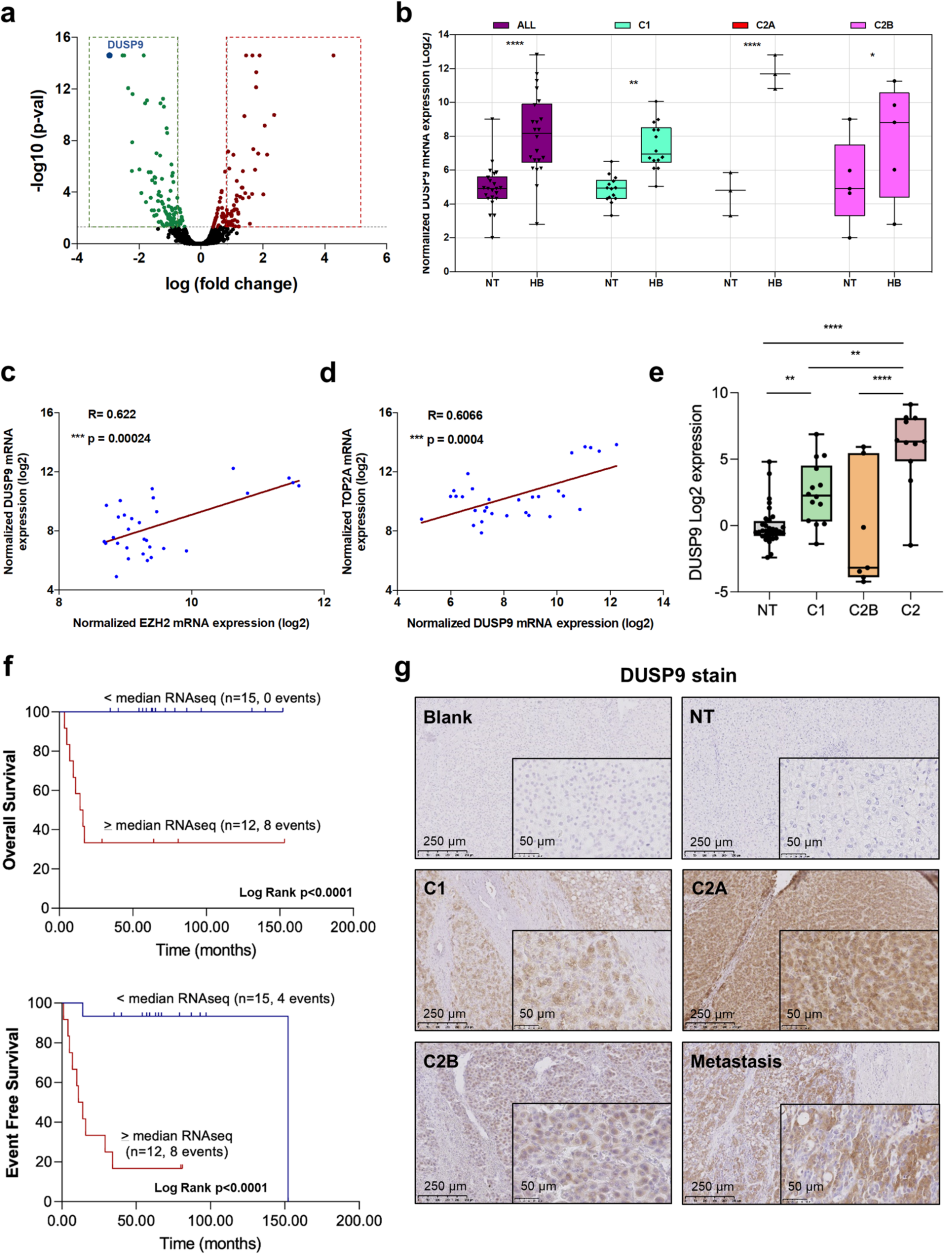
Expression of *DUSP9 mRNA and protein* in HB and correlative study. (**a**) Volcano plot of protein level fold change (X-axis, log2) *versus* q-values (Y-axis, log10) in siEZH2-1 *versus* siCTRL Huh6 cells. Proteins significantly up- and down-regulated in *EZH2*-depleted cells are shown in red and green, respectively. DUSP9 protein is depicted as a blue spot. (**b**) Expression of *DUSP9* in C1, C2A and C2B subgroups of HB and NT samples from Raymond’s dataset (gse104766 [10]), (Wilcoxon matched pairs signed rank test). (**c-d**) Graphs recapitulate the Two-tailed Pearson R correlation between *DUSP9* and *EZH2* (**c**) or *TOP2A* transcripts (**d**) (gse104766 [10]). (**e**) Expression levels of *DUSP9* mRNA in NT (*n* = 32), C1 (*n* = 14), C2B (*n* = 7) and C2 tumors (*n* = 11) (gse133039 [11]). One-way ANOVA test, ****p < 0.0001; Tukey post-test. (**f**) Overall survival (top) and event-free survival (bottom) Kaplan Meier plots for patients with follow-up of more than 2 years. Patients were categorized as high or low *DUSP9* mRNA expression according to the median of the tumoral RNAseq gene expression data. (**g**) Immunoshistochemical stains of HB patient tissues classed as NT, C1, C2A, C2B and a lung metastasis. Five representative samples, one from each subgroup, were stained using an antibody against DUSP9. *p < 0.05; **p < 0.01; ***p < 0.001; ****p < 0.0001

To explore the molecular mechanisms underlying the oncogenic effect of *EZH2* in HB, we performed comparative proteomic analysis in *EZH2*-silenced and control Huh6 cells (Fig. 2a). Using a cut-off fold change of 1.8 and a cut-off p-value of 0.05, 54 and 87 proteins were significantly and respectively up- and down-regulated in *EZH2*-silenced cells *versus* control (Fig. 3a and Supplementary Table S3). The down-regulation of CTSV and DUSP9 proteins, as well as the upregulation of P16-INK4a and β-catenin proteins in *EZH2*-silenced HB cells were confirmed by Western blotting partly supporting our proteomic data (Supplementary Fig. S13).

Next, we focused our attention on dual-specificity phosphatase 9 (DUSP9), a member of the threonine/tyrosine dual-specific phosphatase family, which is known to dephosphorylate ERK1/2, p38, JNK and ASK1 and to control various MAPK-associated pathways [33]. Moreover, DUSP9 is part of the 16-gene signature discriminating C1 and C2 tumors, and is more expressed in the poor prognostic C2 subgroup as defined by Cairo, Armengol et al. [6]. Additional in vitro and in vivo analyses (using the CAM assay as described above) confirmed that both *DUSP9* mRNA and protein levels are strongly decreased in *EZH2*-silenced Huh6 and HepG2 cells (Supplementary Fig. S14). Importantly, *DUSP9* transcript was upregulated in all tumors from our cohort (with a higher increase in C2A group as for *EZH2* [10]) and from six additional datasets (Fig. 3b and Supplementary Fig. S15). Moreover, *DUSP9* mRNA expression correlated positively with *EZH2* mRNA expression in HB samples from our cohort and from 5 out of 6 additional datasets (Fig. 3c and Supplementary Fig. S16 and Table S4), and with the proliferative biomarker *TOP2A* in all HB datasets (Fig. 3d and Supplementary Fig. S17 and Table S2). suggesting direct connection between *DUSP9*, *EZH2* and *TOP2A* in HB. In agreement with a previous study [6], *DUSP9* upregulation correlated strongly with several poor prognostic factors including event-free and overall survival, the C2 group defined by Carrillo-Reixach et al. [11], the Epi-CB group, the intermediate- and high-risk groups with MRS2/3 groups, the high 14q32 group and the death of patients (Fig. 3e-f and Supplementary Fig. S18a-e) [11]. In terms of gene set enrichment, *DUSP9* had the same profile as *EZH2* (compare Supplementary Fig. S4a and S18f). Finally, using immunohistochemical analysis and in line with the transcriptomic data (Fig. 3b), we showed that DUSP9 protein is present in the cytoplasm of HB cells from a diaphragmatic metastasis and from all tumor groups (with a higher expression in C2A compared to C1 and C2B), but not in NT (Fig. 3g and Supplementary Fig. S19).

Since DUSP9 phosphatase is a regulator of MAPK pathways [33, 34] and a biomarker of poorly differentiated and proliferative HB, we investigated its role in HB. By RNA interference, we found that *DUSP9* silencing slightly reduced HB cell growth and unexpectedly, inactivated ERK kinase and MAPK signaling (Supplementary Fig. S20), a result in agreement with some data reported in liver cancer [33, 34]. On the contrary, ectopic expression of *DUSP9* had no effect on HB cells growing as monolayer or spheroids, likely because these cells are already actively proliferating (Supplementary Fig. S21).

Altogether, these results suggest that EZH2 positively regulates *DUSP9* expression in HB, likely at the transcriptional level. They also show that *DUSP9* constitutes a biomarker of the C2A group [10] and it acts as a moderate oncogene in proliferative HB cells, despite being highly expressed in C2A group.

### EZH2 contributes to HB carcinogenesis and drug resistance by repressing DUSP5

**Fig. 4:**
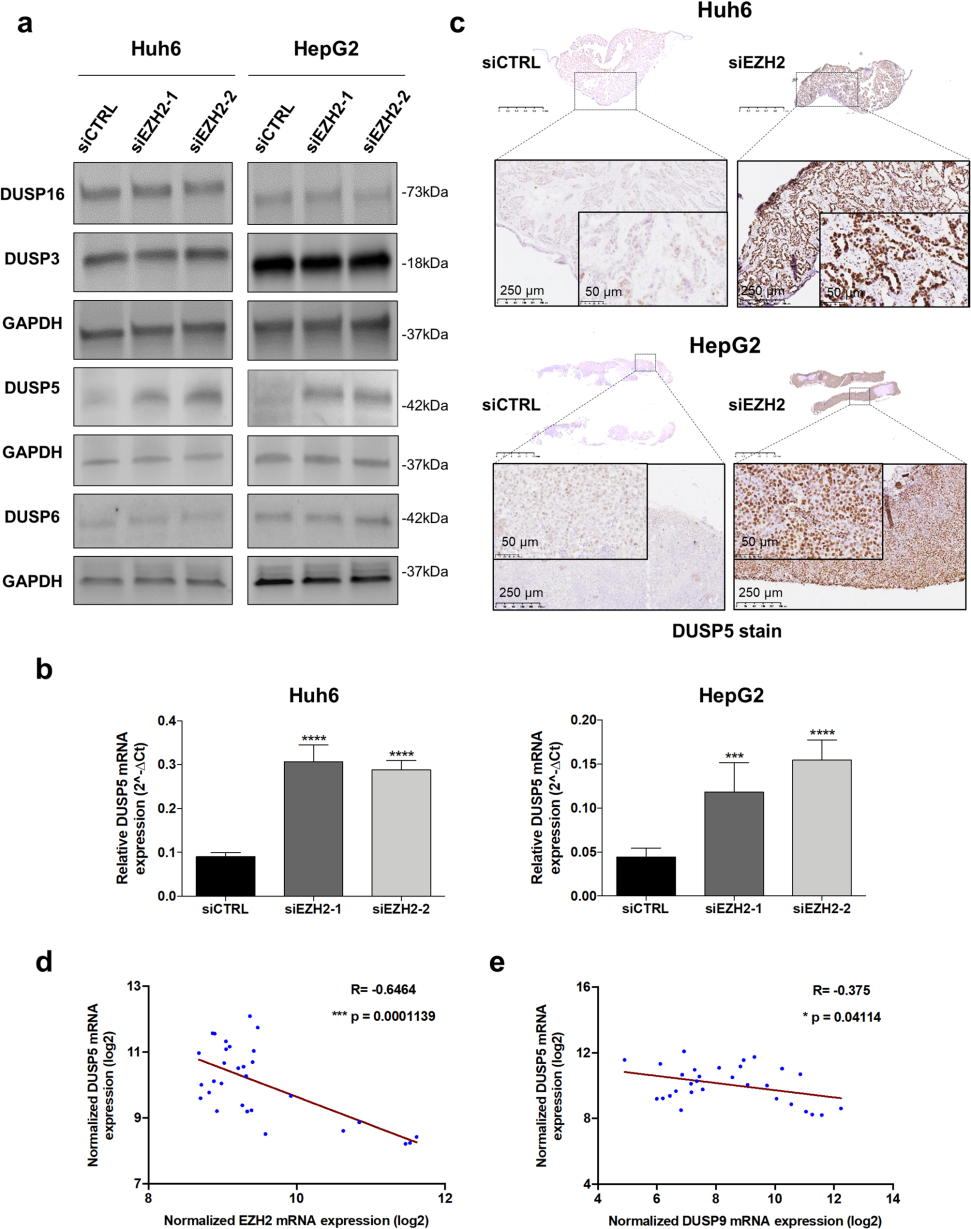
EZH2 supports the MAPK signaling in proliferative HB by repressing *DUSP5* expression. (**a**) Level of DUSP3, DUSP5, DUSP6 and DUSP16 proteins in siCTRL *versus EZH2*-depleted Huh6 (left) or HepG2 (right) cells using siEZH2-1 or siEZH2-2 as indicated. Representative blots of three independent experiments or more are shown in cropped images (loading control: GAPDH). (**b**) Relative expression of *DUSP5* mRNA in siCTRL *versus EZH2*-depleted Huh6 (left) or HepG2 (right) cells. Bar graphs show means ± SD (*n* = 3, One way-ANOVA, *p* < 0,0001; Sidak’s multiple comparisons post-test). (**c**) Immunoshistochemical stains of Huh6-derived (top) or HepG2-derived (bottom) CAM tumors. Representative siCTRL (left side) and EZH2-depleted (right side) tumors were stained using an antibody against DUSP5. (**d-e**) Graphs recapitulate the two-tailed Pearson R correlations between *DUSP5* and *EZH2* (**d**) or DUSP9 (**e**) transcripts (gse104766 [10]). ***p < 0.001; ****p < 0.0001

As the role of *DUSP9* was unable to elucidate the oncogenic effect of *EZH2* in proliferative HB, we studied the involvement of other DUSP family members with a focus on DUSPs downregulated in HB. Using our transcriptomic dataset, we found that *DUSP5*, *DUSP6* and *DUSP16* mRNAs, but not *DUSP3* mRNA, were down-regulated in all HB groups compared to NT samples (Supplementary Fig. S22) [33, 34]. In Western blot experiments, DUSP5 protein was the only DUSP-family member significantly increasing following *EZH2* loss and its up-regulation in HB cells was also observed in vivo in the nucleus of HB cells implanted on CAM and in vitro at the mRNA level (Fig. 4a-c). In contrast, ectopic expression of WT EZH2 decreased both DUSP5 protein and mRNA levels in Huh6 cells, while EZH2* had no effect (Supplementary Fig. S23a-b). It should be mentioned that DUSP5 was poorly expressed in Huh6 cells and not detectable in HepG2 cells under our experimental conditions. To assess whether the genomic sequence of *DUSP5* was marked by H3K27me3 in HB cells, we performed a ChIP assay. In contrast to androgen receptor (AR) and CDKN2A promoters - here used as positive controls [35, 36] - our results showed that *DUSP5* gene was not associated with the H3K27me3 mark in Huh6 cells (Supplementary Fig. S23c), suggesting that *DUSP5* expression is not regulated by this epigenetic mechanism in this pediatric cancer.

DUSP5 is a typical nuclear MAP kinase phosphatase (MKP) and a tumor suppressor gene in many cancers [33, 34]. Our transcriptomic analyses showed that *DUSP5* transcript is decreased in all HB datasets with a stronger decrease in the C2A group from our dataset (Supplementary Fig. S22 and S24) [10], and is inversely correlated with *EZH2*, *DUSP9* and TOP2A mRNAs in all analyzed HB datasets except one (Fig. 4d-e and Supplementary Fig. S25-S27 and Tables S2 and S4). *DUSP5* down-regulation also correlated strongly with several poor prognostic factors including the C2 group defined by Carrillo-Reixach et al. [11], the Epi-CB group, the intermediate- and high-risk groups with MRS2/3 groups, the high 14q32 group, the death of patients, and disease recurrence (Supplementary Fig. S28a-g) [11]. In terms of gene set enrichment, *DUSP5* had the exact opposite profile to that described for *EZH2* and *DUSP9* (compare Supplementary Fig. S4a, S18f and S28h).

**Fig. 5:**
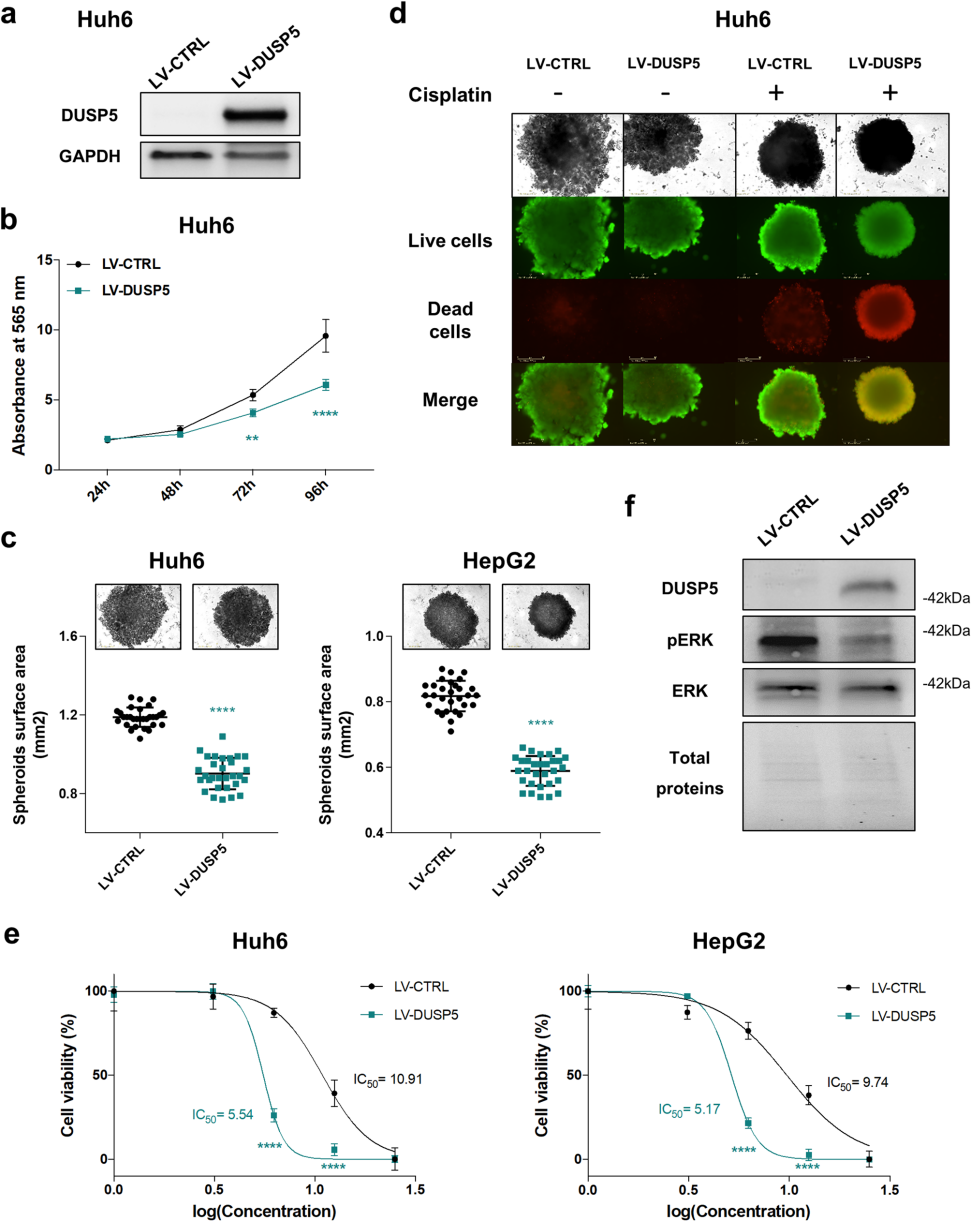
DUSP5 acts as a major tumor suppressor gene in HB cells. (**a**) Level of DUSP5 protein in Huh6 cells expressing the DUSP5 (LV-DUSP5) or CTRL (LV-CTRL) cassette. (**b**) Growth (Absorbance at 565 nm) of CTRL and DUSP5-expressing Huh6 cells (*n* = 3, Two way-ANOVA, **p < 0.01; Sidak’s multiple comparisons post-test). (**c**) Top panels: representative phase contrast micrographs of 96-h-old spheroids deriving from CTRL and DUSP5-expressing Huh6 (left) or HepG2 (right) cells. Bottom panels: graphs presenting spheroid surface area in mm2 in the conditions described above (*n* = 4, Unpaired Mann Whitney test). (**d**) Huh6 cells expressing DUSP5 or the CTRL cassette were cultured as spheroids and treated or not with cisplatin at IC_50_ dose. Top panels: phase contrast micrographs of representative spheroids. Bottom panels: live and dead cells stained with calcein-AM and ethidium homodimer-1 reagents. In this figure and the following, live cells appear in green and dead cells in red. Representative images of four independent experiments. (**e**) Percentage of viable CTRL and DUSP5-expressing Huh6 (left) and HepG2 (right) cells treated with increasing concentrations of cisplatin (*n* = 3; bars = means +/- SD; Two way-ANOVA, ***p < 0.001; Sidak’s multiple comparisons post-test). (**f**) Level of DUSP5, phospho-ERK (pERK) and total ERK proteins in CTRL and DUSP5-expressing Huh6 cells. Representative blots of three experiments are shown in cropped images (loading control: total proteins). **p < 0.01; ****p < 0.0001

To evaluate the potential tumor suppressive role of *DUSP5* in HB, we ectopically expressed the phosphatase in Huh6 and HepG2 cells (Fig. 5a and Supplementary Fig. S29a). Compared to control empty transgene, the forced expression of *DUSP5* inhibited the proliferation of HB cells growing as monolayer or spheroids, and significantly increased their sensitivity to cisplatin (Fig. 5b-e, Supplementary Fig. S29b-c). Finally, by Western blotting, we showed that DUSP5 expression strongly reduces ERK phosphorylation and consequently, inactivates the MAPK pathway in huh6 cells (Fig. 5f). To assess whether EZH2 regulates tumor function through DUSP5, we also conducted both in vitro and in vivo experiments using Huh6 cells engineered to ectopically express EZH2, DUSP5 or both (Supplementary Fig. S30a). As expected, EZH2 promoted the growth and migration of Huh6 cells in vitro, and tumor development in vivo in zebrafish, while DUSP5 had the totally opposite effect (Supplementary Fig. S30b-c and S31). Interestingly, co-expression of DUSP5 and EZH2 revealed a dominant inhibitory effect of DUSP5 over EZH2 (Supplementary Fig. S30b-c and S31). These findings indicate that suppression of DUSP5 protein is essential for EZH2 to exert its pro-oncogenic function in HB cells.

Altogether, these results suggest a negative and indirect regulation of *DUSP5* expression by EZH2 through its methyl transferase activity and show that DUSP5 phosphatase acts as a tumor suppressor in HB by inhibiting cell proliferation and sensitizing HB cells to cisplatin.

## Cisplatin and EZH2 inhibitor GSK126 cooperate to eradicate HB cells

**Fig. 6:**
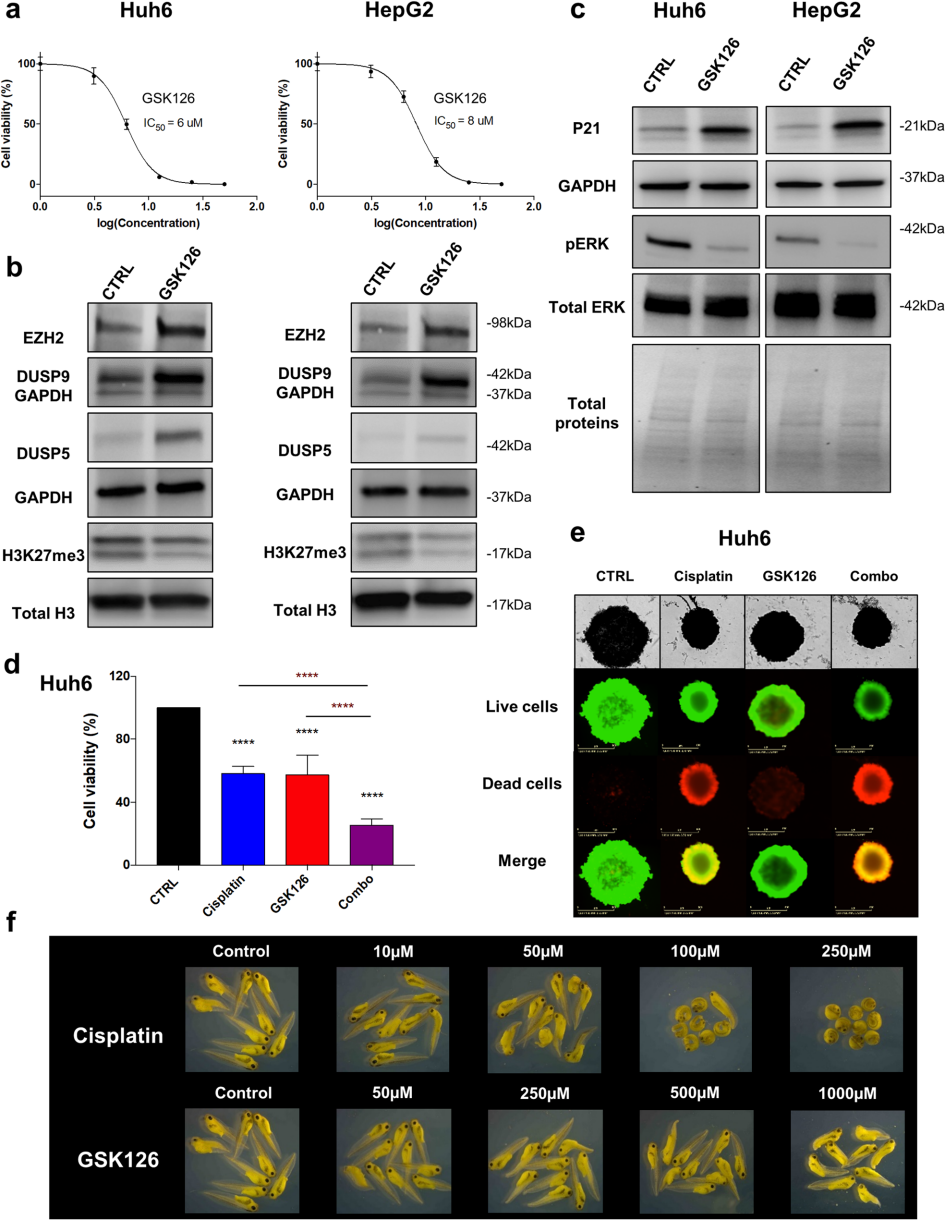
Effect of GSK126 alone or in combination with cisplatin on HB cells. (**a**) Graphs show the percentage of viable Huh6 (left) and HepG2 (right) cells treated with increasing concentrations of GSK126 (*n* = 3; bars = means +/- SD; the IC_50_ is as shown). (**b**) Levels of EZH2, DUSP9, DUSP5 and H3K27me3 proteins in control (DMSO) *versus* GSK126-treated Huh6 (left) or HepG2 (right) cells. (**c**) Levels of p21, phospho-ERK (pERK) and total ERK proteins in control (DMSO) *versus* GSK126-treated Huh6 (left) or HepG2 (right) cells. (**b-c**) GSK126 used at IC_50_. Representative blots of three independent experiments are shown in cropped images (loading control: GAPDH, histone H3 protein or total proteins as indicated). (**d-e**) GSK126 and cisplatin cooperate to eradicate Huh6 cells in vitro. (**d**) Graphs show the percentage of viable cells cultured as monolayer and treated or not for 24 h by cisplatin at IC_50_, and then treated or not for 48 h by GSK126 at IC_50_, or by the combo. Bar graphs recapitulate means ± SD (*n* = 5, One way-ANOVA, ****p < 0.0001; Sidak’s multiple comparisons post-test). CTRL: drug solvent. (**e**) Images showing live and dead cells cultured as spheroids and treated with drug(s) as described in **d**. Top panels: phase contrast micrographs of representative spheroids. Bottom panels: cells stained with calcein-AM and ethidium homodimer-1 reagents. Representative images of four independent experiments. (**f**) Toxicological study using xenopus embryo development as readout following treatment by increasing doses (as indicated) of cisplatin or GSK126. Representative images of three independent experiments

Since the key role of EZH2 methyl transferase activity in HB, we tested the sensitivity of tumor cells to cisplatin and two EZH2 inhibitors, referred to as GSK126 and EPZ6438 (also known as tazemetostat) [37]. As shown in Fig. 6a and Supplementary Figure S32, the IC_50_ for GSK126 and EPZ6438 in Huh6 and HepG2 cells were 6 to 8 µM and above 28 µM, respectively. Based on these results, we selected GSK126 for further analyses. In line with our previous data in WT EZH2- or EZH2*-expressing HB cells, the H3K27me3 mark was significantly reduced and the level of DUSP5 protein was increased in GSK126-treated Huh6 and HepG2 cells compared to control (Fig. 6b), further supporting the hypothesis that EZH2 epigenetically represses *DUSP5* gene expression through its methyltransferase activity. Importantly, GSK126-treated HB cells also expressed higher levels of EZH2 and DUSP9 proteins (Fig. 6b) suggesting an activation of a compensatory mechanism when EZH2 methyl transferase activity was inhibited and further supporting a positive regulation of *DUSP9* by EZH2 through its transcriptional co-regulation function [19, 20]. GSK126 also induced P21 expression and ERK dephosphorylation predicting a proliferation arrest and an entry in senescence of HB cells in presence of this inhibitor (Fig. 6c, see also Supplementary Fig. S8). Next, HB cells were treated with cisplatin, GSK126 or both drugs and the cell viability was measured. As shown in Fig. 6d-e and Supplementary Figure S33, cisplatin and GSK126 cooperated to eliminate HB cells in vitro, whether the cells were growing as monolayer or spheroid. Importantly, GSK126 was 20-fold less deleterious than cisplatin in vivo using the *Xenopus* embryo as toxicological assay (Fig. 6f). Indeed, embryonic development was affected at a dose of 50 µM for cisplatin, while 1 mM of GSK126 was required to give similar developmental alterations.

Altogether, these results show that the combination of cisplatin and GSK126 could be beneficial in clinical practice to improve first-line treatment efficacy or to treat patients in relapse presenting with resistance to cisplatin and/or proliferative tumor.

### EZH2 inhibitors synergize with cholesterol-lowering Statins to eradicate tumor cells

**Fig. 7:**
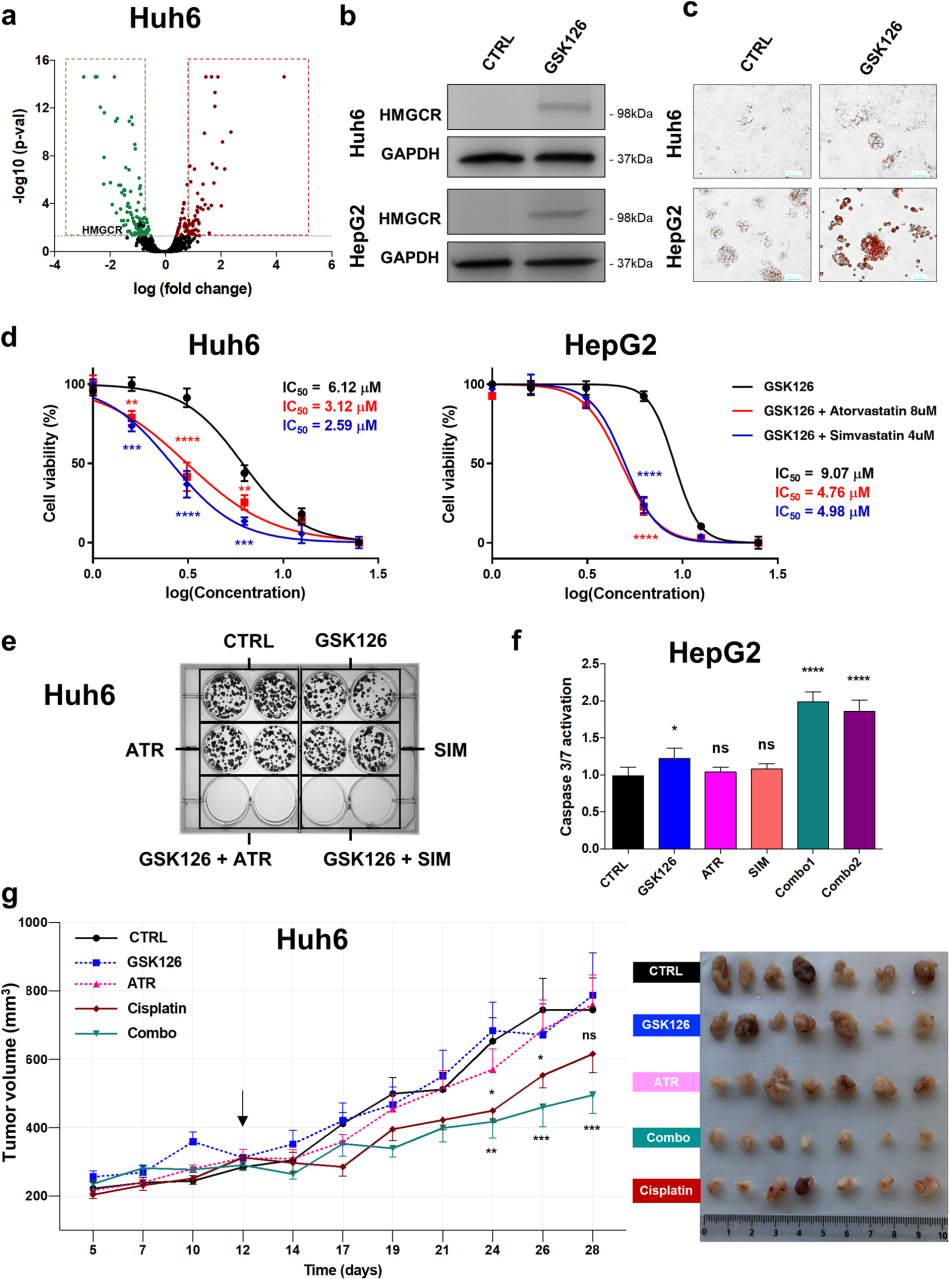
Mevalonate pathway is involved in the response of HB cells to GSK126. (**a**) Volcano plot as shown in Fig. 3a. HMCGR protein is depicted as a black spot in down-regulated genes (left green spots). (**b**) Levels of HMGCR protein in control (DMSO) *versus* GSK126-treated Huh6 (top) or HepG2 (bottom) cells (GSK126 used at IC_50_). Representative blots of three independent experiments are shown in cropped images (loading control: GAPDH). (**c**) Red oil staining of Huh6 (top) or HepG2 (bottom) cells treated with DMSO (CTRL) or GSK126 used at IC_50_ dose as indicated. Scale bar = 100 μm. (**d**) Graphs show the percentage of viable Huh6 (left) or HepG2 (right) cells treated with increasing doses of GSK126 in combination or not with a non-toxic dose of atorvastatin (8 µM) or simvastatin (4 µM) as indicated (*n* = 3; bars = means +/- SD; Two way-ANOVA, ***p < 0.001; Sidak’s multiple comparisons post-test). (**e**) Survival and proliferation of Huh6 cells treated with DMSO (control: CTRL), GSK126 at IC25 dose (3 µM), a statin (ATR: atorvastatin at 8 µM; SIM: simvastatin at 4 µM) or the combination of both. Representative images of three independent experiments. (**f**) Graphs presenting caspase 3/7 activity in HepG2 cells after treatment with GSK126, atorvastatin (ATR), simvastatin (SIM), Combo 1 (GSK126 + atorvastatin) or Combo 2 (GSK126 + simvastatin) (*n* = 3, One way-ANOVA, not significant; Sidak’s multiple comparisons post-test). ns, not significant. (**g**) Left panel: graph represents tumor volume kinetics in mice repeatedly treated with the vehicle, cisplatin (1 mg/kg), GSK126 (50 mg/kg), atorvastatin (20 mg/kg) or the combination of GSK126 and atorvastatin (Two way-ANOVA, ***p < 0.001; Sidak’s multiple comparisons post-test). Arrow: treatment starting point. Right panel: images of the extracted tumors for each group. ns, not significant; *p < 0.05; **p < 0.01; ***p < 0.001; ****p < 0.0001

Since studies have reported the link between EZH2 function and lipid synthesis [38–40], we assessed the level of 3-hydroxy-3-methylglutaryl coenzyme A-CoA reductase (HMGCR) protein in our proteomic data. Our analyses revealed that this key enzyme of the mevalonate pathway [41, 42] was decreased two-fold in *EZH2*-silenced cells compared to control cells (Fig. 7a, Supplementary Table S3). The downregulation of HMGCR protein in *EZH2*-silenced HB cells was confirmed by Western blotting (Supplementary Fig. S34a). Consecutive to *EZH2* depletion and HMGCR protein decrease, the number of lipid droplets was strongly reduced in Huh6 cell-derived spheroids compared to control spheroids as revealed by transmission electron microscopy (Supplementary Fig. S34b). Moreover, mitochondria were smaller in *EZH2*-silenced Huh6 cells and their cristae were darker, suggesting a profound metabolic reprograming and a potential defect in oxidative phosphorylation activity [43]. In contrast, blocking EZH2 methyl transferase activity with GSK126 induced the expression of HMGCR protein and the accumulation of lipid droplets in HB cells further indicating a positive connection between *EZH2* and *HMGCR* in these cells (Fig. 7b-c).

In tumors, *HMGCR* transcript was upregulated in most HB datasets with a stronger increase in the C2A group as for *EZH2* and *DUSP9*, and its expression correlated with *EZH2* and *TOP2A* mRNAs in most analyzed HB datasets (Supplementary Fig. S35-S37 and Tables S2 and S4). *HMGCR* transcript was particularly increased in the Epi-CB, the intermediate- and high-risk groups with MRS2/3 groups and the high 14q32 groups (Supplementary Fig. S38a-e). However, unlike *EZH2* and *DUSP9* mRNAs, its expression did not correlate with patients survival or death (Supplementary Fig. S38f-h). Finally, *HMGCR* had the same gene set enrichment profile as described for *EZH2* and *DUSP9* (compare Supplementary Fig. S4a, S18f and S39).

To counteract the enzymatic activity of HMGCR, we treated HB cells with atorvastatin or simvastatin, two statins widely used for the treatment of heart diseases and dyslipidemia [41, 42]. As shown in Supplementary Figure S40a, alone, both statins inhibited HB cell growth only at doses comprised between 16 and 50 µM. By treating HB cells with increasing doses of GSK126, EPZ6438 or cisplatin and a non-cytotoxic dose of atorvastatin or simvastatin, we found that statins synergistically increased the sensitivity of HB cells to EZH2 inhibitors, and not to cisplatin (Fig. 7d and Supplementary Fig. S40a-c). Importantly, the sensitivity of HB cells to atorvastatin and simvastatin was also considerably increased by the addition of a non-cytotoxic dose of GSK126 (used at 50% of IC_50_ dose), and HB cells died even more rapidly when cisplatin was added (at 25% of IC_50_ dose) to the combination (Supplementary Fig. S40a). Moreover, the synergistic effect of GSK126 with statins totally impeded the capacity of HB cells to form clones and to migrate in vitro, and the combination of GSK126 with any of the two statins strongly induced the apoptosis of HB cells (Fig. 7e and f, and Supplementary Fig. S41). Finally, the combination of GSK126 and atorvastatin, and not the drugs alone, significantly impeded HB development in vivo using a xenograft model of HB in mice [10], without affecting blood parameters or the body weight (Fig. 7g and Supplementary Fig. S42). Here, the cisplatin was used as positive control. To complete this work, we tested in vitro the benefit of combining GSK126 and statins in HB patient-derived xenograft (PDX) and additional non-HB tumor cell lines. Data in Supplementary Figures S43-S50 showed that statins significantly increase the sensitivity of different tumor cell types to GSK126. The combination was particularly effective on HB PDX HB303 cells, adult lung carcinoma-derived NCI-H23 cells and the pediatric osteosarcoma-derived HOS-MNNG and U2-OS cells.

Altogether, these data demonstrate the benefit of combining EZH2 inhibitors and statins to eradicate HB cells and non-HB cells, as previously shown with diffuse midline glioma H3K27-altered cells [40].

## Discussion

EZH2 is a multifunctional protein with numerous modes of action in the cell [19, 20]. By trimethylating H3 histone lysine 27, EZH2 controls chromatin structure and gene regulation, but it can also methylate various non-histone substrates as GATA4, STAT3 and β-catenin, and participate as a co-factor in transcriptional complexes by interacting with TCF/β-catenin, nuclear factor-kappa B and estrogen receptor alpha [19, 20]. Through these regulations, EZH2 supports multiple pathways including the Wnt, MAPK/ERK and Myc pathways, which are all critical in HB [6, 10, 14]. However, its oncogenic role in HB is not fully understood [21–24].

Our findings confirm the upregulation of *EZH2* in HB [21, 24, 44] and go a step further by showing that *EZH2* transcript is particularly increased in tumors with pejorative clinical features, including proliferative TOP2A-positive C2A/C2 tumors [10, 11], disease recurrence and patients’ poor survival and death, thus making *EZH2* expression a marker of poor prognosis. Our data also point to the oncogenic role of *EZH2* in HB [22, 45], and demonstrate that EZH2 histone methyltransferase activity is required for both the growth, migration, survival, clonogenicity and cisplatin resistance of HB cells. Finally, we show that EZH2 protein participates actively in tumor growth and angiogenesis in vivo. All these data are in agreement with the widely reported tumorigenic functions of this enzyme and its key role as an epigenetic regulator in many adult and pediatric cancers including HB [19, 20, 24, 46].

**Fig. 8:**
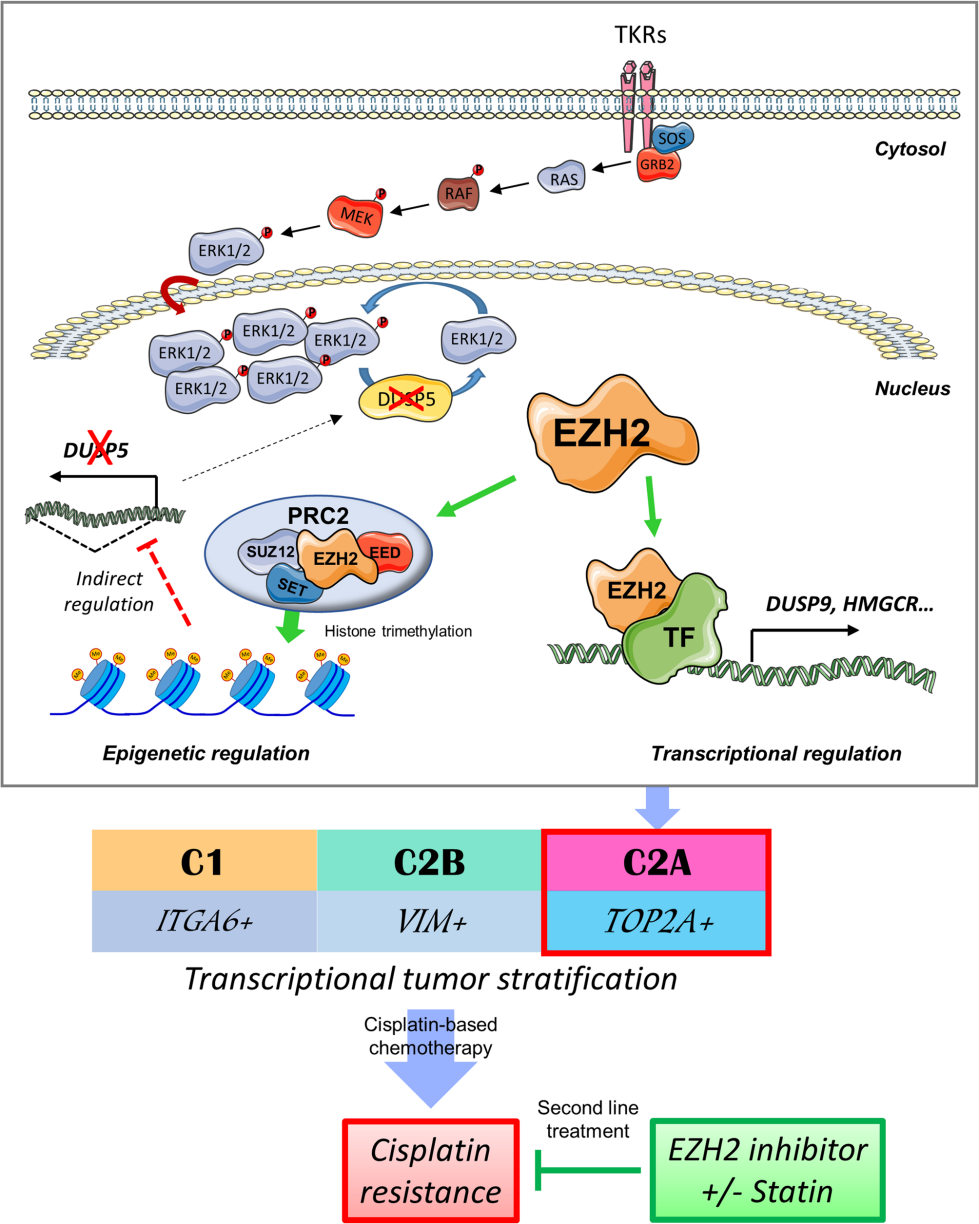
Graphical representation of the regulation of *DUSP9, HMGCR* and *DUSP5* genes by EZH2 in hepatoblastoma cells. EZH2 acts as a transcriptional activator of *DUSP9 and HMGCR* expressions likely through its transcriptional co-factor function [20], but as an epigenetic repressor of *DUSP5*. DUSP5 phosphatase negatively regulates ERK1/2-dependent MAPK pathway. *EZH2* and DUSP9 upregulations in HB are correlated with *TOP2A* expression, while TOP2A protein is a specific marker of C2A group [10]. EZH2 inhibitor and statin could be combined with cisplatin in second-line treatment for patients with a cisplatin-resistant tumor. TF: transcriptional factor; TKRs: tyrosine kinase receptors

Molecularly, we showed that EZH2 supports HB development by differentially regulating *DUSP5* and *DUSP9*, two MKPs highly involved in tumorigenic processes [33, 34]. DUSP9 phosphatase has been reported to dephosphorylate ERK1/2, p38, JNK and ASK1 kinases and to act as a tumor suppressor in many cancers, except in liver malignancies in which its role is still a matter of debate [33]. In our hands, DUSP9 was poorly oncogenic, and it unexpectedly sustained MAPK/ERK signaling, strongly suggesting its indirect and positive regulation of ERK1/2 phosphorylation in HB cells. In connection with this, we recently documented the co-activation of the Wnt/β-catenin and MAPK/ERK signaling pathways in HB cells [14], paving the way for combinatory therapies using cisplatin and sorafenib for the treatment of HB patients with high-risk tumors or in relapse [47, 48]. Like *EZH2*,* DUSP9* expression positively correlated with *TOP2A* expression and several poor prognostic factors in HB including tumor recurrence and patients’ survival and death. These data are in agreement with a previous report showing that *DUSP9*, which belongs to the 16-gene signature, is upregulated in the C2 group that has an unfavorable HB prognosis [6]. Here, we show for the first time that EZH2 regulates *DUSP9* positively at the protein and mRNA levels. Because the EZH2 inhibitor GSK126 promoted the expression of DUSP9 protein (and of EZH2 protein) (Fig. 6b) we believe that EZH2 supports *DUSP9* expression in HB cells at a transcriptional level through a PRC2-independent mechanism (Fig. 8) [19, 20], likely through a direct interaction with β-catenin [24].

Unlike *DUSP9* and in agreement with published data in adult gastrointestinal cancers [49–52], our data reveal a negative association between *EZH2* and *DUSP5* in HB cells. Indeed, both *DUSP5* mRNA and protein increased in HB cells when *EZH2* expression was silenced, and *DUSP5* transcript was inversely correlated with *EZH2* transcript in HB tissues (Fig. 4a-d and Supplementary Fig. S25 and Table S4). Moreover, the forced expression of EZH2 induced a decrease in *DUSP5* mRNA and protein in Huh6 cells, while the methyl transferase-dead H689A mutated variant EZH2* had no effect (Supplementary Fig. S23a-b), and the forced expression of DUSP5 antagonized the pro-oncogenic effects of EZH2 both in vitro and in vivo (Supplementary Fig. S30-31). Finally, the treatment of HB cells with EZH2 inhibitor GSK126 induced the expression of DUSP5 protein, while the trimethylation of histone H3 decreased (Fig. 6b). As the *DUSP5* gene was not marked by H3K27me3 in Huh6 cells (Supplementary Fig. S23c), we propose that EZH2 suppresses *DUSP5* expression in HB cells through an indirect, yet unidentified mechanism (Fig. 8).

*DUSP5* transcript was decreased in all analyzed HB datasets and particularly in proliferative C2A/C2 tumors, and in Epi-CB and high 14q32 subgroups [10, 11]. Moreover, its expression was not associated with survival but it correlated with tumor recurrence and dead patients. *DUSP5* is known to be a key tumor suppressor gene in many cancers (including liver malignancies) and its main target is ERK1/2 [33, 34]. In line with these studies, we showed that DUSP5 induces a dephosphorylation of ERK in Huh6 cells (Fig. 5f). Moreover, we recently demonstrated that *DUSP5* expression is induced by the forced expression of the transcriptional factor *LHX2*, which acts as a tumor suppressor in liver and blocks HB and hepatocellular carcinoma development by inhibiting both the Wnt/β-catenin and the MAPK pathways [14]. At this stage, the link between LHX2, DUSP5 and EZH2 in HB remains elusive, so new investigations are needed to clarify this issue. Interestingly, *DUSP5* expression correlated inversely with *EZH2*,* DUSP9* and *TOP2A* expression in most HB samples and was associated with disease recurrence. While *DUSP5* transcript was particularly decreased in the Epi-CB, high 14q32 and MRS3 groups, *EZH2* and *DUSP9* transcripts were highly expressed in the same groups. Importantly, the epigenetic cluster Epi-CB, which is associated with a poor prognosis in patients with HB, is characterized by the global hypomethylation and hypermethylation of CpG islands compared to Epi-CA, and a strong 14q32-gene signature [11]. All these data suggest a strong genomic and molecular connection between *EZH2*, *DUSP5* and *DUSP9* genes that remains to be clarified. Altogether, our data confirm the tumor suppressive role of DUSP5 in cancer by blocking the MAPK pathway and reveal a strong connection between *DUSP5* gene, its repression by EZH2, and the response of HB cells to cisplatin.

As shown for *DUSP9*, we also found a positive regulation of the *HMGCR* gene by EZH2 in HB cells, likely through a transcriptional PRC2-independent mechanism. First, the protein level of HMGCR declined in HB cells silenced for *EZH2*. Second, *HMGCR* transcript positively correlated with *EZH2* mRNA expression in most HB datasets. Finally, HMGCR and EZH2 proteins increased following GSK126 treatment (Figs. 6b and 7b), suggesting an enhancement of EZH2 transactivating functions as a consequence of its GSK126-induced increase and its interaction with TCF/β-catenin complex [20, 24, 53]. We also showed that the number of intracellular lipid droplets decreased in *EZH2*-silenced Huh6 cells compared to control cells (Supplementary Fig. S34b), while lipid droplets accumulated in the cytoplasm of GSK126-treated HB cells (Fig. 7c). Interestingly, Yang et al. reported the accumulation of lipids in GSK343-treated hepatocellular carcinoma cells through the activation of sterol regulatory element-binding protein 2 (SREBP2) and p38α [38] and Zheng et al. showed the contribution of EZH2 and SREBP2 targets *LDLR*, *FDFT1*, and *HMGCR* in the resistance of ovarian cancer cells to cisplatin [54]. Together, these data suggest that EZH2 positively regulates lipid metabolism in HB cells in part through *HMGCR* induction.

Based on recent reports showing the *de novo* synthesis of cholesterol in GSK126-treated tumor cells [39, 40], we combined an EZH2 inhibitor (GSK126 or EPZ6438) with a statin (atorvastatin or simvastatin) and found a synergistic anti-tumoral effect of combining these two drugs on HB cell survival, migration and clonogenicity. Addition of cisplatin to the combination further accelerated the elimination of HB cells. Moreover, statins significantly increased the sensitivity of tumor cells to GSK126, including two HB PDX cell lines and several non-HB childhood and adult tumoral cell lines (Supplementary Fig. S43). To finish, we further demonstrated the benefit of combining GSK126 and atorvastatin to inhibit HB development using a xenograft model of HB in mice, paving the way for EZH2 inhibitor-statin combinatory therapy for the treatment of HB patients with tumors presenting high amounts of EZH2, DUSP9 and TOP2A, including those in relapse.

To conclude, our findings demonstrate the oncogenic role of EZH2 methyl transferase in HB tumorigenesis in children and show that the PRC2-component EZH2 is crucial for the maintenance of HB hallmarks, MAPK signaling activity, tumor aggressiveness and cisplatin resistance. We also found a differential regulation of the genes *DUSP5*, *DUSP9* and *HMGCR* in HB cells through opposite transcriptional mechanisms related to the functional diversity of EZH2 protein and its methyl transferase activity (Fig. 8) [19, 20, 46]. We also confirm the activation of ERK1/2 kinase-dependent signaling in HB cells already displaying an aberrant Wnt signal, and found that DUSP5 phosphatase was partly responsible for its negative regulation (Fig. 8). The overall *DUSP5* downregulation observed in HB samples, which seems to be partly mediated by EZH2, therefore feeds the tumorigenic processes observed in HB and participates in cisplatin resistance. Thus, our work opens novel perspectives for future investigations in HB to find new drug combinations that act on the epigenetic mechanisms [17], as well as ERK, Wnt and mevalonate pathways which might significantly enhance the overall survival of children presenting with a high-risk, aggressive, recurrent and/or chemoresistant HB.

## Supplementary Information

Additional file 1.

Additional file 2.

## Data Availability

No datasets were generated during the current study. Transcriptomic data and datasets used and analyzed in this study are available in the NCBI's Gene Expression Omnibus (https://www.ncbi.nlm.nih.gov/geo) and R2: Genomics analysis and visualization platform (https://r2.amc.nl) repositories. All data supporting the conclusions of this article are included within the article and its supplementary files. Any other information is available from the corresponding authors upon reasonable request.

## Abbreviations

ANOVA: analysis of variance
CAM: chick chorioallantoic membrane
CTRL: control
DUSP: dual specificity protein phosphatases
DUSP5: dual specificity protein phosphatase 5
DUSP9: dual specificity protein phosphatase 9
ERK: extracellular signal-regulated kinase
EZH2: enhancer of zest homologue 2
GSEA: Gene Set Enrichment Analysis
HB: hepatoblastoma
H&E: hematoxylin-eosin
HMGCR: 3-hydroxy-3-methylglutaryl coenzyme A-CoA reductase
IHC: immunohistochemistry
MAPK/ERK: mitogen‐activated protein kinase/extracellular signal‐regulated kinase
MKP: mitogen-activated protein kinase phosphatase
mRNA: messenger RNA
MRS: Molecular Risk Stratification
NS: not significant
NT: non-tumoral
PDX: patient-derived xenograft
PRC2: polycomb repressive complex 2
RT-qPCR: reverse transcription quantitative polymerase chain reaction
SD: standard deviation
siRNA: small interfering RNA
TOP2A: topoisomerase 2-alpha
WT: wild-type

## References

[1] Hager J, Sergi CM. Hepatoblastoma. Sergi CM, editor Liver Cancer Brisbane (AU). Exon; 2021.

[2] Meyers RL, Maibach R, Hiyama E, Haberle B, Krailo M, Rangaswami A et al. Risk-stratified staging in paediatric hepatoblastoma: a unified analysis from the Children’s Hepatic tumors International Collaboration. The lancet oncology 2016. 10.1016/S1470-2045(16)30598-8 PMC5650231 27884679

[3] Zsiros J, Brugieres L, Brock P, Roebuck D, Maibach R, Zimmermann A, et al. Dose-dense cisplatin-based chemotherapy and surgery for children with high-risk hepatoblastoma (SIOPEL-4): a prospective, single-arm, feasibility study. Lancet Oncol. 2013;14:834–42. 23831416 10.1016/S1470-2045(13)70272-9 PMC3730732

[4] Czauderna P, Lopez-Terrada D, Hiyama E, Haberle B, Malogolowkin MH, Meyers RL. Hepatoblastoma state of the art: pathology, genetics, risk stratification, and chemotherapy. Curr Opin Pediatr. 2014;26:19–28. 24322718 10.1097/MOP.0000000000000046

[5] Buendia MA. Unravelling the genetics of hepatoblastoma: few mutations, what else? J Hepatol. 2014;61:1202–4. 25239079 10.1016/j.jhep.2014.09.016

[6] Cairo S, Armengol C, De Reynies A, Wei Y, Thomas E, Renard CA, et al. Hepatic stem-like phenotype and interplay of Wnt/beta-catenin and Myc signaling in aggressive childhood liver cancer. Cancer Cell. 2008;14:471–84. 19061838 10.1016/j.ccr.2008.11.002

[7] Jia D, Dong R, Jing Y, Xu D, Wang Q, Chen L, et al. Exome sequencing of hepatoblastoma reveals novel mutations and cancer genes in the Wnt pathway and ubiquitin ligase complex. Hepatology. 2014;60:1686–96. 24912477 10.1002/hep.27243

[8] Eichenmuller M, Trippel F, Kreuder M, Beck A, Schwarzmayr T, Haberle B, et al. The genomic landscape of hepatoblastoma and their progenies with HCC-like features. J Hepatol. 2014;61:1312–20. 25135868 10.1016/j.jhep.2014.08.009

[9] Sumazin P, Chen Y, Trevino LR, Sarabia SF, Hampton OA, Patel K et al. Genomic analysis of hepatoblastoma identifies distinct molecular and prognostic subgroups. Hepatology 2016. 10.1002/hep.28888 27775819

[10] Hooks KB, Audoux J, Fazli H, Lesjean S, Ernault T, Dugot-Senant N, et al. New insights into diagnosis and therapeutic options for proliferative hepatoblastoma. Hepatology. 2018;68:89–102. 29152775 10.1002/hep.29672

[11] Carrillo-Reixach J, Torrens L, Simon-Coma M, Royo L, Domingo-Sabat M, Abril-Fornaguera J, et al. Epigenetic footprint enables molecular risk stratification of hepatoblastoma with clinical implications. J Hepatol. 2020;73(2):328–41. 32240714 10.1016/j.jhep.2020.03.025 PMC12452110

[12] Sumazin P, Chen Y, Trevino LR, Sarabia SF, Hampton OA, Patel K, et al. Genomic analysis of hepatoblastoma identifies distinct molecular and prognostic subgroups. Hepatology. 2017;65:104–21. 27775819 10.1002/hep.28888

[13] Hirsch TZ, Pilet J, Morcrette G, Roehrig A, Monteiro BJE, Molina L, et al. Integrated genomic analysis identifies driver genes and Cisplatin-resistant progenitor phenotype in pediatric liver cancer. Cancer Discov. 2021;11:2524–43. 33893148 10.1158/2159-8290.CD-20-1809 PMC8916021

[14] Mosca N, Khoubai FZ, Fedou S, Carrillo-Reixach J, Caruso S, Del Rio-Alvarez A, et al. LIM homeobox-2 suppresses hallmarks of adult and pediatric liver cancers by inactivating MAPK/ERK and Wnt/Beta-Catenin pathways. Liver Cancer. 2022;11:126–40. 35634422 10.1159/000521595 PMC9109075

[15] Ghousein A, Mosca N, Cartier F, Charpentier J, Dupuy JW, Raymond AA, et al. MiR-4510 blocks hepatocellular carcinoma development through RAF1 targeting and RAS/RAF/MEK/ERK signalling inactivation. Liver Int. 2020;40:240–51. 31612616 10.1111/liv.14276

[16] Han ZG. Mutational landscape of hepatoblastoma goes beyond the Wnt-beta-catenin pathway. Hepatology. 2014;60:1476–8. 25078725 10.1002/hep.27347

[17] Claveria-Cabello A, Herranz JM, Latasa MU, Arechederra M, Uriarte I, Pineda-Lucena A, et al. Identification and experimental validation of druggable epigenetic targets in hepatoblastoma. J Hepatol. 2023;79:989–1005. 37302584 10.1016/j.jhep.2023.05.031

[18] Yamagishi M, Uchimaru K. Targeting EZH2 in cancer therapy. Curr Opin Oncol. 2017;29:375–81. 28665819 10.1097/CCO.0000000000000390

[19] Gan L, Yang Y, Li Q, Feng Y, Liu T, Guo W. Epigenetic regulation of cancer progression by EZH2: from biological insights to therapeutic potential. Biomark Res. 2018;6:10. 29556394 10.1186/s40364-018-0122-2 PMC5845366

[20] Liu Y, Yang Q. The roles of EZH2 in cancer and its inhibitors. Med Oncol. 2023;40:167. 37148376 10.1007/s12032-023-02025-6 PMC10162908

[21] Schlachter K, Gyugos M, Halasz J, Lendvai G, Baghy K, Garami M, et al. High tricellulin expression is associated with better survival in human hepatoblastoma. Histopathology. 2014;65:631–41. 24735023 10.1111/his.12436

[22] Wang Y, Xiao Y, Chen K, Chen S, Zhang M, Wu Z, et al. Enhancer of Zeste homolog 2 depletion arrests the proliferation of hepatoblastoma cells. Mol Med Rep. 2016;13:2724–8. 26848027 10.3892/mmr.2016.4864

[23] Zhang Y, Solinas A, Cairo S, Evert M, Chen X, Calvisi DF. Molecular mechanisms of hepatoblastoma. Semin Liver Dis. 2021;41:28–41. 33764483 10.1055/s-0040-1722645 PMC8524782

[24] Glaser K, Schepers EJ, Zwolshen HM, Lake CM, Timchenko NA, Karns RA, et al. EZH2 is a key component of hepatoblastoma tumor cell growth. Pediatr Blood Cancer. 2023. 10.1002/pbc.30774. 37990130 10.1002/pbc.30774 PMC10842061

[25] Hiyama E. Gene expression profiling in hepatoblastoma cases of the Japanese Study Group for Pediatric Liver Tumors-2 (JPLT-2) trial. Science Repository OU; 2019. 2019-02-12.

[26] Valanejad L, Cast A, Wright M, Bissig KD, Karns R, Weirauch MT, et al. PARP1 activation increases expression of modified tumor suppressors and pathways underlying development of aggressive hepatoblastoma. Commun Biol. 2018;1:67. 30271949 10.1038/s42003-018-0077-8 PMC6123626

[27] Edgar R, Domrachev M, Lash AE. Gene expression omnibus: NCBI gene expression and hybridization array data repository. Nucleic Acids Res. 2002;30:207–10. 11752295 10.1093/nar/30.1.207 PMC99122

[28] Subramanian A, Tamayo P, Mootha VK, Mukherjee S, Ebert BL, Gillette MA, et al. Gene set enrichment analysis: a knowledge-based approach for interpreting genome-wide expression profiles. Proc Natl Acad Sci U S A. 2005;102:15545–50. 16199517 10.1073/pnas.0506580102 PMC1239896

[29] Kim J, Lee Y, Lu X, Song B, Fong KW, Cao Q, et al. Polycomb- and Methylation-Independent roles of EZH2 as a transcription activator. Cell Rep. 2018;25:2808–20. e2804. 30517868 10.1016/j.celrep.2018.11.035 PMC6342284

[30] Indersie E, Hooks KB, Capdevielle C, Fabre M, Dugot-Senant N, Desplat A, et al. Tracking cellular and molecular changes in a species-specific manner during experimental tumor progression in vivo. Oncotarget. 2018;9:16149–62. 29662633 10.18632/oncotarget.24598 PMC5882324

[31] Indersie E, Lesjean S, Hooks KB, Sagliocco F, Ernault T, Cairo S, et al. MicroRNA therapy inhibits hepatoblastoma growth in vivo by targeting beta-catenin and Wnt signaling. Hepatol Commun. 2017;1:168–83. 29404451 10.1002/hep4.1029 PMC5721429

[32] Puppe J, Opdam M, Schouten PC, Jozwiak K, Lips E, Severson T, et al. EZH2 is overexpressed in BRCA1-like breast tumors and predictive for sensitivity to High-Dose Platinum-Based chemotherapy. Clin Cancer Res. 2019;25:4351–62. 31036541 10.1158/1078-0432.CCR-18-4024

[33] Khoubai FZ, Grosset CF. DUSP9, a dual-specificity phosphatase with a key role in cell biology and human diseases. Int J Mol Sci. 2021;22:1–21. 10.3390/ijms222111538 PMC8583968 34768967

[34] Seternes OM, Kidger AM, Keyse SM. Dual-specificity MAP kinase phosphatases in health and disease. Biochimica et Biophysica Acta (BBA). 2019;1866:124–43. 10.1016/j.bbamcr.2018.09.002 PMC6227380 30401534

[35] Gao SB, Xu B, Ding LH, Zheng QL, Zhang L, Zheng QF, et al. The functional and mechanistic relatedness of EZH2 and Menin in hepatocellular carcinoma. J Hepatol. 2014;61:832–9. 24845612 10.1016/j.jhep.2014.05.015

[36] Stelloo S, Nevedomskaya E, Kim Y, Schuurman K, Valle-Encinas E, Lobo J, et al. Integrative epigenetic taxonomy of primary prostate cancer. Nat Commun. 2018;9:4900. 30464211 10.1038/s41467-018-07270-2 PMC6249266

[37] Fioravanti R, Stazi G, Zwergel C, Valente S, Mai A. Six Years (2012–2018) of Researches on Catalytic EZH2 Inhibitors: The Boom of the 2‐Pyridone Compounds. Chem Rec. 2018;18(12):1818–32. 30338896 10.1002/tcr.201800091 PMC7410397

[38] Yang PM, Hong YH, Hsu KC, Liu TP. P38alpha/S1P/SREBP2 activation by the SAM-competitive EZH2 inhibitor GSK343 limits its anticancer activity but creates a druggable vulnerability in hepatocellular carcinoma. Am J Cancer Res. 2019;9:2120–39. 31720078 PMC6834481

[39] Xu X, Chen J, Li Y, Yang X, Wang Q, Wen Y, et al. Targeting epigenetic modulation of cholesterol synthesis as a therapeutic strategy for head and neck squamous cell carcinoma. Cell Death Dis. 2021;12:482. 33986254 10.1038/s41419-021-03760-2 PMC8119982

[40] Rahal F, Capdevielle C, Rousseau B, Izotte J, Dupuy JW, Cappellen D, et al. An EZH2 blocker sensitizes histone mutated diffuse midline glioma to cholesterol metabolism inhibitors through an off-target effect. Neuro-Oncol Adv. 2022;4:vdac018. 10.1093/noajnl/vdac018 PMC8923007 35300150

[41] Alipour Talesh G, Trezeguet V, Merched A. Hepatocellular Carcinoma Statins Biochem. 2020;59:3393–400. 10.1021/acs.biochem.0c00476 32865979

[42] Alannan M, Fayyad-Kazan H, Trezeguet V, Merched A. Targeting lipid metabolism in liver cancer. Biochemistry. 2020;59:3951–64. 32930581 10.1021/acs.biochem.0c00477

[43] Han M, Bushong EA, Segawa M, Tiard A, Wong A, Brady MR et al. Spatial mapping of mitochondrial networks and bioenergetics in lung cancer. Nature 2023: 615, 712-719. 10.1038/s41586-023-05793-3 10.1038/s41586-023-05793-3 PMC10033418 36922590

[44] Hajosi-Kalcakosz S, Dezso K, Bugyik E, Bodor C, Paku S, Pavai Z, et al. Enhancer of Zeste homologue 2 (EZH2) is a reliable immunohistochemical marker to differentiate malignant and benign hepatic tumors. Diagn Pathol. 2012;7:86. 22809481 10.1186/1746-1596-7-86 PMC3436720

[45] Chen Y, Lin MC, Yao H, Wang H, Zhang AQ, Yu J, et al. Lentivirus-mediated RNA interference targeting enhancer of Zeste homolog 2 inhibits hepatocellular carcinoma growth through down-regulation of stathmin. Hepatology. 2007;46:200–8. 17596871 10.1002/hep.21668

[46] Duan R, Du W, Guo W. EZH2: a novel target for cancer treatment. J Hematol Oncol. 2020;13:104. 32723346 10.1186/s13045-020-00937-8 PMC7385862

[47] Eicher C, Dewerth A, Kirchner B, Warmann SW, Fuchs J, Armeanu-Ebinger S. Treatment effects of the multikinase inhibitor Sorafenib on hepatoblastoma cell lines and xenografts in NMRI-Foxn1 *Nu* mice. Liver Int. 2012;32:574–81. 22176637 10.1111/j.1478-3231.2011.02729.x

[48] Eicher C, Dewerth A, Thomale J, Ellerkamp V, Hildenbrand S, Warmann SW, et al. Effect of Sorafenib combined with cytostatic agents on hepatoblastoma cell lines and xenografts. Br J Cancer. 2013;108:334–41. 23257893 10.1038/bjc.2012.539 PMC3566826

[49] Ma Z, Gao X, Shuai Y, Wu X, Yan Y, Xing X, et al. EGR1-mediated linc01503 promotes cell cycle progression and tumorigenesis in gastric cancer. Cell Prolif. 2021;54:e12922. 33145887 10.1111/cpr.12922 PMC7791171

[50] Liu B, Li J, Liu X, Zheng M, Yang Y, Lyu Q, et al. Long non-coding RNA HOXA11-AS promotes the proliferation HCC cells by epigenetically silencing DUSP5. Oncotarget. 2017;8:109509–21. 29312625 10.18632/oncotarget.22723 PMC5752538

[51] Ding J, Li J, Wang H, Tian Y, Xie M, He X, et al. Long noncoding RNA CRNDE promotes colorectal cancer cell proliferation via epigenetically silencing DUSP5/CDKN1A expression. Cell Death Dis. 2017;8:e2997. 28796262 10.1038/cddis.2017.328 PMC5596537

[52] Wang XY, Jian X, Sun BQ, Ge XS, Huang FJ, Chen YQ. LncRNA ROR1-AS1 promotes colon cancer cell proliferation by suppressing the expression of DUSP5/CDKN1A. Eur Rev Med Pharmacol Sci. 2020;24:1116–25. 32096171 10.26355/eurrev_202002_20162

[53] Kim KH, Roberts CW. Targeting EZH2 in cancer. Nat Med. 2016;22:128–34. 26845405 10.1038/nm.4036 PMC4918227

[54] Zheng L, Li L, Lu Y, Jiang F, Yang XA. SREBP2 contributes to cisplatin resistance in ovarian cancer cells. Exp Biol Med (Maywood). 2018;243:655–62. 29466876 10.1177/1535370218760283 PMC6582395

