# Supplementary material for: EZH2 is a key prognostic marker and therapeutic target in aggressive and proliferative hepatoblastoma
Source: Mol Cancer. 2026 Feb 23;25:77. doi: 10.1186/s12943-025-02474-9 (PMC13001322; doi:10.1186/s12943-025-02474-9)
Supplement: Supplementary file 1 — Additional file 1. [file 12943_2025_2474_MOESM1_ESM.docx]

**Supplementary Material and Methods**

*Bioinformatic analyses*

In this work we used transcriptomic datasets from Ikeda et al (gse131329, [1]), Carrillo-Reixach et al (gse133039, [2]), Lopez-Terrada et al (gse75271, [3]), Karns et al (gse81928, [4]), Buendia et al [5], Kappler et al (gse151347, [6]) and Raymond et al (gse104766, [7]). Data relative to these datasets are presented in Supplementary Table S1.

*Cell culture*

Human liver cancer cell lines HepG2 and Huh-7 were cultured in DMEM 4.5g/L (Gibco) containing 10% fetal bovine serum and penicillin/streptomycin (1,000 units/mL). Human liver cancer cell line Huh6 was cultured in DMEM 1g/L (Gibco) containing 10% fetal bovine serum and penicillin/streptomycin (1,000 units/mL). PDX-derived cell lines HB282 and HB303 were cultured in Advanced DMEM F12 (Gibco) containing 8% decomplemented fetal bovine serum, 1% L-Glutamine (2mM), penicillin/streptomycin (1,000 units/mL) and 20 µM Rho-associated kinase (ROCK) inhibitor Y-27632 (MCE, HY-10071). Human lung adenocarcinoma cell line NCI-H23 and human rhabdoid tumor of the kidney cell line WT-CLS1 were cultured in RPMI-1640 medium (Gibco) containing 10% fetal bovine serum and penicillin/streptomycin (1,000 units/mL). Human osteosarcoma cell lines HOS-MNNG and U2-OS were cultured in DMEM 1g/L (Gibco) containing 10% fetal bovine serum, 1% L-glutamine (2mM) and penicillin/streptomycin (1,000 units/mL). Cells were incubated at 37 °C in a humidified incubator with an atmosphere of 5% CO_2_. Authentication of all cell lines was carried out every year by STR profiling (Supplementary Table S5). Cells were tested weekly for mycoplasma-free infection.

*siRNA transfection*

Cells were transfected with siRNAs targeting EZH2 or DUSP9 (Supplementary Table S6) or with a control siRNA (AllStars Negative Control siRNA, Qiagen). The transfection was carried out in 6-well microplates with 250,000 cells per well. siRNAs were diluted in 1x siMAX dilution buffer (6mM HEPES, 20mM KCl, 0,2mM MgCl2, pH=7.3; Eurofins, Ebersberg, Germany). siRNAs were brought into contact with lipofectamine (RNAiMax Invitrogen) diluted to 1/100 in the transfection medium (OptiMEM, Gibco). A concentration of 20nM was used for each siRNA. The mixture was incubated for 20 minutes at room temperature to allow the formation of liposomes and then added to the cells incubated in an antibiotic-free medium for 6 hours. After transfection, the medium was replaced with fresh medium with antibiotics.

*Mutagenesis and plasmid construction*

The lentiviral plasmid pSIN-EF1alphaL-eGFP-IRES-Puro was used to carry out the cloning. Four inserts corresponding to WT EZH2, DUSP5 and DUSP9 and the mutated version of EZH2 (EZH2*) were cloned. Briefly, EZH2, DUSP5 and DUSP9 open reading frames were amplified by PCR using cDNAs ordered from SinoBiological as matrix. H689A mutant referred to as EZH2* was obtained by mutagenesis as described by Jung Kim *et al* [8]. PCR amplification was carried out with the primers described in Supplementary Table S6. The PCR product was cloned in the NheI/MluI sites of pSIN-EF1αL-eGFP-IRES-Puro vector and fully sequenced with the primers described in Supplementary Table S6 before being sent to the Vectorology core facility.

*Lentiviral production, titration, and cell transduction*

Production and titration of infectious lentiviral particles was done by the Vectorology platform VECT’UB. Procedures and policies were described previously by Maurel M et al, and Laloo B et al [9, 10]. The lentiviral particles were added to the cells and incubated for 24 hours. After that, the cells were washed twice with PBS and then cultured for a few days before experimental use. The ectopic expression of proteins was confirmed by western blot for each experiment. The Tomato transgene-expressing lentiviruses were provided by the Vectorology platform VECT’UB.

*Drug treatments and IC_50_ measurement*

Cisplatin (S1166), EPZ6438 (S7128), GSK126 (S7061), atorvastatin (S5715) and simvastatin (S1792) were purchased from SelleckChem. Cisplatin was dissolved with NaCl 0,9%. EPZ6438, GSK126, atorvastatin and simvastatin were dissolved with DMSO (dimethyl sulfoxide). Prior to use in cell assays, simvastatin was activated by NaOH in EtOH treatment. All drugs were stored at -20 °C.

For IC_50_ determination, a dose-response curve was generated for each cell line using increasing concentrations of the drug. The IC_50_ of each drug was calculated by determining the concentration of drug required to reduce of 50% the cell survival. For synergistic effect determination, a new dose-response curve was generated using increasing concentrations of drug A and a fixed dose of drug B (fdB) (as indicated in the corresponding graph), which has no growth inhibitory effect on the corresponding cells, as described by A. Palmer at the Laboratory of Systems Pharmacology, Havard Medical School (<https://images.app.goo.gl/a87wEK8snLyotE5i8>) and Shyr et al [11]. The combination of drug A with drug B was considered synergistic when the IC_50_ of drug A decreased of 25% or more when adding a fdB with no growth inhibitory effect. The formula used was “(IC_50_ drug A – IC_50_ drugs A+fdB) / IC_50_ drug A*100”.

*Cell proliferation and viability assays*

For 2D culture, 2,000 cells/well for Huh6 and 3,000 cells/well for HepG2 were seeded in 96-well microtiter plates. Cell growth was evaluated 24h, 48h, 72h or 96h after genetic manipulation and 48h or 72h after treatment using the in vitro MTS assay Kit or the Sulforhodamine B (SRB) assay (Sigma) according to the manufacturer’s instructions. For 3D culture, spheroids were formed with 10,000 cells per well in a low-adherence 96-well plate. In each well, the cells were mixed with 100 µl of media and 100 µl of methylcellulose at a final concentration of 0.5%. The plates were then incubated in the IncuCyte® S3 live cell analysis system (Essen BioScience) and scanned every 8 hours. For gene manipulation, transduction or depletion were done before plating. For the treatments, depending on the experiment and the drug, spheroids were treated at day 4 or 5 for 48h or 72h. Cell viability assay in spheroids was performed using 1 μM calcein AM (BioLegend) and 2 μM ethidium homodimer 1 (Sigma) for 30 minutes at 37°C. Spheroids were imaged by the IncuCyte® S3 live cell analysis system (Essen BioScience). For all cell viability assays, drugs were used at half maximal inhibitory concentration (IC_50_).

*Cell senescence*

For 2D culture, cells were seeded in a 24-well plate. Three days later, cells were fixed and beta-galactosidase activity was measured by senescence assay using the beta-Galactosidase Staining kit (Cell Signaling, Danvers, Massachusetts, USA) according to the manufacturer’s instructions. Cells were observed and imaged using an InCellis microscope (Bertin Technologies, France). Senescent cells were counted using ImageJ.

For 3D culture, spheroids were formed for four days in a low-adherence 96-well plate. After that, they were fixed and beta-galactosidase activity was measured by Senescence assay using the beta-Galactosidase Staining kit (Cell Signaling, Danvers, Massachusetts, USA) according to manufacturer’s instructions. Spheroids were observed and imaged by stereomicroscopy (SMZ745T) and camera (DS-Fi2) from Nikon.

*Apoptosis*

Apoptosis was measured in cells seeded in a low-adherence 96-well plate after genetic manipulation or treatment using caspase 3/7 activity assay (Promega Corp., Madison, WI, USA).

*Cell migration*

To study the effect of drugs on cell migration, Huh6 cells were plated in a 96-well plate at a density of 30,000 cells per well and incubated until attachment. The scratches were made using the Incucyte WoundMaker (96-pin woundmaking tool) and cells were washed to remove any cells in suspension. Immediately after, cells were treated for 24 h with DMSO (control: CTRL), GSK126 at IC25 dose (3 µM), statin (ATR: atorvastatin at 8 µM; SIM: simvastatin at 4 µM) or a combination of both. Cell migration was automatically monitored over time by scanning each well every hour for 24 hours using an IncuCyte® S3 live cell analysis system.

*Clonogenicity assay*

Huh6 and HepG2 cells were seeded at 500 and 1,000 cells/well, respectively, in 12-well plates. After attachment, cells were treated with DMSO (control: CTRL), GSK126 at IC25 dose (3 and 4 µM for Huh6 and HepG2 cells, respectively), statin (ATR: atorvastatin at 8 µM; SIM: simvastatin at 4 µM) or the combination of both. Following incubation at 37°C for 10 days, the cells were fixed with 4% paraformaldehyde (PFA). The colonies were stained with 0.05% crystal violet and plates were imaged using the Fusion FX imager (Vilber Lourmat).

*Red oil assay*

Huh6 or HepG2 cells were seeded at 250,000 cells/well in 6-well plates. After attachment, cells were treated with DMSO (control: CTRL) or GSK126 at IC50 dose (6 and 8 µM for Huh6 and HepG2 cells, respectively). Following incubation at 37°C for 48 hours, the cells were fixed with 4% PFA for 15 minutes then washed twice with PBS and stained with the red oil for 15 minutes at room temperature. Cells were washed several times and scanned using InCellis microscope (Bertin Technologies, France).

*RNA purification and real-time quantitative PCR analyses*

Total RNA extraction was done with the TRI Reagent (Sigma) according to the manufacturer’s instructions. For quantification of messenger RNA (mRNA) expression, total RNA was retrotranscribed using the Maxima Reverse Transcriptase (Thermo Scientific). Then, quantitative RT-PCR amplifications were performed in 12µL multiplex PCR reactions containing 1X SYBR® Premix Ex Taq™ (Takara Bio Europe). Forward and reverse primers were as described in Supplementary Table S6. The GAPDH mRNA served as internal control for normalization.

*Co-Immunoprecipitation, Western blotting and antibodies*

Co-IP experiments were performed using the DynabeadsTM Co-IP Kit (Invitrogen #14321D) according to the manufacturer's instructions. Cells were lysed and total proteins were extracted 48 hours after treatment or 72 hours after genetic manipulation using a mix of RIPA buffer (Sigma), protease inhibitor and phosphatase inhibitor cocktail (Roche). Co-IP experiments were performed using the DynabeadsTM Co-IP Kit (Invitrogen #14321D) according to the manufacturer's instructions. After protein quantification with BCA Protein Assays (ThermoFisher), 40ug of cell extracts were loaded in 4-15 % pre-casted gels (Bio-Rad) for migration. Then proteins were transferred onto nitrocellulose membranes (Transblot® Turbo midi-size, Bio Rad). The membranes were blocked with BSA 5% or Odyssey blocking buffer (LI-COR Biosciences) and detected with corresponding antibodies (Supplementary Table S7). Revelation was performed by chemiluminescence using Fusion FX (Vilber Lourmat) and quantification was done using ImageJ software (National Institutes of Health, Bethesda, Maryland, USA).

*Proteomics*

Proteomic analysis was performed in an Huh6 cell line depleted in EZH2 or control by the Proteomics Core Facility at the University of Bordeaux (<https://proteome.cgfb.u-bordeaux.fr/en>). All steps were done as described by Ghousein et al [12]. Proteomic data are presented in Supplementary Table S3.

*Spheroid preparation, transmission electron microscopy*

Spheroids were fixed with a 2% paraformaldehyde and 2.5% solution of glutaraldehyde in 0.15 M cacodylate buffer (pH 7.4) for 2 hours at room temperature (RT) and overnight at 4°C. A scanning electron microscopy preparation was applied. Spheroids were washed five times in cacodylate buffer (cacodylate buffer 0.15M + 2 mM of calcium chloride (CaCl2)) and stained with heavy metal with 2% osmium (OsO4), 1.5% potassium ferrocyanide in 0.15 M cacodylate buffer and 2 mM CaCl2 for 1 hour on ice in the dark. After staining, spheroids were quickly washed five times (2 of 1 min and 3 of 3 min) in ultrapure water. To increase the membrane contrast and lipid droplet detection, spheroids were further incubated for 20 min with 1% thiocarbohydrazide (TCH) at RT in the dark, and then washed as described above. An additional staining step was performed with 2% osmium for 30 min at RT followed by five additional washes. At this stage, spheroids were stored overnight at 4°C with 1% uranyl acetate, washed five times and incubated for 30 min with lead aspartate at 60°C before five additional washes. Next, the alcoholic dehydration of spheroids was performed at 4°C using incremental baths of ethanol (20%, 50%, 70%, 90% and 2x 100%, 5 min each) and three baths of 100% acetone (1 of 5 min at 4°C and 2 of 10 min at RT). Resin embedding was done using incremental resin epon-solvent mixes (25% fresh resin/ acetone, 50 % fresh resin/ acetone, 75% fresh resin/ acetone, each mix for 2 hours at RT). To finish, a bath of 100% fresh resin/acetone was done overnight at RT. The third day, impregnation in pure resin was performed for 5 hours at RT followed by inclusion of spheroids in fresh resin in reduced mold, with pieces of a plastic pipette and polymerization at 60°C for 48 h. Next, the excess resin around the spheroids was removed with a razor blade without damaging the samples. Finally, spheroids were positioned and glued on the top of a resin block to facilitate cutting by a diamond knife (DIATOME 35° 2mm).

The resin-embedded samples were cut with an ultramicrotome (UC7 Leica). 70-nm ultrathin sections were deposited on grids (G150HEX-CU, 3.05 mm, Delta Microscopy) and analyzed with a transmission electron microscope (Hitachi H7650).

ChIP-qPCR

Chromatin immunoprecipitation (ChIP) was performed using previously described protocol from Holliday et al. [13]. 15x10^6^ cells were cross-linked with 1% paraformaldehyde for 15 minutes at room temperature and quenched with 125 mM glycine. After washing in cold PBS supplemented with protease/phosphatase inhibitors, nuclear lysates were sonicated under on a Bioruptor Pico^®^ sonication device (Diagenode) for 5 cycles of 30 seconds, yielding DNA fragments ranging from 100 to 500 bp (Supplementary Figure S51). The supernatant was immuno-precipitated overnight at 4°C with the following antibodies: H3K27me3 (9733T, Cell Signaling) and isotype control IgG (3900S, Cell Signaling), both diluted at 1/100 (Supplementary Table S7). After elution and crosslink reversal, DNA was purified in 50 µl elution buffer with Nucleospin Gel and PCR clean up (Macherey Nagel). Purified DNA was used for qPCR to detect the enrichment of DNA fragments in promoter region of DUSP5. ChIP-qPCR values were expressed as percentage of input. ChIP-qPCR primers and reagents used for the assay are listed in Supplementary Table S6 and S8, respectively.

*Chick chorioallantoic membrane (CAM) assay*

Animal procedures were carried out as described before [14, 15] in agreement with the European (directive 2010/63/UE) and French (decree 2013-118) guidelines. Briefly, fertilized embryos were received at the stage of segmentation. Then, they were incubated at 37.4°C and 70% humidity. At day 3 of development, the eggshell was opened on the top and the opening sealed with medical-grade Durapore tape. At day 10 of embryonic development, 1 million Huh6 or HepG2 cells were embedded in Matrigel® (growth-factor reduced, Corning) droplets (40µL) and deposited on the CAM. Tumor growth was monitored by stereomicroscopy (SMZ745T) and pictures were taken using a camera (DS-Fi2) on day 1, 3 and 7. At day 7, all tumors were fixed with PFA 4%, extracted, and imaged using a cell phone. Then, the weight was measured using a precision balance and bleeding was evaluated as low or high.

*Xenopus embryo*

Batches of gastrula stage embryos (10 embryos by batch) were incubated in 24-well plates in presence of cisplatin or GSK126 at the indicated concentrations. Embryos were left in solution until untreated embryos (control) reached stage 41. Control or cisplatine-treated embryos were incubated in 0.1x Marc's Modified Ringer. GSK-126-treated embryos were incubated in 0.1x Marc's Modified Ringer supplemented with 0.1% DMSO.

*Tumor xenografts in mice*

NOD/LtSz-scid IL2R gamma null mice were bred in standard conditions compliant with regulations (Reference APAFIS #32917-2021121316283534 v2, n°A-32917, French government). Sterilized food and water were accessible ad libitum. One million Huh6 cells in 50% of Matrigel were subcutaneously injected in a total volume of 100 μl in the right flank of 8- to 9-week-old female mice (26–32 g). Mice body weight was measured three times a week. Tumor growth was measured with a caliper three times a week. When tumors reached an average volume of 250 mm3 (day 12, see arrow), mice were randomly divided in 5 groups and treated.

Cisplatin-treated mice received intraperitoneal (IP) injections of cisplatin (1.0 mg/kg in 200 μl/30 g body weight of saline with 5% final percentage of DMSO) twice a week. GSK126-treated mice received IP injections of GSK126 (50 mg/kg in 200 μl/30 g body weight of saline supplemented with 5% final percentage of DMSO) three times per week. Atorvastatin-treated mice received IP injections of atorvastatin (20 mg/kg in 200 μl/30 g body weight of saline supplemented with 5% final percentage of DMSO) three times per week. GSK126- and atorvastatin-treated mice received IP injections three times per week of the combo (50 mg/kg GSK126 and 20 mg/kg atorvastatin in 200 μl/30 g body weight of saline with 5% final percentage of DMSO). Control animals received IP injections of the same volume of saline supplemented with 5% DMSO on the same days. Mice were euthanized on day 28 when control tumors reached a size of 2,000 mm3. In parallel, blood was collected from euthanatized mice and ASAT, ALAT, creatinine, and urea were measured using a Pentra PC400 clinical chemistry analyzer (Horiba Medical, USA) to assess liver and kidney toxicity.

*Tumor xenografts in zebrafish*

Tomato positive Huh6 cells, expressing either LV-CTRL, LV-EZH2, LV-DUSP5 or LV-EZH2 + LV-DUSP5 (for all transductions, m.o.i. = 5), were injected into the Curvier canal of Casper (48HPf) zebrafishes (Xenofish platform). Tomato+ cells were monitored by fluorescence microscopy and pictures were taken at day 0 and day 2. Tumor growth was measured using tumor surface and the image J software, and normalized using a day 2/day 0 ratio.

*Immunohistochemistry (IHC)*

Patients’ samples were obtained with written informed consent and the study protocol was approved by the ethic committees of the French Government (BCT-Centre de Ressources biologiques, Hôpital Le Kremlin-Bicêtre, 94275 Le Kremlin-Bicêtre, France; reference number: B-CRB-DE-PSAD13-004-01; cession number: 2018-ACP-BCT-01R; BRIF number: BB-0033-00089; N°ID-RCB-A00180-49; CPP N°CO-15-003; CNIL N°915640; CCTIRS N°15.700; MESR N° DC2009-939; Supplementary Table S9). Liver samples were clinically, histologically, and genetically characterized (Supplementary Table S9). Liver or CAM tissues were immediately frozen in liquid nitrogen and stored at −80°C until used for molecular studies.

*Samples staining:* IHC analysis was performed on HB patient tissues or on fixed, and paraffin embedded CAM tissues. 2.5 μm thick microtome sections were stained with Eosin/hematoxylin or immuno-stained. First, sections were de-paraffinized and rehydrated, and antigen retrieval in a citrate buffer pH 6 solution (Ki67) or Tris-EDTA pH 9 solution (EZH2, DUSP9 and DUSP5). The sections were blocked using EnVisionTM Flex peroxidase-blocking reagent (SM801, Dako-Agilent, Santa Clara, CA, USA) to block endogenous peroxidase, then washed and incubated with mouse anti-Ki67 (1:100); rabbit anti-EZH2 (1:100); rabbit anti-DUSP9 (1:500) or rabbit anti-DUSP5 (1:50). Incubation in horseradish peroxidase (EnVision Flex/HRP, SM802, Dako-Agilent) was used for signal amplification. The slides were counterstained with hematoxylin, dehydrated and mounted. Each immunohistochemical run contained a negative control (buffer, no primary antibody). Sections were visualized with a Hamamatsu NANOZOOMER Digital slide scanner 2.0 HT at 20× magnification (Hammamatsu, Japan).

*Statistical analysis*

Statistical analyses were performed using GraphPad Prism 6.0 or 7.0 software. All data are displayed as the mean of at least three independent experiments and error bars indicate standard deviation (SD) of the mean. When it contained two unmatched groups of values, the nonparametric Mann-Whitney test was used for the comparison of means. When it contained two matched groups of values and depending on whether data were considered to follow a Gaussian distribution or not, the parametric *t*-test or the nonparametric Wilcoxon matched-pairs signed ranked test was used. When it contained three groups of values or more, the regular one-way analysis of variance (ANOVA). When it contained three groups of values or more and two experimental factors, the two-way ANOVA was used for the comparison of multiple means and conditions. One-way and two-way ANOVA tests were followed by Tukey’s or Sidak's multiple comparisons post-test, as indicated in the corresponding figure legend. When the experiment contained two groups of categorical variables, the two-tailed Chi-square test was used. When comparing the expression of two genes, we used the two-tailed Pearson R correlation test. Data are given as mean ± standard deviation. All correlative analyses performed in this work are presented in Supplementary Tables S2 and S4. For survival analysis, Kaplan-Meier’s method and the Log rank test were performed to compare differences between curves. Samples were categorized according to low and high expression of gene of interest according to tumor expression median. Results were considered significant when p < 0.05. For all data in figures, *: *p*< 0.05, **: *p*< 0.01, ***: *p*<0.001, ****: *p*<0.0001 or exact *p*-values where indicate.


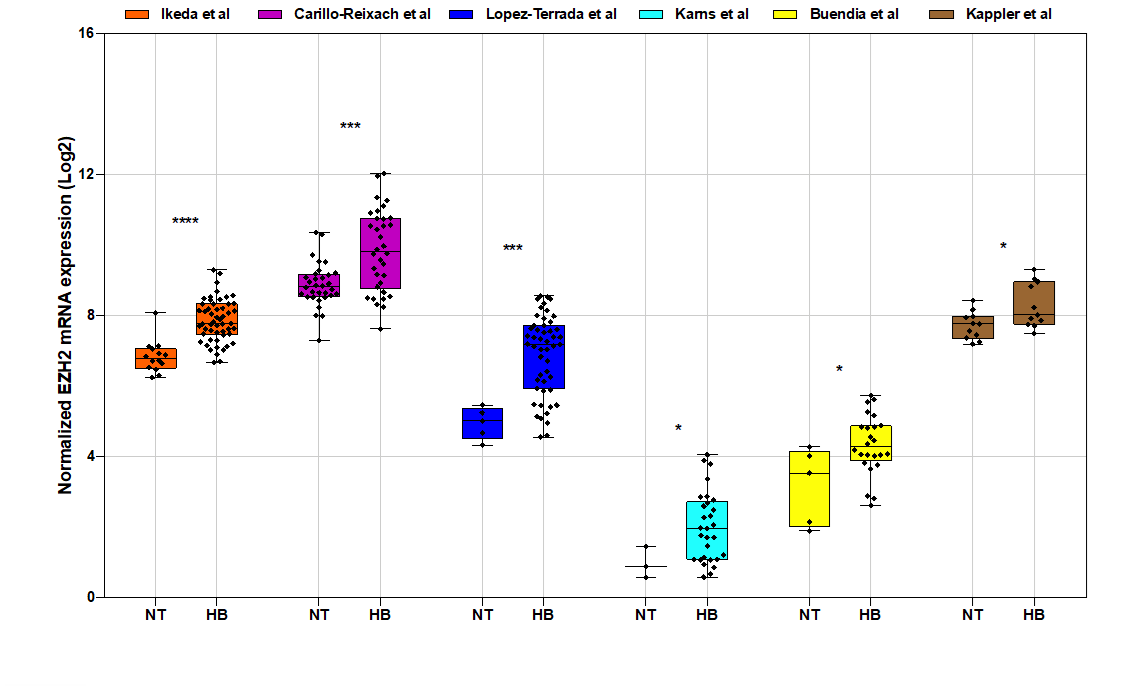


**Supplementary FIG. S1.** **Expression of *EZH2* mRNA in six hepatoblastoma datasets.** Expression of *EZH2* transcript in HB and non-tumoral (NT, in this figure and the following) samples from Ikeda’s dataset (gse131329, [1]), Carrillo-Reixach’s dataset (gse133039, [2]), Lopez-Terrada’s dataset (gse75271, [3]), Karns’s dataset (gse81928, [4]), Buendia’s dataset [5] and Kappler’s dataset (gse151347, [6]). Unpaired Mann & Whitney test. *p<0.05; ***p<0.001; ****p<0.0001.

**
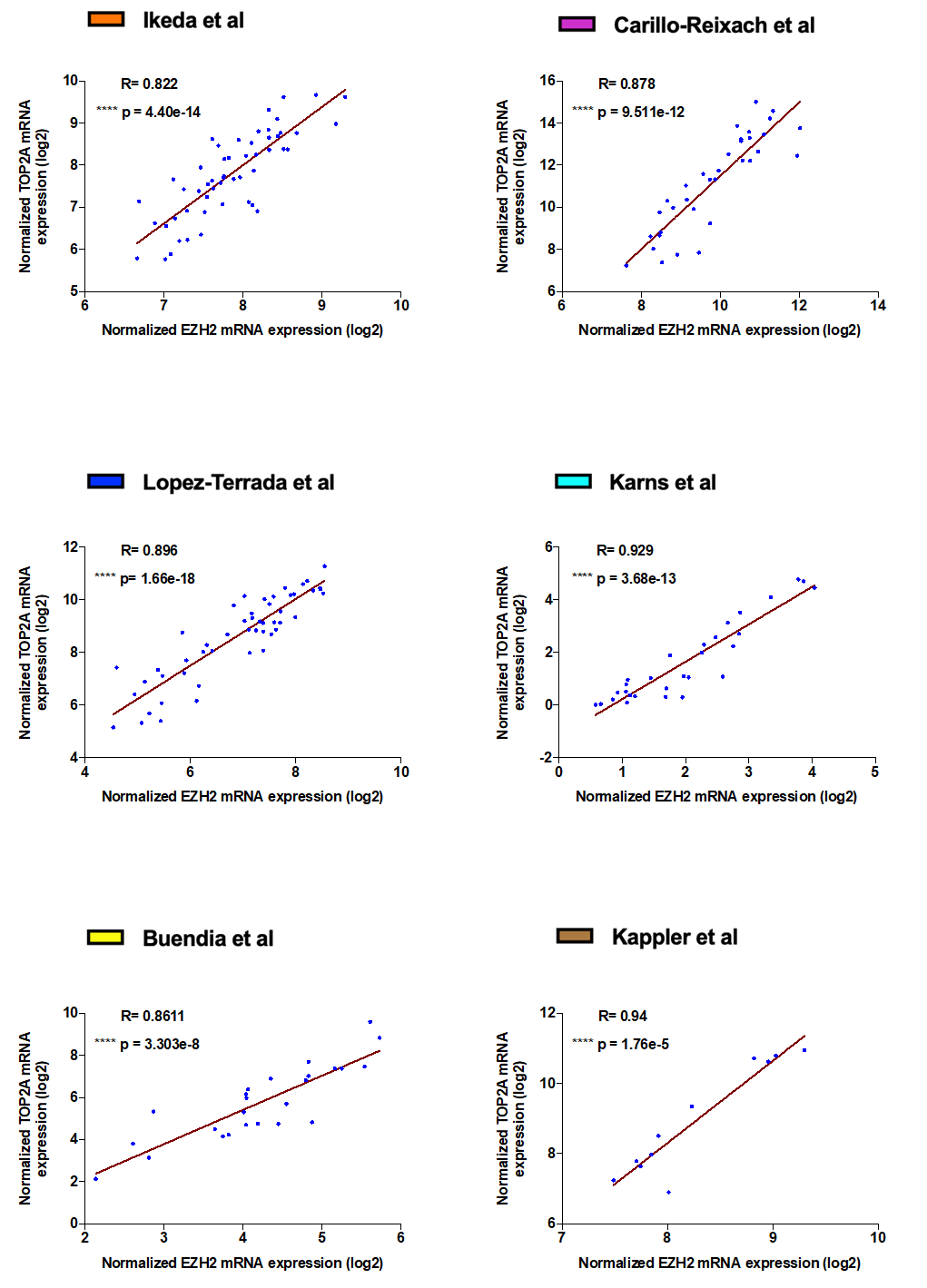
**

**Supplementary FIG. S2. Correlative analysis between *EZH2* and *TOP2A* transcripts in hepatoblastoma.** Graphs show the two-tailed Pearson R correlations between *EZH2* and *TOP2A* transcripts in HB samples from Ikeda’s dataset (gse131329, [1]), Carrillo-Reixach’s dataset (gse133039, [2]), Lopez-Terrada’s dataset (gse75271, [3]), Karns’s dataset (gse81928, [4]), Buendia’s dataset [5] and Kappler’s dataset (gse151347, [6]). For each dataset, the R and p-values are as shown in the corresponding graph.


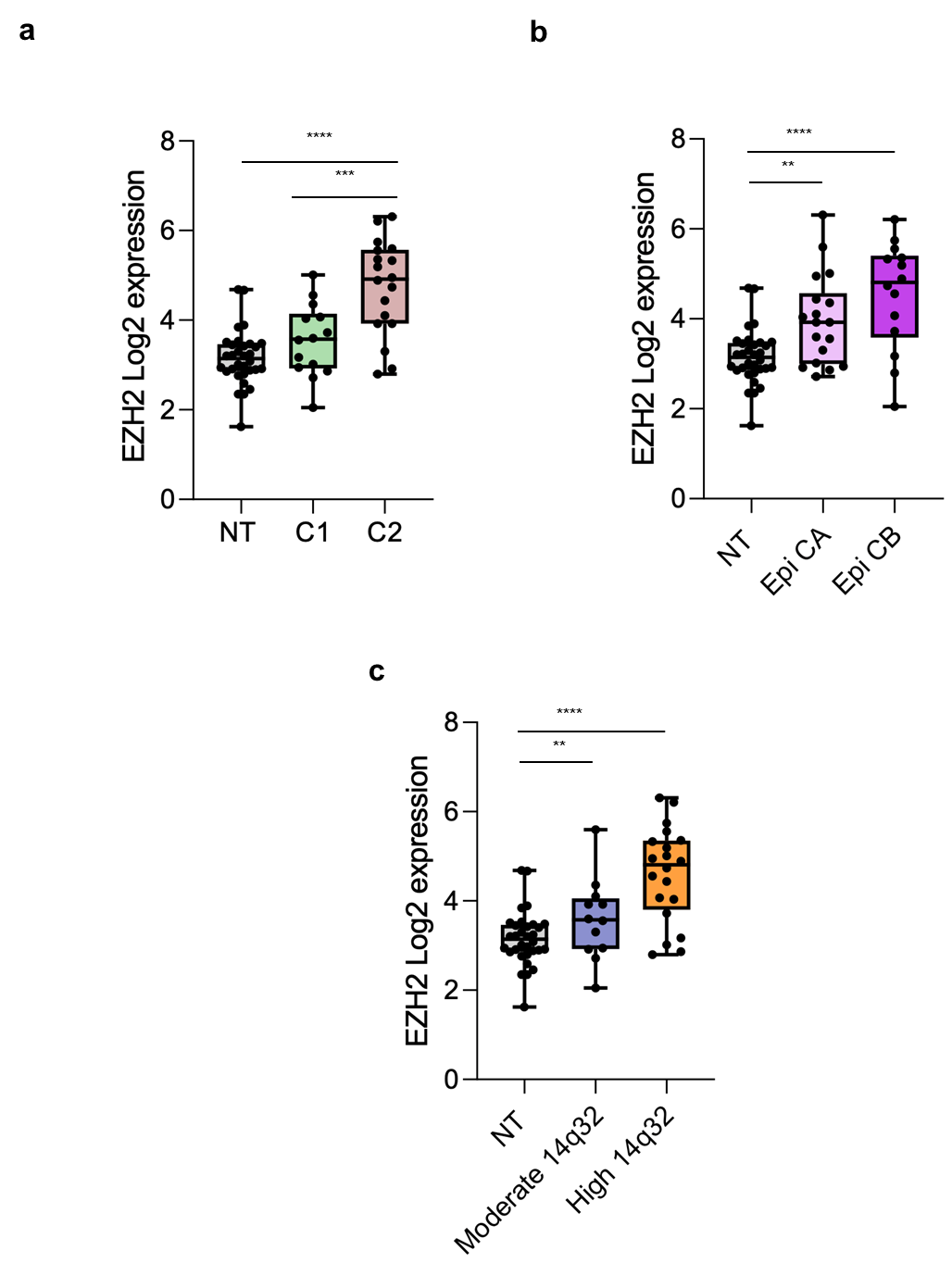


**Supplementary FIG. S3. Expression of *EZH2* mRNA in hepatoblastoma and correlative study.** (**a-c**) Expression levels of *EZH2* mRNA in NT (n=32), C1 (n=14) and C2 tumors (n=18) (**a**, Buendia’s dataset, [5]), in NT (n=32), Epi CA (n = 18) and Epi CB (n=14) (**b**) or in NT (n=32), moderate (n=12) and high 14q32 expression tumors (n=20) (**c**). (**a-c**) One-way ANOVA test; Tukey post-test. **p<0.01; ***p<0.001; ****p<0.0001.

**
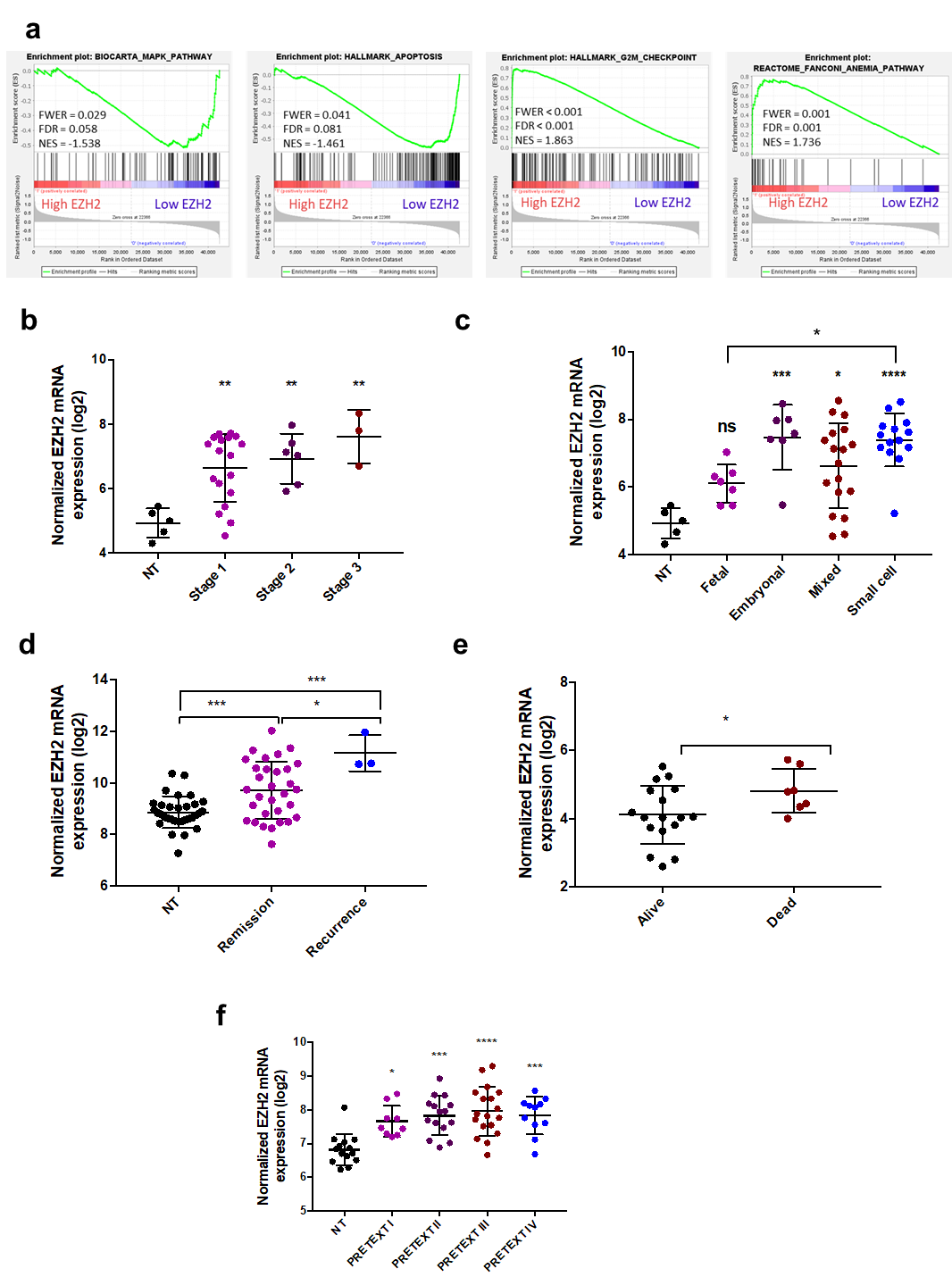
**

**Supplementary FIG. S4. Expression of *EZH2* mRNA in hepatoblastoma according to clinical, histological, or radiological features.** (**a**) GSEA of *EZH2* mRNA low- and high-expression samples categorized according to tumor median. FDR q value<0.25 indicates statistically significant trend. NES, Normalized Enrichment Score. Left in red, high expression of *EZH2* mRNA; Right in blue, low expression of *EZH2* mRNA. (**b-f**) Correlative analyses between *EZH2* transcript expression and clinical, histological or radiological features [**b**, Stage (gse75271, [3]); **c**, Histopathological subtypes as indicated (gse75271, [3]); **d**, Remission and recurrence (gse133039, [2]; **e**, Alive and deceased patients (MAS5.0 - u133a, Buendia et al dataset [5]; **f**, PRETEXT staging system (gse131329, [1]); One-way ANOVA, **p<0.01 to ****p<0.0001, Sidak's multiple comparisons post-test for **b**, **c**, **d** and **f**; Unpaired Mann Whitney test for **e**]. ns, not significant; *p<0.05; **p<0.01; ***p<0.001; ****p<0.0001.


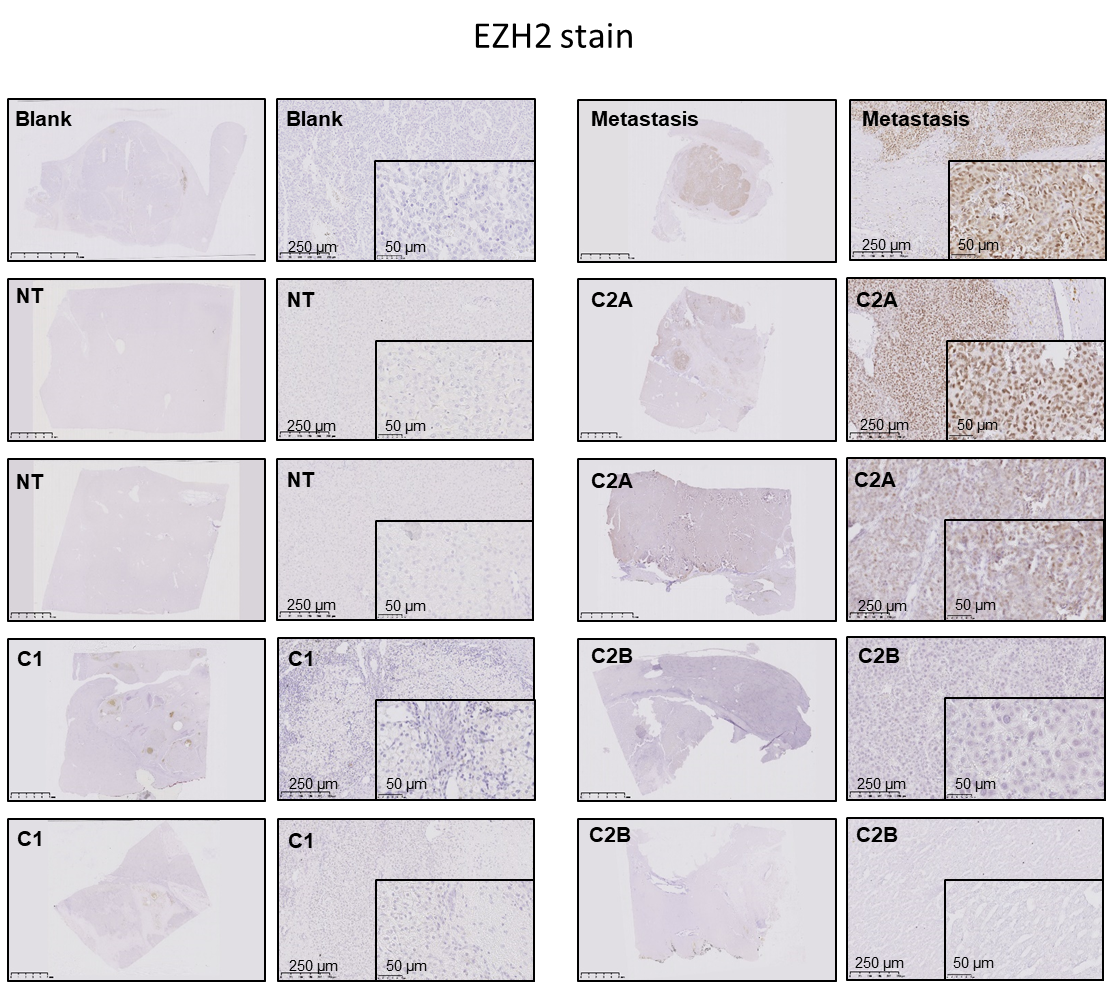


**Supplementary FIG. S5. Expression of EZH2 protein in hepatoblastoma patient tissues.** Immunoshistochemical stains of HB patient tissues classed as NT, C1, C2A, C2B and a lung metastasis. Nine representative samples, one from a metastasis and two from each other subgroup, were stained using an antibody against EZH2. Blank: control stain with no primary antibody. Scale bars are as indicated.


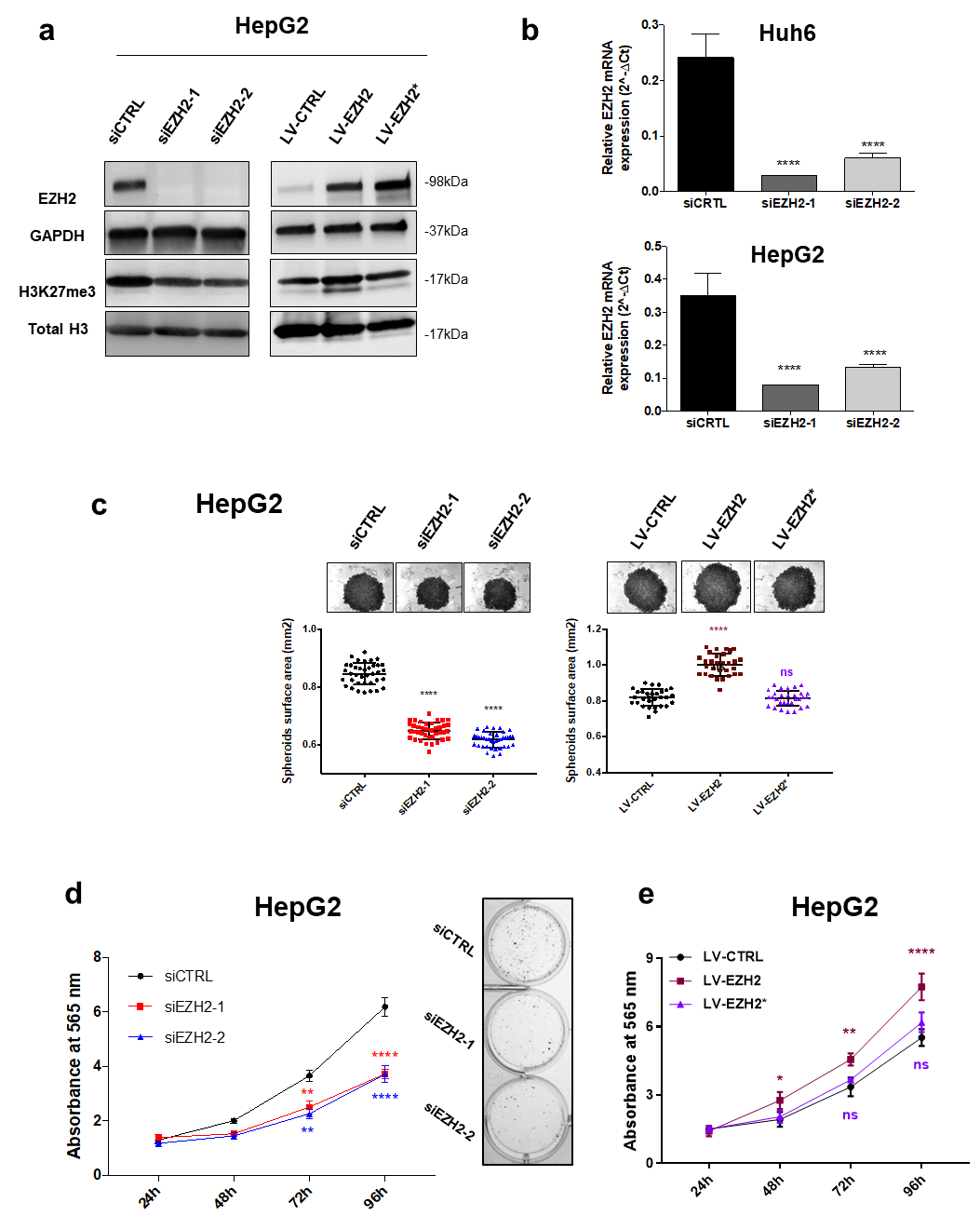


**Supplementary FIG. S6. Role of EZH2 and its methyl transferase activity in HB.** (**a**) Left blots: level of EZH2 and H3K27me3 proteins in control (siCTRL) and *EZH2*-depleted HepG2 cells using siEZH2-1 or siEZH2-2 as indicated. Right blots: level of EZH2 and H3K27me3 proteins in HepG2 cells transduced by LV-CTRL (empty cassette), LV-EZH2 (wild-type EZH2) or LV-EZH2* (H698A-mutant EZH2) lentiviruses (in this figure and the following). Representative blots of three independent experiments or more are shown in cropped images (loading control: GAPDH or total histone H3 protein as indicated). (**b**) Relative level of *EZH2* mRNA in control (siCTRL) and *EZH2*-depleted Huh6 (top) or HepG2 (bottom) cells using siEZH2-1 or siEZH2-2 as indicated. (**c**) Top panels: phase contrast micrographs of representative 96-h-old spheroids deriving from siCTRL and EZH2-depleted HepG2 cells (left) or from HepG2 cells transduced with LV-CTRL, LV-EZH2 or LV-EZH2* (right). Bottom panels: graphs presenting spheroid surface area in mm2 for each cellular model as described in top panels. (**b-c**) n=4, One way-ANOVA, ****p<0.0001; Sidak's multiple comparisons post-test. (**d-e**) Growth (Absorbance at 565 nm) of siCTRL *versus* *EZH2*-depleted HepG2 cells with a representative clonogenic test shown on the right (**d**) or of HepG2 cells ectopically expressing the CTRL cassette or EZH2 or EZH2* protein (**e**) (n=3, Two way-ANOVA, ****p<0.0001; Sidak's multiple comparisons post-test). ns, not significant; *p<0.05; **p<0.01; ****p<0.0001.


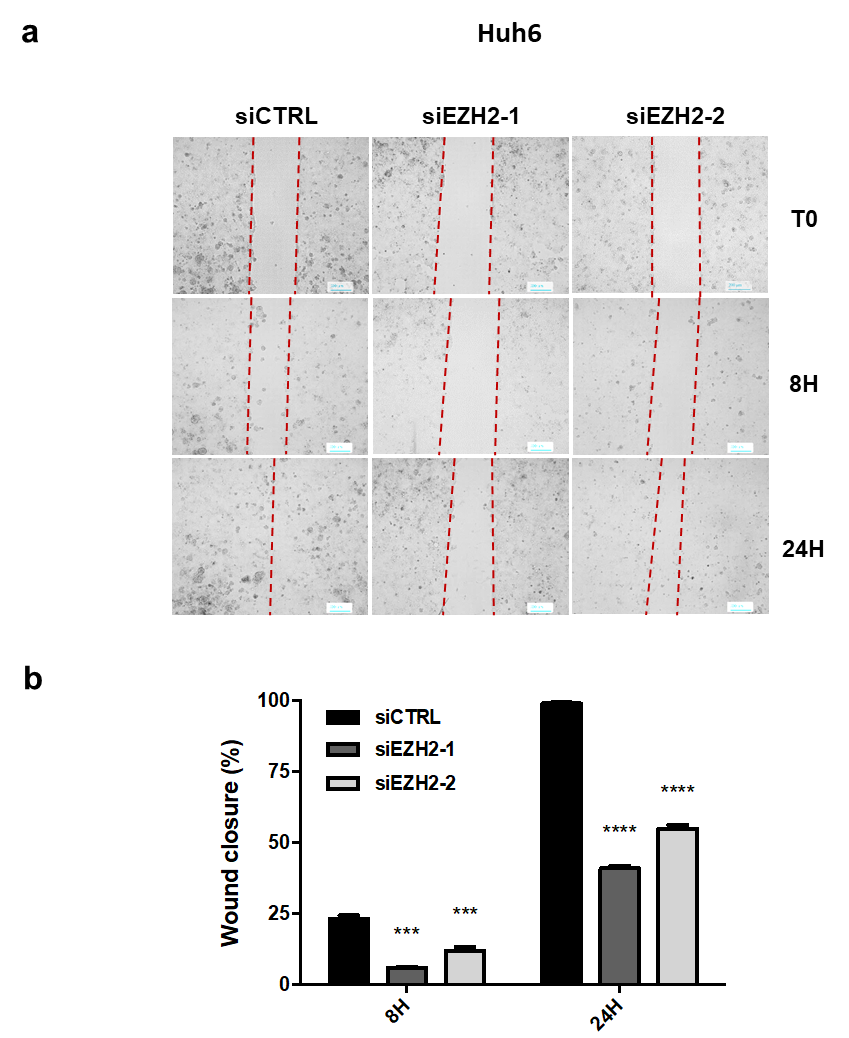


**Supplementary FIG. S7. *EZH2* silencing impedes Huh6 cell migration.** (**a-b**) Migration of siCTRL *versus* *EZH2*-depleted Huh6 cells. (**a**) Representative images of four independent experiments. (**b**) Bar graphs show means ± SD (n=4, Two-way-ANOVA, ****p<0.0001; Sidak's multiple comparisons post-test). ***p<0.001; ****p<0.0001.


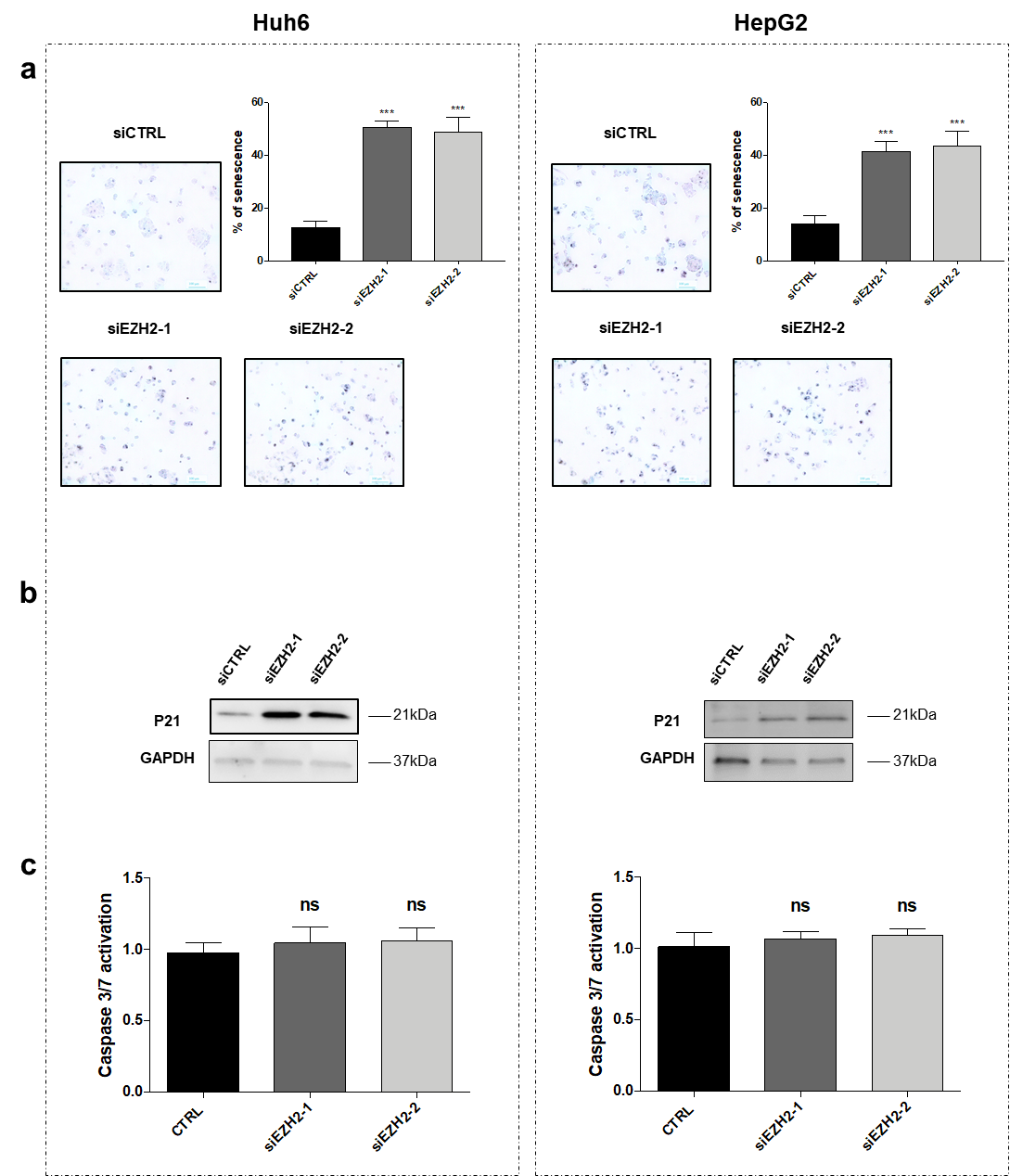


**Supplementary FIG. S8. *EZH2* silencing induces HB cell senescence with no sign of apoptotic cell death in monolayer culture conditions.** (**a**) Senescence measured in siCTRL *versus* *EZH2*-depleted Huh6 (left) or HepG2 (right) cells. Representative experiments are shown, and bar graphs recapitulate means ± SD (n=5, One way-ANOVA, ***p<0.001; Sidak's multiple comparisons post-test). (**b**) Relative level of cell cycle inhibitor protein P21 in siCTRL *versus* *EZH2*-depleted Huh6 (left) or HepG2 (right) cells. Representative blots of three independent experiments or more are shown in cropped images (loading control: GAPDH). (**c**) Graphs presenting caspase 3/7 activity in siCTRL *versus* *EZH2*-depleted Huh6 (left) or HepG2 (right) cells (n=3, One way-ANOVA, not significant; Sidak's multiple comparisons post-test). ns, not significant. ***p<0.001.


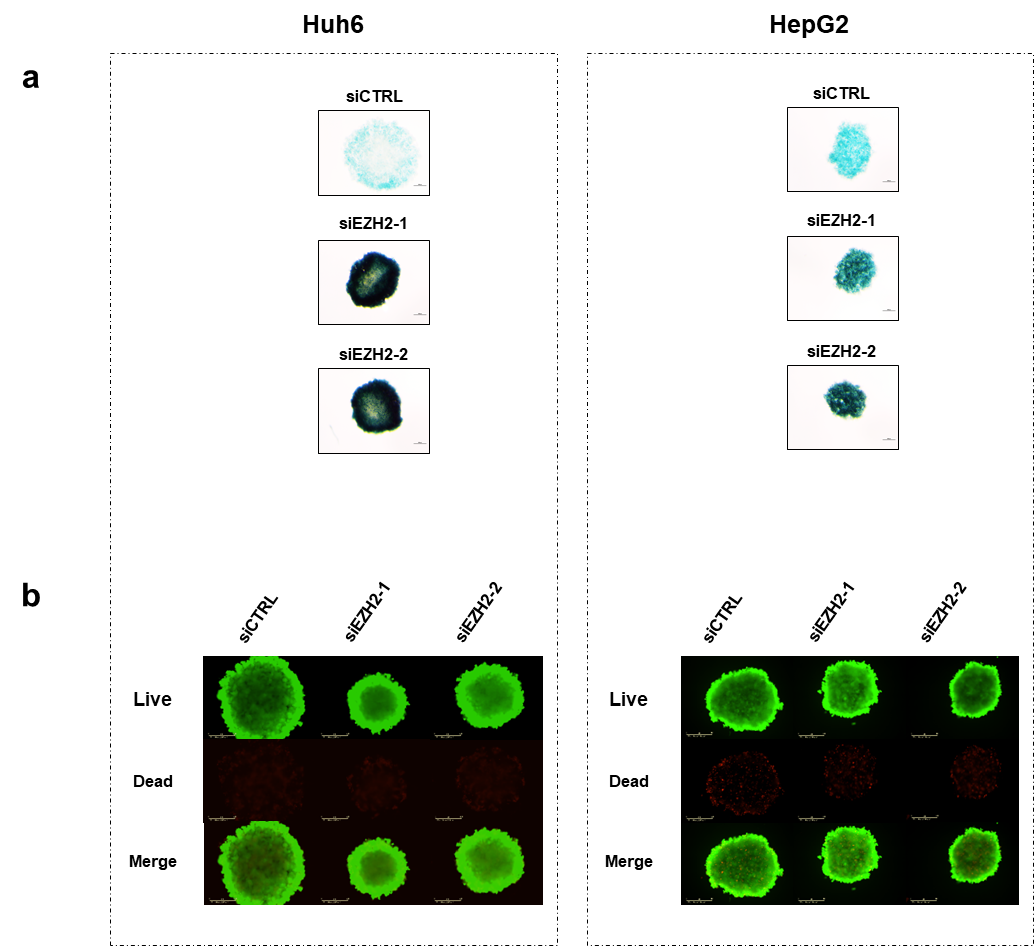


**Supplementary FIG. S9. *EZH2* silencing induces HB cell senescence with no sign of cell death in spheroids.** (**a**) Senescence measured in spheroids deriving from siCTRL and *EZH2*-depleted Huh6 (left) or HepG2 (right) cells using siEZH2-1 or siEZH2-2 as indicated. Representative phase contrast micrographs of four independent experiments are shown. Senescent cells are in dark blue. (**b**) siCTRL and *EZH2*-depleted Huh6 (left) or HepG2 (right) cells stained with calcein-AM and ethidium homodimer-1 reagents. In this figure and the following, live cells appear in green and dead cells in red. Representative images of four independent experiments.


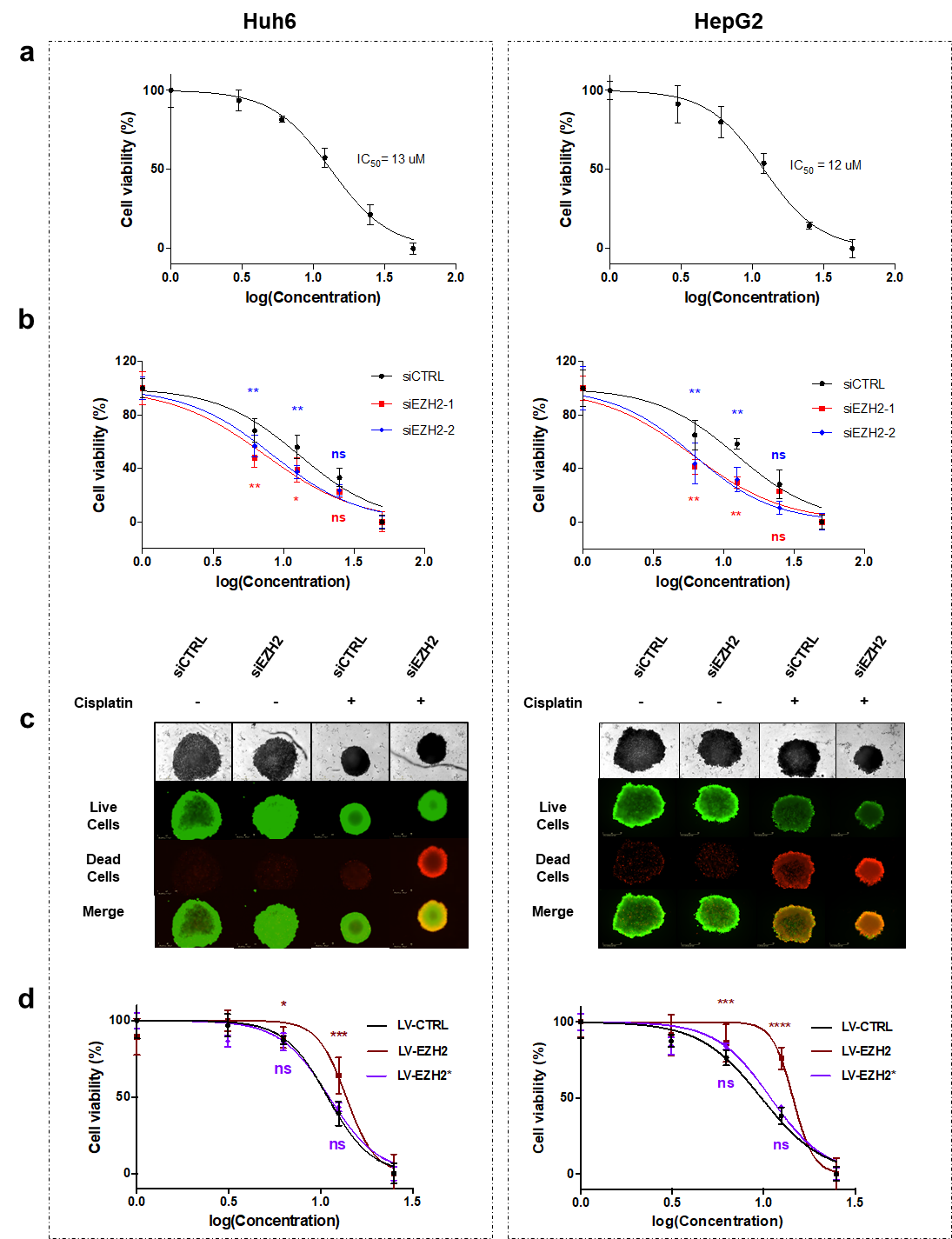


**Supplementary FIG. S10. Cisplatin resistance in hepatoblastoma cells is partly mediated by EZH2 methyl transferase activity.** (**a**) Graphs show the percentage of viable Huh6 (left) or HepG2 (right) cells treated with increasing concentrations of cisplatin (n=3; bars = means +/- SD). For each cell line, the IC_50_ is shown in the corresponding graph. (**b-c**) Response of siCTRL *versus* *EZH2*-depleted Huh6 (left) and HepG2 (right) cells to cisplatin (two different siRNAs used as indicated). (**b**) Graphs show the percentage of viable cells cultured as monolayer and treated by increasing concentrations of cisplatin (n=4; bars = means +/- SD; Two way-ANOVA, **p<0.01; Sidak's multiple comparisons post-test). (**c**) Representative images showing live and dead Huh6 (left) and HepG2 (right) cells cultured as spheroids and treated or not for 48 hr with cisplatin at IC_50_. (**d**) Response of Huh6 (left) and HepG2 (right) cells expressing the LV-CTRL (empty cassette), LV-EZH2 (wild-type EZH2) or LV-EZH2* (H698A-mutant EZH2) cassette to cisplatin. Graphs show the percentage of viable Huh6 (left) and HepG2 (right) cells (n=3; bars = means +/- SD; Two way-ANOVA, ****p<0.0001; Sidak's multiple comparisons post-test). ns, not significant; *p<0.05; **p<0.01; ***p<0.001; ****p<0.0001.


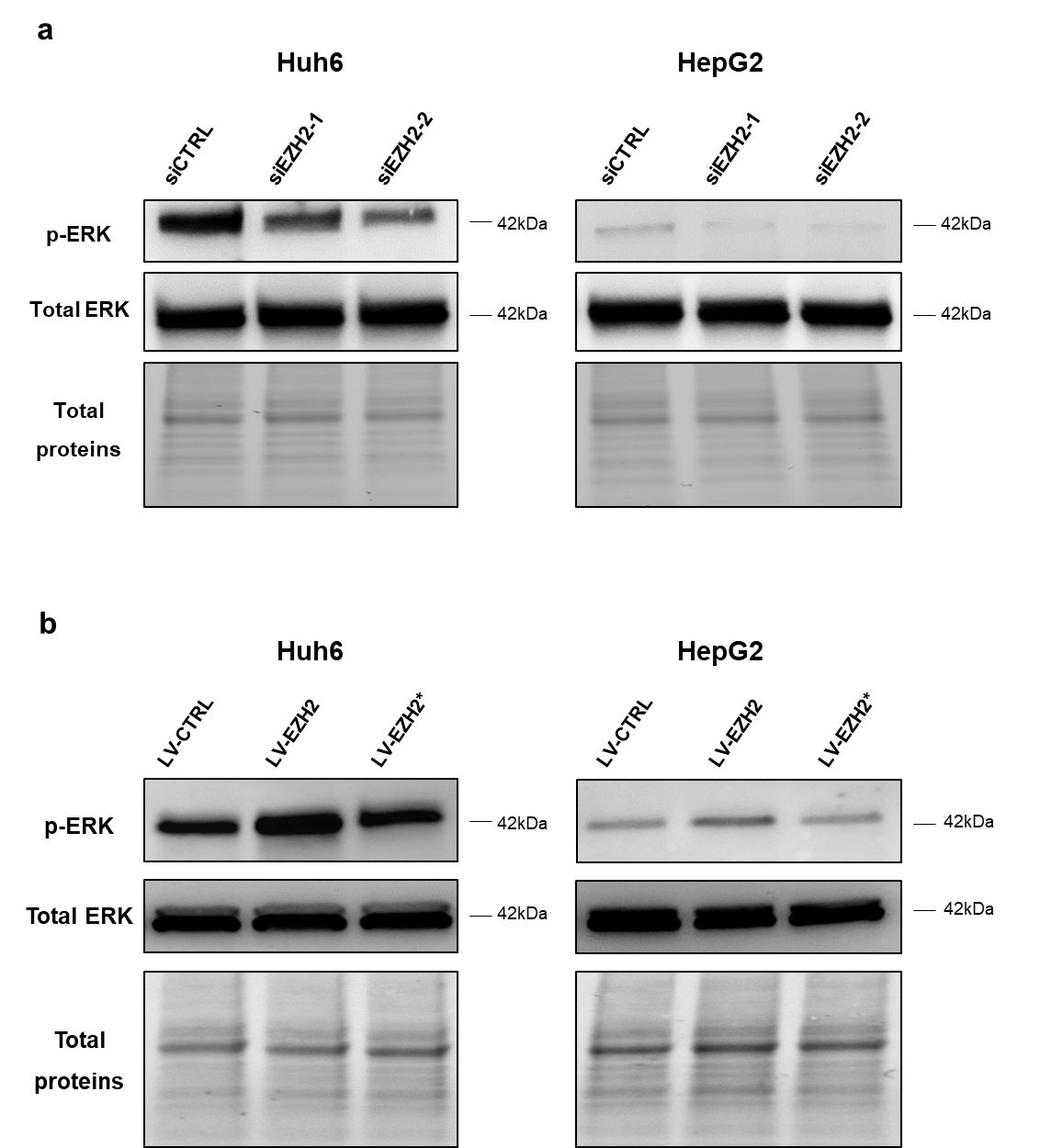


**Supplementary FIG. S11. EZH2 methyl transferase activity supports ERK phosphorylation and MAPK pathway signaling in hepatoblastoma.** (**a**) Levels of total ERK and phospho-ERK (p-ERK) proteins in siCTRL *versus* *EZH2*-depleted Huh6 (left) or HepG2 (right) cells. (**b**) Levels of total ERK and phospho-ERK (p-ERK) proteins in Huh6 (left) or HepG2 (right) cells expressing CTRL, EZH2 or LV-EZH2* cassette. Representative blots of three experiments are shown in cropped images. (**a-b**) Loading control: total proteins.


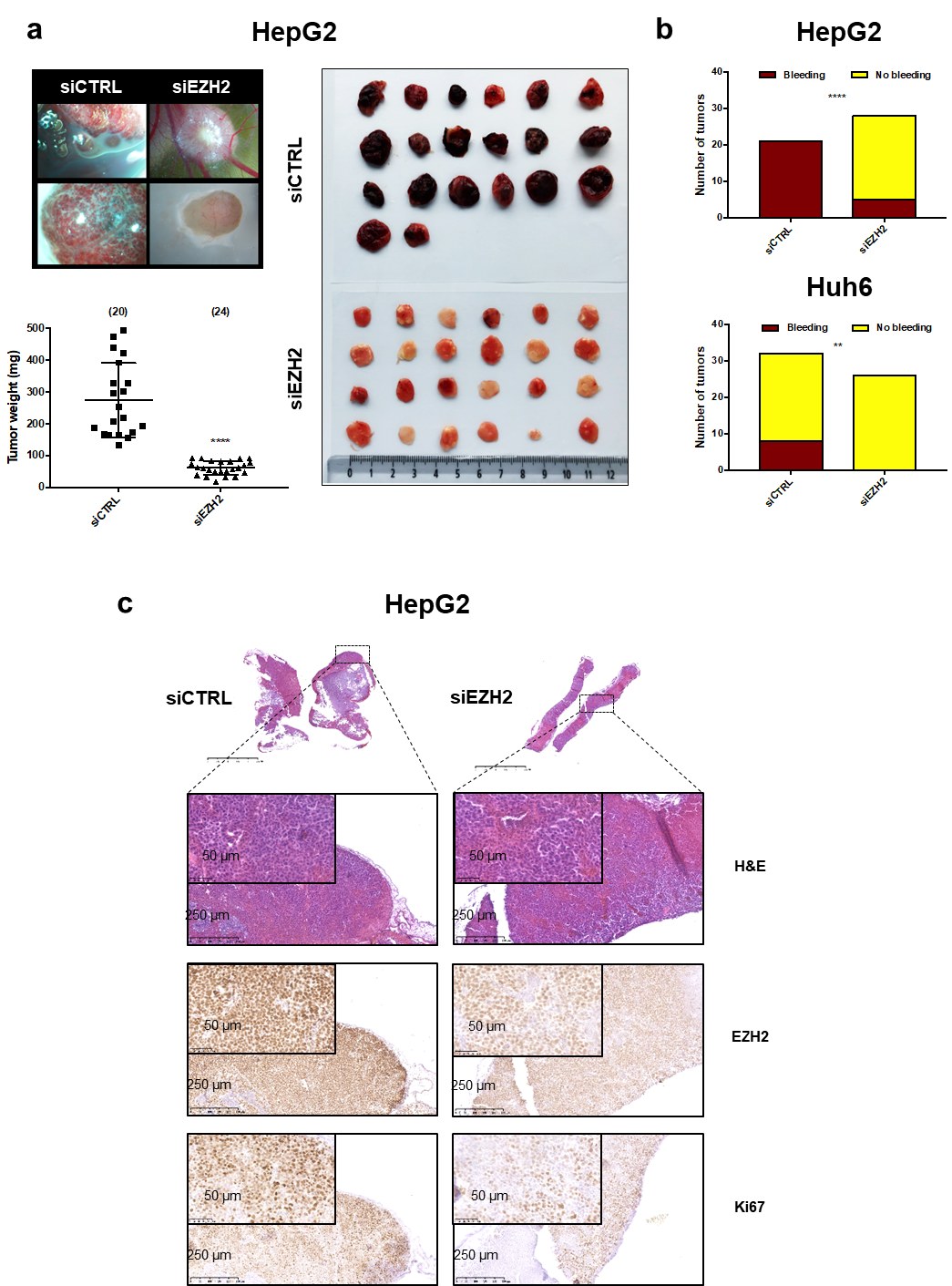


**Supplementary FIG. S12. *EZH2* silencing inhibits the growth and vascularization of HepG2-derived CAM tumors *in vivo***. (**a-c**) CAM assay after implantation of siCTRL and *EZH2*-depleted (using siEZH2-1) HepG2 cells on the chick chorioallantoic membrane. (**a**) Top left panel: representative stereomicroscopic images of 7-day-old tumors (top) and representative images of extracted and fixed tumors (bottom). Bottom left panel: weight of 7-day-old tumors. The number of eggs analyzed per group is shown in brackets above the corresponding group of values. Horizontal line and whiskers represent mean ± SD (Wilcoxon matched-pairs signed rank test). Right panel: Image of 7-day extracted tumors [siCTRL (top) and siEZH2 (bottom)]. (**b**) Bleeding in tumors deriving from grafted Huh6 (top) or HepG2 (bottom) cells (two-tailed Chi-square test). (**c**) Immunoshistochemical stains of HepG2-derived CAM tumors. Representative siCTRL (left) and *EZH2*-depleted (right) tumors were stained using hematoxylin-eosin (H&S) or antibodies against EZH2 and Ki67. Scale bars are as indicated. **p<0.01; ***p<0.001, ****p<0.0001.


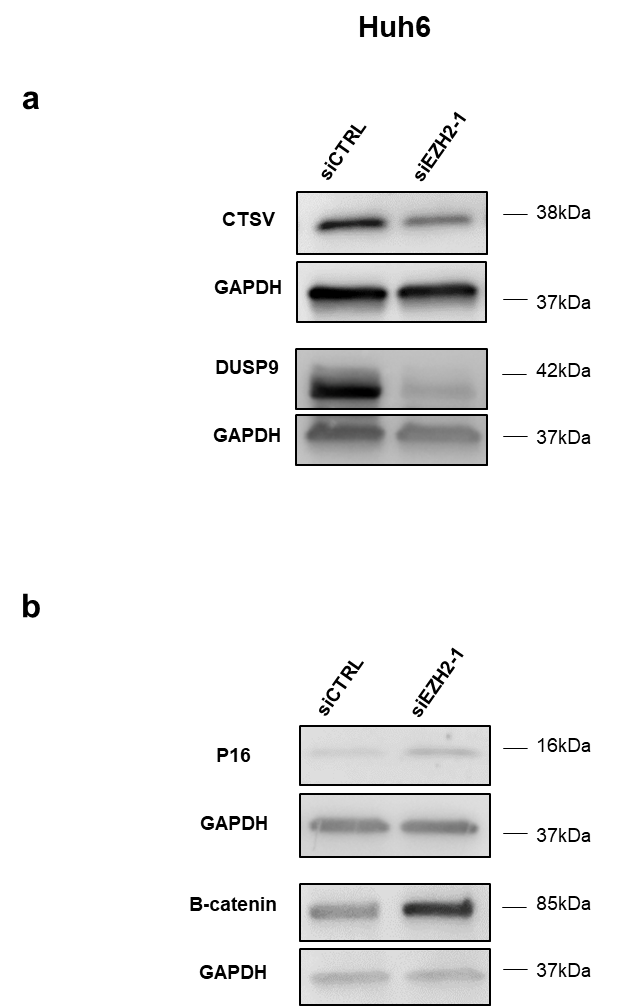


**Supplementary FIG. S13. Proteins deregulated in *EZH2*-silenced Huh6 cells and measured by proteomics.** (**a-b**) Relative level of CTSV and DUSP9 proteins (downregulated, **a**) or of P16 and β-catenin proteins (upregulated, **b**) in siCTRL *versus* *EZH2*-depleted Huh6 cells. Representative blots of three independent experiments are shown in cropped images (loading control: GAPDH).


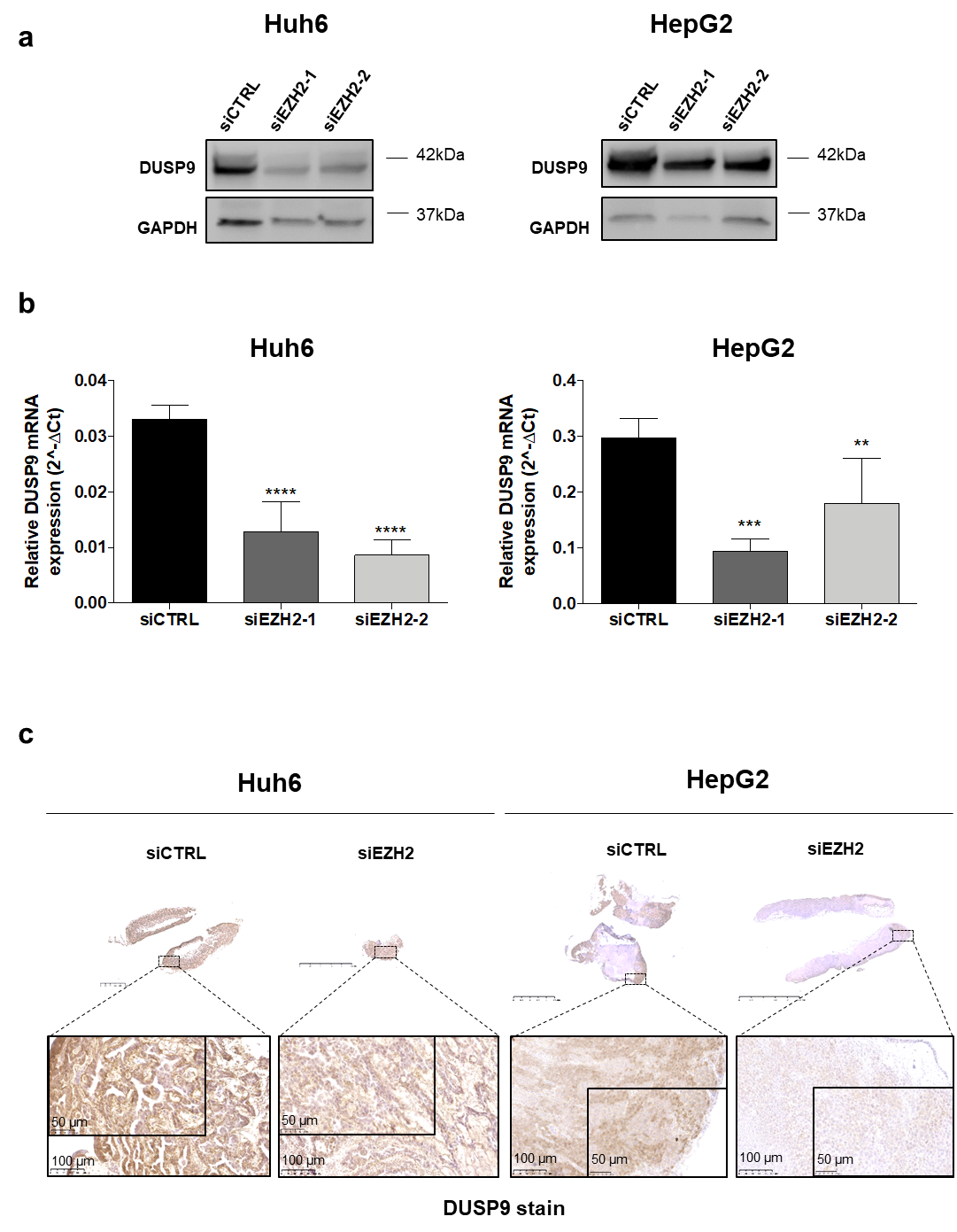


**Supplementary FIG. S14. Down-regulation of *DUSP9* mRNA and protein in *EZH2*-silenced HB cells.** (**a-b**) Level of DUSP9 protein (**a**) and mRNA (**b**) in siCTRL *versus* *DUSP9*-depleted Huh6 (left) or HepG2 (right) cells using siDUSP9-1 or siDUSP9-2 as indicated. (**b**) Bar graphs show means ± SD (n=3, One way-ANOVA, p<0,0001; Sidak's multiple comparisons post-test). (**c**) Immunoshistochemical stains of Huh6-derived (left) or HepG2-derived (right) CAM tumors. Representative siCTRL (left side) and *EZH2*-depleted (right side) tumors were stained as indicated using an antibody against DUSP9. Scale bars are as indicated. **p<0.01; ***p<0.001; ****p<0.0001.


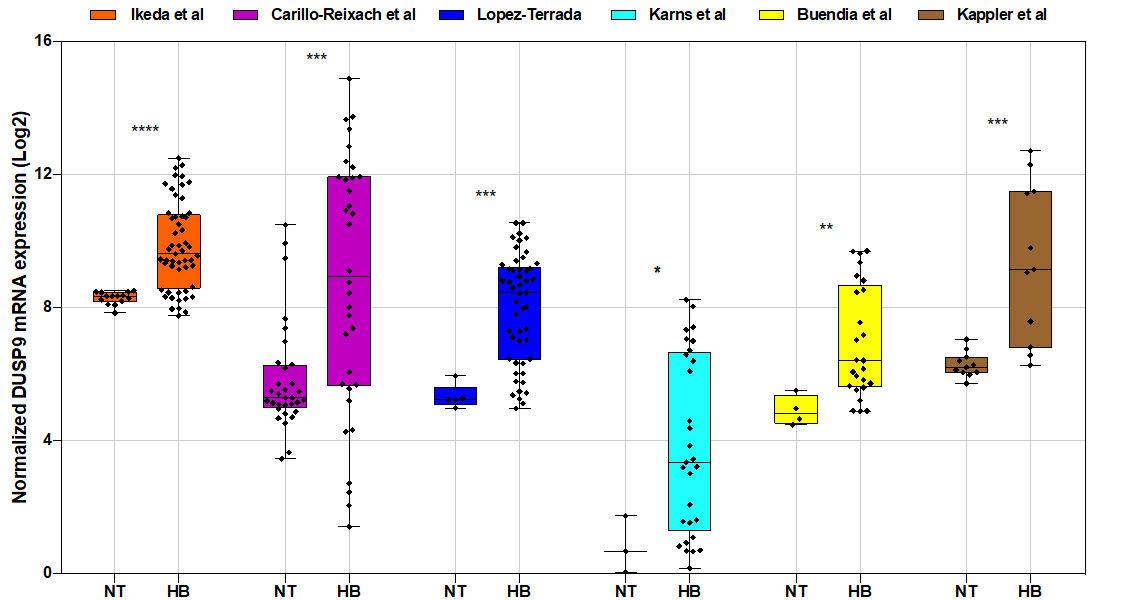


**Supplementary FIG. S15.** **Expression of *DUSP9* mRNA in hepatoblastoma.** (**a**) Expression of *DUSP9* transcript in HB and NT samples from Ikeda’s dataset (gse131329, [1]), Carrillo-Reixach’s dataset (gse133039, [2]), Lopez-Terrada’s dataset (gse75271, [3]), Karns’s dataset (gse81928, [4]), Buendia’s dataset [5] and Kappler’s dataset (gse151347, [6]). Unpaired Mann & Whitney test. *p<0.05; **p<0.01; ***p<0.001; ****p<0.0001.


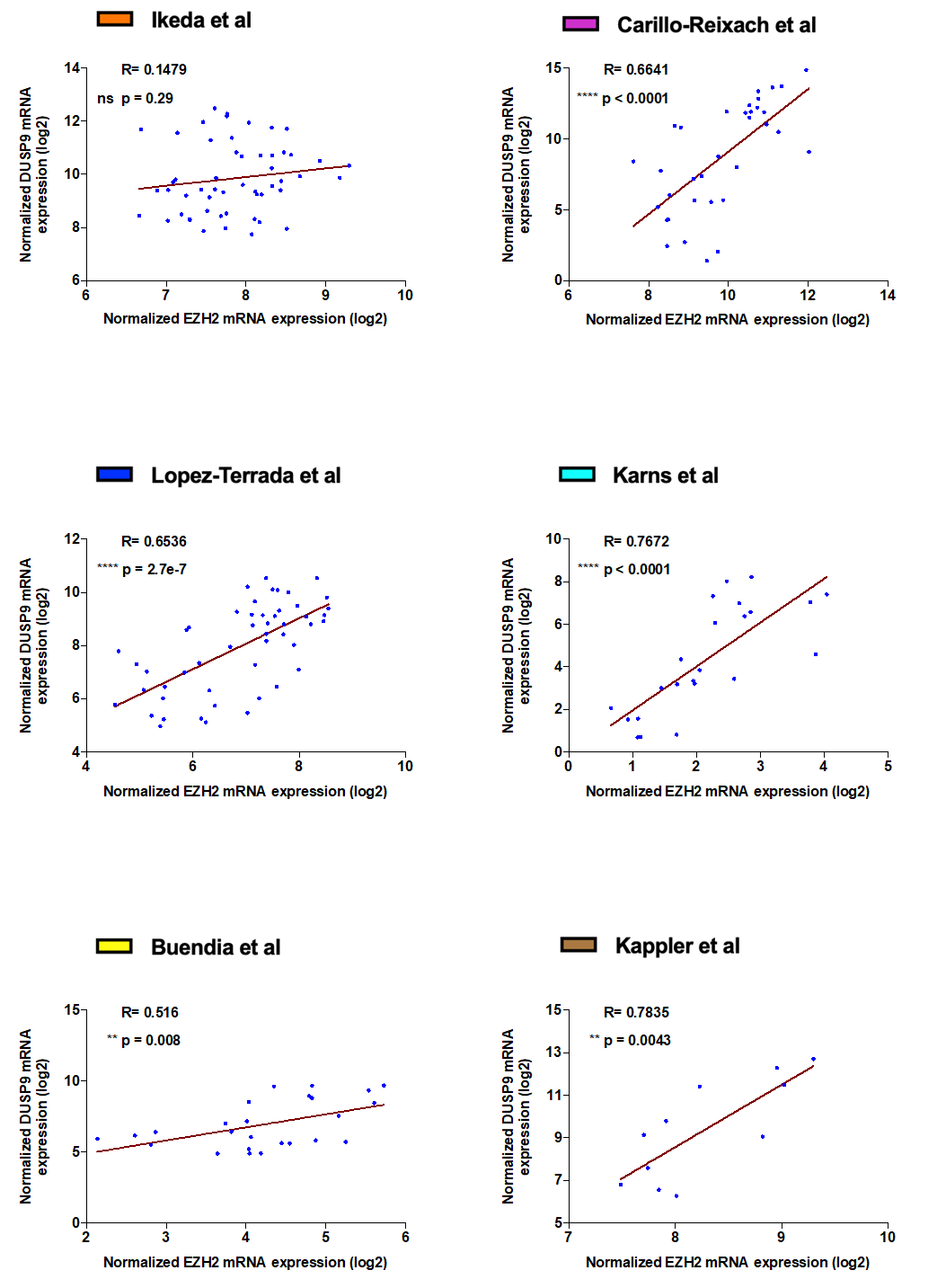


**Supplementary FIG. S16. Correlative analysis between *EZH2* and *DUSP9* transcripts in hepatoblastoma.** Graphs show the two-tailed Pearson R correlations between *EZH2* and *DUSP9* transcripts in HB samples from Ikeda’s dataset (gse131329, [1]), Carrillo-Reixach’s dataset (gse133039, [2]), Lopez-Terrada’s dataset (gse75271, [3]), Karns’s dataset (gse81928, [4]), Buendia’s dataset [5] and Kappler’s dataset (gse151347, [6]). For each dataset, the R and p-values are as shown in the corresponding graph.


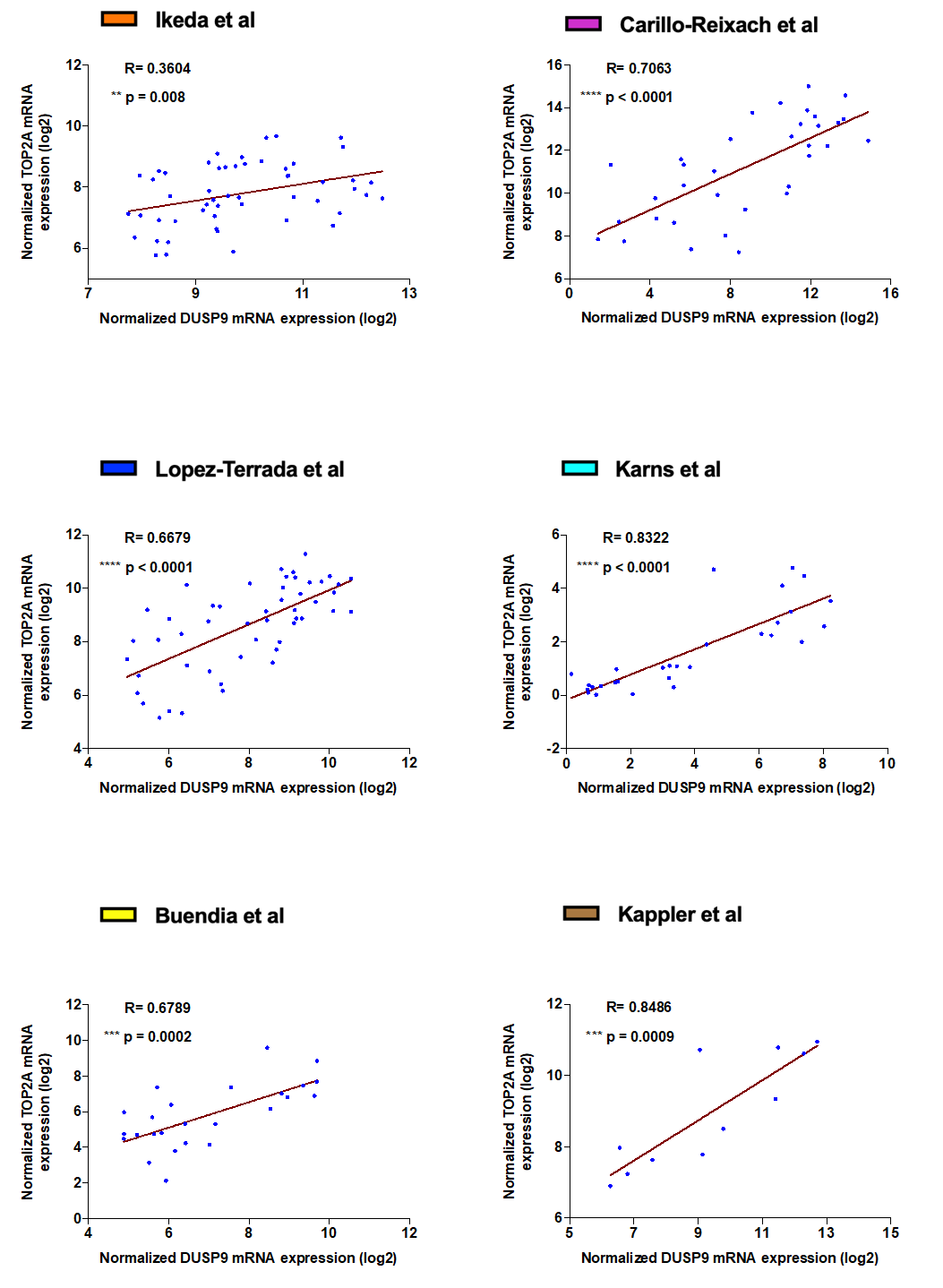


**Supplementary FIG. S17. Correlative analysis between *DUSP9* and *TOP2A* transcripts in hepatoblastoma.** Graphs show the two-tailed Pearson R correlations between *DUSP9* and *TOP2A* transcripts in HB samples from Ikeda’s dataset (gse131329, [1]), Carrillo-Reixach’s dataset (gse133039, [2]), Lopez-Terrada’s dataset (gse75271, [3]), Karns’s dataset (gse81928, [4]), Buendia’s dataset [5] and Kappler’s dataset (gse151347, [6]). For each dataset, the R and p-values are as shown in the corresponding graph.


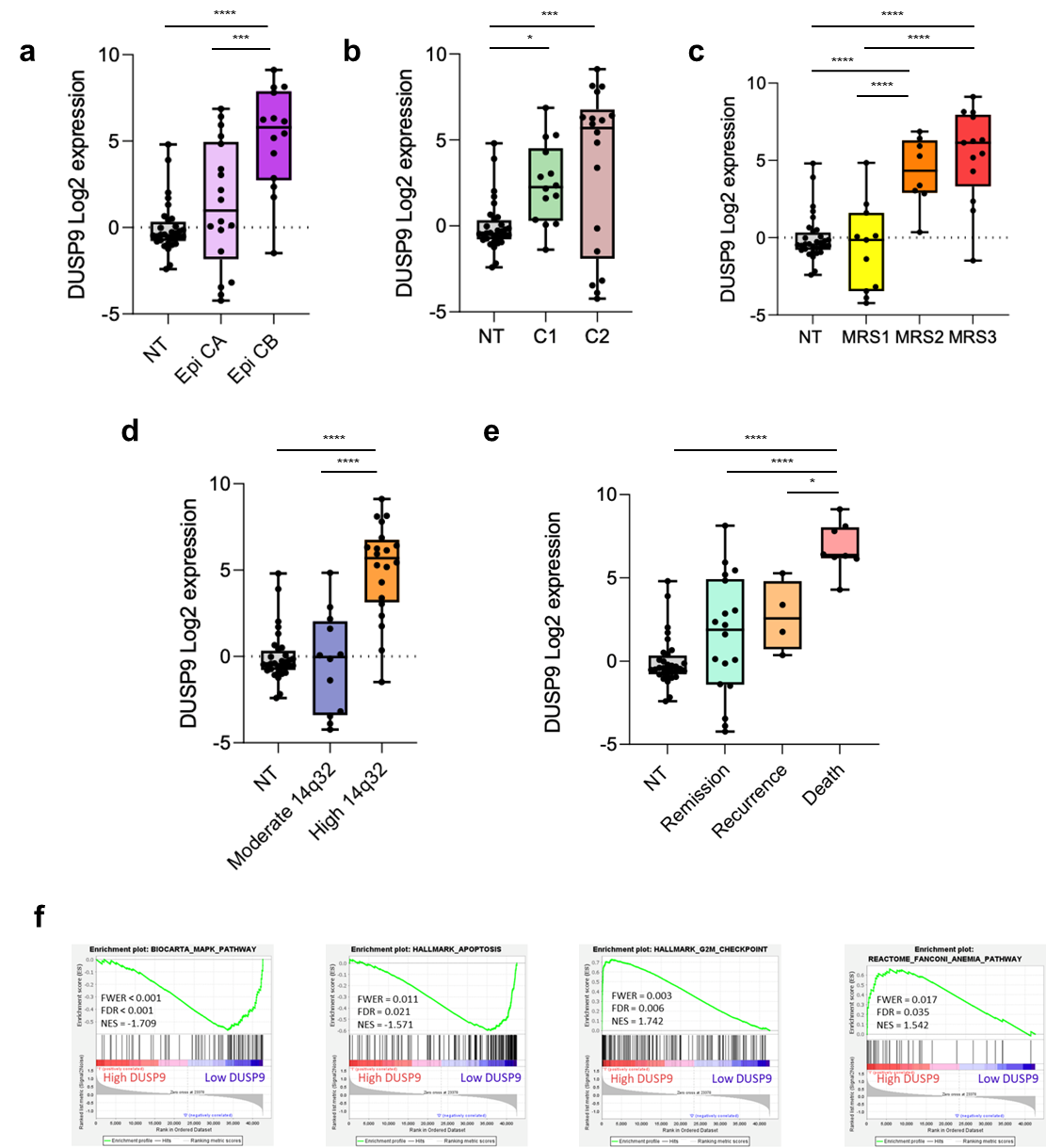


**Supplementary FIG. S18. Links between *DUSP9* transcript expression and clinical data in hepatoblastoma.** (**a-e**) Expression levels of *DUSP9* mRNA in NT (n=32), Epi CA (n = 18) and Epi CB (n=14) (**a**), in NT (n=32), C1 (n=14) and C2 tumors (n=18) (**b**, Buendia’s dataset, [5]), in NT (n=32), MRS-1 (n=11), MRS-2 (n=8) and MRS-3 (n=13) (**c**, gse133039, [2]), in NT (n=32), moderate (n=12) and high 14q32 expression tumors (n=20) (**d**) or in NT (n=32), patients in remission (n=18), in recurrence (n=4) and those deceased (n=8) (**e**, gse133039, [2]). One-way ANOVA test, ****p<0.0001; Tukey post-test. (**f**) GSEA of *DUSP9* mRNA low expression and high expression samples categorized according to tumor median. FDR q value<0.25 indicate statistically significant trend. NES, Normalized Enrichment Score. Left in red, high expression of *DUSP9* mRNA; Right in blue, low expression of *DUSP9* mRNA. *p<0.05; ***p<0.001; ****p<0.0001.

**
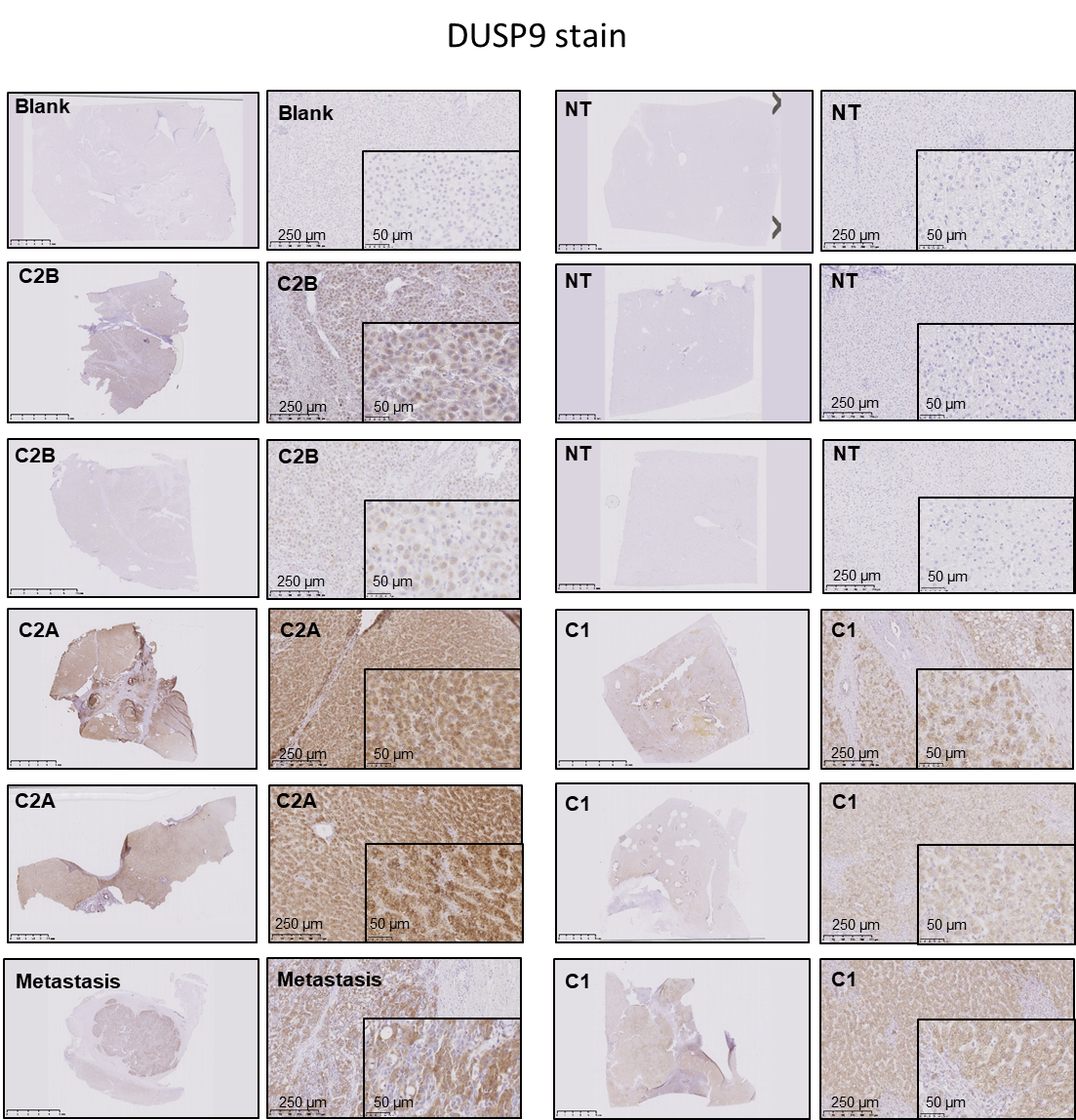
**

**Supplementary FIG. S19. Expression of DUSP9 protein in hepatoblastoma patient tissues.** Immunoshistochemical stains of HB patient tissues classed as NT, C1, C2A, C2B and a lung metastasis. Eleven representative samples, one from a metastasis, two from C2A and C2B, and three from NT and C1, were stained using an antibody against DUSP9. Blank: control stain with no primary antibody. Scale bars are as indicated.


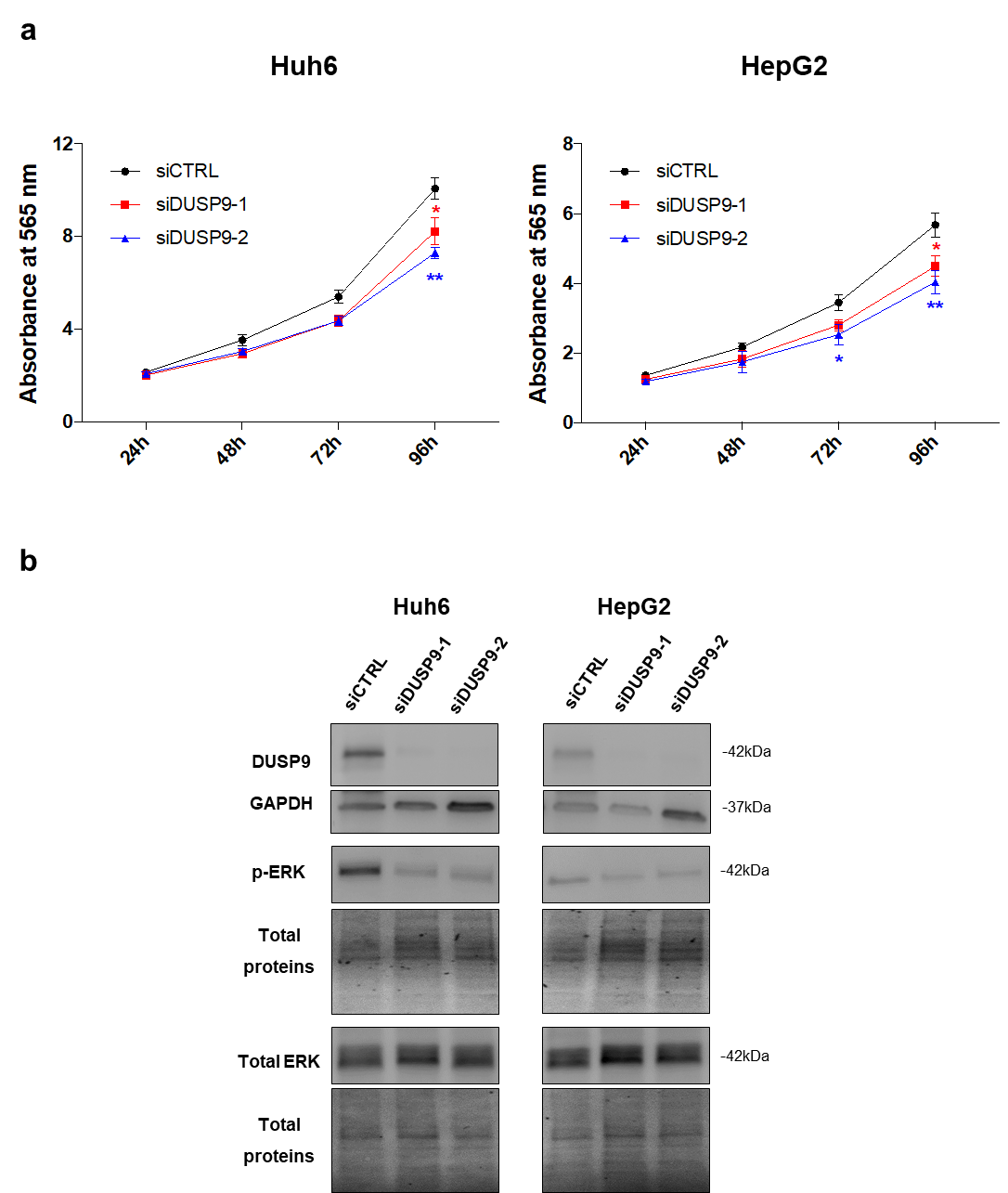


**Supplementary FIG. S20. Impact of *DUSP9* depletion in hepatoblastoma cells.** (**a**) Growth (Absorbance at 565 nm) of siCTRL *versus* *DUSP9*-depleted Huh6 (left) or HepG2 (right) cells (n=3, Two way-ANOVA, **p<0.01; Sidak's multiple comparisons post-test). (**b**) Levels of DUSP9, phospho-ERK (p-ERK) and total ERK proteins in siCTRL *versus* *DUSP9*-depleted Huh6 (left) or HepG2 (right) cells. Representative blots of three experiments or more are shown in cropped images (loading control: GAPDH or total proteins as shown). *p<0.05; **p<0.01.


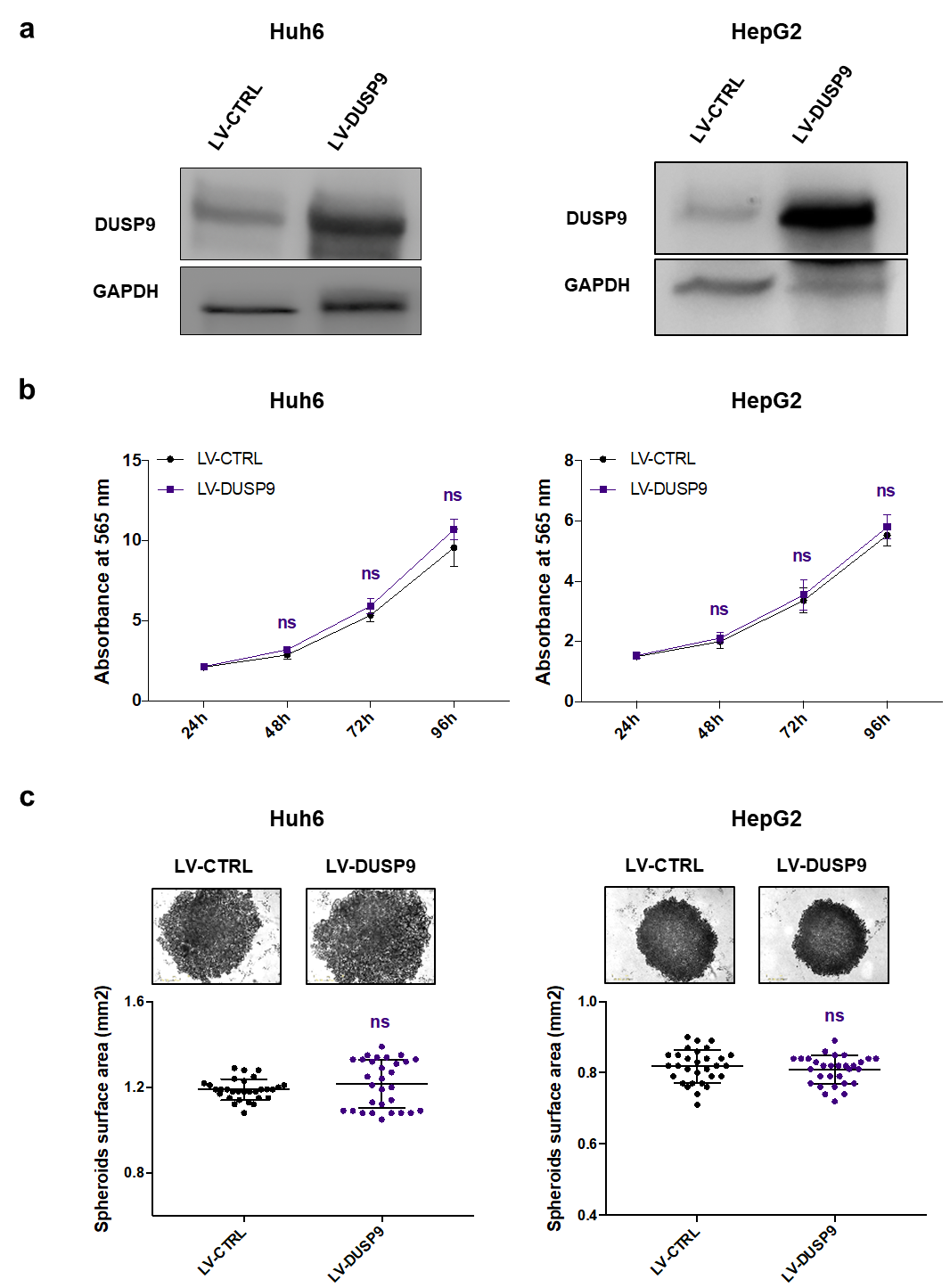


**Supplementary FIG. S21. Forced expression of DUSP9 does not alter hepatoblastoma cell growth capacity.** (**a**) Relative expression of DUSP9 protein in Huh6 (left) and HepG2 (right) cells ectopically expressing DUSP9 cassette (LV-DUSP9) or an empty cassette (LV-CTRL). (**b**) Growth (Absorbance at 565 nm) of Huh6 (left) and HepG2 (right) cells ectopically expressing DUSP9 or the CTRL cassette (n=3, Two way-ANOVA, ns; Sidak's multiple comparisons post-test). (**c**) Top panels: Representative phase contrast micrographs of 96-h-old spheroids deriving from Huh6 (left) and HepG2 (right) cells ectopically expressing DUSP9 (LV-DUSP9) or an empty cassette (LV-CTRL). Bottom panel: Graphs presenting spheroid surface area in mm2 in the conditions described above (n=4, Unpaired Mann Whitney test). (**b-c**) ns, not significant.


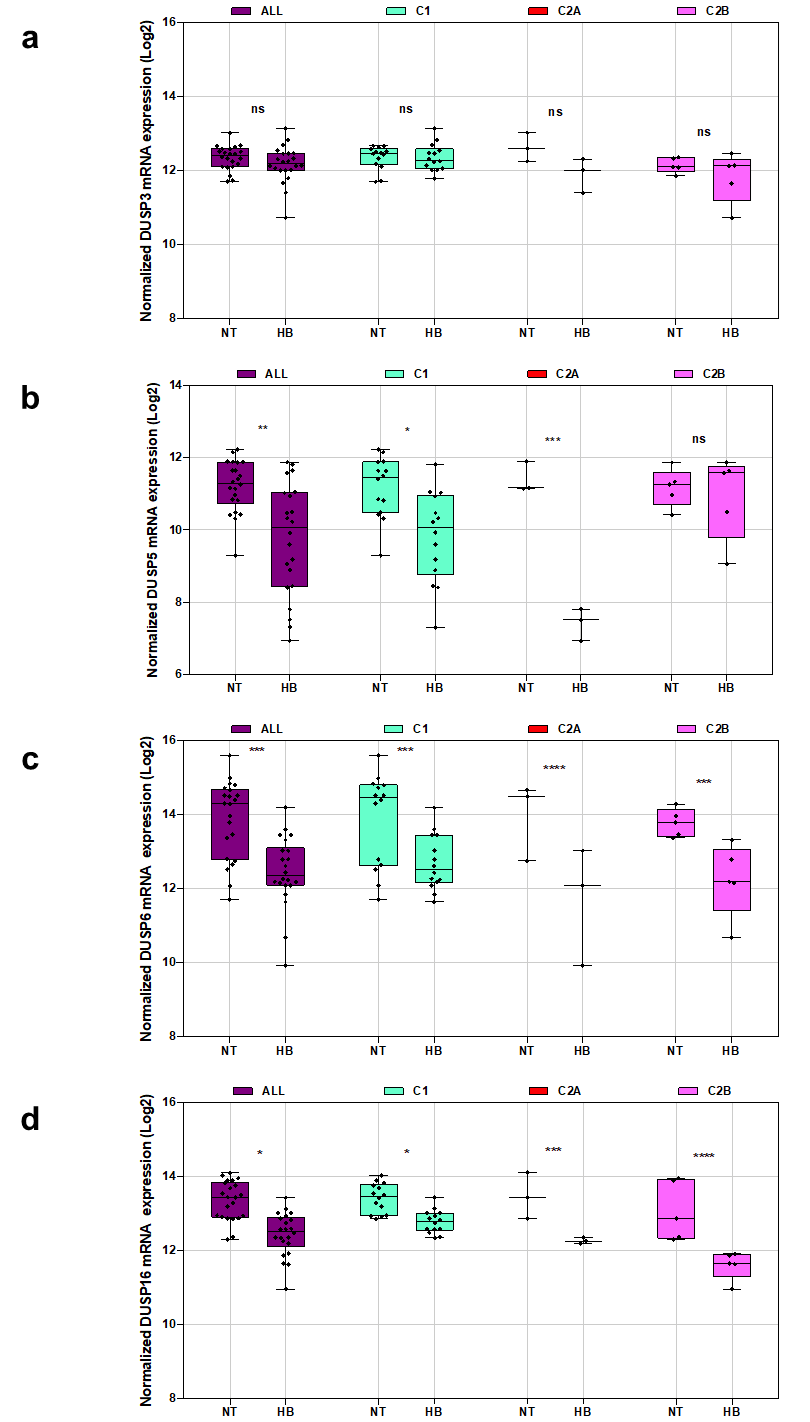


**Supplementary FIG. S22. Expression of *DUSP* family members in hepatoblastoma.** Expression of *DUSP3*, *DUSP5*, *DUSP6* and *DUSP16* transcripts (top to bottom order) in all, C1, C2A and C2B tumors and NT samples from Raymond’s dataset (gse104766, [7]) (Wilcoxon matched pairs signed rank test). ns, not significant; *p<0.05; **p<0.01; ***p<0.001; ****p<0.0001.

**
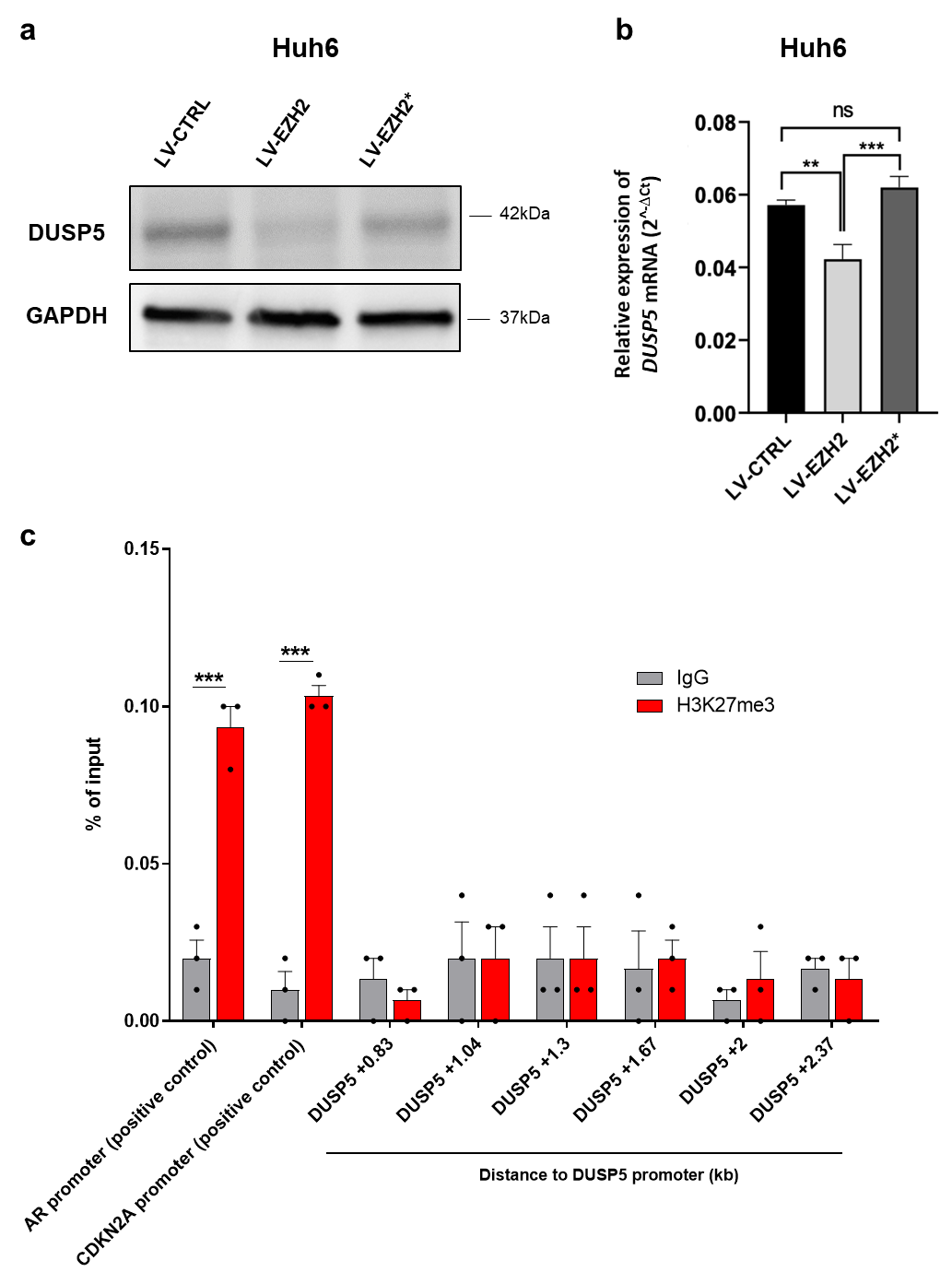
**

**Supplementary FIG. S23. EZH2 represses *DUSP5* expression in HB cells through its methyl transferase activity.** (**a**) Level of DUSP5 protein in Huh6 cells expressing the CTRL, EZH2 or EZH2* cassette. Representative blots of three experiments are shown in cropped images (loading control: GAPDH). (**b**) Relative expression of *DUSP5* mRNA in Huh6 cells expressing the CTRL, EZH2 or EZH2* cassette. Bar graphs show means ± SD (n=3, One way-ANOVA, p<0,0001; Sidak's multiple comparisons post-test). (**c**) *DUSP5* promoter is not associated with HEK27me3 marks. ChIP was performed in Huh6 cells using anti-H3K27me3 and control IgG antibodies and then subjected to qPCR using primer pairs targeting windows within +0.83 kb to +2.3 kb of the *DUSP5* transcriptional starting site gene. Androgen receptor and *CDKN2A* promoters were used as positive controls [16, 17]. Data shown are mean (± SEM) of % of input of 3 biological replicates. Statistical significance was evaluated using multiple *t*-test (n=3); ns, not significant; **p<0.01; ***p<0.001.


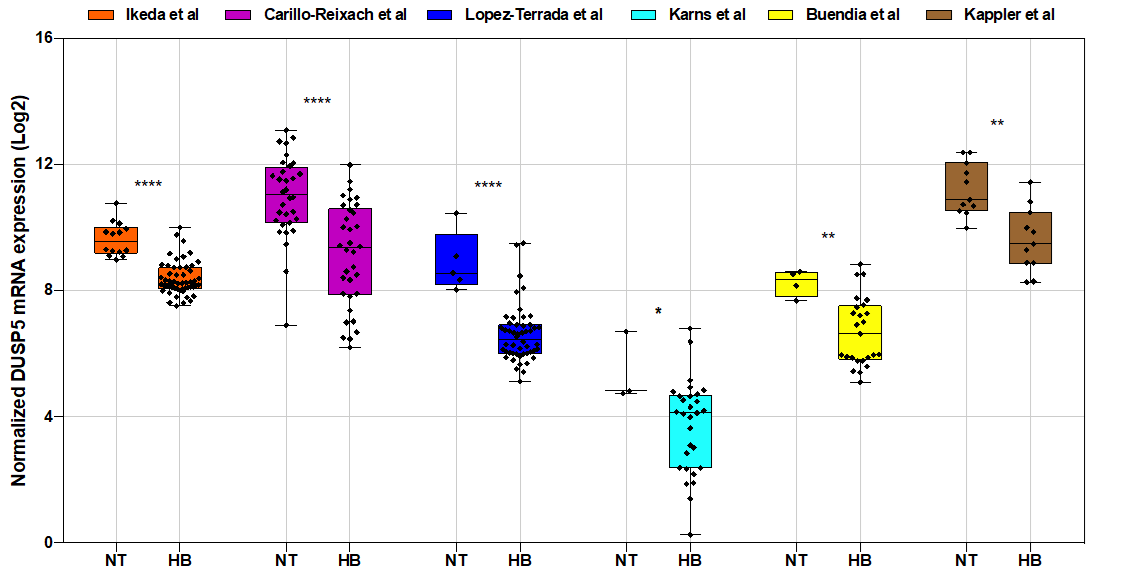


**Supplementary FIG. S24.** **Expression of *DUSP5* transcript in hepatoblastoma.** (**a**) Expression of *DUSP5* transcript in HB and NT samples from Ikeda’s dataset (gse131329, [1]), Carrillo-Reixach’s dataset (gse133039, [2]), Lopez-Terrada’s dataset (gse75271, [3]), Karns’s dataset (gse81928, [4]), Buendia’s dataset [5] and Kappler’s dataset (gse151347, [6]). Unpaired Mann & Whitney test. *p<0.05; **p<0.01; ****p<0.0001.


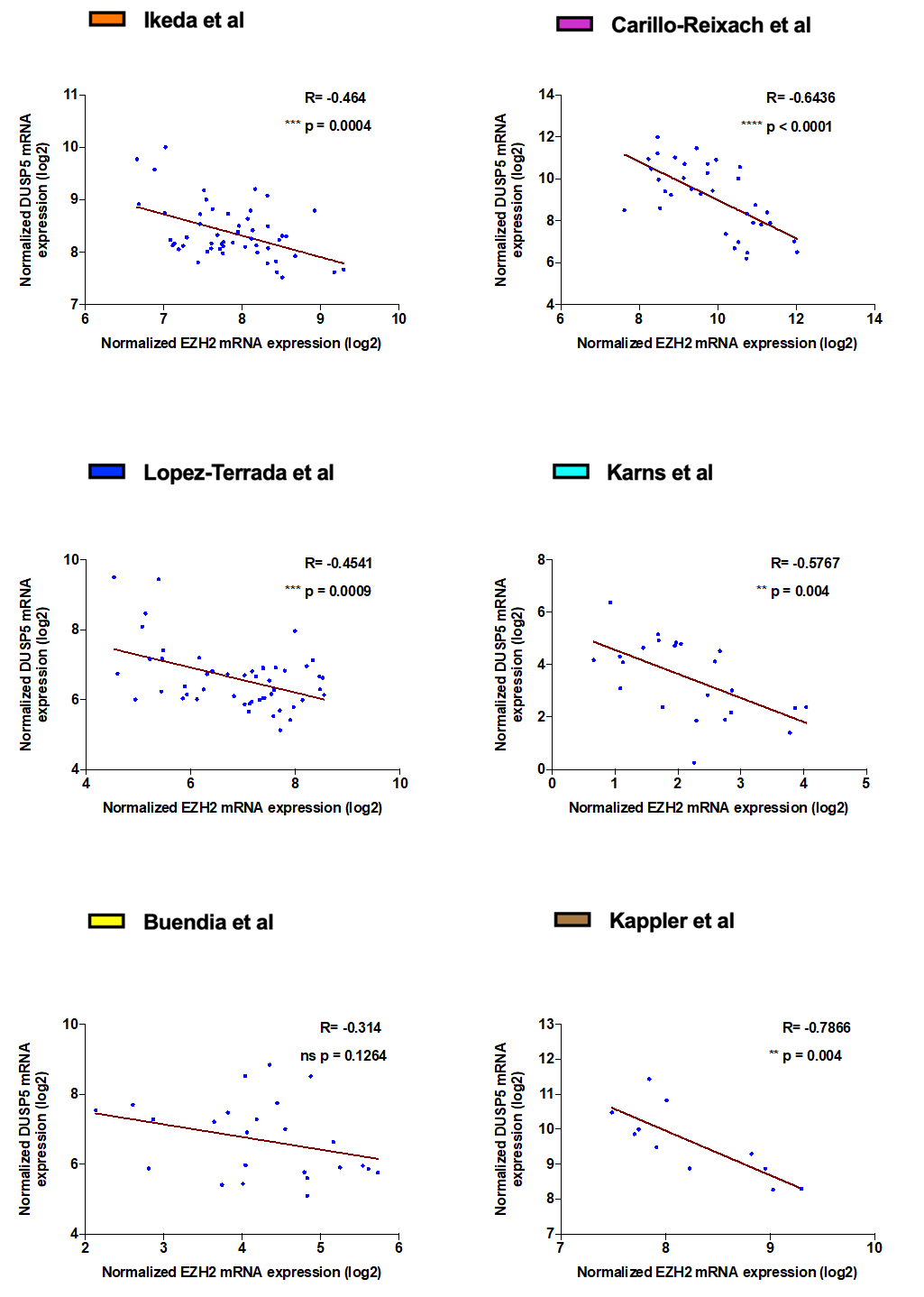


**Supplementary FIG. S25. Correlative analysis between *DUSP5* and *EZH2* transcripts in hepatoblastoma.** Graphs show the two-tailed Pearson R correlations between *DUSP5* and *EZH2* transcripts in HB samples from Ikeda’s dataset (gse131329, [1]), Carrillo-Reixach’s dataset (gse133039, [2]), Lopez-Terrada’s dataset (gse75271, [3]), Karns’s dataset (gse81928, [4]), Buendia’s dataset [5] and Kappler’s dataset (gse151347, [6]). For each dataset, the R and p-values are as shown in the corresponding graph.


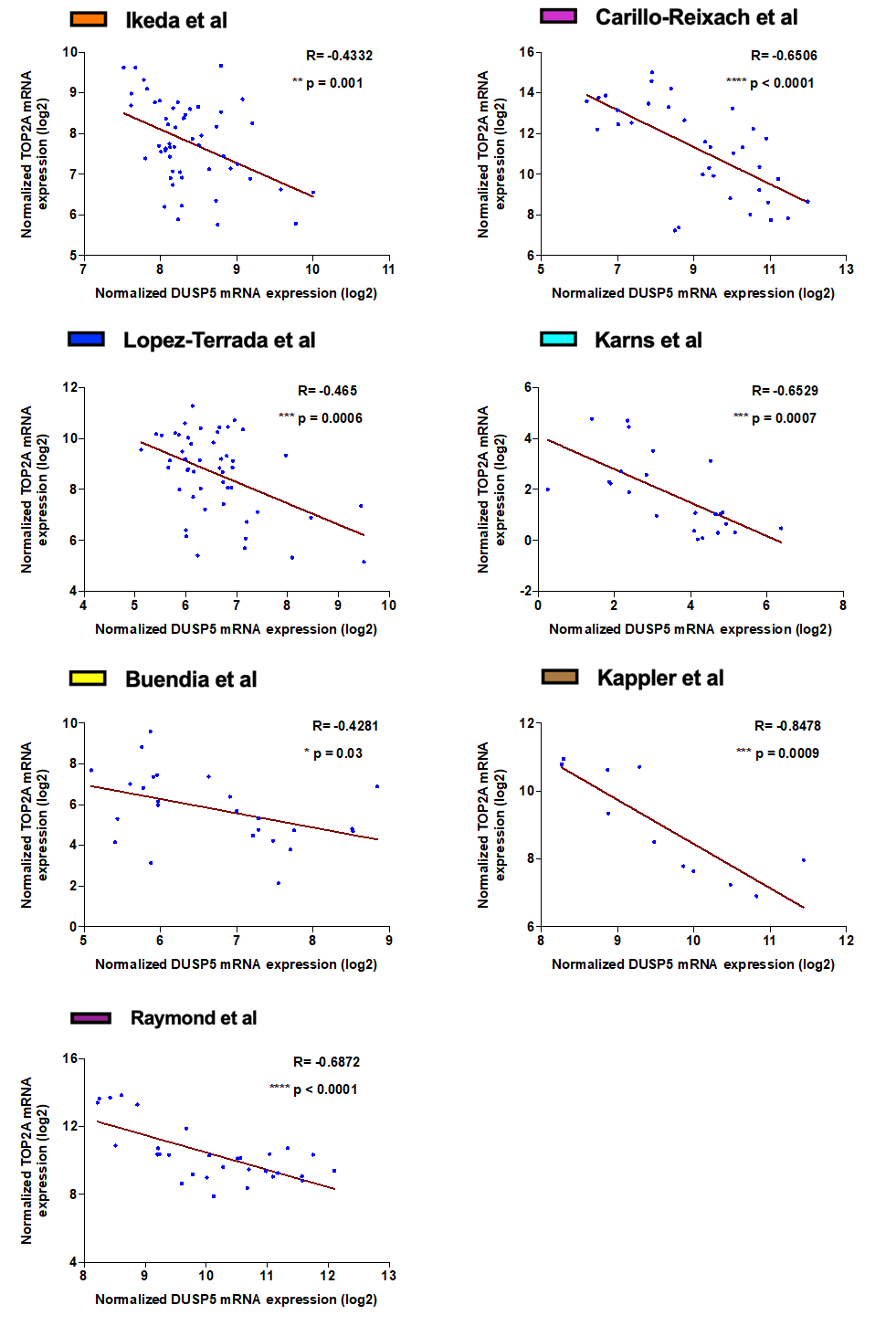


**Supplementary FIG. S26. Correlative analysis between *DUSP5* and *TOP2A* in hepatoblastoma.** Graphs show the two-tailed Pearson R correlations between *DUSP5* and *TOP2A* transcripts in HB samples from Ikeda’s dataset (gse131329, [1]), Carrillo-Reixach’s dataset (gse133039, [2]), Lopez-Terrada’s dataset (gse75271, [3]), Karns’s dataset (gse81928, [4]), Buendia’s dataset [5], Kappler’s dataset (gse151347, [6]) and Raymond’s dataset (gse104766, [7]). For each dataset, the R and p-values are as shown in the corresponding graph.


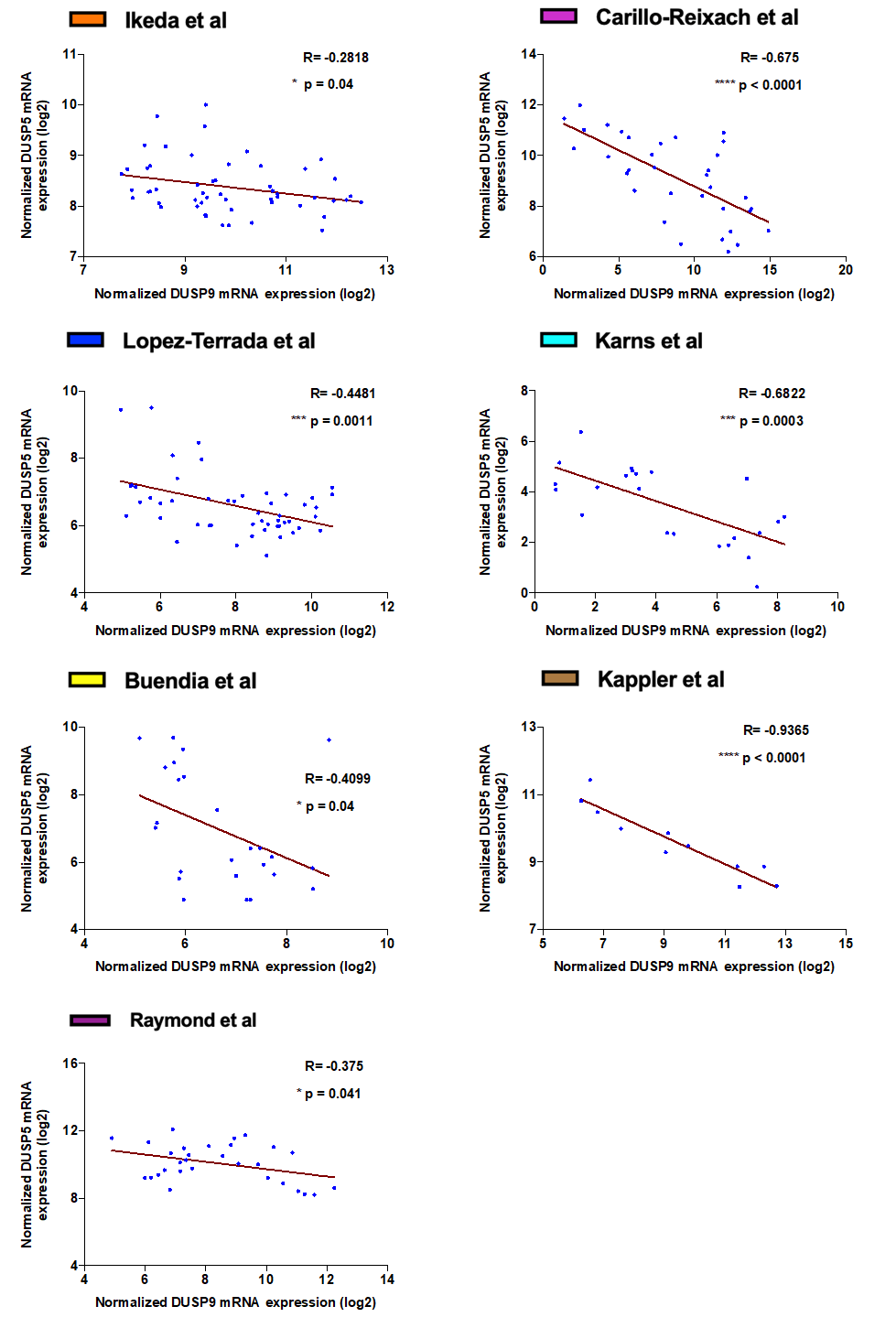


**Supplementary FIG. S27. Correlative analysis between *DUSP5* and *DUSP9* transcripts in hepatoblastoma.** Graphs show the two-tailed Pearson R correlations between *DUSP5* and *DUSP9* transcripts in HB samples from Ikeda’s dataset (gse131329, [1]), Carrillo-Reixach’s dataset (gse133039, [2]), Lopez-Terrada’s dataset (gse75271, [3]), Karns’s dataset (gse81928, [4]), Buendia’s dataset [5] and Kappler’s dataset (gse151347, [6]). For each dataset, the R and p-values are as shown in the corresponding graph.


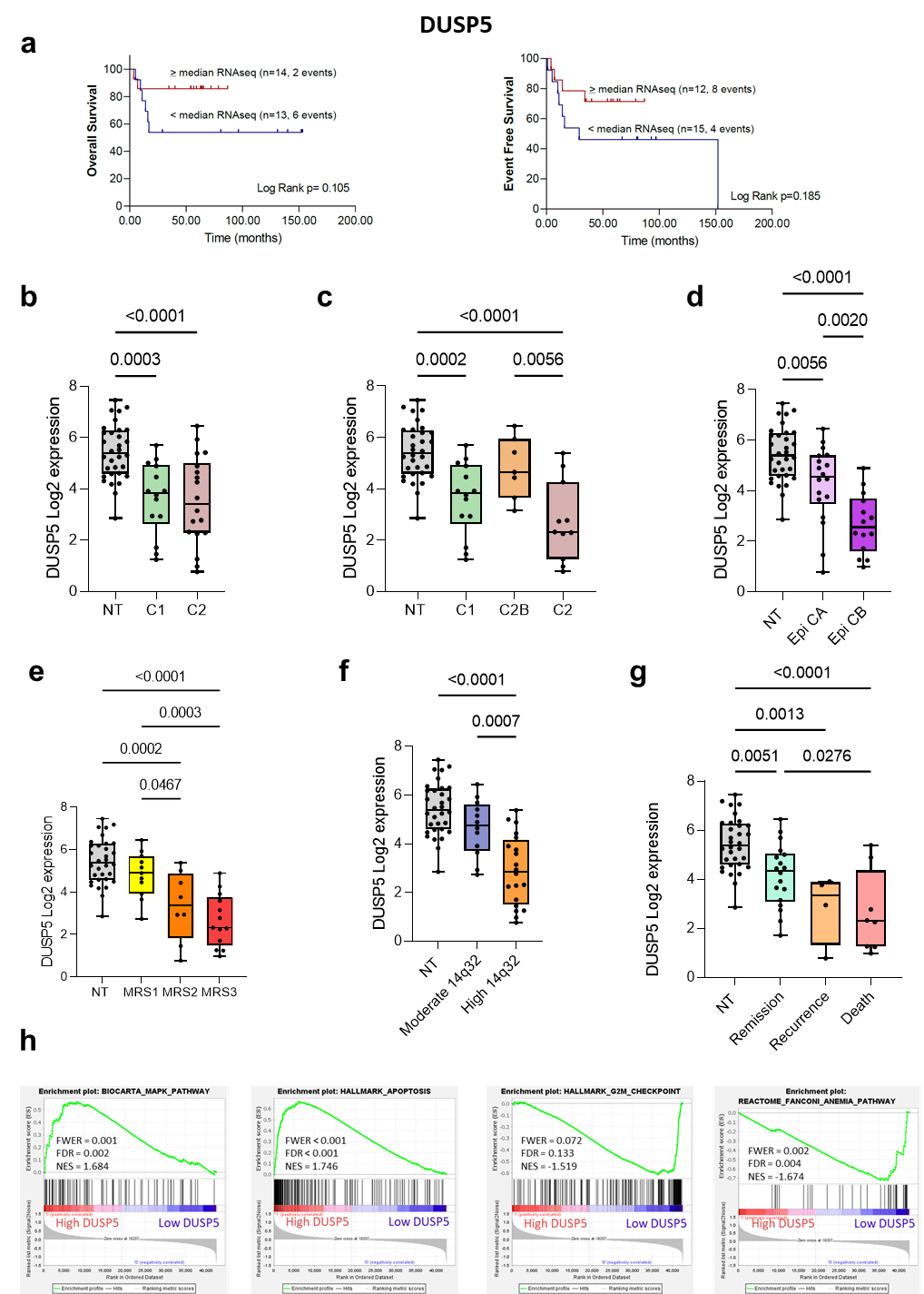


**Supplementary FIG. S28. Links between *DUSP5* mRNA expression and clinical data in hepatoblastoma.** (**a**) Overall survival (left) and event-free survival (right) Kaplan Meier plots for patients with follow-up of more than 2 years. Patients were categorized as high or low *DUSP5* mRNA expression according to the median of the tumoral RNAseq gene expression data. (**b-g**) Expression levels of *DUSP5* mRNA in NT (n=32), C1 (n=14) and C2 tumors (n=18) (**b**, Buendia’s dataset, [5]), in NT (n=32), C1 (n=14), C2B (n=7) and C2 tumors (n=11) (**c**, gse133039, [2]), in NT (n=32), Epi CA (n = 18) and Epi CB (n=14) (**d**), in NT (n=32), MRS-1 (n=11), MRS-2 (n=8) and MRS-3 (n=13) (**e**, gse133039, [2]), in NT (n=32), moderate (n=12) and high 14q32 expression tumors (n=20) (**f**) or in NT (n=32), patients in remission (n=18), in recurrence (n=4) and those deceased (n=8) (**g**, gse133039, [2]). (**h**) GSEA of *DUSP5* mRNA low expression and high expression samples categorized according to tumor median. FDR q value<0.25 indicate statistically significant trend. NES, Normalized Enrichment Score. Left in red, high expression of *DUSP5* mRNA; Right in blue, low expression of *DUSP5* mRNA. (**b-g**) One-way ANOVA test, ****p<0.0001, Tukey post-test. (**a-g**) p-values are as shown in the corresponding graph.


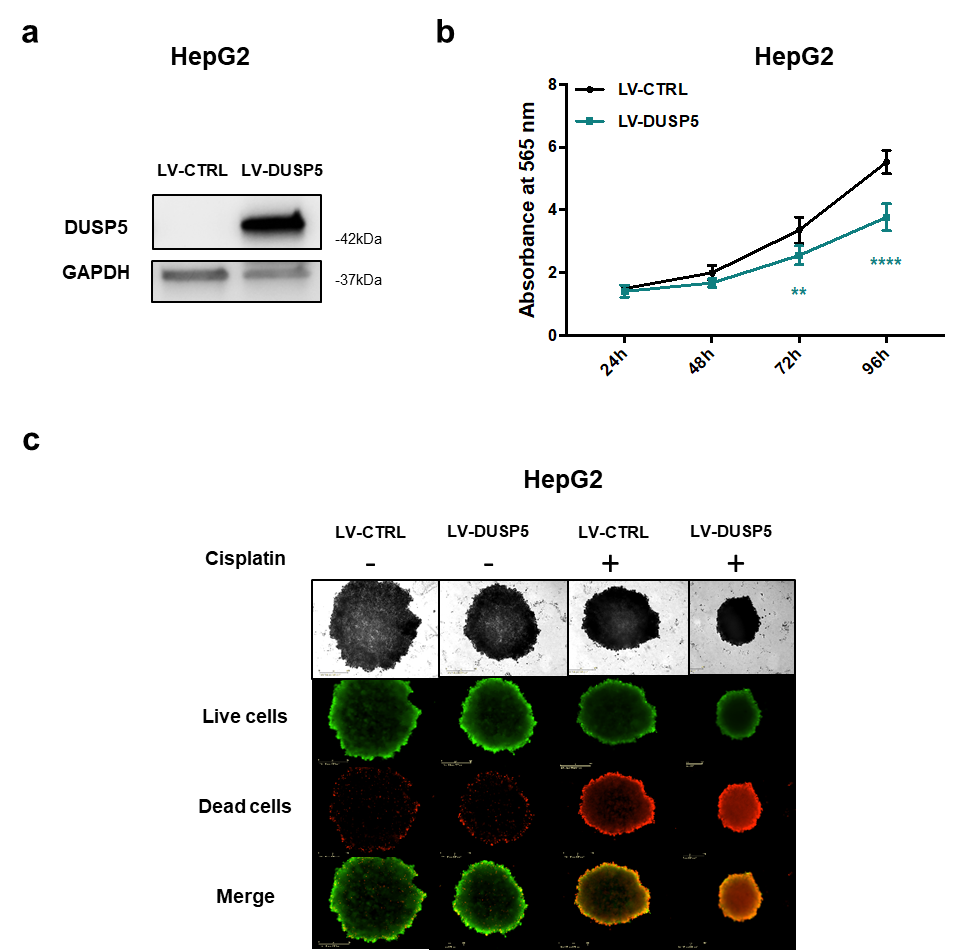


**Supplementary FIG. S29. *DUSP5 counteracts the growth and cisplatin resistance capacities of HepG2 cells*.** (**a**) Level of DUSP5 protein in HepG2 cells expressing DUSP5 or the CTRL cassette. (**b**) Growth (Absorbance at 565 nm) of CTRL and DUSP5-expressing HepG2 cells (n=3, Two way-ANOVA, ***p<0.001; Sidak's multiple comparisons post-test). (**c**) HepG2 cells expressing DUSP5 or the CTRL cassette were cultured as 3D spheroids and treated or not with cisplatin at IC_50_ dose. Top panels: phase contrast micrographs of representative spheroids. Bottom panels: live and dead cells stained with calcein-AM and ethidium homodimer-1 reagents. Representative images of four independent experiments. **p<0.01; ****p<0.0001.

**
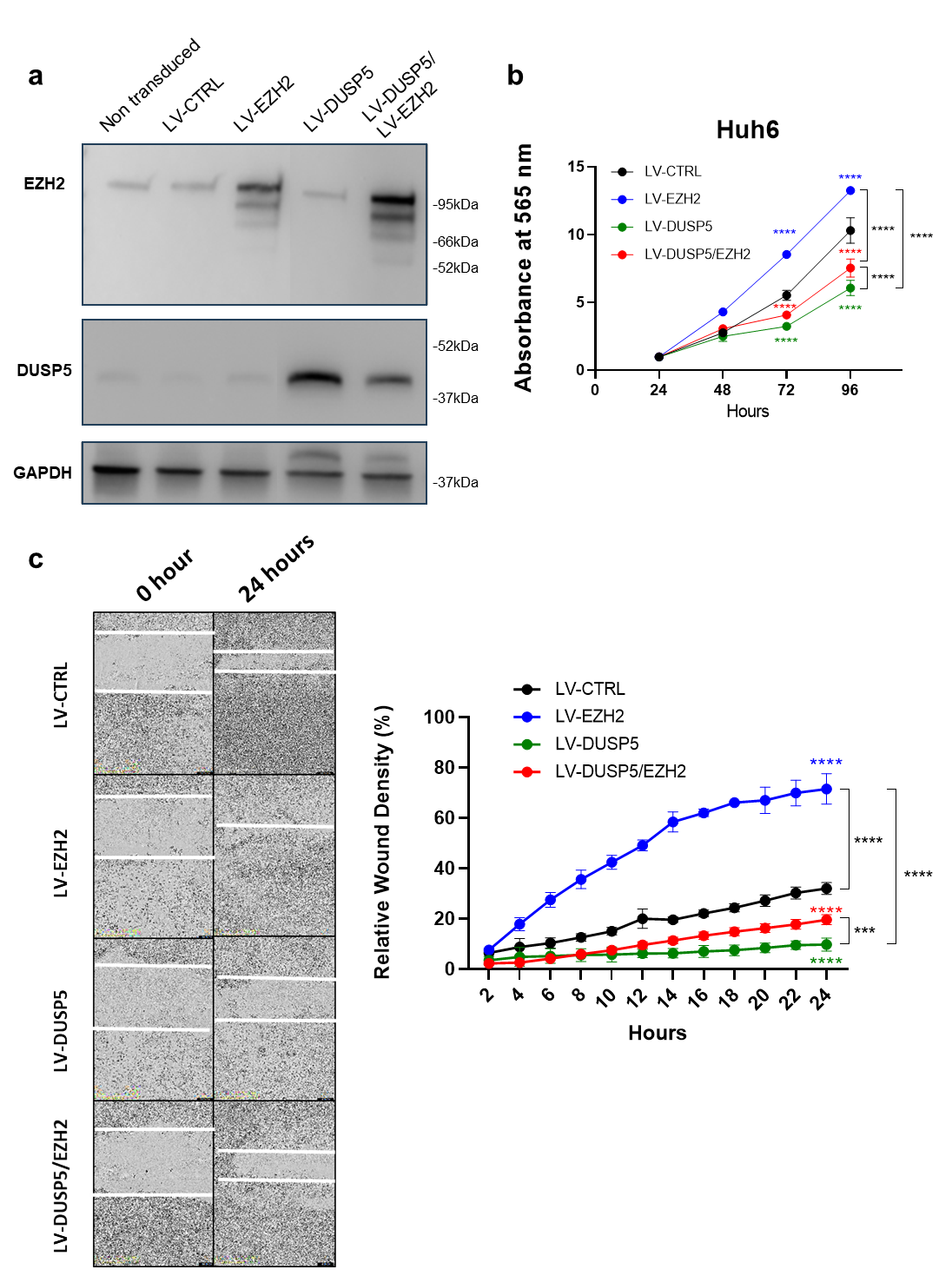
**

**Supplementary FIG. S30. *DUSP5 counteracts the oncogenic function of EZH2 in vitro in Huh6 cells*.** (a) Levels of EZH2 and DUSP5 in non-transduced Huh6 cells and Huh6 cells transduced by LV-CTRL, LV-EZH2, LV-DUSP5 or both as indicated. Representative blots of three experiments or more are shown in cropped images (loading control: GAPDH as shown). (b) Growth (Absorbance at 565 nm) of Huh6 cells ectopically expressing the control cassette (LV-CTRL), EZH2, DUSP5 or both (n=3, Two way-ANOVA, ****p<0.0001; Sidak's multiple comparisons post-test); (c) Left panel: Representative images of three independent experiments of migrating Huh6 cells ectopically expressing the control cassette (LV-CTRL), EZH2, DUSP5 or both at 0 and 24 h after wound-making. Right panel: Kinetic analysis of Huh6 cell migration following transduction with LV-CTRL, LV-EZH2, LV-DUSP5 or both (n=3, Two-way-ANOVA at 24 h, ****p<0.0001; Sidak's multiple comparisons post-test). ***p<0.001; ****p<0.0001.

**
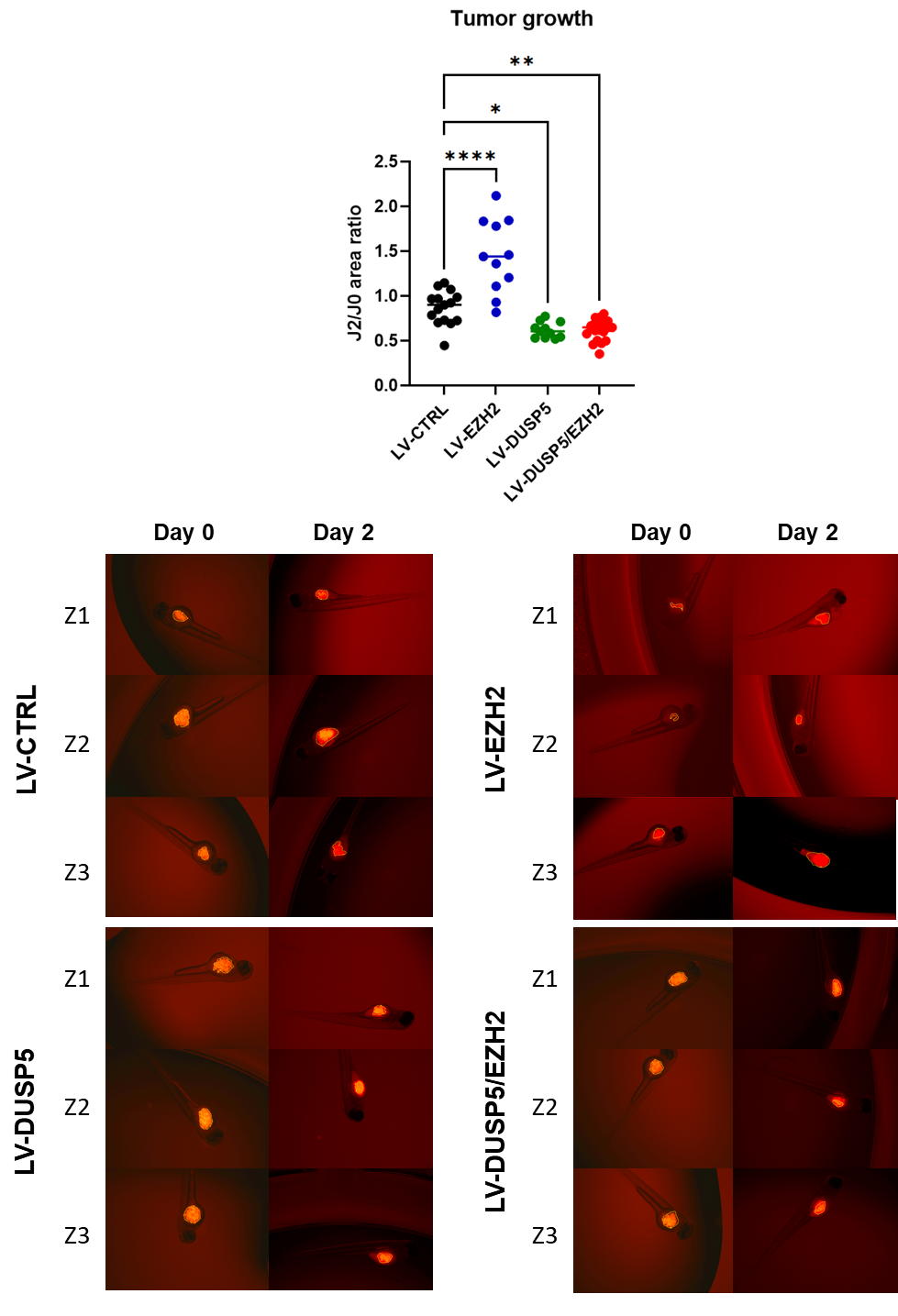
**

**Supplementary FIG. S31. *DUSP5 counteracts the oncogenic function of EZH2 in vivo in Huh6 cells*.** Top panel: Two-day tumor growth in zebrafish after implantation of Tomato-positive Huh6 cells ectopically expressing the control cassette (LV-CTRL), EZH2, DUSP5 or both (n=2, One way-ANOVA, ****p<0.0001; Sidak's multiple comparisons post-test). Bottom panel: Representative images of three zebrafishes with tumor (Z) at Day 0 and Day 2 after implantation of Huh6 cells ectopically expressing the control cassette (LV-CTRL), EZH2, DUSP5 or both. *p<0.05; **p<0.01; ****p<0.0001.


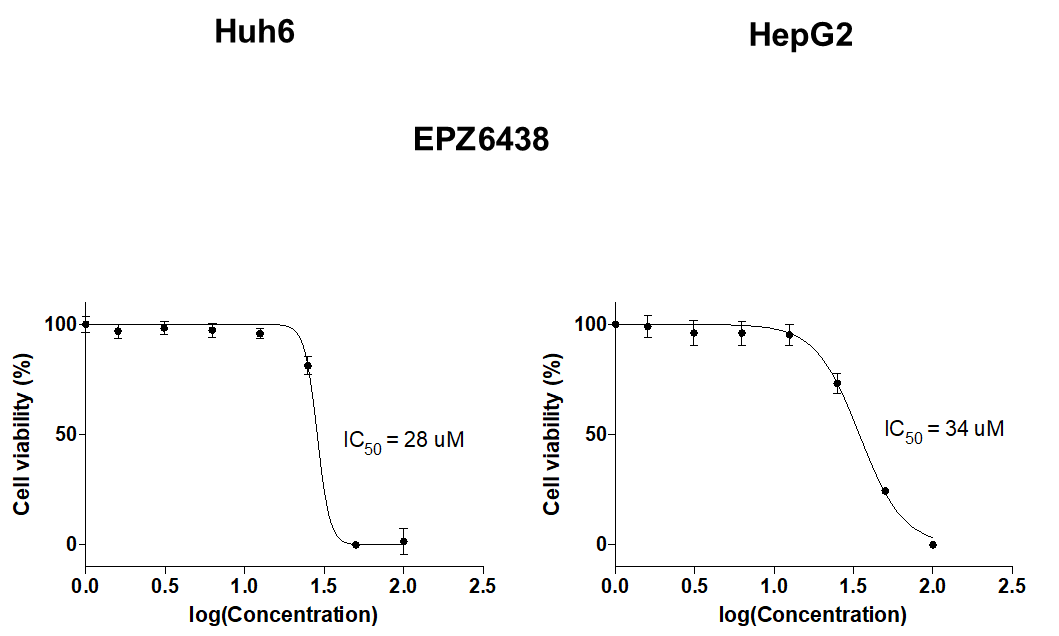


**Supplementary FIG. S32. *In vitro* response of hepatoblastoma cells to tazemetostat.** Graphs show the percentage of viable Huh6 (left) or HepG2 (right) cells treated with increasing concentrations of tazemetostat (also known as EPZ6438) (n=3; bars = means +/- SD; the IC50 is as shown).


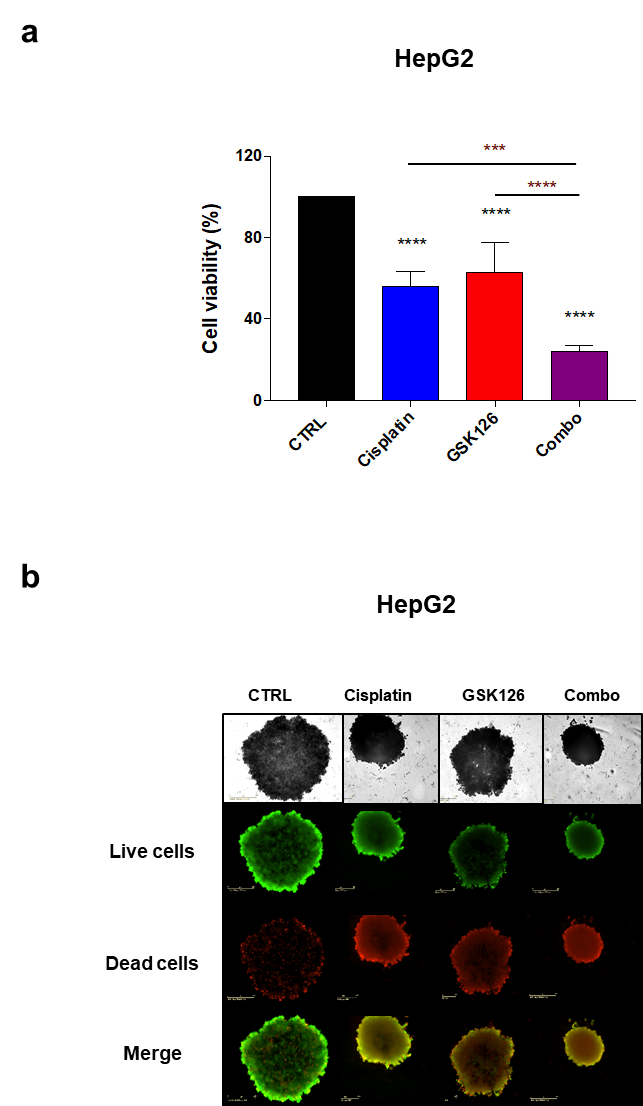


**Supplementary FIG. S33. GSK126 and cisplatin collaborate to eliminate HepG2 cells *in vitro*.** (**a**) Graphs show the percentage of viable HepG2 cells cultured as monolayer and treated or not for 24 h by cisplatin at IC_50_, and then treated or not for 48 hr by GSK126 at IC_50_, or by the combo. Bar graphs recapitulate means ± SD (n=5, One way-ANOVA, ****p<0.0001; Sidak's multiple comparisons post-test). CTRL: drug solvent. (**b**) Images showing live and dead HepG2 cells cultured as spheroids and treated with drug(s) as described in **a**. Top panels: phase contrast micrographs of representative spheroids. Bottom panels: HepG2 cells stained with calcein-AM and ethidium homodimer-1 reagents. Representative images of four independent experiments.


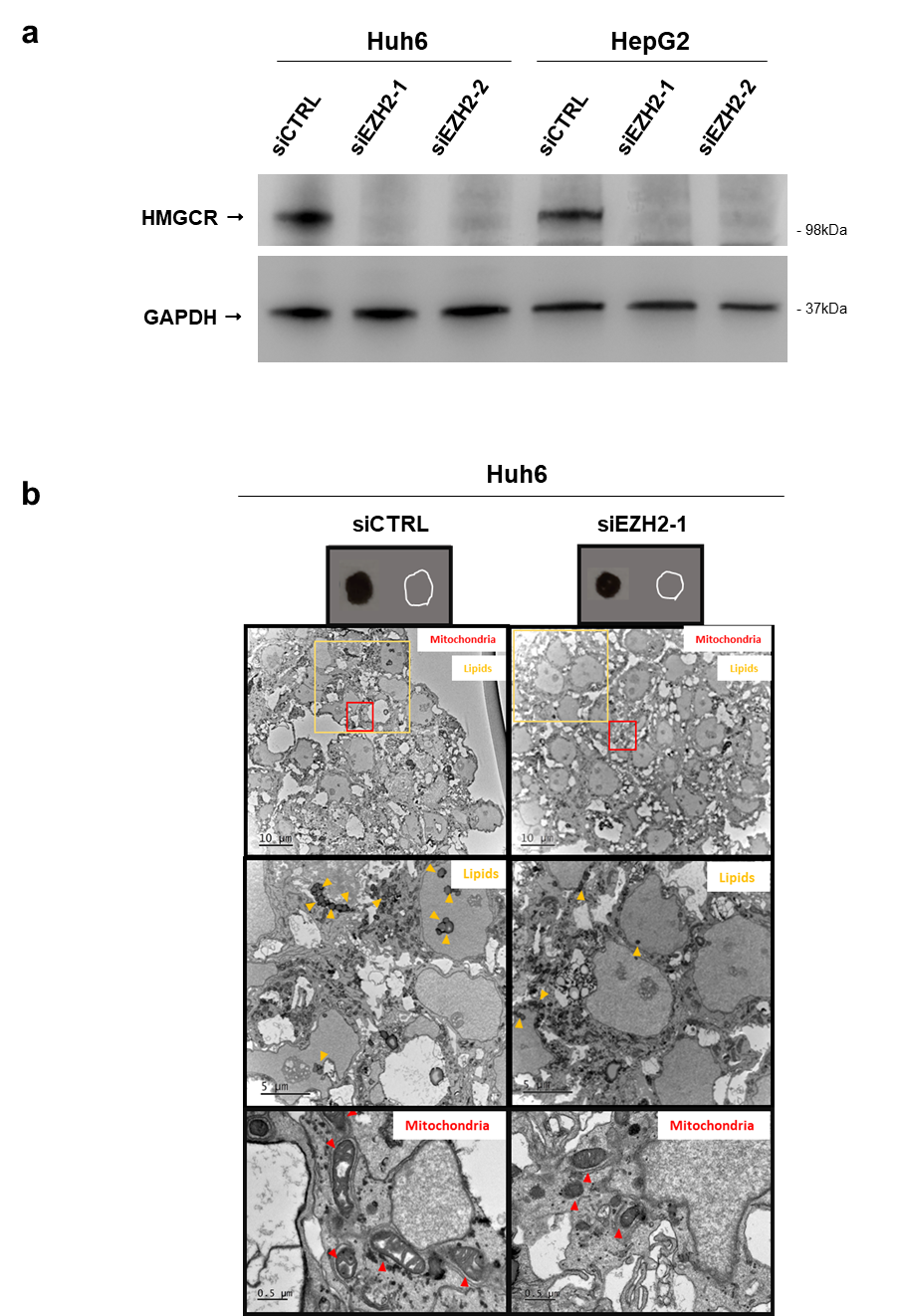


**Supplementary FIG. S34. *EZH2* silencing decreases the amount of HMGCR protein, intracellular lipid droplets** **and mitochondria size in hepatoblastoma cells.** (**a**) Level of HMGCR protein in siCTRL *versus* *EZH2*-depleted Huh6 (left) or HepG2 (right) cells (two EZH2 siRNAs used as indicated). Representative blots of three independent experiments are shown in cropped images using non-denatured loaded proteins (loading control: GAPDH). (**b**) Top panels: representative images of fixed spheroids in black with the corresponding mask (empty white line). Bottom panels: representatives electron microscopy images of siCTRL *versus* *EZH2*-depleted Huh6 cells using siEZH2-1 with magnifications increasing from the top to bottom panels. Yellow and red arrow heads show intracellular lipid droplets and mitochondria, respectively. Scale bars are shown at the bottom left of each image.


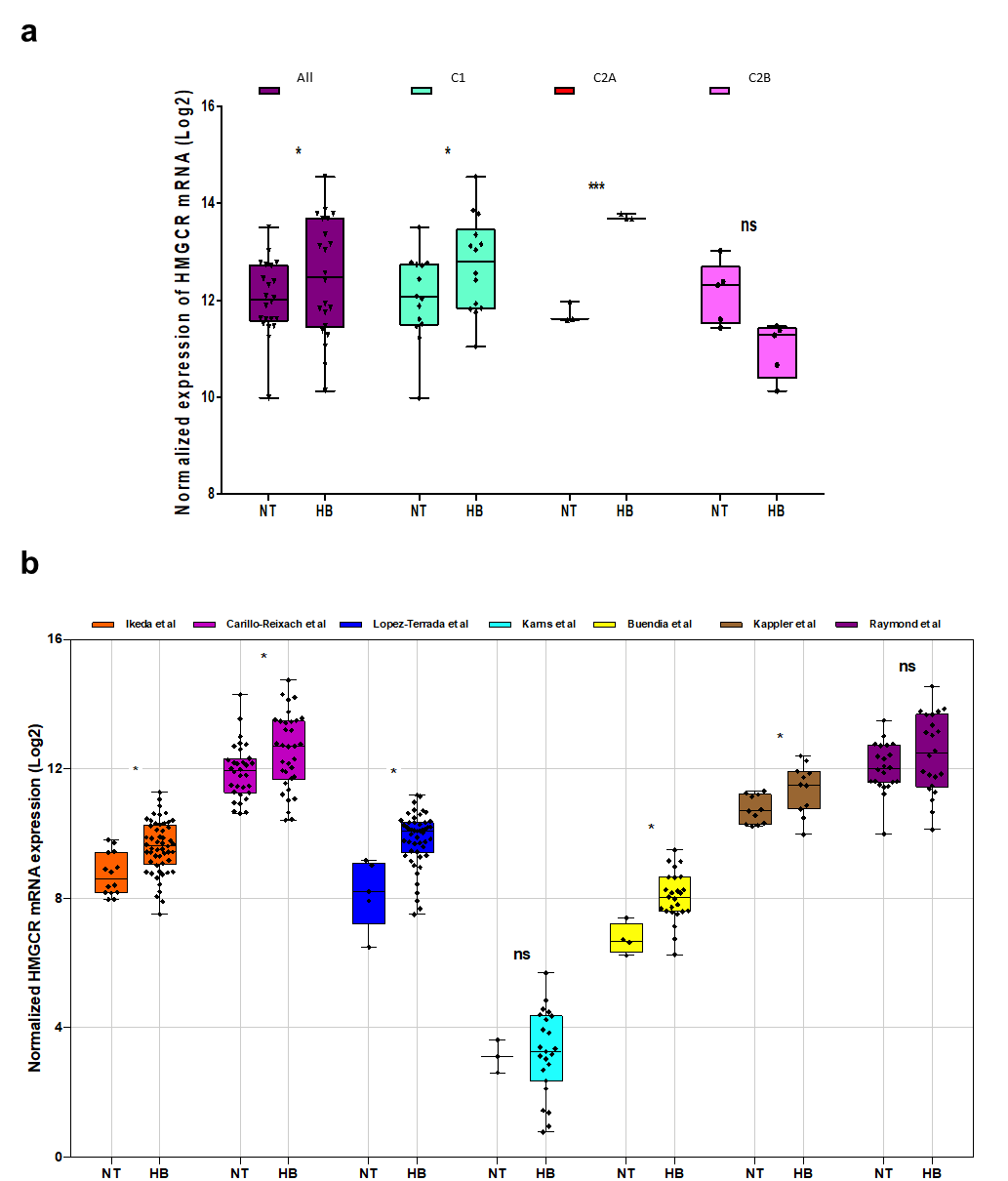


**Supplementary FIG. S35.** **Expression of *HMGCR* transcript in hepatoblastoma.** (**a**) Expression of *HMGCR* transcript in C1, C2A and C2B subgroups and NT samples from Raymond’s dataset (gse104766, [7]) (Wilcoxon matched pairs signed rank test). (**b**) Expression of *HMGCR* transcript in HB and NT samples from Ikeda’s dataset (gse131329, [1]), Carrillo-Reixach’s dataset (gse133039, [2]), Lopez-Terrada’s dataset (gse75271, [3]), Karns’s dataset (gse81928, [4]), Buendia’s dataset [5] and Kappler’s dataset (gse151347, [6]). Unpaired Mann & Whitney test. ns, not significant. *p<0.05; **p<0.01; ***p<0.001.


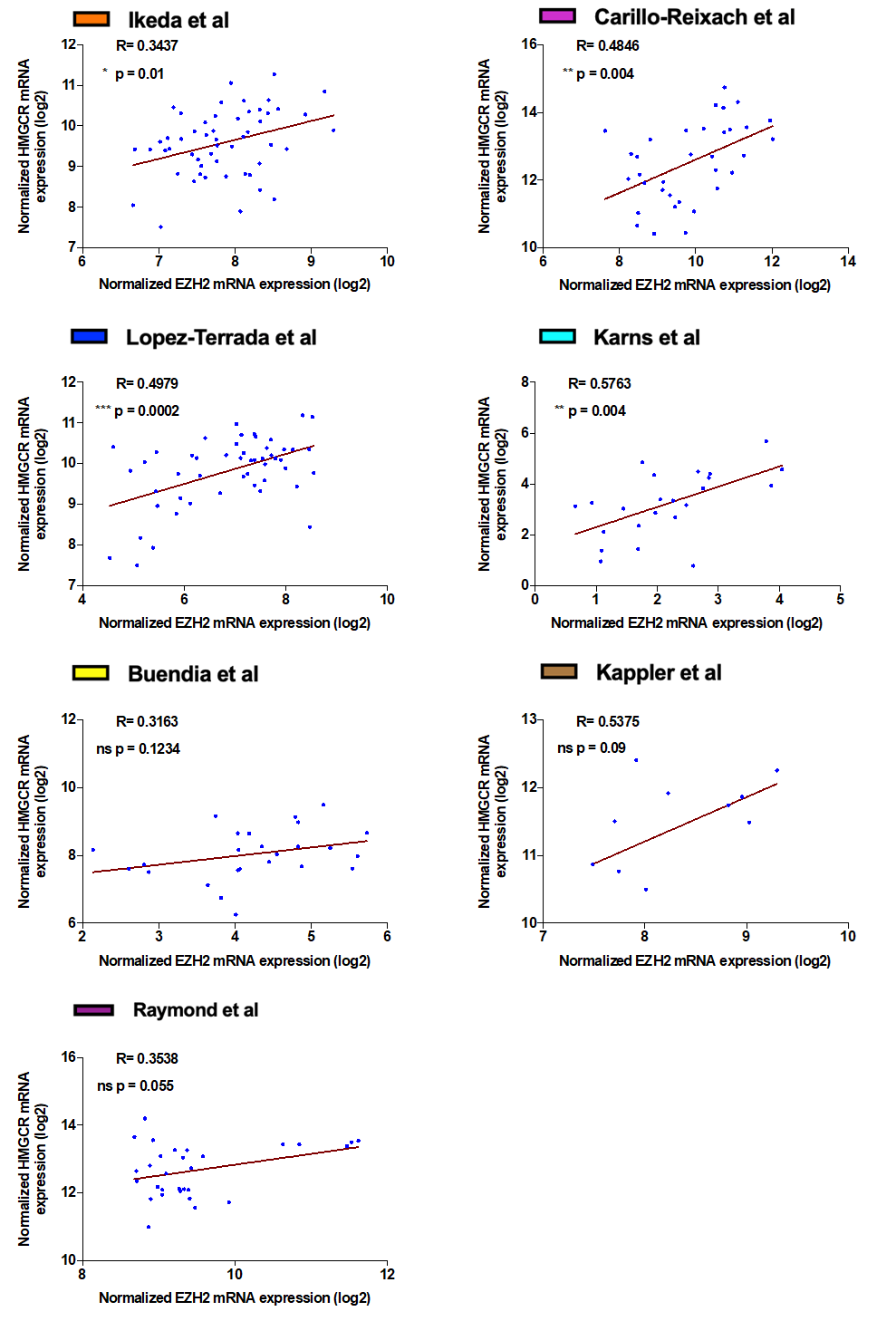


**Supplementary FIG. S36. Correlative analysis between *EZH2* and *HMGCR* transcripts in hepatoblastoma.** Graphs show the two-tailed Pearson R correlations between *HMGCR* and *EZH2* transcripts in HB samples from Ikeda’s dataset (gse131329, [1]), Carrillo-Reixach’s dataset (gse133039, [2]), Lopez-Terrada’s dataset (gse75271, [3]), Karns’s dataset (gse81928, [4]), Buendia’s dataset [5], Kappler’s dataset (gse151347, [6]) and Raymond’s dataset (gse104766, [7]). For each dataset, the R and p-values are as shown in the corresponding graph.


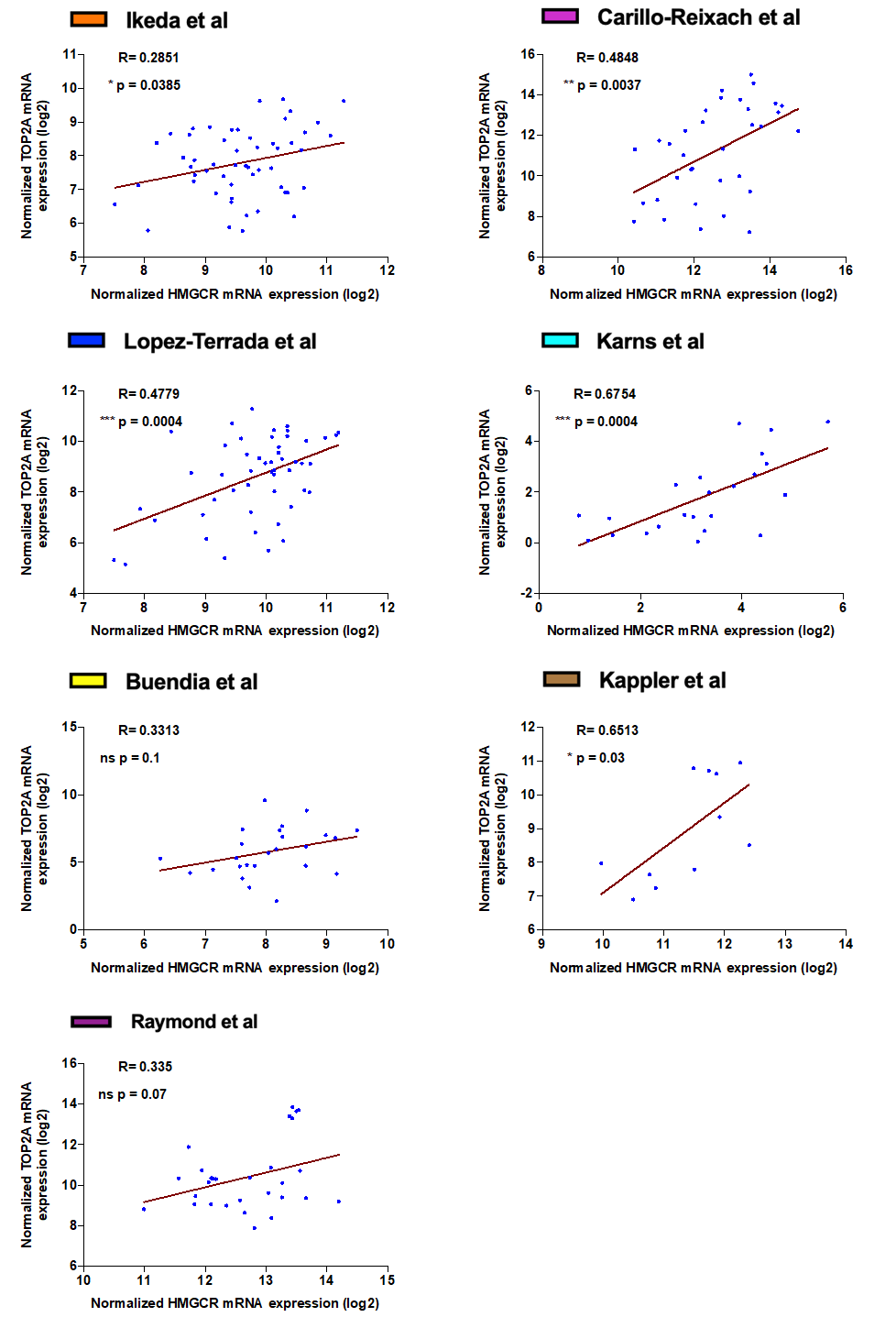


**Supplementary FIG. S37. Correlative analysis between *HMGCR* and *TOP2A* transcripts in hepatoblastoma.** Graphs show the two-tailed Pearson R correlations between *HMGCR* and *TOP2A* transcripts in HB samples from Ikeda’s dataset (gse131329, [1]), Carrillo-Reixach’s dataset (gse133039, [2]), Lopez-Terrada’s dataset (gse75271, [3]), Karns’s dataset (gse81928, [4]), Buendia’s dataset [5], Kappler’s dataset (gse151347, [6]) and Raymond’s dataset (gse104766, [7]). For each dataset, the R and p-values are as shown in the corresponding graph.

**
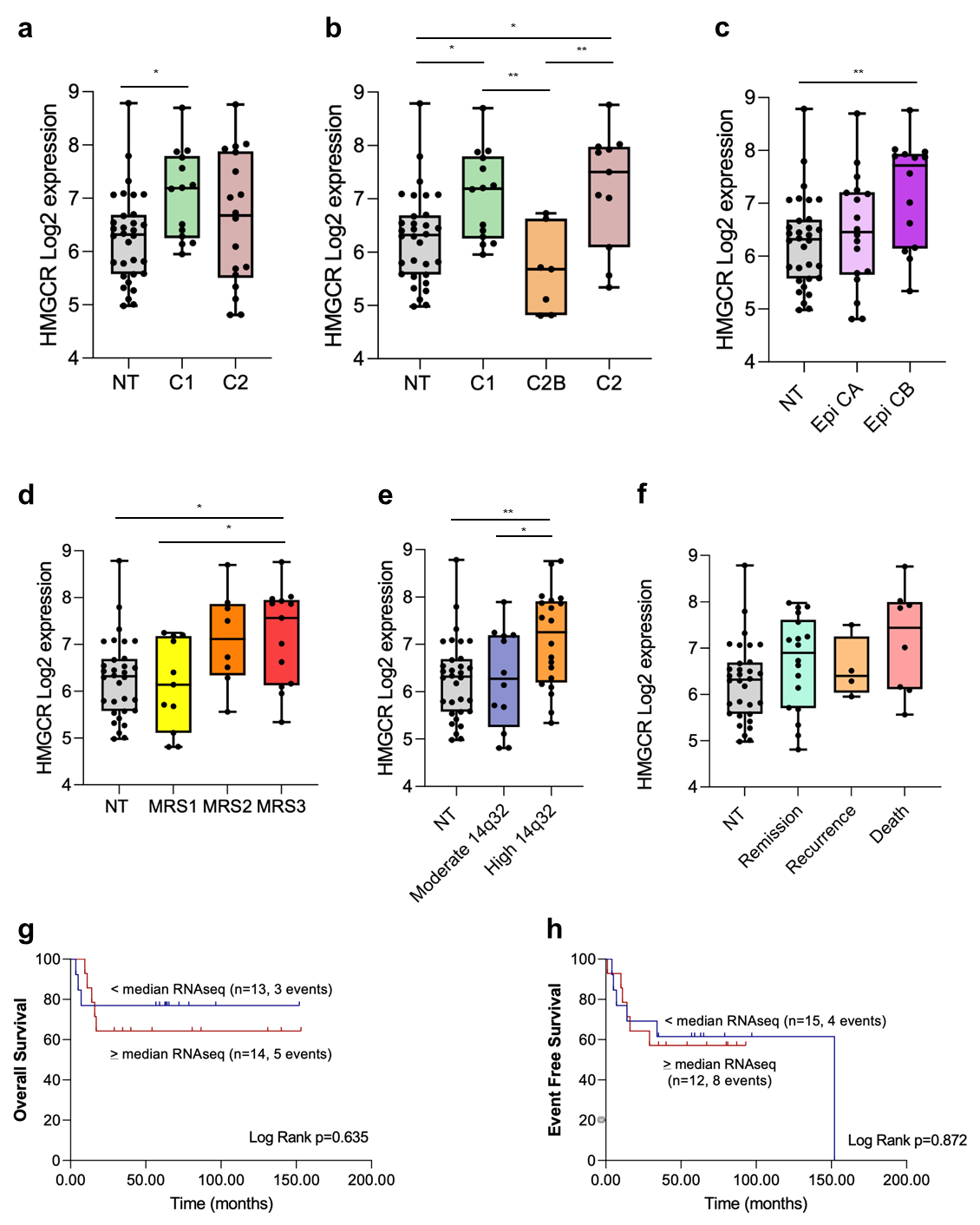
**

**Supplementary FIG. S38. Links between *HMGCR* transcript expression and clinical data in hepatoblastoma.** (**a-f**) Expression levels of *HMGCR* mRNA in NT (n=32), C1 (n=14) and C2 tumors (n=18) (**a**, Buendia’s dataset, [5]), in NT (n=32), C1 (n=14), C2B (n=7) and C2 tumors (n=11) (**b**, gse133039, [2]). in NT (n=32), Epi CA (n = 18) and Epi CB (n=14) (**c**), in NT (n=32), MRS-1 (n=11), MRS-2 (n=8) and MRS-3 (n=13) (**d**, gse133039, [2]), in NT (n=32), moderate (n=12) and high 14q32 expression tumors (n=20) (**e**) or in NT (n=32), patients in remission (n=18), in recurrence (n=4) and those deceased (n=8) (**f**, gse133039, [2]). (**a-f**) One-way ANOVA test; Tukey post-test. (**g-h**) Overall survival (**g**) and event-free survival (**h**) Kaplan Meier plots for patients with follow-up of more than 2 years. Patients were categorized as high or low *HMGCR* mRNA expression according to the median of the tumoral RNAseq gene expression data. p-values are as shown in the corresponding graph. *p<0.05; **p<0.01.


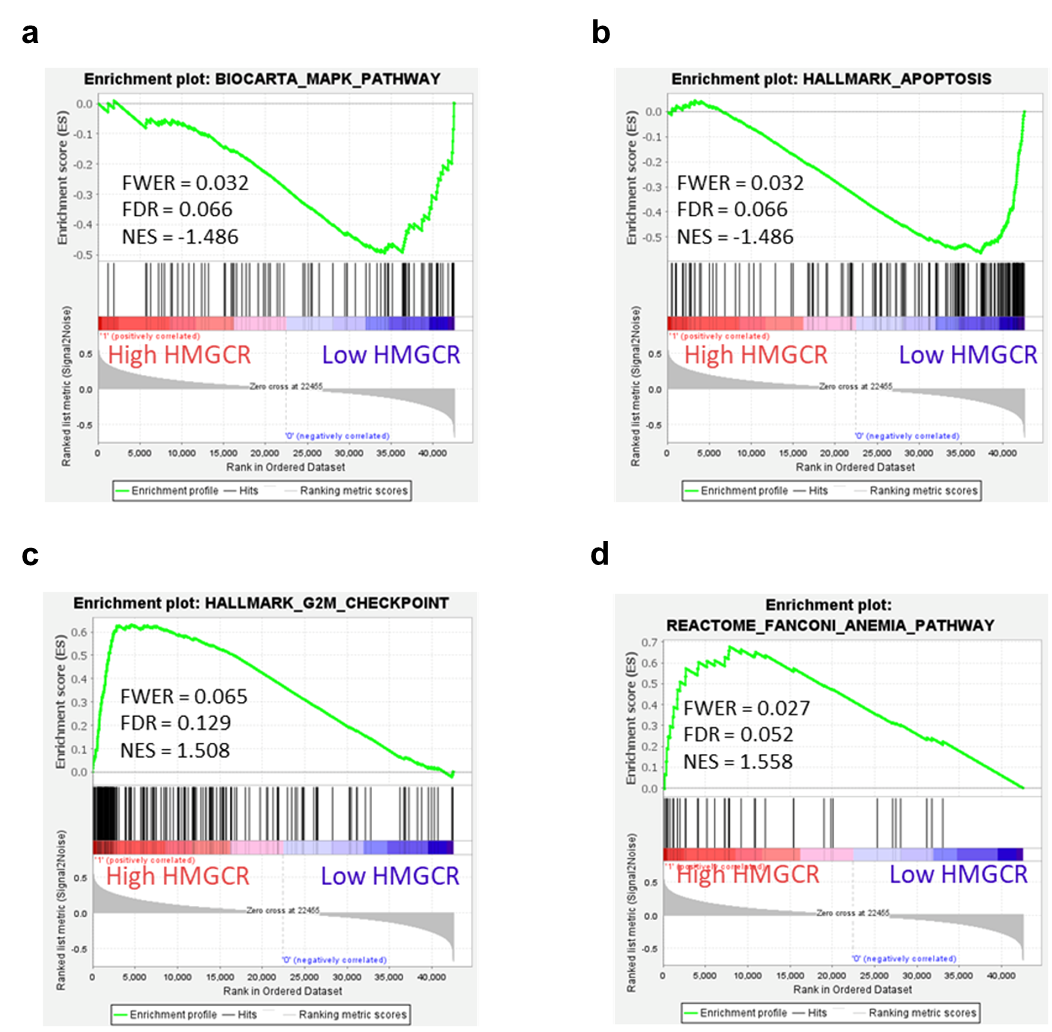


**Supplementary FIG. S39. Additional links between *HMGCR*** **transcript expression and clinical data in hepatoblastoma.** GSEA of *HMGCR* mRNA low expression and high expression samples categorized according to tumor median. FDR q value<0.25 indicate statistically significant trend. NES, Normalized Enrichment Score. Left in red, high expression of *HMGCR* mRNA; Right in blue, low expression of *HMGCR* mRNA.

**
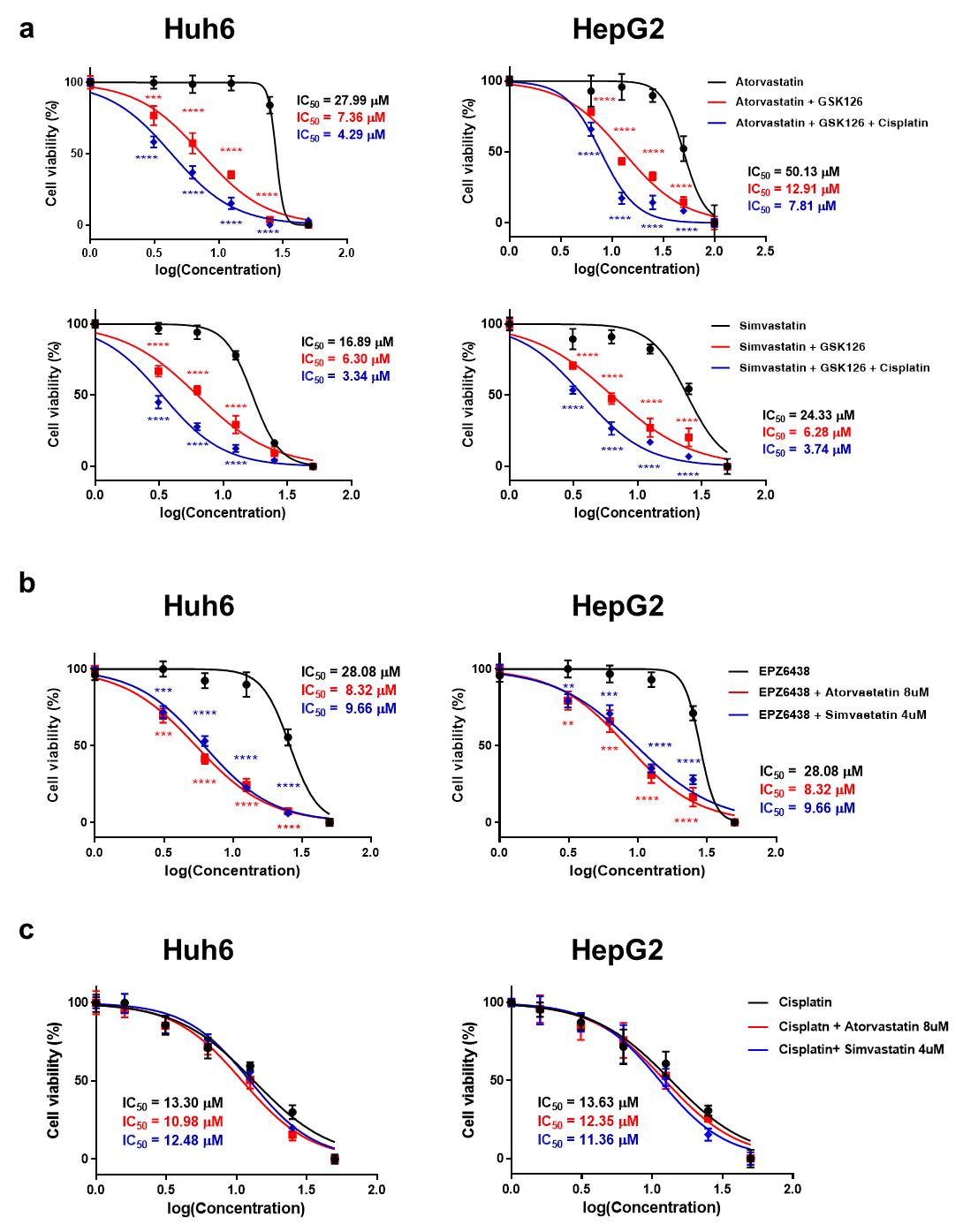
**

**Supplementary FIG. S40. Statins sensitize hepatoblastoma cells to EZH2 inhibitors but not to cisplatin.** (**a**) Graphs show the percentage of viable Huh6 (left) and HepG2 (right) cells treated with increasing doses of atorvastatin (top graphs) or simvastatin (bottom graphs) alone or in combination with a non-cytotoxic dose of GSK126 (3 and 4 µM for Huh6 and HepG2 cells, respectively) in absence or in presence of a non-cytotoxic dose of cisplatin (3 µM for both Huh6 and HepG2 cells) (n=3; bars = means +/- SD; Two way-ANOVA, ***p<0.00001; Sidak's multiple comparisons post-test). (**b**) Graphs show the percentage of viable Huh6 (left) or HepG2 (right) cells treated with increasing doses of EPZ6438 in combination or not with a non-toxic dose of atorvastatin (8 µM) or simvastatin (4 µM) as indicated (n=3; bars = means +/- SD; Two way-ANOVA, ***p<0.001; Sidak's multiple comparisons post-test). (**c**) Graphs show the percentage of viable Huh6 (left) and HepG2 (right) cells treated with increasing concentrations of cisplatin alone or in combination with a non-cytotoxic dose of simvastatin (4 µM) or atorvastatin (8 µM) (n=3; bars = means +/- SD; Two way-ANOVA, not significant; Sidak's multiple comparisons post-test). **p<0.01; ***p<0.001; ****p<0.0001.


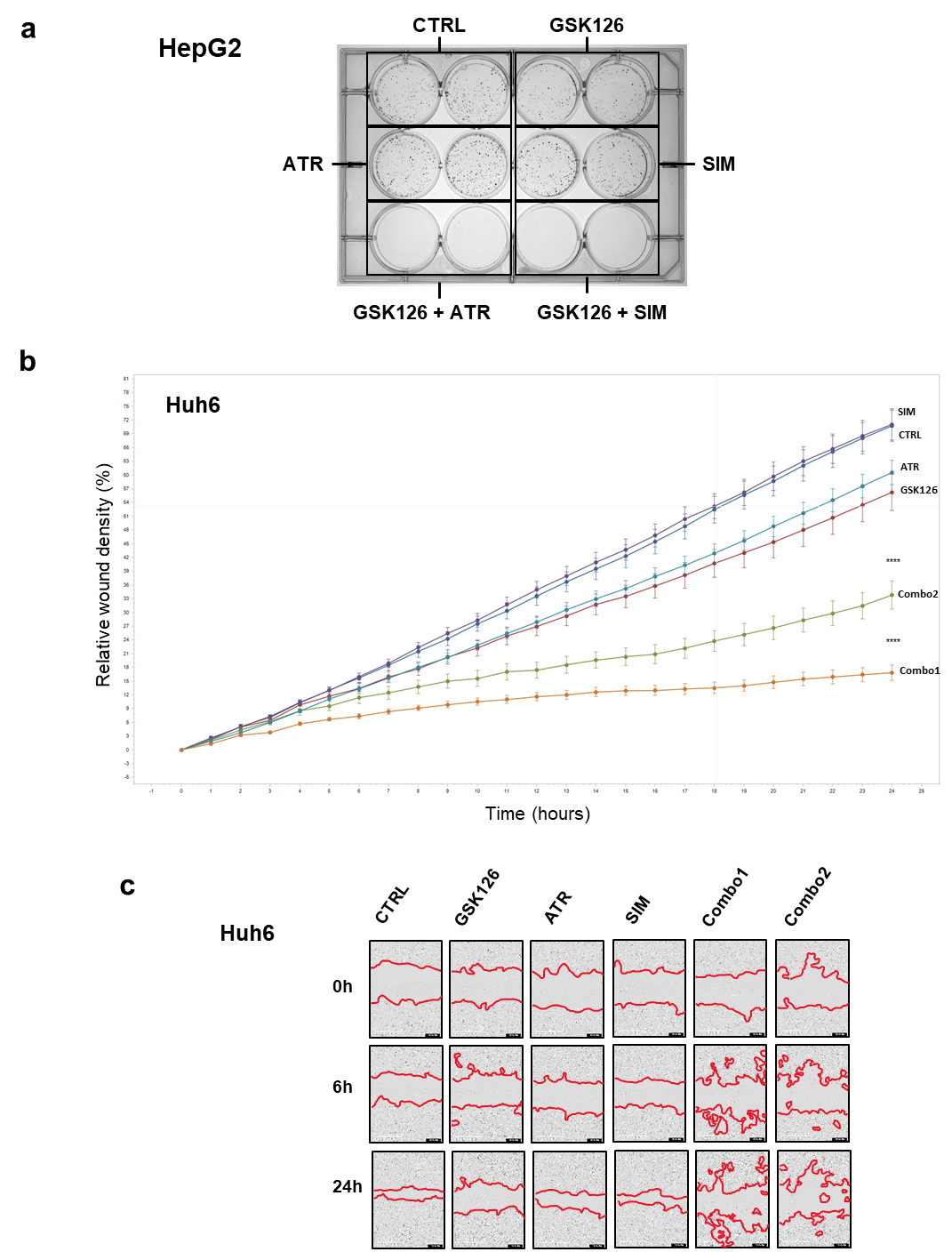


**Supplementary FIG. S41. GSK126 and statins cooperate to inhibit the clonogenic and migratory capacities of HB cells and to induce cell apoptosis.** (**a**) Survival and proliferation of HepG2 cells treated with DMSO (control: CTRL), GSK126 at IC25 dose (4 µM), a statin (ATR: atorvastatin at 8 µM; SIM: simvastatin at 4 µM) or the combination of both. Representative images of three independent experiments. (**b**) Kinetic analysis of Huh6 cell migration treated for 24 h with DMSO (control: CTRL), GSK126 at IC25 dose (3 µM), a statin (ATR: atorvastatin at 8 µM; SIM: simvastatin at 4 µM) or a combination of both. In this panel and the following: Combo 1: GSK126 + atorvastatin; Combo 2 GSK126 + simvastatin (n=3, Two-way-ANOVA at 24 h, ****p<0.0001; Sidak's multiple comparisons post-test). (**c**) Representative images of three independent experiments of migrating Huh6 cells at 6 and 24 h after wound-making and treatment with the indicated drug or combo. ****p<0.0001.


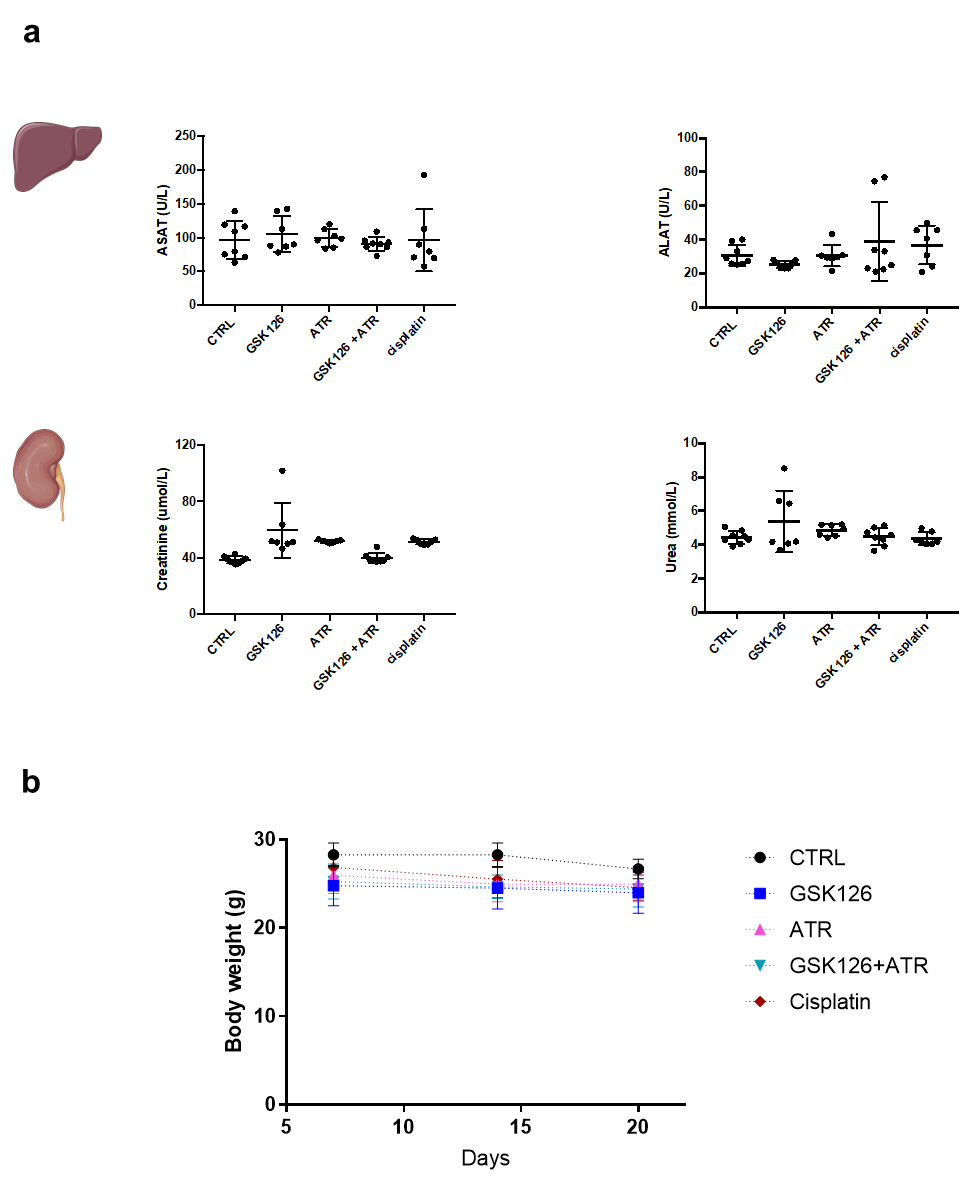


**Supplementary FIG. S42. The combination of GSK126 and statin is not toxic in mice.** (**a**) Blood circulating levels of ASAT, ALAT, creatinine and urea in each group of treated mice. ns, not significant. (**b**) Mice weight kinetics after treatment with the indicated drug or combination.


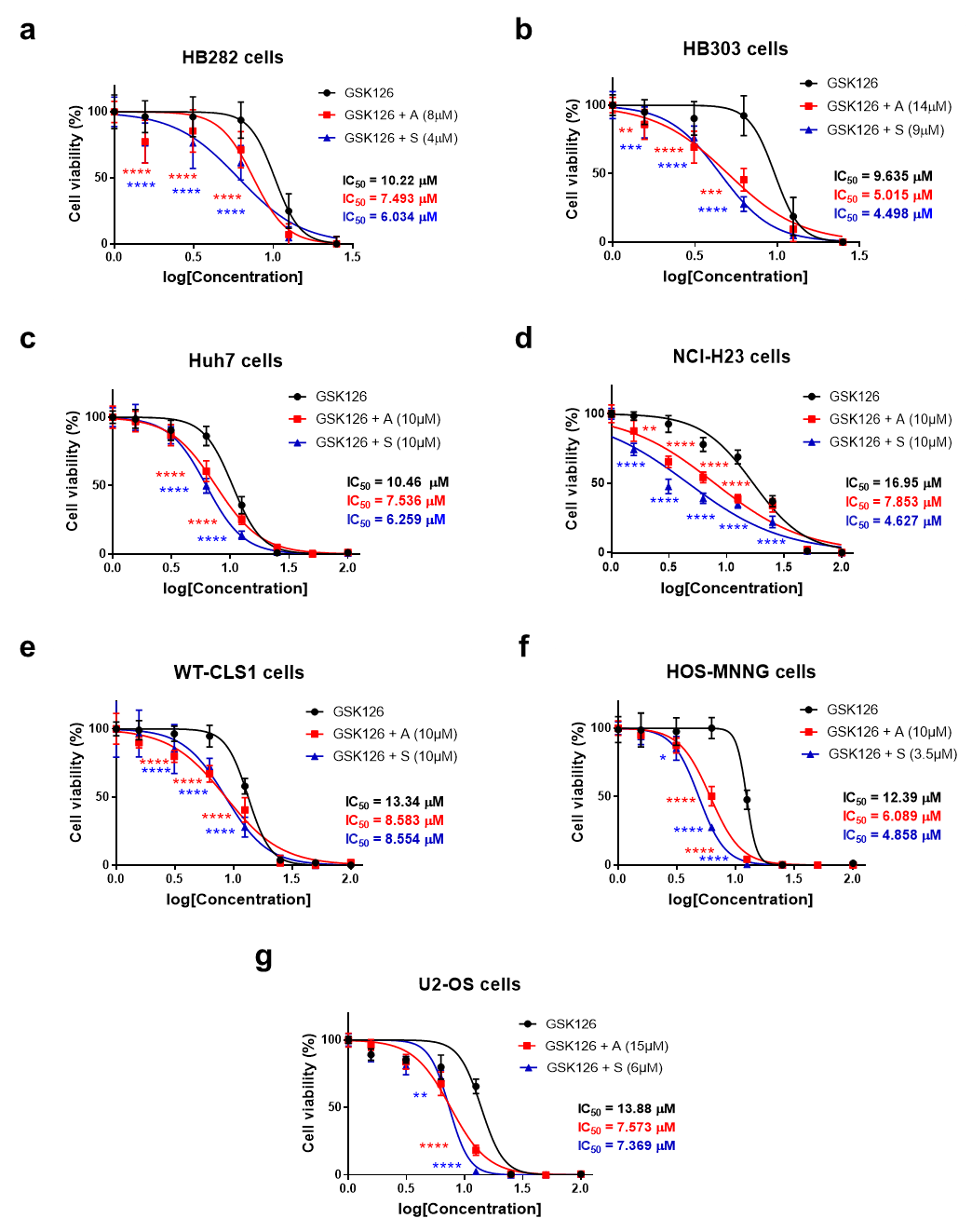


**Supplementary FIG. S43. Statins sensitize tumor cells to EZH2 inhibitor GSK126.** (**a-g**) Graphs show the percentage of viable HB282 (**a**), HB303 (**b**), Huh7 (**c**), NCI-H23 (**d**), WT-CLS1 (**e**), HOS-MNNG (**f**) and U2-OS cells (**g**) cells treated with increasing doses of GSK126 alone or in combination with a non-cytotoxic dose (as indicated in brackets) of atorvastatin (A) or simvastatin (S) (n=3 to 4; bars = means +/- SD; Two way-ANOVA, ****p<0.0001; Sidak's multiple comparisons post-test). *p<0.05; **p<0.01; ***p<0.001; ****p<0.0001.


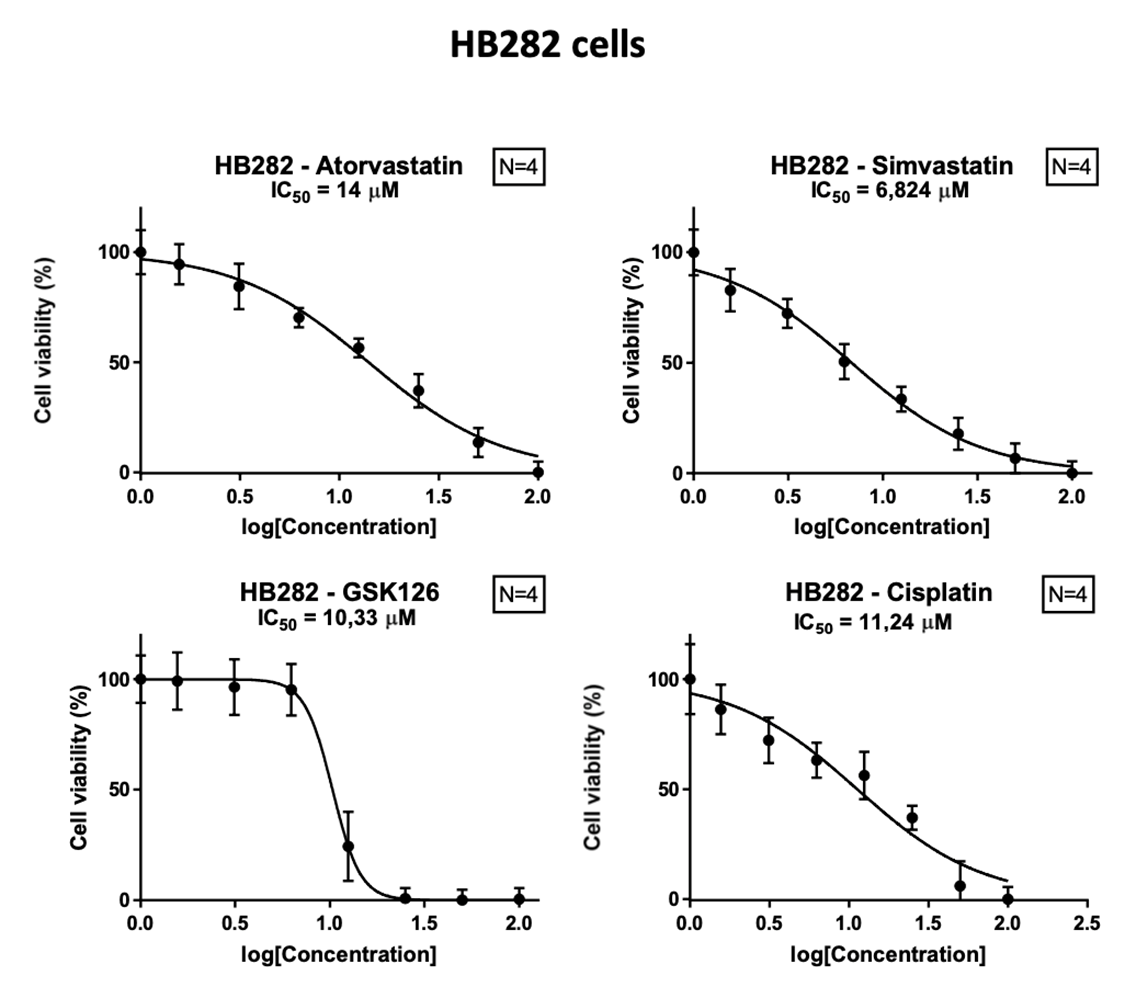


**Supplementary FIG. S44. Effect of drugs on hepatoblastoma-derived HB282 cell growth.** Graphs show the percentage of viable PDX HB282 cells treated with increasing concentrations of atorvastatin (top left), simvastatin (top right), GSK126 (bottom left) or cisplatin (bottom right) (n=4; horizontal bars = means). For each drug, the IC_50_ is shown above the corresponding graph.


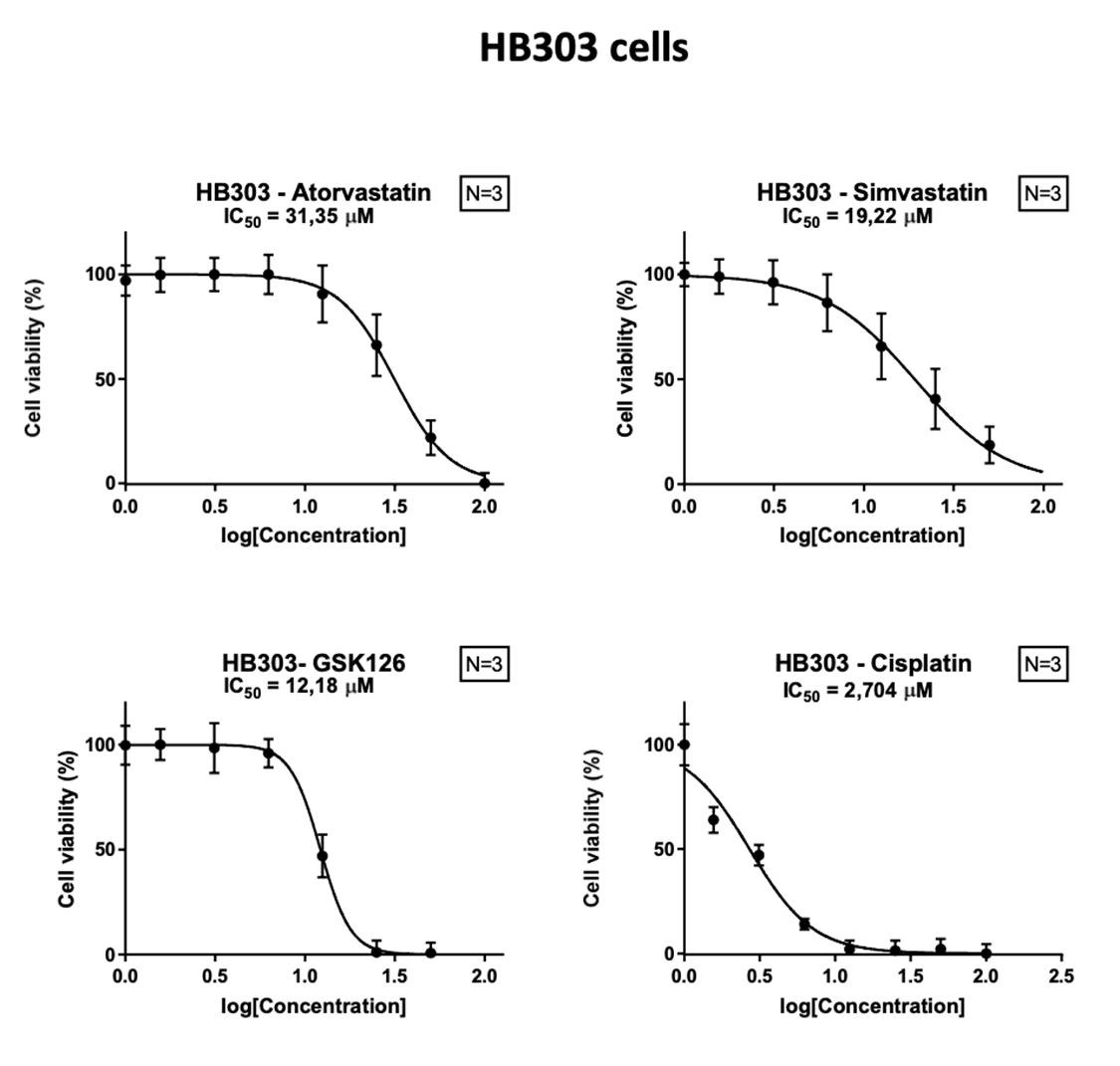


**Supplementary FIG. S45. Effect of drugs on hepatoblastoma -derived HB303 cell growth.** Graphs show the percentage of viable PDX HB303 cells treated with increasing concentrations of atorvastatin (top left), simvastatin (top right), GSK126 (bottom left) or cisplatin (bottom right) (n=3; horizontal bars = means). For each drug, the IC_50_ is shown above the corresponding graph.


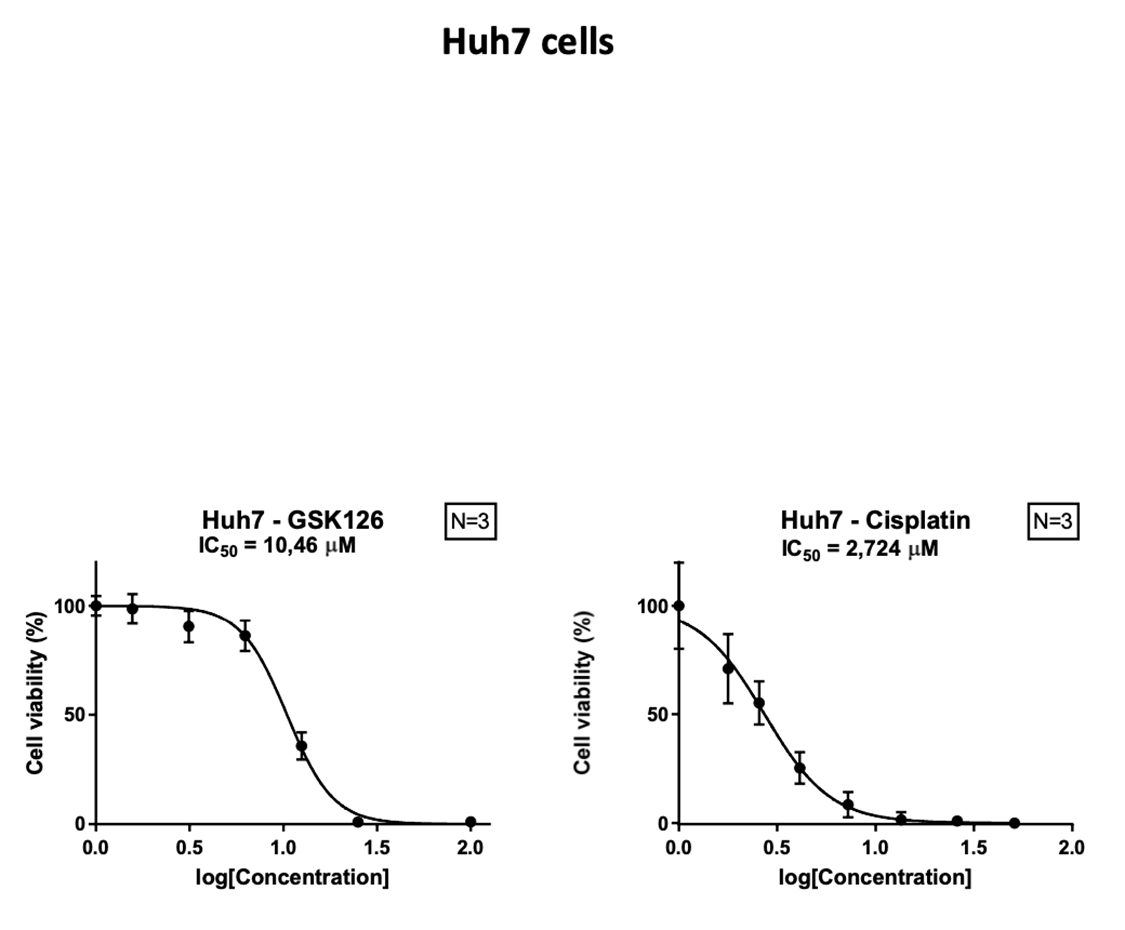


**Supplementary FIG. S46. Effect of drugs on hepatocellular carcinoma-derived Huh7 cell growth.** Graphs show the percentage of viable Huh7 cells treated with increasing concentrations of GSK126 (left) or cisplatin (right) (n=3; horizontal bars = means). For each drug, the IC_50_ is shown above the corresponding graph. Atorvastatin and simvastatin had no inhibitory effect on Huh7 cell growth at a dose up to 100 µM.


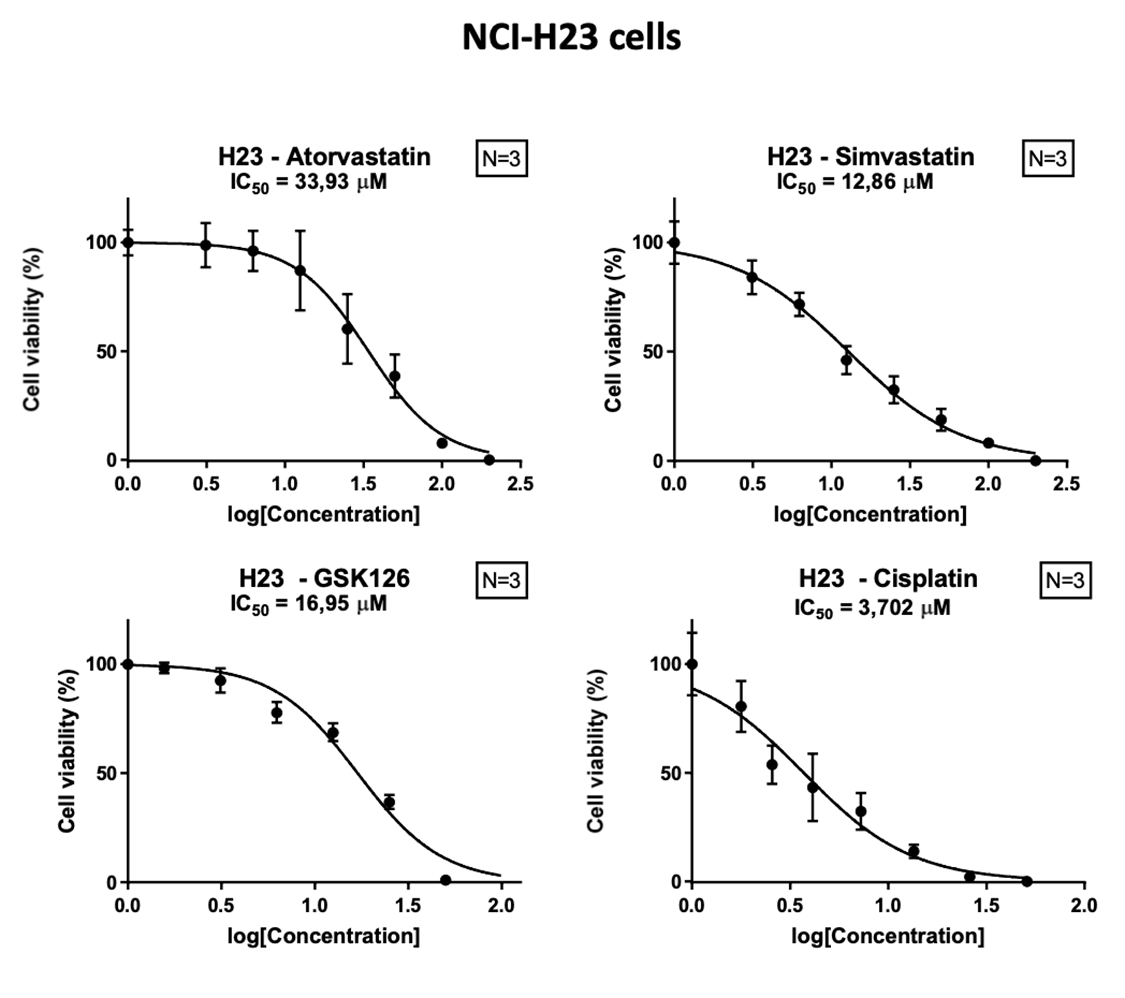


**Supplementary FIG. S47. Effect of drugs on lung carcinoma-derived NCI-H23 cell growth.** Graphs show the percentage of viable NCI-H23 cells treated with increasing concentrations of atorvastatin (top left), simvastatin (top right), GSK126 (bottom left) or cisplatin (bottom right) (n=3; horizontal bars = means). For each drug, the IC_50_ is shown above the corresponding graph.


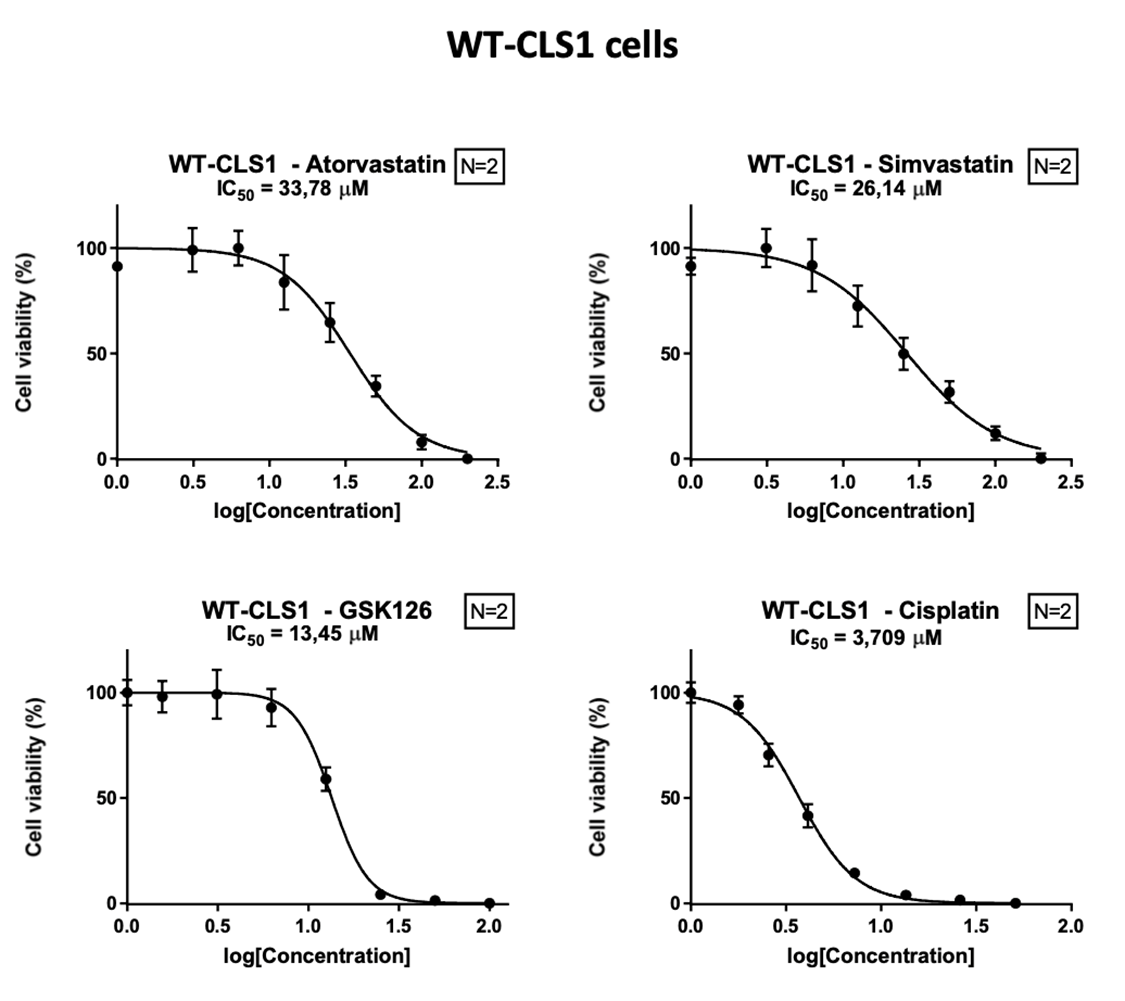


**Supplementary FIG. S48. Effect of drugs on malignant rhabdoid tumor-derived WT-CLS1 cell growth.** Graphs show the percentage of viable WT-CLS1 cells treated with increasing concentrations of atorvastatin (top left), simvastatin (top right), GSK126 (bottom left) or cisplatin (bottom right) (n=2; horizontal bars = means). For each drug, the IC_50_ is shown above the corresponding graph.


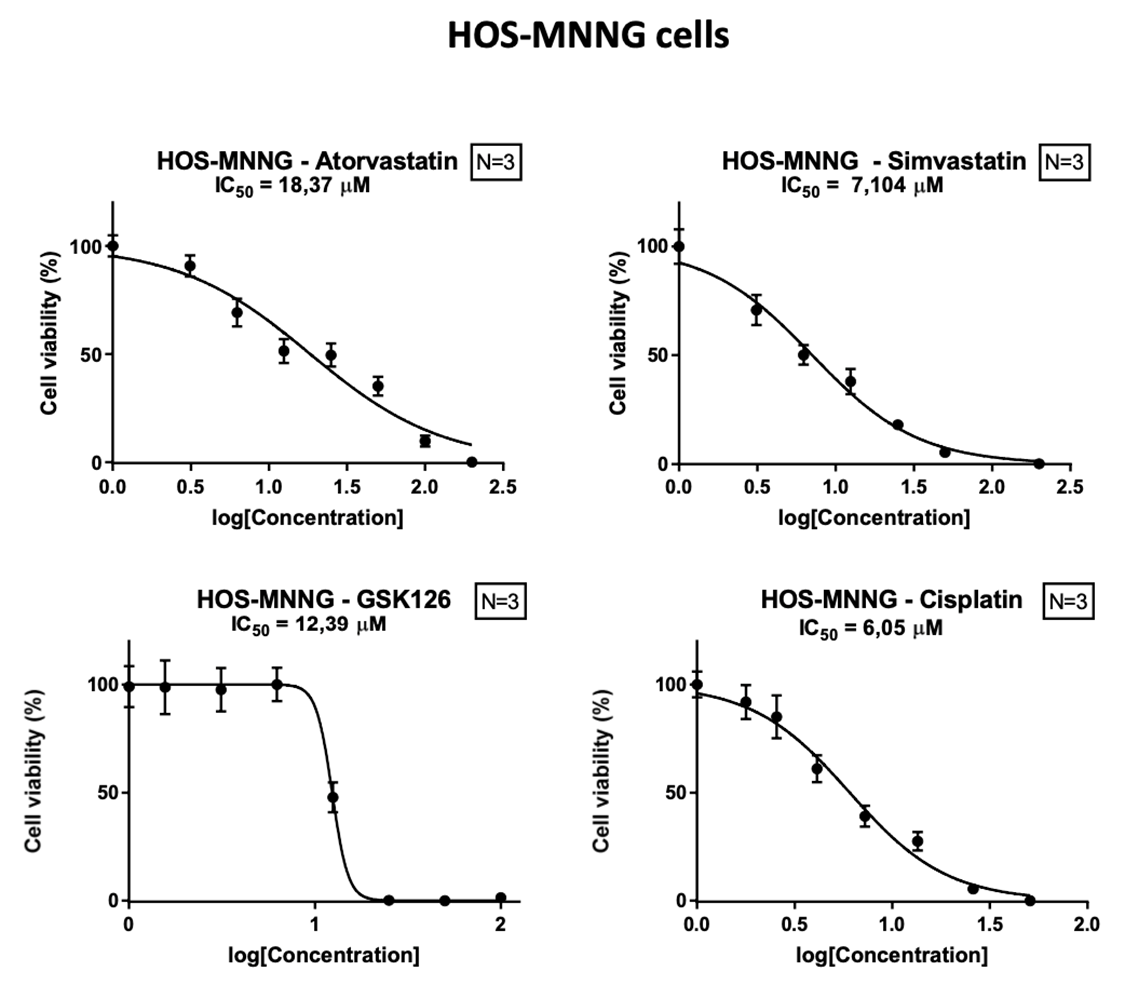


**Supplementary FIG. S49. Effect of drugs on malignant bone tumor-derived HOS-MNNG cell growth.** Graphs show the percentage of viable HOS-MNNG cells treated with increasing concentrations of atorvastatin (top left), simvastatin (top right), GSK126 (bottom left) or cisplatin (bottom right) (n=3; horizontal bars = means). For each drug, the IC_50_ is shown above the corresponding graph.


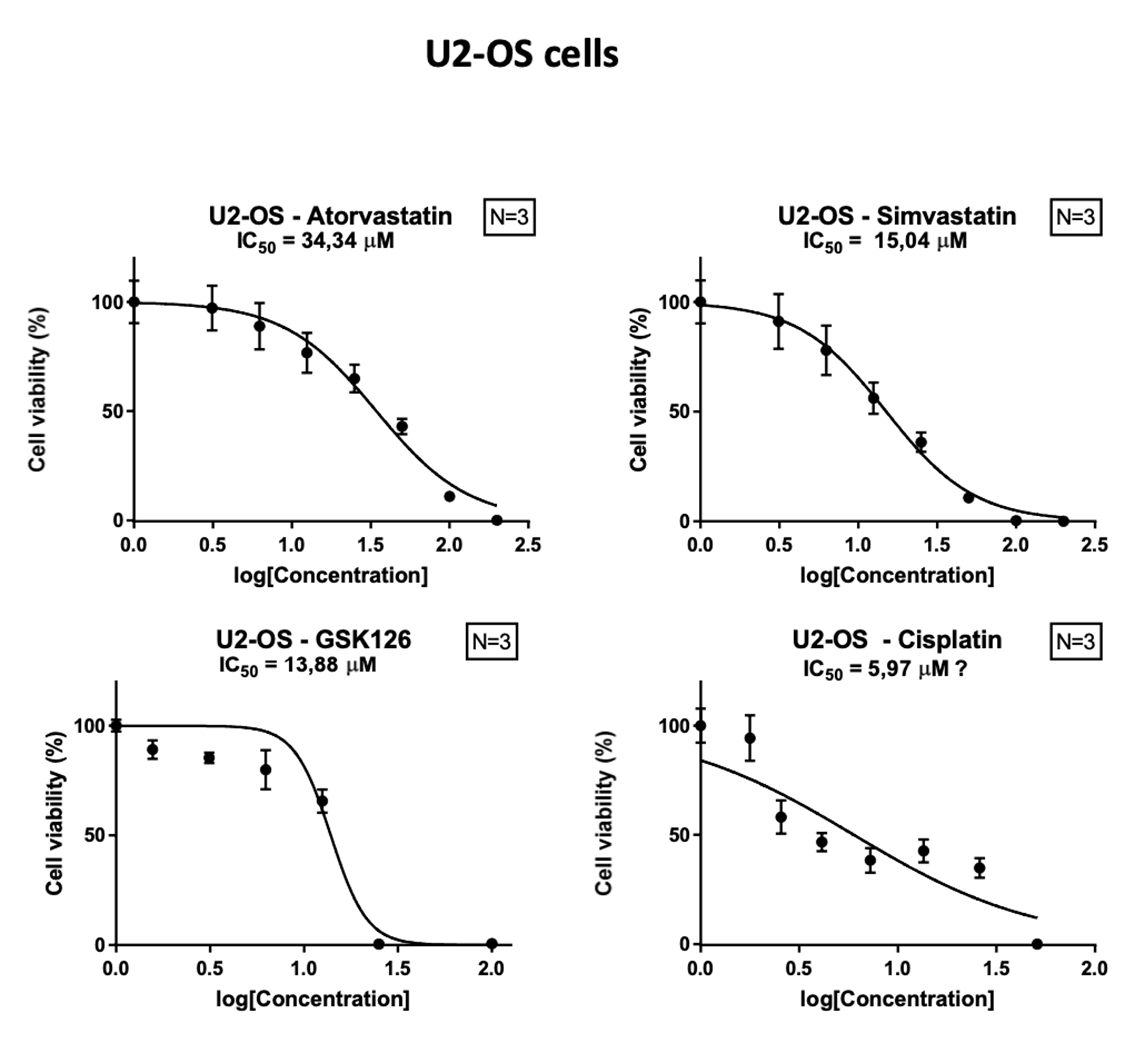


**Supplementary FIG. S50. Effect of drugs on malignant bone tumor-derived U2-OS cell growth.** Graphs show the percentage of viable U2-OS cells treated with increasing concentrations of atorvastatin (top left), simvastatin (top right), GSK126 (bottom left) or cisplatin (bottom right) (n=3; horizontal bars = means). For each drug, the IC_50_ is shown above the corresponding graph.

**
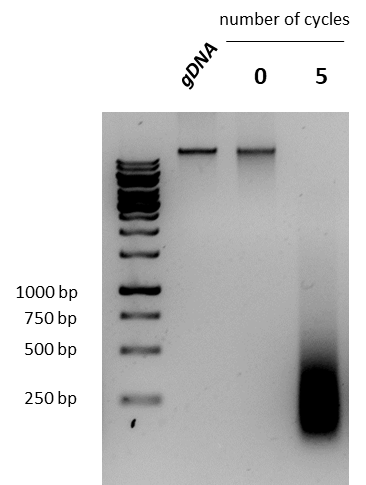
**

**Supplementary FIG. S51. Assessment of genomic DNA fragmentation before ChIP assay.** Following sonication genomic DNA was loaded on a 2% agarose gel along with a 1000 bp ladder. After migration, data showed DNA fragments ranging from 100 to 500 bp.

| **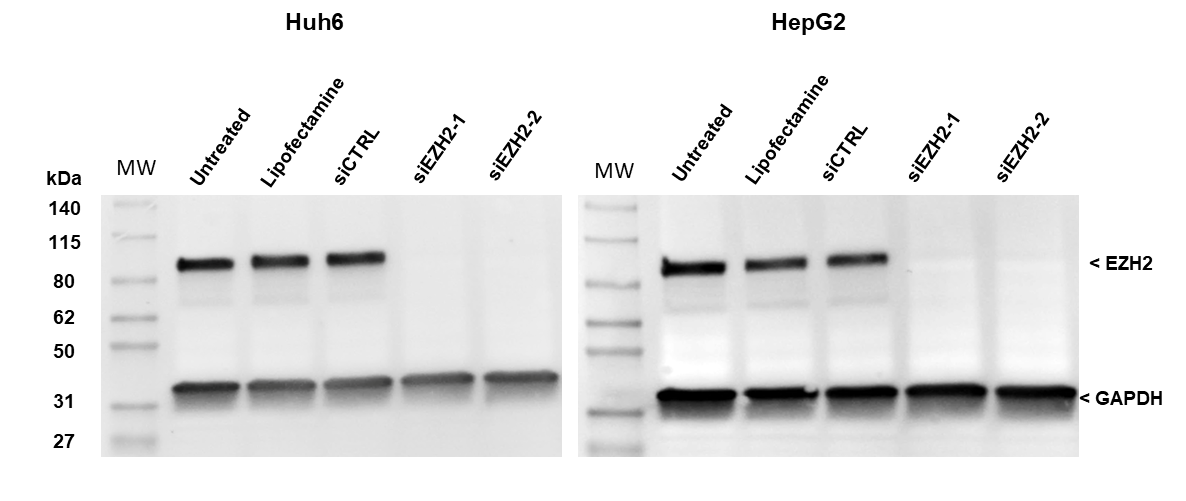** |
| --- |

**Supplementary FIG. S52. Control of EZH2 silencing in HB cells.** Measure of EZH2 (top signals) and GAPDH (bottom signals) levels by western blotting using total proteins extracted from Huh6 (left panels) and HepG2 (right panels) cells untreated, treated with Lipofectamin alone or in presence of siCTRL, siEZH2-1 or siEZH2-2 as indicated. Protein markers are shown on the left (MW: molecular weights). Representative of 3 independent experiments or more.

| **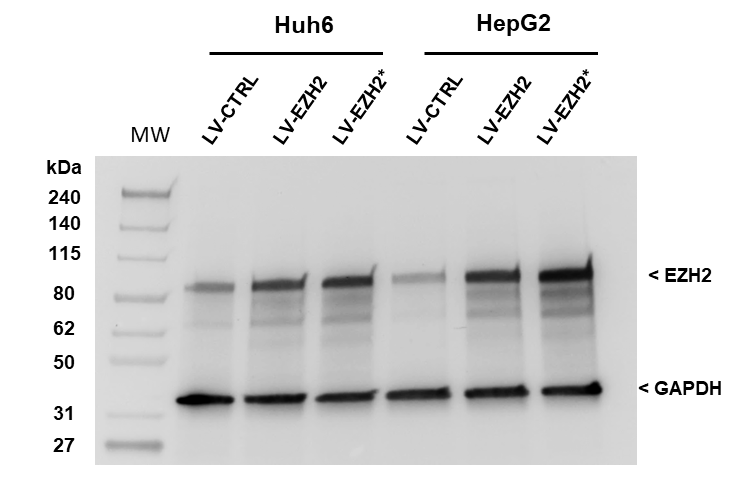** |
| --- |

**Supplementary FIG. S53. Development of CTRL-, EZH2- and EZH2*-expressing HB cell lines.** Measure of EZH2 (top signals) and GAPDH (bottom signals) levels by western blotting using total proteins extracted from Huh6 (left lanes) and HepG2 (right lanes) cells stably expressing the LV-CTRL, LV-EZH2 or LV-EZH2* transgene cassette. Protein markers are shown on the left (MW: molecular weights). Representative of 3 independent experiments or more.

| **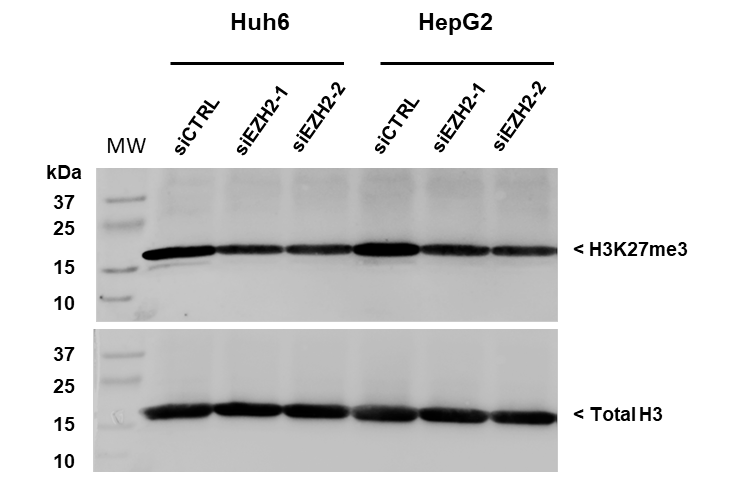** |
| --- |

**Supplementary FIG. S54. EZH2 silencing reduces the trimethylation of histone H3 in HB cells.** Measure of H3K27me3 (top signals) and total histone H3 (bottom signals) levels by western blotting using total proteins extracted from Huh6 (left lanes) and HepG2 (right lanes) cells treated with Lipofectamin in presence of siCTRL, siEZH2-1 or siEZH2-2 as indicated. Protein markers are shown on the left (MW: molecular weights). Representative of 3 independent experiments or more.

| **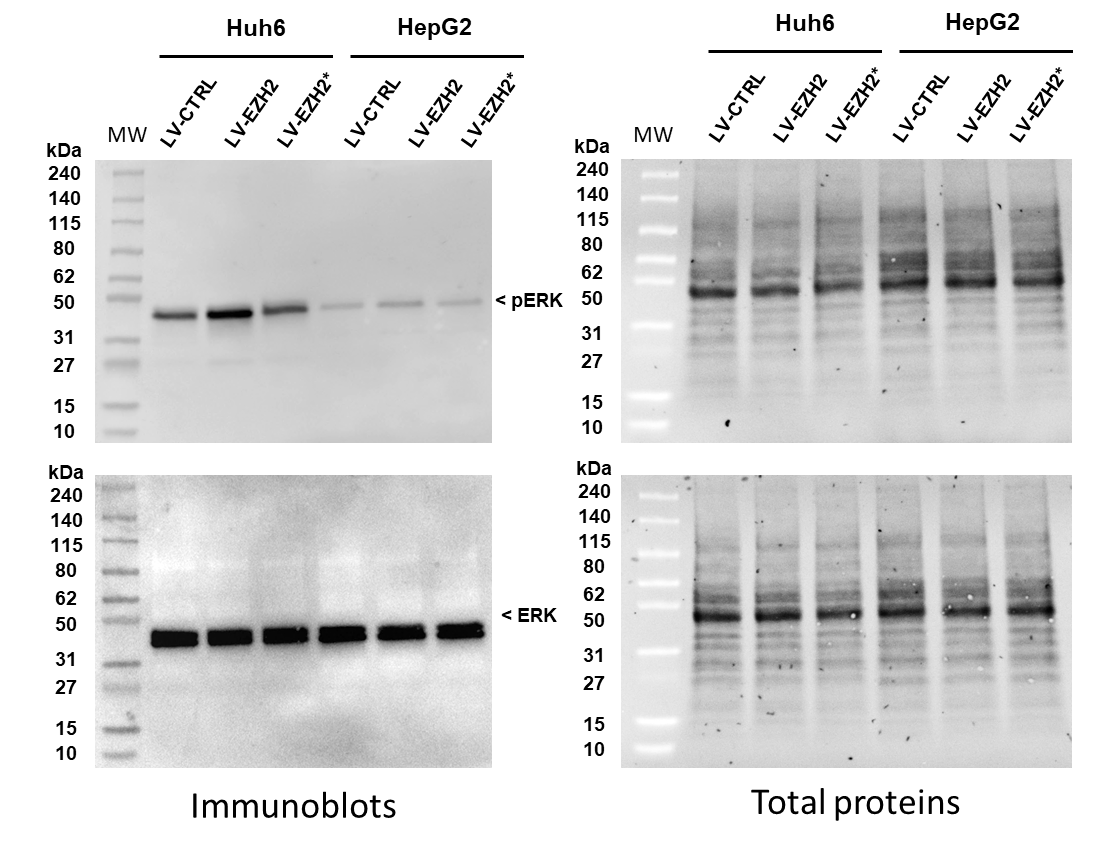** |
| --- |

**Supplementary FIG. S55. The methyltransferase activity of EZH2 potentiates the phosphorylation of ERK in HB cells.** Measure of phospho-ERK (pERK, top signals) and total ERK (bottom signals) levels by western blotting (immunoblots on the left) using total proteins (shown on the right) extracted from Huh6 (left lanes) and HepG2 (right lanes) cells stably expressing the LV-CTRL, LV-EZH2 or LV-EZH2* transgene cassette. Protein markers are shown on the left (MW: molecular weights). Representative of 3 independent experiments or more.

| **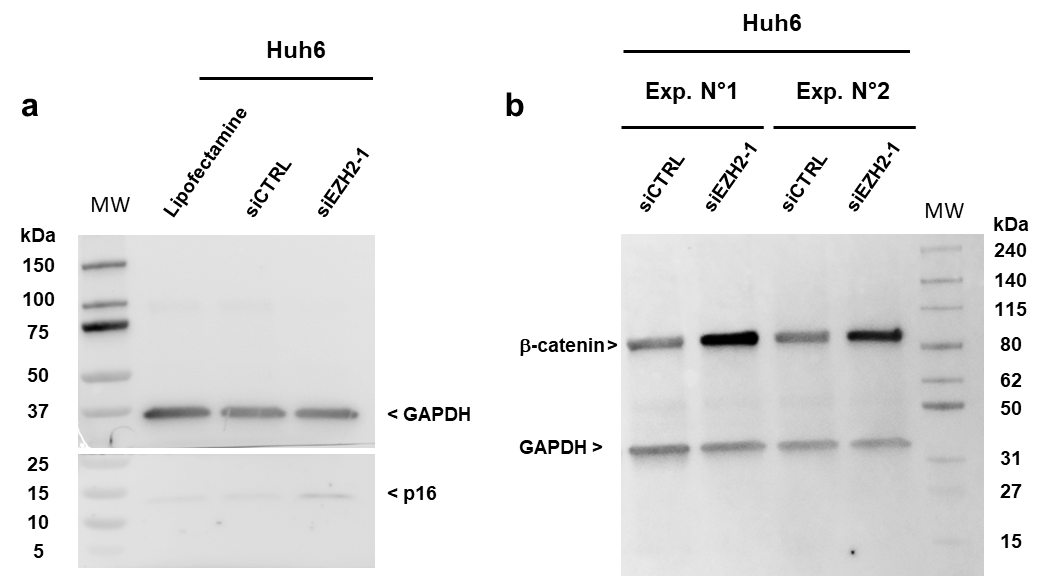** |
| --- |

**Supplementary FIG. S56. The silencing of EZH2 increases the level of p16 and β-catenin proteins in HB cells.** Measure of p16 (**a**, bottom signals) and β-catenin (**b**, top signals, 2 experiments are shown as indicated) levels by western blotting using total proteins extracted from Huh6 cells treated with Lipofectamin alone (**a**) or in presence of siCTRL or siEZH2-1 (**a** and **b**) as indicated. Protein markers are shown on the left or right (MW: molecular weights). GAPDH was used as loading control (**a**, top signals; **b**, bottom signals). Representative of 3 independent experiments or more.

| **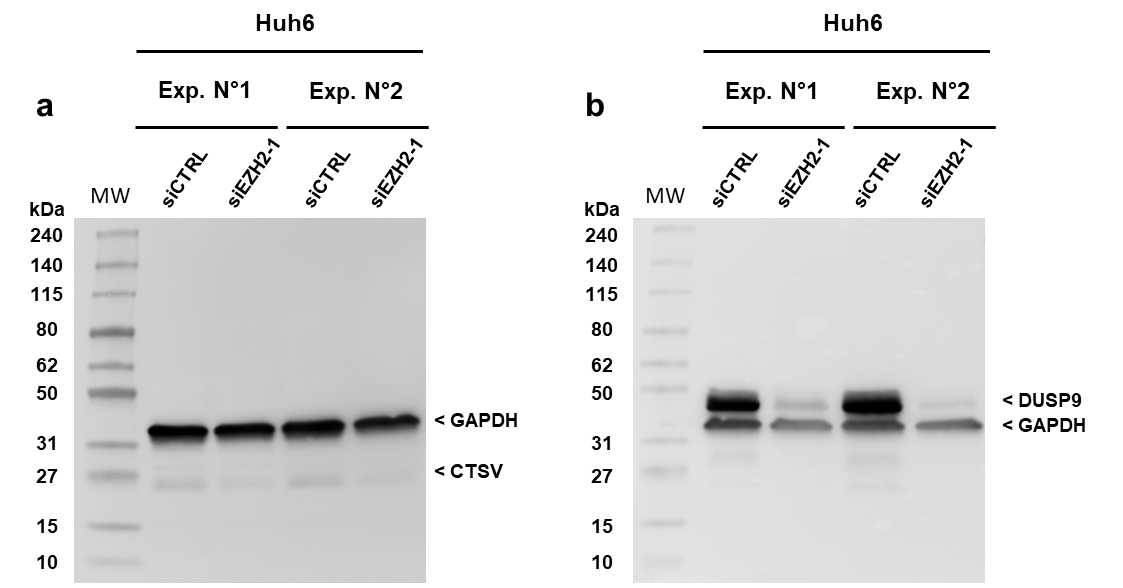** |
| --- |

**Supplementary FIG. S57. The silencing of EZH2 decreases the level of CTSV and DUSP9 proteins in HB cells.** Measure of CTSV (**a**, bottom signals) and DUSP9 (**b**, top signals) levels by western blotting using total proteins extracted from Huh6 cells treated with Lipofectamin in presence of siCTRL or siEZH2-1 as indicated (2 experiments are shown as indicated). Protein markers are shown on the left (MW: molecular weights). GAPDH was used as loading control (**a**, top signals; **b**, bottom signals). Representative of 3 independent experiments or more.

| **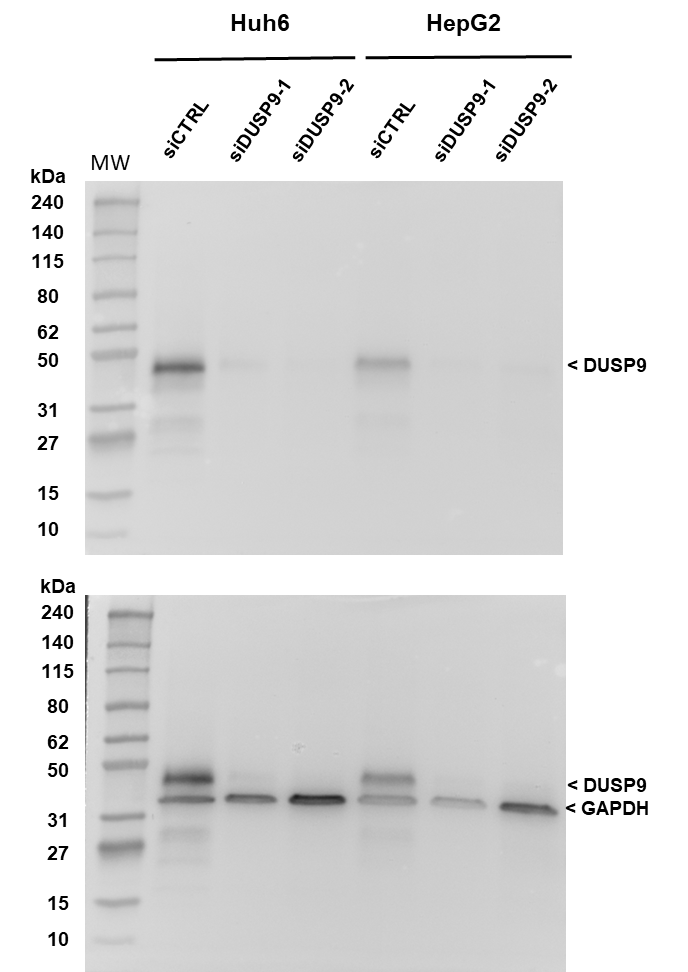** |
| --- |

**Supplementary FIG. S58. Silencing of DUSP9 protein in HB cells.** Measure of DUSP9 (top and bottom signals) and GAPDH (bottom signals) levels by western blotting using total proteins extracted from Huh6 cells treated with Lipofectamin in presence of siCTRL; siDUSP9-1 or si-DUSP9-2 as indicated. Protein markers are shown on the left (MW: molecular weights). Representative of 3 independent experiments or more.

| **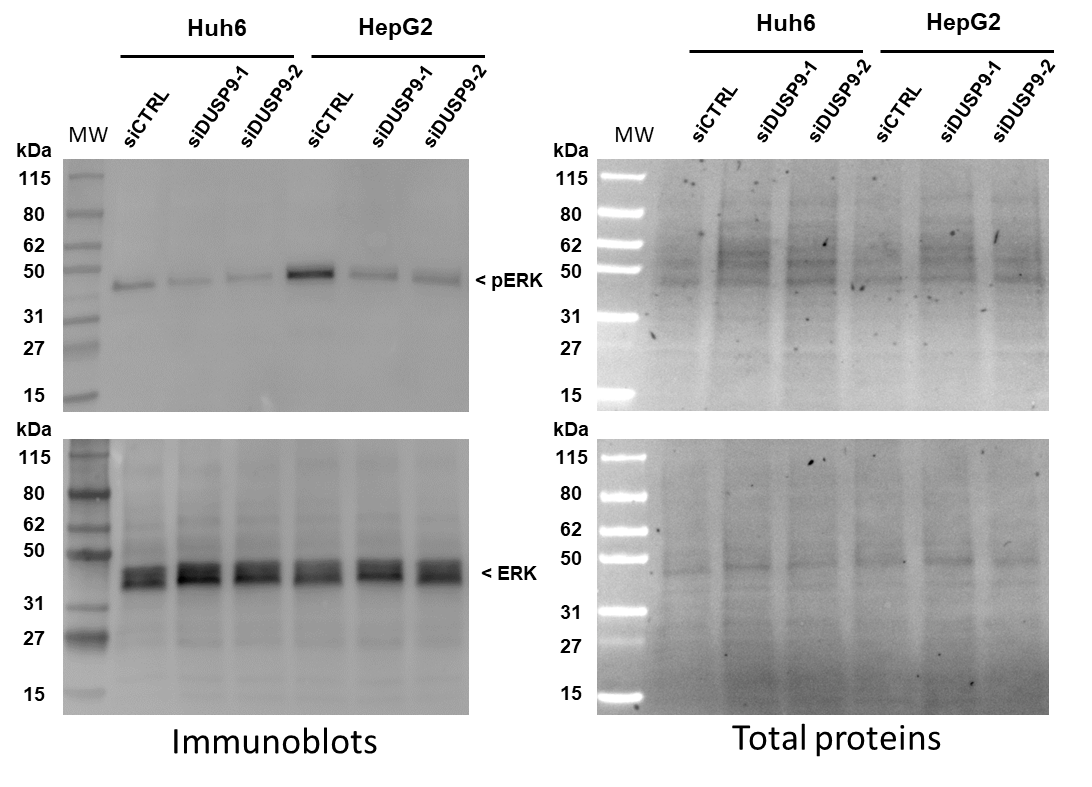** |
| --- |

**Supplementary FIG. S59. The silencing of DUSP9 decreases the level of phosphorylated ERK in HB cells.** Measure of phospho-ERK (p-ERK, top signals) and total ERK (bottom signals) levels by western blotting (immunoblots on the left) using total proteins (shown on the right) extracted from Huh6 (left lanes) and HepG2 (right lanes) cells treated with Lipofectamin in presence of siCTRL; siDUSP9-1 or si-DUSP9-2 as indicated. Protein markers are shown on the left (MW: molecular weights). Representative of 3 independent experiments or more.

| **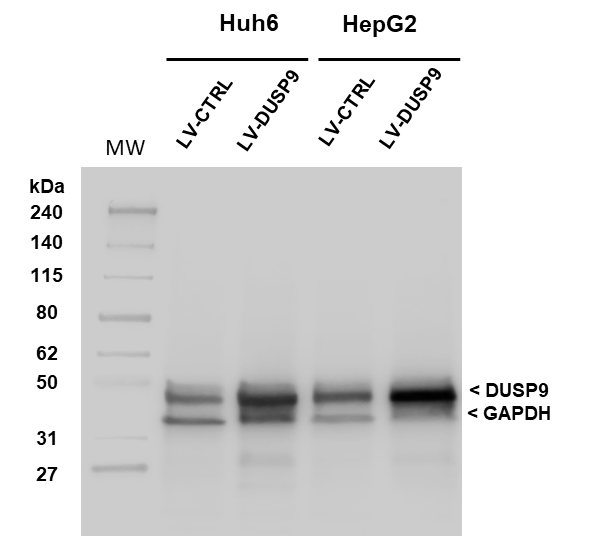** |
| --- |

**Supplementary FIG. S60. Development of HB cells ectopically expressing DUSP9.** Measure of DUSP9 (top signals) and GAPDH (bottom signals) levels by western blotting using total proteins extracted from Huh6 (left lanes) and HepG2 (right lanes) cells expressing the LV-CTRL or LV-DUSP9 cassette as indicated. Protein markers are shown on the left (MW: molecular weights). Representative of 3 independent experiments or more.

| **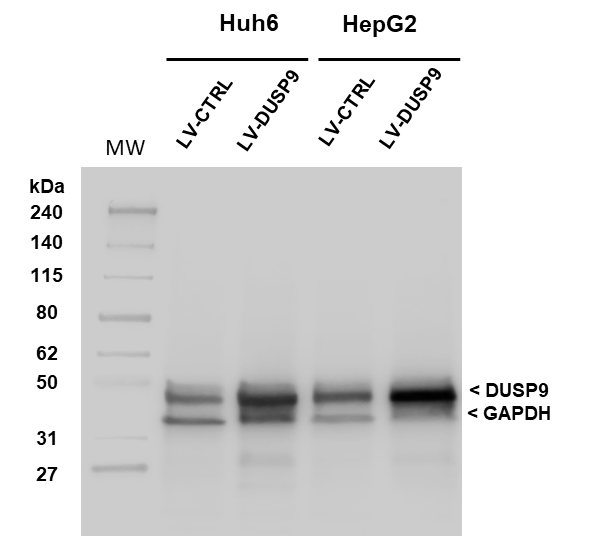** |
| --- |

**Supplementary FIG. S61. Development of HB cells ectopically expressing DUSP9.** Measure of DUSP9 (top signals) and GAPDH (bottom signals) levels by western blotting using total proteins extracted from Huh6 (left lanes) and HepG2 (right lanes) cells expressing the LV-CTRL or LV-DUSP9 cassette as indicated. Protein markers are shown on the left (MW: molecular weights). Representative of 3 independent experiments or more.

| **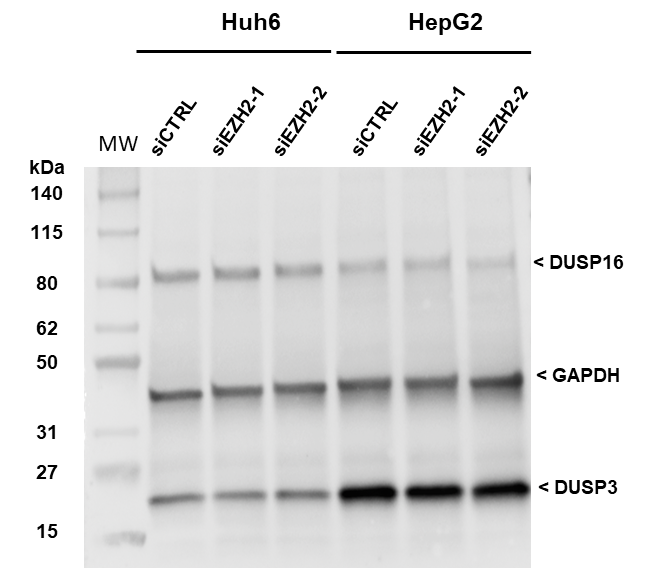** |
| --- |

**Supplementary FIG. S62. EZH2 silencing has not impact on the expression of DUSP3 and DUSP16 in HB cells.** Measure of DUSP16 (top signals), GAPDH (middle signals) and DUSP3 (bottom signals) levels by western blotting using total proteins extracted from Huh6 (left lanes) and HepG2 (right lanes) cells transfected with Lipofectamin in presence of siCTRL, siEZH2-1 or siEZH2-2 as indicated. Protein markers are shown on the left (MW: molecular weights). Representative of 3 independent experiments or more.

| **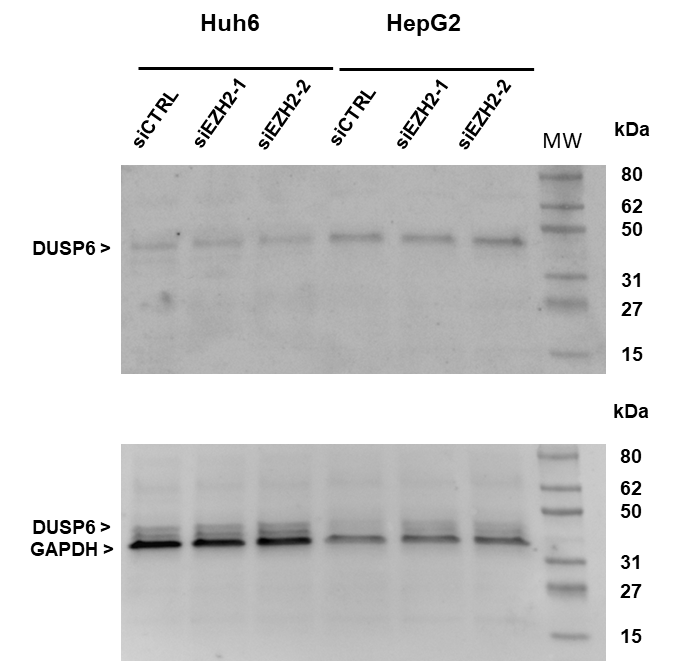** |
| --- |

**Supplementary FIG. S63. EZH2 silencing has not impact on the expression of DUSP6 in HB cells.** Measure of DUSP6 (top signals in top and bottom blots) and GAPDH (bottom signals in bottom blot) levels by western blotting using total proteins extracted from Huh6 (left lanes) and HepG2 (right lanes) cells transfected with Lipofectamin in presence of siCTRL, siEZH2-1 or siEZH2-2 as indicated. Protein markers are shown on the right (MW: molecular weights). Representative of 3 independent experiments or more.

| **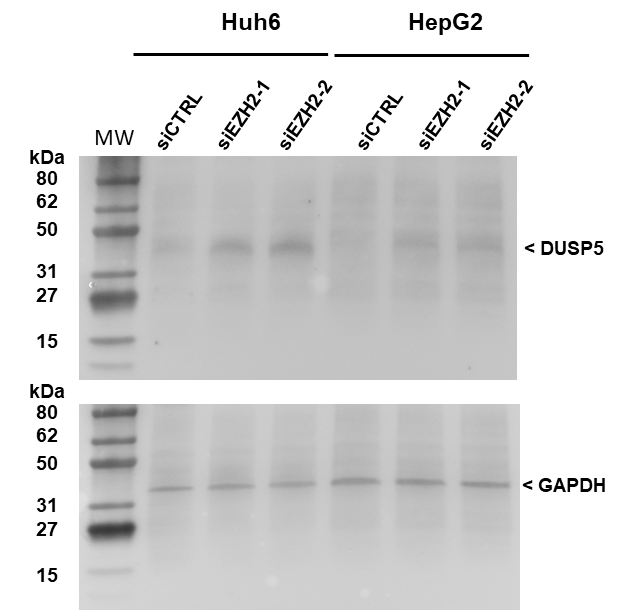** |
| --- |

**Supplementary FIG. S64. EZH2 silencing increased the expression of DUSP5 in HB cells.** Measure of DUSP5 (top signals) and GAPDH (bottom signals) levels by western blotting using total proteins extracted from Huh6 (left lanes) and HepG2 (right lanes) cells transfected with Lipofectamin in presence of siCTRL, siEZH2-1 or siEZH2-2 as indicated. Protein markers are shown on the right (MW: molecular weights). Representative of 3 independent experiments or more.

| **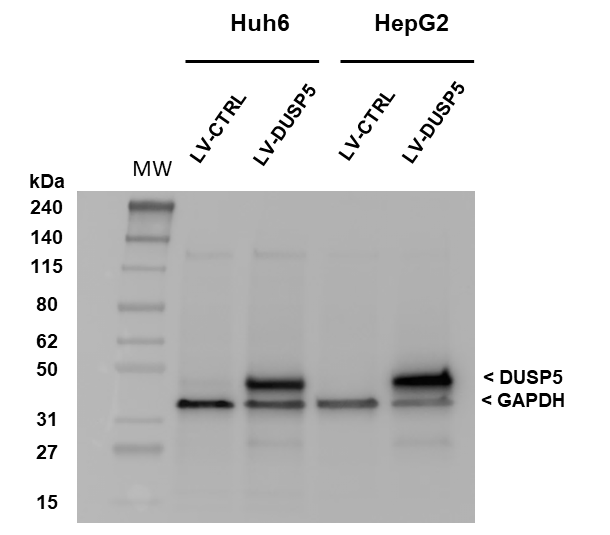** |
| --- |

**Supplementary FIG. S65. Development of HB cells ectopically expressing DUSP5.** Measure of DUSP5 (top signals) and GAPDH (bottom signals) levels by western blotting using total proteins extracted from Huh6 (left lanes) and HepG2 (right lanes) cells expressing the LV-CTRL or LV-DUSP5 cassette as indicated. Protein markers are shown on the left (MW: molecular weights). Representative of 3 independent experiments or more.

| **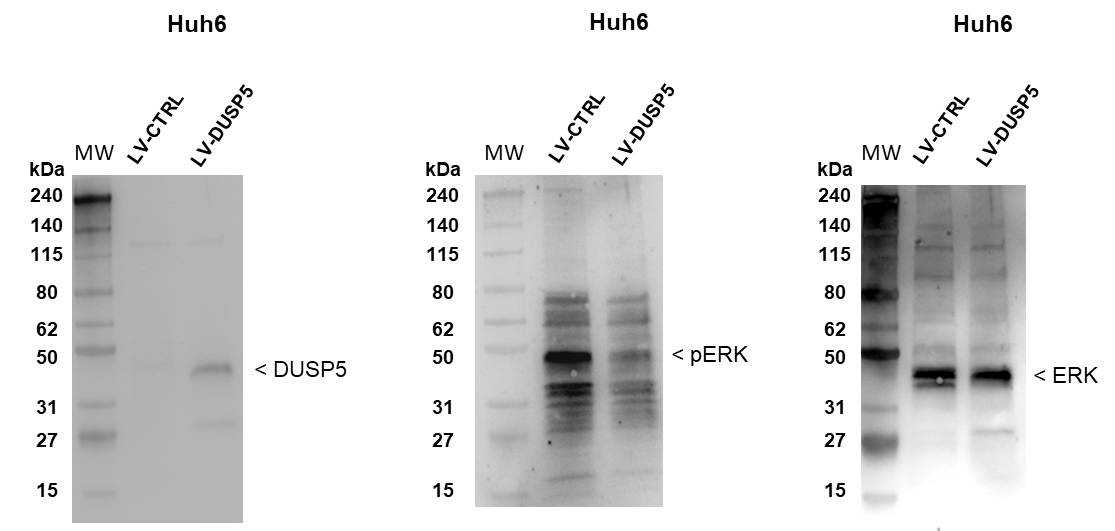** |
| --- |

**Supplementary FIG. S66. The forced expression of DUSP5 decreases the phosphorylation of ERK in HB cells.** Measure of DUSP5 (left signals), phospho-ERK (middle signals) and ERK (right signals) levels by western blotting using total proteins extracted from Huh6 cells expressing the LV-CTRL or LV-DUSP5 cassette as indicated. Protein markers are shown on the left (MW: molecular weights). Representative of 3 independent experiments or more.

| **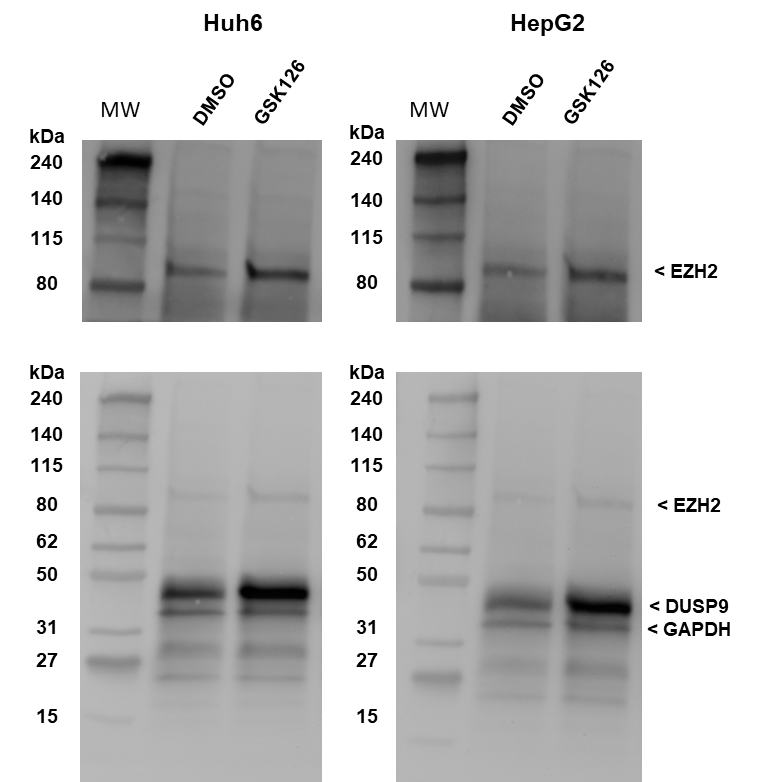** |
| --- |

**Supplementary FIG. S67. Treatment of HB cells by GSK126 increases EZH2 and DUSP9 expressions.** Measure of EZH2 (top blots), DUSP9 (bottom blots, top signals) and GAPDH (bottom blots, bottom signals) levels by western blotting using total proteins extracted from Huh6 (left blots) and HepG2 (right blots) cells treated by DMSO or GSK126 as indicated. Protein markers are shown on the left (MW: molecular weights). Representative of 3 independent experiments or more.

| **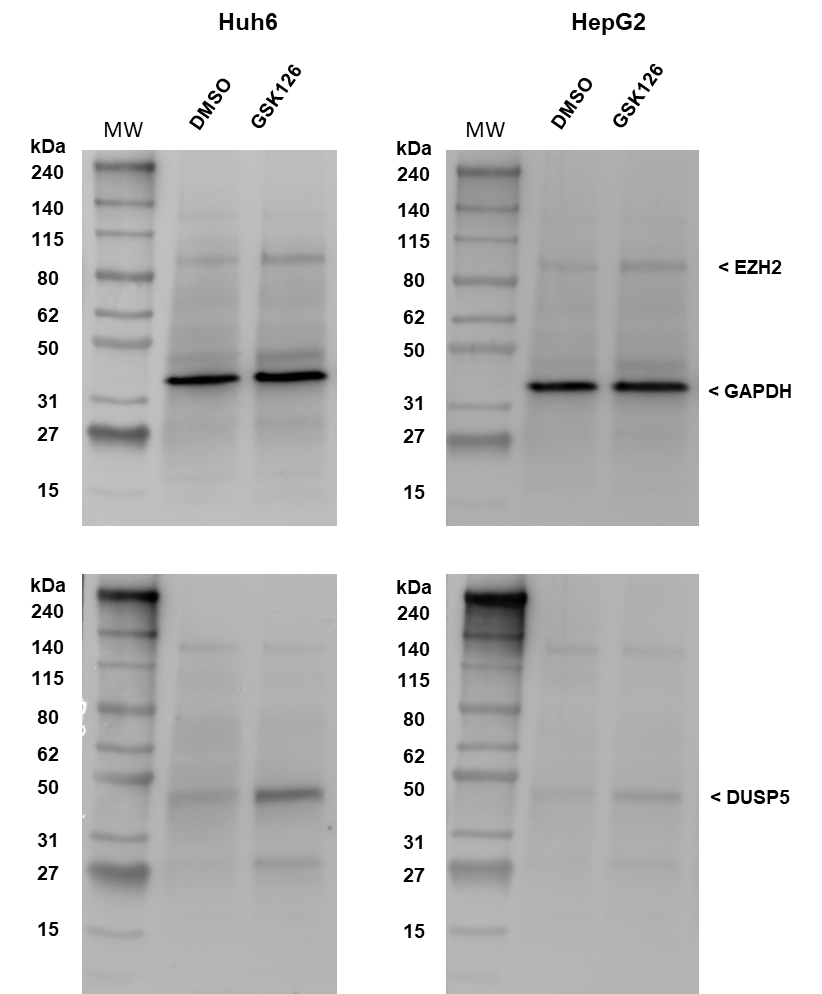** |
| --- |

**Supplementary FIG. S68. Treatment of HB cells by GSK126 increases EZH2 and DUSP5 expressions.** Measure of EZH2 (top blots, top signals), GAPDH (top blot, bottom signals) and DUSP5 (bottom blots) levels by western blotting using total proteins extracted from Huh6 (left blots) and HepG2 (right blots) cells treated by DMSO or GSK126 as indicated. Protein markers are shown on the left (MW: molecular weights). Representative of 3 independent experiments or more.

| **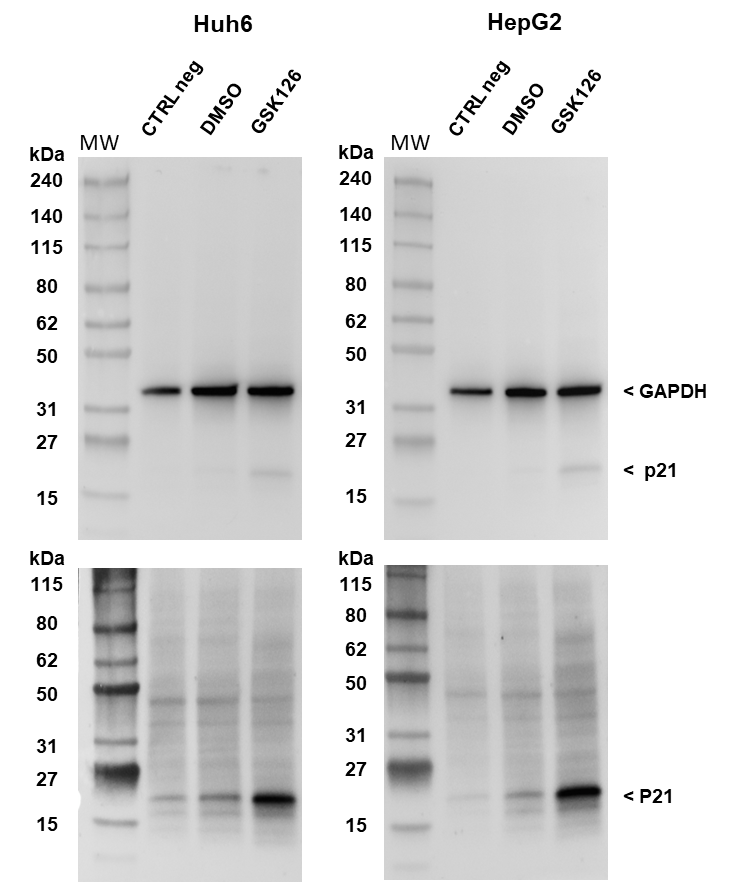** |
| --- |

**Supplementary FIG. S69. Treatment of HB cells by GSK126 increases p21.** Measure of p21 (top blots, bottom signals; bottom blots) and GAPDH (top blot, top signals) levels by western blotting using total proteins extracted from Huh6 (left blots) and HepG2 (right blots) cells untreated (CTRL neg) or treated by DMSO or GSK126 as indicated. Protein markers are shown on the left (MW: molecular weights). Representative of 3 independent experiments or more.

| **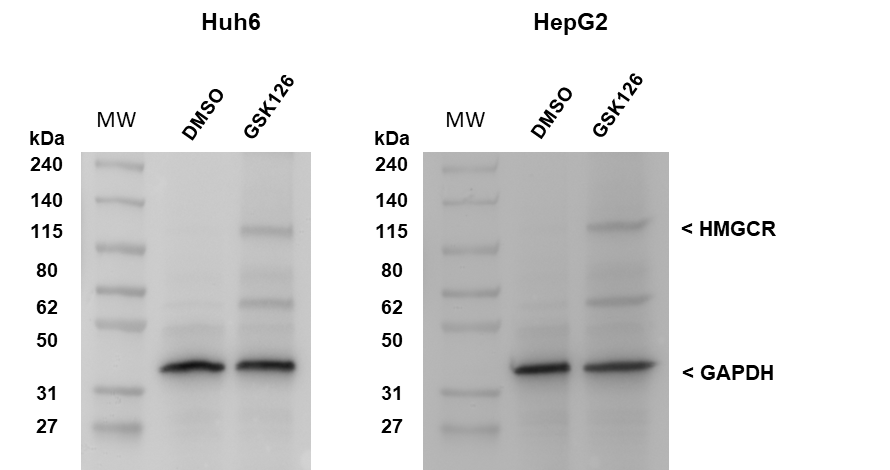** |
| --- |

**Supplementary FIG. S70. Treatment of HB cells by GSK126 increases HMGCR.** Measure of HMGCR (top signals) and GAPDH (bottom signals) levels by western blotting using total proteins extracted from Huh6 (left blots) and HepG2 (right blots) cells treated by DMSO or GSK126 as indicated. Protein markers are shown on the left (MW: molecular weights). Representative of 3 independent experiments or more.

|  |
| --- |

**
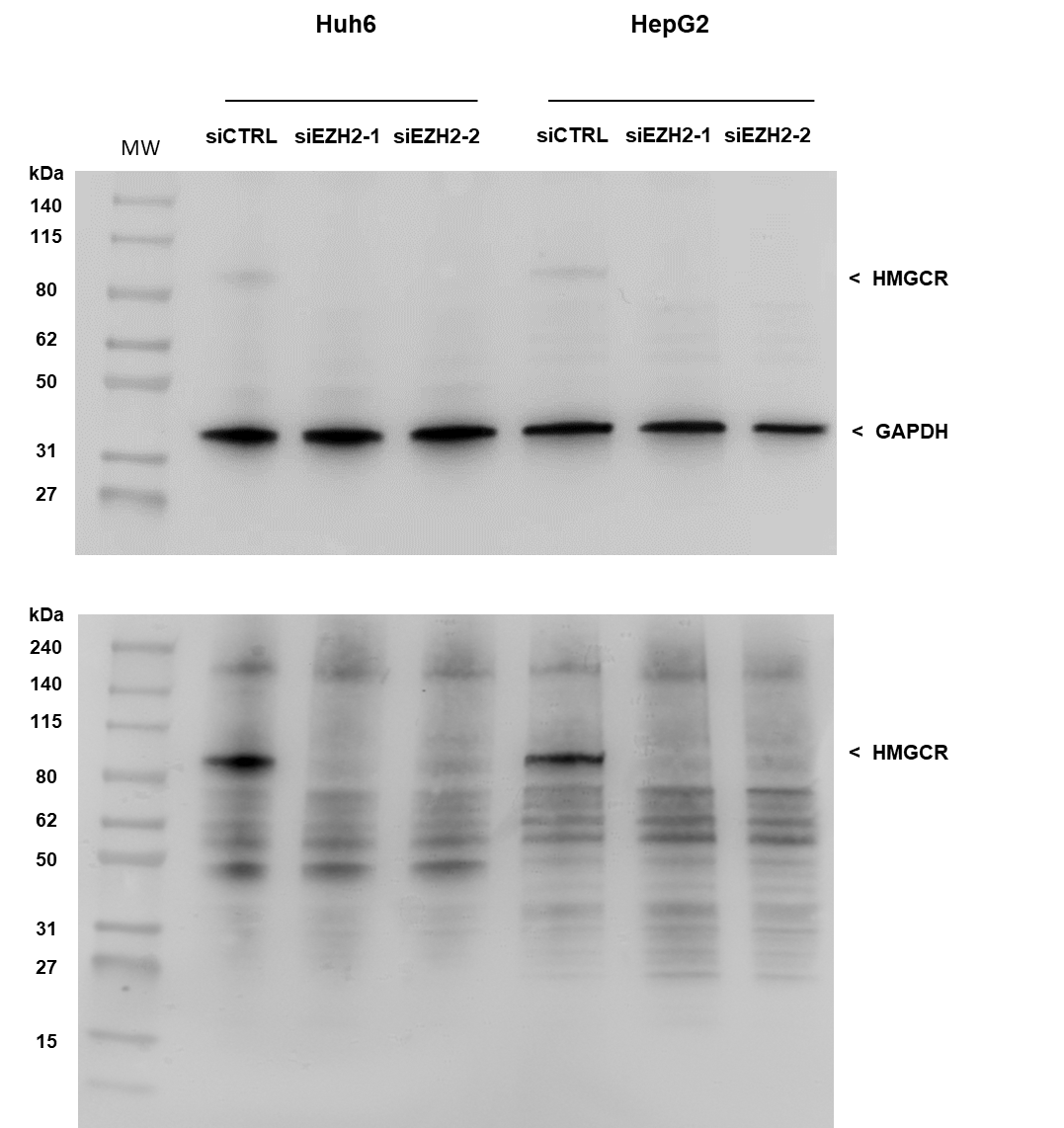
**

**Supplementary FIG. S71. EZH2-silencing induces a decrease of HMGCR in HB cells.** Measure of HMGCR (top signals in top and bottom blots) and GAPDH (top blot, bottom signals) levels by western blotting using total proteins extracted from Huh6 (left panels) and HepG2 (right panels) cells treated with Lipofectamin in presence of siCTRL, siEZH2-1 or siEZH2-2 as indicated. Protein markers are shown on the left (MW: molecular weights). Representative of 3 independent experiments or more.

**References**

[1] Hiyama E. Gene expression profiling in hepatoblastoma cases of the Japanese Study Group for Pediatric Liver Tumors-2 (JPLT-2) trial: Science Repository OU; 2019 2019-02-12.

[2] Carrillo-Reixach J, Torrens L, Simon-Coma M, Royo L, Domingo-Sabat M, Abril-Fornaguera J, et al. Epigenetic footprint enables molecular risk stratification of hepatoblastoma with clinical implications. J Hepatol 2020;73:328-341.

[3] Sumazin P, Chen Y, Trevino LR, Sarabia SF, Hampton OA, Patel K, et al. Genomic analysis of hepatoblastoma identifies distinct molecular and prognostic subgroups. Hepatology 2017;65:104-121.

[4] Valanejad L, Cast A, Wright M, Bissig KD, Karns R, Weirauch MT, et al. PARP1 activation increases expression of modified tumor suppressors and pathways underlying development of aggressive hepatoblastoma. Commun Biol 2018;1:67.

[5] Cairo S, Armengol C, De Reynies A, Wei Y, Thomas E, Renard CA, et al. Hepatic stem-like phenotype and interplay of Wnt/beta-catenin and Myc signaling in aggressive childhood liver cancer. Cancer Cell 2008;14:471-484.

[6] Eichenmuller M, Trippel F, Kreuder M, Beck A, Schwarzmayr T, Haberle B, et al. The genomic landscape of hepatoblastoma and their progenies with HCC-like features. J Hepatol 2014;61:1312-1320.

[7] Hooks KB, Audoux J, Fazli H, Lesjean S, Ernault T, Dugot-Senant N, et al. New insights into diagnosis and therapeutic options for proliferative hepatoblastoma. Hepatology 2018;68:89-102.

[8] Kim J, Lee Y, Lu X, Song B, Fong KW, Cao Q, et al. Polycomb- and Methylation-Independent Roles of EZH2 as a Transcription Activator. Cell Rep 2018;25:2808-2820 e2804.

[9] Maurel M, Jalvy S, Ladeiro Y, Combe C, Vachet L, Sagliocco F, et al. A functional screening identifies five microRNAs controlling glypican-3: role of miR-1271 down-regulation in hepatocellular carcinoma. Hepatology 2013;57:195-204.

[10] Laloo B, Simon D, Veillat V, Lauzel D, Guyonnet-Duperat V, Moreau-Gaudry F, et al. Analysis of post-transcriptional regulations by a functional, integrated, and quantitative method. Mol Cell Proteomics 2009;8:1777-1788.

[11] Shyr ZA, Cheng Y-S, Zheng W. 2.33 - Drug Combinations. In: Kenakin T, editor. Comprehensive Pharmacology. Oxford: Elsevier; 2022. p. 789-812.

[12] Ghousein A, Mosca N, Cartier F, Charpentier J, Dupuy JW, Raymond AA, et al. miR-4510 blocks hepatocellular carcinoma development through RAF1 targeting and RAS/RAF/MEK/ERK signalling inactivation. Liver Int 2020;40:240-251.

[13] Holliday H, Khoury A, Swarbrick A. Chromatin immunoprecipitation of transcription factors and histone modifications in Comma-Dbeta mammary epithelial cells. STAR Protoc 2021;2:100514.

[14] Indersie E, Hooks KB, Capdevielle C, Fabre M, Dugot-Senant N, Desplat A, et al. Tracking cellular and molecular changes in a species-specific manner during experimental tumor progression in vivo. Oncotarget 2018;9:16149-16162.

[15] Indersie E, Lesjean S, Hooks KB, Sagliocco F, Ernault T, Cairo S, et al. MicroRNA therapy inhibits hepatoblastoma growth in vivo by targeting beta-catenin and Wnt signaling. Hepatol Commun 2017;1:168-183.

[16] Gao SB, Xu B, Ding LH, Zheng QL, Zhang L, Zheng QF, et al. The functional and mechanistic relatedness of EZH2 and menin in hepatocellular carcinoma. J Hepatol 2014;61:832-839.

[17] Stelloo S, Nevedomskaya E, Kim Y, Schuurman K, Valle-Encinas E, Lobo J, et al. Integrative epigenetic taxonomy of primary prostate cancer. Nat Commun 2018;9:4900.
