# Supplementary material for: EZH2 is a key prognostic marker and therapeutic target in aggressive and proliferative hepatoblastoma
Source: Mol Cancer. 2026 Feb 23;25:77. doi: 10.1186/s12943-025-02474-9 (PMC13001322; doi:10.1186/s12943-025-02474-9)
Supplement: Supplementary file 2 — Additional file 2. [file 12943_2025_2474_MOESM2_ESM.pdf]

Supplementary Table S1: Datasets and number of patients with *EZH2* transcript level below the median of non-tumoral livers.

Source	Identifier	N° of samples	n° patients (%) with EZH2 > median NT	n° patients (%) with DUSP9 > median NT	n° patients (%) with DUSP5 < median NT	n° patients (%) with HMGCR > median NT	References
Buendia *et al.*	/	29<br>(4 NTL, 25 T)	19 (76%)	25 (100%)	22 (88%)	24 (96%)	[5]
GEO	GSE151347	22<br>(11 NTL, 11 T)	8 (72,72%)	11 (100%)	10 (90,90%)	9 (81,81%)	[6]
GEO	GSE104766	44<br>(22 NTL, 22 T)	16 (72,72%)	21 (95,45%)	18 (81,81%)	12 (54,54%)	[7]
GEO	GSE131329	67<br>(14 NTL, 53 T)	51 (96,22%)	44 (83,01%)	50 (94,33%)	48 (90,56%)	[1]
GEO	GSE133039	66<br>(32 NTL, 31 T, 3R)	26 (76,47%)	27 (79,41%)	31 (94,17%)	22 (64.7%)	[2]
GEO	GSE75271	55<br>(5 NTL, 50 T)	47 (94%)	46 (92%)	48 (96%)	47 (94%)	[3]
GEO	GSE81928	32<br>(3 NTL, 29 T)	26 (89,65%)	27 (93,10%)	24 (82,75%)	16 (55,17%)	[4]

GEO: Gene Expression Omnibus; NT: non-tumoral livers; T: Tumors; R: recurrence.

Supplementary Table S2: Correlative analysis between TOP2A and other genes in HB.

	**Ikeda et al**	**Carillo-Reixach et al**	**Lopez-Terrada et al**	**Karns et al**	**Buendia et al**	**Kappler et al**	**Raymond et al**
**EZH2 vs TOP2A**	R=0.82	R=0.87	R=0.89	R=0.91	R=0.86	R=0.93	R=0.91
	**p<0.0001**	**p<0.0001**	**p<0.0001**	**p<0.0001**	**p<0.0001**	**p<0.0001**	**p<0.0001**
**DUSP9 vs TOP2A**	R=-0.36	R=-0.70	R=-0.66	R=-0.80	R=-0.67	R=-0.84	R=0.60
	**p<0.01**	**p<0.0001**	**p<0.0001**	**p<0.0001**	**p<0.001**	**p<0.001**	**p<0.001**
**DUSP5 vs TOP2A**	R=-0.43	R=-0.65	R=-0.46	R=-0.65	R=-0.42	R=-0.84	R=-0.68
	**p<0.01**	**p<0.0001**	**p<0.001**	**p<0.001**	**p<0.05**	**p<0.001**	**p<0.0001**
**HMGCR vs TOP2A**	R=0.28	R=0.48	R=0.47	R=0.67	R=0.33	R=0.65	R=0.33
	**p<0.05**	**p<0.01**	**p<0.001**	**p<0.001**	p=0.1	**p<0.05**	p=0.07

Supplementary Table S3: Proteins differentialy expressed in *EZH2* -depleted Huh6 cells *versus* CTRL Huh6 cells (Proteomic analysis). Proteins in yellow were selected for validation by Western blot.

Accession	Abundance Ratio: (siEZH2) / (Control)	Abundance Ratio Adj. P-Value: (siEZH2) / (Control)	Log2(abundance Ratio)	Log10(p-Value)
**Q99956 (DUSP9)**	0,129	2,49209E-15	-2,954557029	14,60343628
P13995	0,173	2,49209E-15	-2,531156057	14,60343628
P02458	0,179	2,49209E-15	-2,481968507	14,60343628
Q9NQX3	0,196	8,34183E-13	-2,351074441	12,07873866
Q8WXI9	0,213	2,23776E-06	-2,231074664	5,650186493
Q3ZAQ7	0,215	1,33384E-08	-2,217591435	7,874896263
A0A0R4J2G3	0,216	2,45728E-12	-2,210896782	11,60954535
P14543	0,252	1,72378E-06	-1,988504361	5,763518162
Q8NFQ8	0,263	0,000113601	-1,926865295	3,944617846
P02647	0,278	2,49209E-15	-1,846843212	14,60343628
O75391	0,287	1,25732E-11	-1,800877358	10,90055418
Q8WWH5	0,292	0,000589635	-1,775959726	3,229416745
Q13907	0,299	7,4557E-12	-1,74178261	11,12751158
Q5TBH8	0,311	2,80202E-06	-1,685013515	5,552528769
P22792	0,326	2,76234E-06	-1,61705613	5,558722868
Q5W0V3	0,326	0,000274834	-1,61705613	3,560929541
A0A2R8YFX5	0,333	0,002534513	-1,586405918	2,596105477
Q8NBF2	0,336	1,18258E-05	-1,573466862	4,92716947
P48066	0,349	0,002515631	-1,518701058	2,599353062
Q5SYV1	0,352	0,00657935	-1,506352666	2,18181701
P10155	0,354	6,86194E-06	-1,498178735	5,163553084
Q9BU61	0,359	1,86488E-05	-1,477944251	4,729349109
P50443	0,36	7,53595E-06	-1,473931188	5,122861992
Q15043	0,369	9,50356E-08	-1,438307279	7,022113679
P48723	0,37	0,005684637	-1,434402824	2,245297263
Q9UBK8	0,383	0,000492908	-1,384583703	3,307234133
Q9NQ84	0,383	0,07073519	-1,384583703	1,150364476
P21810	0,385	0,000153967	-1,377069649	3,812572352
A0A3B3IRU6	0,389	0,020811722	-1,36215794	1,681691984
**Q15022 (SUZ12)**	0,389	0,002531707	-1,36215794	2,596586558
P81605	0,392	9,39482E-05	-1,351074441	4,027111536
Q99735	0,4	1,41644E-07	-1,321928095	6,848801817
P01009	0,404	1,27569E-11	-1,307572802	10,89425485
**O60911 (CTSV)**	0,407	0,019202398	-1,2968993	1,716644533
A0A2Y9D025	0,408	0,000198072	-1,293358943	3,703176913
Q9BV57	0,417	0,000542558	-1,261880711	3,265553828
P00846	0,418	9,30935E-06	-1,258425153	5,031080641
P42575	0,423	0,030824664	-1,241270432	1,511101649
Q9NVP2	0,426	0,000782479	-1,231074664	3,106527309
Q9Y3F4	0,428	5,72599E-12	-1,224317298	11,24214941

P04181	0,436	2,29473E-11	-1,19759996	10,63926841
Q99424	0,438	0,041466528	-1,190997225	1,382302327
P08582	0,44	0,001541369	-1,184424571	2,81209338
A0A087WXK2	0,449	0,002932372	-1,15521265	2,532780936
P04114	0,455	0,003375036	-1,13606155	2,47172159
A0A0U1RQV4	0,46	0,039138375	-1,120294234	1,40739721
Q9H9Z2	0,463	1,09026E-09	-1,110915901	8,962469921
O14732	0,464	0,004780426	-1,10780329	2,3205334
Q9Y6M5	0,466	0,026191844	-1,10159814	1,581833925
Q12864	0,469	2,53218E-09	-1,092340172	8,596505426
E7EN19	0,48	0,007245127	-1,058893689	2,139953997
E7EW49	0,48	0,010588994	-1,058893689	1,975145298
M0R205	0,482	0,304723578	-1,052894948	0,516093941
Q8WVY7	0,485	0,001202952	-1,043943348	2,919751701
P61758	0,486	0,000368666	-1,040971781	3,433366913
K7EQG9	0,488	0,193501374	-1,035046947	0,713315947
A0A2R8YD64	0,49	0,031418351	-1,029146346	1,502816613
A0A0J9YXF2	0,496	0,0051915	-1,011587974	2,284707142
Q86XX4	0,499	0,091359712	-1,002888279	1,039245278
Q9NZ08	0,502	0,111575643	-0,994240731	0,952430602
Q8WW52	0,504	0,015310962	-0,988504361	1,814997521
P51812	0,506	7,60175E-05	-0,98279071	4,119086417
Q7Z739	0,509	0,024793822	-0,974262439	1,605656521
G5E948	0,51	0,009872868	-0,971430848	2,005556669
Q9Y281	0,516	0,001625055	-0,954557029	2,789131936
F5GXE4	0,516	0,028645727	-0,954557029	1,542940151
P17931	0,517	6,96921E-07	-0,951763814	6,156816449
P21912	0,521	0,030288096	-0,940644722	1,518728027
Q99626	0,523	0,052751667	-0,935117148	1,277763812
Q99959	0,525	5,27625E-06	-0,929610672	5,277674635
Q9C0D3	0,527	0,00255928	-0,924125133	2,591882197
O00442	0,53	0,016831464	-0,915935735	1,773878107
Q9UFN0	0,531	0,035026419	-0,913216234	1,455604261
Q16831	0,532	0,043201582	-0,910501849	1,364500349
P12277	0,533	3,18265E-06	-0,907792562	5,497211119
Q5SW79	0,533	0,149855324	-0,907792562	0,824327823
Q9HAB8	0,538	0,017438776	-0,894321922	1,758484001
Q15836	0,539	0,056322402	-0,891642822	1,249318832
P00352	0,54	1,60199E-05	-0,888968688	4,795340199
F5H0K0	0,541	0,04684789	-0,886299501	1,329309965
Q6SZW1	0,541	0,105761776	-0,886299501	0,975671265
D4Q8H0	0,542	0,08456199	-0,883635243	1,072824805
Q9BYD2	0,542	0,049389806	-0,883635243	1,30636268
Q9BZF1	0,543	0,022254958	-0,880975897	1,652573221
Q2Q1W2	0,544	0,005140622	-0,878321443	2,288984329
**P04035 (HMGCR)**	0,547	0,006112295	-0,870387262	2,213795693
Q96HR9	0,547	0,010784983	-0,870387262	1,967180535

Q96GX9	0,555	0,083077354	-0,849440323	1,080517344
P20248	0,555	0,195854909	-0,849440323	0,708065539
F5H365	0,565	0,095658605	-0,823677227	1,019275957
P39060	0,565	0,001792618	-0,823677227	2,746512247
Q9H892	0,565	0,24463401	-0,823677227	0,611483166
P31040	0,568	0,00381978	-0,816037165	2,41796165
P30307	0,571	0,606334486	-0,808437349	0,21728773
Q9GZY8	0,576	0,021152666	-0,795859283	1,674634888
S4R347	0,578	0,097991133	-0,790858602	1,008813221
E5RGS4	0,579	0,052185142	-0,788364747	1,28245313
H0Y7Z9	0,58	0,606334486	-0,785875195	0,21728773
P05162	0,582	0,028717376	-0,780908942	1,541855246
Q99470	0,583	0,189208503	-0,778432211	0,72305935
Q9NQ94	0,583	0,287804083	-0,778432211	0,540903049
P34931	0,585	0,122174426	-0,77349147	0,913019693
H3BLV9	0,586	0,033119099	-0,77102743	1,479921487
A0A0G2JMZ8	0,587	0,152826684	-0,768567592	0,81580081
Q99471	0,587	0,18387617	-0,768567592	0,735474551
P15144	0,587	0,535570201	-0,768567592	0,271183595
P02649	0,589	0,000274466	-0,763660461	3,561511447
P52907	0,591	0,001379471	-0,758769964	2,860287425
B7ZKQ9	0,592	0,036438151	-0,756330919	1,438443669
O43347	0,592	0,153402656	-0,756330919	0,814167121
Q01970	0,592	0,21755118	-0,756330919	0,662438557
P26572	0,595	0,180957927	-0,749038426	0,742422388
Q06520	0,596	0,046145726	-0,746615764	1,335868517
Q99715	0,597	0,046096979	-0,744197163	1,336327535
O95081	0,597	0,250350061	-0,744197163	0,601452298
P41235	0,598	0,076291687	-0,74178261	1,117522782
P42167	0,602	0,046467999	-0,732164608	1,332846029
Q7Z4H3	0,602	0,091170803	-0,732164608	1,04014422
O95487	0,602	0,423955057	-0,732164608	0,37268018
A0A087X0S7	0,603	0,524461991	-0,729770093	0,280285981
P31350	0,604	0,045423055	-0,727379545	1,34272366
Q53H96	0,604	0,206424608	-0,727379545	0,685238531
Q9UHV9	0,605	0,0650971	-0,724992953	1,186438358
Q9NPL8	0,61	0,388582914	-0,713118852	0,410516299
E7ESK6	0,611	0,417568022	-0,710755715	0,379272768
Q8N122	0,614	0,507993886	-0,703689439	0,294141515
Q9NWY4	0,615	0,22570847	-0,701341684	0,646452143
O75764	0,616	0,076678923	-0,698997744	1,115323996
P46100	0,617	0,565195738	-0,696657606	0,247801122
Q71RC2	0,618	0,019079119	-0,694321257	1,719441683
P09601	0,618	0,090505744	-0,694321257	1,043323857
Q8NFV4	0,618	0,131633455	-0,694321257	0,88063372
O00767	0,619	0,142112931	-0,691988685	0,847366403
Q5T8P6	0,62	0,224635205	-0,689659879	0,64852218

P17568	0,62	0,252219768	-0,689659879	0,598220878
J3QL71	0,62	0,345572882	-0,689659879	0,461460345
O75874	0,621	0,00196632	-0,687334826	2,706345803
H0YB53	0,621	0,362378107	-0,687334826	0,440838048
P02786	0,622	0,002171913	-0,685013515	2,663157575
P55011	0,624	0,015626037	-0,680382066	1,806151152
Q8IX90	0,624	0,275419289	-0,680382066	0,560005647
E9PF16	0,624	0,267725023	-0,680382066	0,572311035
Q9NVP1	0,628	0,161280773	-0,671163536	0,792417404
P56559	0,63	0,512863053	-0,666576266	0,289998587
Q9NXC5	0,63	0,623488242	-0,666576266	0,205171732
P55060	0,632	0,003600649	-0,662003536	2,443619213
O95816	0,633	0,02806451	-0,659722595	1,551842536
G3V4S9	0,633	0,304723578	-0,659722595	0,516093941
I3L187	0,634	0,341482275	-0,657445255	0,466631834
Q14118	0,637	0,179870826	-0,650634722	0,745039271
A0A1W2PR36	0,638	0,19258744	-0,648371671	0,71537204
Q9UNQ0	0,638	0,172704809	-0,648371671	0,762695569
Q13177	0,641	0,019202398	-0,641603738	1,716644533
P51654	0,641	0,378566109	-0,641603738	0,421858269
P20338	0,642	0,366759981	-0,639354798	0,435618058
Q4G104	0,643	0,606334486	-0,637109357	0,21728773
Q9HD26	0,648	0,354482483	-0,625934282	0,450405221
Q9UMZ2	0,648	0,624190559	-0,625934282	0,204682804
P09525	0,649	0,00821546	-0,623709617	2,085368115
Q86YZ3	0,649	0,425263401	-0,623709617	0,371341992
P52701	0,65	0,008567262	-0,621488377	2,067157952
O60508	0,65	0,451397499	-0,621488377	0,345440851
Q86WX3	0,651	0,631445345	-0,619270551	0,199664234
Q13126	0,652	0,269146447	-0,61705613	0,570011349
P15428	0,655	0,165535348	-0,610433188	0,781109254
O75131	0,655	0,247685672	-0,610433188	0,606099116
Q9H7B2	0,655	0,524461991	-0,610433188	0,280285981
O43237	0,657	0,359008243	-0,606034724	0,44489558
Q04760	0,658	0,014315131	-0,603840511	1,844204673
O75695	0,658	0,180447282	-0,603840511	0,743649655
Q8TCD5	0,658	0,380873442	-0,603840511	0,419219309
Q9H2G2	0,659	0,240555713	-0,60164963	0,618784325
Q15293	0,66	0,015466648	-0,59946207	1,810603798
P15104	0,66	0,19258744	-0,59946207	0,71537204
Q9UNS1	0,66	0,507993886	-0,59946207	0,294141515
Q6NZY4	0,66	0,521579836	-0,59946207	0,282679207
A0A0B4J1V8	0,66	0,660706512	-0,59946207	0,179991413
Q96GS4	0,662	0,668800867	-0,595096878	0,174703173
Q96FC7	0,662	0,421972717	-0,595096878	0,374715628
H3BNX8	0,663	0,199983928	-0,592919225	0,699004906
O60216	0,665	0,383227609	-0,588573754	0,41654321

Q08380	0,666	0,330591571	-0,586405918	0,480708224
H3BNQ7	0,667	0,492479671	-0,584241333	0,307611692
Q96ST3	0,668	0,377123447	-0,582079992	0,423516465
B4DNG0	0,668	0,465699011	-0,582079992	0,331894684
Q9Y2V2	0,669	0,285582861	-0,579921884	0,54426786
Q6P1L8	0,671	0,567270199	-0,575615328	0,246210031
Q06323	0,672	0,088048666	-0,573466862	1,05527722
P34896	0,674	0,099330734	-0,569179503	1,002916355
F8W8I6	0,674	0,606334486	-0,569179503	0,21728773
Q96GX5	0,675	0,674764721	-0,567040593	0,170847632
Q9UIG0	0,675	0,683260797	-0,567040593	0,165413497
P07099	0,676	0,091902001	-0,564904848	1,036675033
F5GWT4	0,677	0,362378107	-0,562772261	0,440838048
E9PE17	0,677	0,535570201	-0,562772261	0,271183595
O00505	0,678	0,094444152	-0,560642822	1,024824929
Q92576	0,678	0,785625184	-0,560642822	0,104784603
O96006	0,678	0,51361169	-0,560642822	0,2893651
Q14257	0,679	0,378402287	-0,55851652	0,422046247
Q9UKG1	0,68	0,558977452	-0,556393349	0,25260571
P00374	0,68	0,175560735	-0,556393349	0,75557261
Q8WUK0	0,681	0,508833548	-0,554273297	0,293424263
A0A0C4DGW6	0,681	0,766992332	-0,554273297	0,115208978
P78310	0,683	0,518618785	-0,550042516	0,285151757
Q02318	0,684	0,408092044	-0,54793177	0,389241872
P03905	0,684	0,606334486	-0,54793177	0,21728773
Q15493	0,685	0,578990962	-0,545824107	0,237328216
Q9H2U2	0,685	0,645084986	-0,545824107	0,190383066
Q15035	0,685	0,606334486	-0,545824107	0,21728773
Q02252	0,687	0,087020646	-0,541617996	1,060377697
O00148	0,688	0,436550505	-0,53951953	0,359965505
O15160	0,689	0,436145912	-0,537424112	0,360368194
P00395	0,689	0,567923402	-0,537424112	0,245710235
Q6NUK1	0,69	0,544676325	-0,535331733	0,263861501
P08590	0,691	0,44926922	-0,533242384	0,347493334
Q9BWH6	0,691	0,606334486	-0,533242384	0,21728773
Q96KB5	0,691	0,524461991	-0,533242384	0,280285981
Q09666	0,692	0,047237379	-0,531156057	1,325714208
P31327	0,692	0,363464403	-0,531156057	0,439538117
H0YM70	0,692	0,318385603	-0,531156057	0,497046579
Q13257	0,692	0,426731257	-0,531156057	0,369845545
Q9BSH4	0,692	0,535570201	-0,531156057	0,271183595
A0A0C4DG02	0,692	0,544676325	-0,531156057	0,263861501
Q08426	0,693	0,246303392	-0,529072743	0,608529607
P13051	0,693	0,606334486	-0,529072743	0,21728773
E5RFU2	0,693	0,606334486	-0,529072743	0,21728773
Q8IUC4	0,694	0,408401443	-0,526992432	0,388912732
I3L2J8	0,695	0,889945084	-0,524915117	0,050636792

Q8IV08	0,695	0,606334486	-0,524915117	0,21728773
Q9UPY3	0,696	0,761093027	-0,522840789	0,118562257
P14373	0,696	0,69590729	-0,522840789	0,157448614
Q9BVG4	0,698	0,164074729	-0,518701058	0,784958304
Q96SZ6	0,698	0,606334486	-0,518701058	0,21728773
O60563	0,698	0,819870143	-0,518701058	0,086254929
O00462	0,699	0,606334486	-0,516635639	0,21728773
O95235	0,7	0,509124601	-0,514573173	0,293175917
P09001	0,701	0,606334486	-0,512513651	0,21728773
Q13572	0,702	0,600706235	-0,510457064	0,22133786
O60488	0,703	0,417752341	-0,508403406	0,379081108
O60341	0,703	0,367051482	-0,508403406	0,435273018
A6NMQ1	0,703	0,582657369	-0,508403406	0,234586757
A0A0C4DG76	0,703	0,766992332	-0,508403406	0,115208978
Q9H9A5	0,704	0,643510684	-0,506352666	0,191444238
A0A0A0MTI1	0,705	0,07573591	-0,504304837	1,120698152
P51003	0,705	0,206960251	-0,504304837	0,684113058
Q96R06	0,705	0,517566333	-0,504304837	0,286033982
Q9BTY7	0,705	0,606334486	-0,504304837	0,21728773
O95104	0,707	0,739475794	-0,50021788	0,131076038
Q9H9B1	0,707	0,823365464	-0,50021788	0,084407353
P00167	0,708	0,084279116	-0,498178735	1,074280028
O94992	0,708	0,507993886	-0,498178735	0,294141515
P22748	0,708	0,423955057	-0,498178735	0,37268018
Q9HCL2	0,708	0,61796081	-0,498178735	0,209039066
Q4G0N4	0,711	0,245738075	-0,492078535	0,609527548
Q15813	0,711	0,431483907	-0,492078535	0,365035397
Q15061	0,712	0,557826041	-0,490050854	0,253501215
K7ESQ2	0,712	0,823365464	-0,490050854	0,084407353
P43304	0,713	0,453875972	-0,488026018	0,343062808
Q68CZ2	0,713	0,548367122	-0,488026018	0,260928592
Q9BVG9	0,713	0,852680663	-0,488026018	0,069213586
A0A384DVK7	0,713	0,773685257	-0,488026018	0,111435679
Q9UKV8	0,714	0,650717945	-0,486004021	0,186607216
H0YDE2	0,715	0,813831176	-0,483984853	0,089465677
Q96DE0	0,715	0,606334486	-0,483984853	0,21728773
P20700	0,716	0,105455215	-0,481968507	0,976931939
Q70UQ0	0,716	0,482124548	-0,481968507	0,316840755
Q9BY89	0,716	0,670318774	-0,481968507	0,173718617
P50225	0,717	0,19944184	-0,479954976	0,700183728
O15164	0,717	0,610733581	-0,479954976	0,2141482
P30085	0,718	0,247925146	-0,477944251	0,605679422
Q9UKL0	0,718	0,666267563	-0,477944251	0,17635133
Q9UEY8	0,719	0,114249336	-0,475936324	0,942146315
O75179	0,719	0,756167518	-0,475936324	0,121381982
Q9NSK0	0,719	0,591374643	-0,475936324	0,228137301
A0A087WYF8	0,719	0,762148929	-0,475936324	0,117960156

Q7Z406	0,72	0,585538736	-0,473931188	0,232444369
P33897	0,72	0,665950567	-0,473931188	0,176558007
P49643	0,721	0,536324266	-0,471928835	0,270572553
Q8IY95	0,721	0,855514423	-0,471928835	0,067772664
A0A087WV24	0,722	0,624190559	-0,469929258	0,204682804
Q7L9L4	0,723	0,606334486	-0,467932448	0,21728773
Q9BYN8	0,723	0,664514249	-0,467932448	0,177495702
Q8N3U4	0,723	0,666267563	-0,467932448	0,17635133
P43246	0,724	0,134690654	-0,465938398	0,870662538
P08133	0,724	0,471146723	-0,465938398	0,326843825
A0A1B0GW23	0,724	0,69590729	-0,465938398	0,157448614
P54725	0,725	0,669966949	-0,4639471	0,173946622
E9PCS8	0,725	0,634837578	-0,4639471	0,197337374
P49406	0,726	0,606334486	-0,461958547	0,21728773
O95707	0,726	0,813831176	-0,461958547	0,089465677
H0Y650	0,726	0,64316584	-0,461958547	0,19167703
Q8IWF2	0,726	0,610213782	-0,461958547	0,214517988
Q9NRG4	0,726	0,810040646	-0,461958547	0,091493189
Q9H9Y6	0,726	0,919602635	-0,461958547	0,036399793
Q13263	0,727	0,146709621	-0,459972731	0,833541405
P19022	0,727	0,302621322	-0,459972731	0,519100476
I3L448	0,727	0,718886374	-0,459972731	0,143339748
O15305	0,728	0,305707349	-0,457989644	0,514694121
P19440	0,729	0,326611876	-0,45600928	0,485968028
A0A1W2PR53	0,729	0,64116843	-0,45600928	0,19302787
O94915	0,729	0,640357929	-0,45600928	0,193577209
Q9P1Y5	0,73	0,709993813	-0,454031631	0,148745436
Q9UJC3	0,73	0,859244314	-0,454031631	0,065883333
Q8IUR7	0,73	0,662976615	-0,454031631	0,17850179
Q96CS3	0,732	0,565195738	-0,450084446	0,247801122
Q9Y547	0,733	0,556394585	-0,448114897	0,254617105
Q9Y613	0,733	0,683750405	-0,448114897	0,165102403
A0A0A0MTL5	0,733	0,723695662	-0,448114897	0,140444031
Q9Y399	0,734	0,544676325	-0,446148032	0,263861501
P50750	0,734	0,68083537	-0,446148032	0,16695789
Q07954	0,735	0,524461991	-0,444183845	0,280285981
O75891	0,735	0,497563406	-0,444183845	0,303151568
Q7L576	0,735	0,612038481	-0,444183845	0,213221271
O43493	0,735	0,695490505	-0,444183845	0,157708795
Q13823	0,736	0,718886374	-0,442222329	0,143339748
Q9UJY4	0,736	0,725618885	-0,442222329	0,139291423
P04920	0,736	0,857696166	-0,442222329	0,066666531
Q709F0	0,737	0,734502401	-0,440263476	0,13400678
Q9H4K7	0,738	0,66798569	-0,438307279	0,175232841
O00499	0,738	0,709993813	-0,438307279	0,148745436
Q9H0T7	0,739	0,731105322	-0,436353731	0,136020055
Q9H9J2	0,741	0,762070805	-0,432454552	0,118004676

P46109	0,741	0,587168196	-0,432454552	0,231237476
Q9BZH6	0,741	0,752195167	-0,432454552	0,123669461
O95551	0,741	0,876901422	-0,432454552	0,057049226
Q9Y3Z3	0,742	0,394153506	-0,430508908	0,404334606
Q99720	0,742	0,634438008	-0,430508908	0,197610807
O00483	0,742	0,7217265	-0,430508908	0,141627348
D6RB89	0,742	0,737151234	-0,430508908	0,132443403
Q8TAE8	0,742	0,917032482	-0,430508908	0,037615281
Q7L266	0,743	0,606334486	-0,428565884	0,21728773
Q15067	0,743	0,68083537	-0,428565884	0,16695789
Q27J81	0,744	0,633928184	-0,426625474	0,197959939
Q9Y2A7	0,745	0,446912496	-0,424687669	0,349777502
A0A0A0MT60	0,745	0,756167518	-0,424687669	0,121381982
C9JG87	0,745	0,576272978	-0,424687669	0,239371744
Q13112	0,745	0,72766436	-0,424687669	0,138068896
Q9HA65	0,745	0,747413415	-0,424687669	0,126439111
P01034	0,747	0,606334486	-0,420819852	0,21728773
H0Y8X6	0,747	0,86458142	-0,420819852	0,063194102
E7EQI7	0,747	0,85777103	-0,420819852	0,066628626
Q9H773	0,748	0,606334486	-0,418889825	0,21728773
P13073	0,749	0,336003067	-0,416962376	0,473656758
A0A2R8Y6L6	0,749	0,758336557	-0,416962376	0,120138008
Q9UBS4	0,75	0,606334486	-0,415037499	0,21728773
Q9Y5K5	0,75	0,660867726	-0,415037499	0,179885457
P40692	0,75	0,796383811	-0,415037499	0,098877577
C9J5N7	0,75	0,762148929	-0,415037499	0,117960156
Q2NL82	0,752	0,468668531	-0,411195433	0,329134206
O00592	0,752	0,362378107	-0,411195433	0,440838048
Q8WVM0	0,752	0,612218264	-0,411195433	0,213093718
F5GY03	0,752	0,778791534	-0,411195433	0,108578778
O14745	0,753	0,288636985	-0,40927823	0,539648021
Q99848	0,753	0,766965493	-0,40927823	0,115224175
H0Y2Q1	0,753	0,763898733	-0,40927823	0,11696421
P52758	0,753	0,758594496	-0,40927823	0,119990313
Q5TDH0	0,754	0,615709713	-0,407363571	0,210623995
O75688	0,754	0,660883395	-0,407363571	0,17987516
P20290	0,755	0,606334486	-0,40545145	0,21728773
P52594	0,755	0,480729835	-0,40545145	0,318098924
Q9BYD1	0,755	0,773685257	-0,40545145	0,111435679
Q9P032	0,755	0,535570201	-0,40545145	0,271183595
Q9Y6M9	0,755	0,763013206	-0,40545145	0,117467945
P0DN79	0,756	0,480951029	-0,40354186	0,317899142
P18085	0,758	0,31792431	-0,399730246	0,497676263
Q9BYC9	0,758	0,819888202	-0,399730246	0,086245363
Q9H7Z6	0,758	0,855819477	-0,399730246	0,067617834
O43676	0,758	0,752280102	-0,399730246	0,123620425
Q8N8S7	0,759	0,633574926	-0,397828209	0,198202018

Q9NRF8	0,759	0,703272039	-0,397828209	0,152876649
J3KQN4	0,759	0,64116843	-0,397828209	0,19302787
Q8IXM3	0,759	0,78357322	-0,397828209	0,105920415
A0A0C4DGL3	0,76	0,659047877	-0,395928676	0,181083035
P18858	0,76	0,656092507	-0,395928676	0,183034922
Q8IWX8	0,76	0,606334486	-0,395928676	0,21728773
O15294	0,76	0,718392509	-0,395928676	0,143638205
Q9BST9	0,76	0,791129771	-0,395928676	0,101752272
Q8NE62	0,761	0,763013206	-0,394031641	0,117467945
O00461	0,761	0,792561103	-0,394031641	0,100967246
Q9Y3B7	0,761	0,889945084	-0,394031641	0,050636792
Q9BX68	0,762	0,641070279	-0,392137097	0,193094357
P28331	0,763	0,4629073	-0,390245038	0,33450597
P41214	0,763	0,781836784	-0,390245038	0,106883901
P21796	0,764	0,35828076	-0,388355457	0,445776513
Q9BTE3	0,764	0,606334486	-0,388355457	0,21728773
Q9NYY8	0,764	0,792561103	-0,388355457	0,100967246
Q8IWA4	0,764	0,859244314	-0,388355457	0,065883333
Q5T4K3	0,764	0,783958713	-0,388355457	0,105706809
Q01780	0,765	0,763013206	-0,386468347	0,117467945
Q96P47	0,765	0,857696166	-0,386468347	0,066666531
Q86SX6	0,765	0,64116843	-0,386468347	0,19302787
Q9HAN9	0,765	0,789806699	-0,386468347	0,102479187
X6R700	0,765	0,795911883	-0,386468347	0,099135011
Q96I15	0,765	0,935144311	-0,386468347	0,029121364
Q13085	0,766	0,535570201	-0,384583703	0,271183595
Q9P2R3	0,766	0,823238104	-0,384583703	0,084474536
Q969X6	0,766	0,965086305	-0,384583703	0,015433847
P35527	0,767	0,378566109	-0,382701517	0,421858269
P49915	0,767	0,381388334	-0,382701517	0,418632595
P50895	0,767	0,664101948	-0,382701517	0,177765246
F5H324	0,767	0,68083537	-0,382701517	0,16695789
A0A087X2D5	0,767	0,829965842	-0,382701517	0,080939781
Q9P270	0,767	0,877985444	-0,382701517	0,056512684
Q9Y5B6	0,768	0,837393418	-0,380821784	0,077070457
A0A3B3ISP1	0,768	0,970944785	-0,380821784	0,012805467
P14854	0,769	0,791129771	-0,378944497	0,101752272
Q96C90	0,77	0,949038704	-0,377069649	0,022716076
G3V5T0	0,77	0,840038112	-0,377069649	0,07570101
O43314	0,77	0,888435404	-0,377069649	0,051374143
P23141	0,771	0,41162733	-0,375197235	0,385495798
P23368	0,771	0,555353294	-0,375197235	0,255430648
Q8WVM8	0,771	0,557089308	-0,375197235	0,254075177
G3V0I5	0,771	0,695147933	-0,375197235	0,157922764
Q7Z417	0,771	0,676632308	-0,375197235	0,169647269
Q9UNN8	0,771	0,864155777	-0,375197235	0,063407962
Q6PI78	0,771	0,813831176	-0,375197235	0,089465677

D6RCB9	0,771	0,915909139	-0,375197235	0,038147608
P04818	0,772	0,568133872	-0,373327247	0,245549317
H0YBL1	0,772	0,805670533	-0,373327247	0,09384252
Q03013	0,773	0,649207511	-0,371459681	0,187616464
Q9GZZ9	0,773	0,843244627	-0,371459681	0,074046417
Q9BWE0	0,773	0,906781701	-0,371459681	0,042497253
P42574	0,773	0,813831176	-0,371459681	0,089465677
Q5JSB5	0,773	0,813831176	-0,371459681	0,089465677
O94925	0,774	0,479409833	-0,369594529	0,319293063
P35251	0,774	0,813831176	-0,369594529	0,089465677
E7EUY6	0,774	0,902383185	-0,369594529	0,044609006
P00367	0,775	0,438282243	-0,367731785	0,358246124
A0A2R8YEK8	0,775	0,791129771	-0,367731785	0,101752272
B7Z3R2	0,775	0,85777103	-0,367731785	0,066628626
Q9UI09	0,776	0,831159928	-0,365871442	0,080315403
Q6IN84	0,776	0,882155787	-0,365871442	0,054454713
H7C1N3	0,776	0,919464224	-0,365871442	0,036465164
Q8NFH3	0,777	0,758336557	-0,364013496	0,120138008
Q9UJQ4	0,778	0,85777103	-0,36215794	0,066628626
A0A087WVZ9	0,778	0,791129771	-0,36215794	0,101752272
Q9NWU5	0,78	0,843020453	-0,358453971	0,074161889
Q12800	0,78	0,843020453	-0,358453971	0,074161889
Q8NI35	0,78	0,976211495	-0,358453971	0,010456083
Q96C01	0,781	0,826264489	-0,356605547	0,082880912
E9PDN5	0,781	0,976211495	-0,356605547	0,010456083
P98164	0,781	0,838715929	-0,356605547	0,076385109
B3KTM8	0,782	0,918554614	-0,354759487	0,036895017
O94888	0,782	0,745527526	-0,354759487	0,127536317
Q9HD33	0,782	0,850959386	-0,354759487	0,070091167
B4DKV7	0,782	0,843020453	-0,354759487	0,074161889
H7BXY3	0,783	0,74224127	-0,352915787	0,129454902
Q96GM5	0,783	0,819615116	-0,352915787	0,086390041
Q96GW9	0,783	0,875544508	-0,352915787	0,057721772
A0A0J9YW33	0,784	0,606334486	-0,351074441	0,21728773
Q9NQ29	0,784	0,864155777	-0,351074441	0,063407962
Q9NRY4	0,784	0,847813729	-0,351074441	0,071699555
Q86XL3	0,784	0,883605373	-0,351074441	0,053741652
P29350	0,784	0,966576772	-0,351074441	0,014763646
P49321	0,785	0,507993886	-0,349235441	0,294141515
Q58FG1	0,785	0,857696166	-0,349235441	0,066666531
Q9HD23	0,785	0,812438319	-0,349235441	0,090209601
Q5R3I4	0,785	0,847463733	-0,349235441	0,071878878
Q9NX02	0,786	0,763013206	-0,347398782	0,117467945
O75175	0,786	0,864155777	-0,347398782	0,063407962
Q9Y2Q9	0,786	0,767120911	-0,347398782	0,115136179
O15084	0,786	0,857696166	-0,347398782	0,066666531
P00403	0,787	0,606334486	-0,345564459	0,21728773

Q8IY81	0,787	0,847463733	-0,345564459	0,071878878
H0Y6I0	0,788	0,866546918	-0,343732465	0,062207918
P49327	0,789	0,535570201	-0,341902795	0,271183595
P51659	0,789	0,535570201	-0,341902795	0,271183595
O43175	0,789	0,532638829	-0,341902795	0,273567177
O43660	0,789	0,87113588	-0,341902795	0,059914098
Q13228	0,789	0,857349286	-0,341902795	0,066842209
Q9UNQ2	0,789	0,857696166	-0,341902795	0,066666531
Q69YN4	0,789	0,911797393	-0,341902795	0,040101654
P49588	0,79	0,54210303	-0,340075442	0,265918165
Q9NR31	0,79	0,606334486	-0,340075442	0,21728773
Q9BYD3	0,79	0,847463733	-0,340075442	0,071878878
Q9H617	0,79	0,843020453	-0,340075442	0,074161889
Q96T60	0,79	0,902069139	-0,340075442	0,044760175
E5RIZ4	0,791	0,85777103	-0,3382504	0,066628626
A8MTY9	0,791	0,838069546	-0,3382504	0,076719941
P31153	0,792	0,589887521	-0,336427665	0,229230791
P54819	0,792	0,581223923	-0,336427665	0,235656519
O75306	0,792	0,659047877	-0,336427665	0,181083035
P00390	0,792	0,762439825	-0,336427665	0,117794427
Q99590	0,792	0,872895766	-0,336427665	0,059037613
Q8TD16	0,792	0,850959386	-0,336427665	0,070091167
F2Z2U4	0,793	0,814339694	-0,334607229	0,089194396
O75489	0,794	0,638457141	-0,332789088	0,194868251
E7EPT4	0,794	0,763013206	-0,332789088	0,117467945
Q15642	0,794	0,810325137	-0,332789088	0,091340689
Q15904	0,794	0,766965493	-0,332789088	0,115224175
P47712	0,794	0,850959386	-0,332789088	0,070091167
Q9H8H0	0,794	0,889603	-0,332789088	0,050803761
O95139	0,794	0,877228668	-0,332789088	0,056887184
Q8NC56	0,794	0,9388065	-0,332789088	0,027423912
Q92968	0,794	0,864155777	-0,332789088	0,063407962
Q16822	0,795	0,634837578	-0,330973234	0,197337374
B4DR80	0,795	0,81309528	-0,330973234	0,08985856
Q16637	0,795	0,865306643	-0,330973234	0,062829962
Q9NRG1	0,795	0,769457205	-0,330973234	0,113815529
O75116	0,795	0,864155777	-0,330973234	0,063407962
Q96P16	0,795	0,864155777	-0,330973234	0,063407962
Q05086	0,795	0,953859691	-0,330973234	0,020515504
H0YCM7	0,795	0,918007555	-0,330973234	0,037153745
Q00688	0,795	0,633574926	-0,330973234	0,198202018
F5H6I7	0,795	0,865619703	-0,330973234	0,062672867
P26583	0,796	0,592775259	-0,329159664	0,227109931
Q8NE01	0,796	0,877228668	-0,329159664	0,056887184
Q8NAV1	0,796	0,919527334	-0,329159664	0,036435356
Q58FF6	0,797	0,643417746	-0,327348371	0,191506965
O43291	0,797	0,813831176	-0,327348371	0,089465677

Q92538	0,798	0,645084986	-0,325539348	0,190383066
Q14258	0,798	0,657596517	-0,325539348	0,182040496
Q9Y6Y8	0,798	0,765872987	-0,325539348	0,115843248
Q9NVN8	0,798	0,904146408	-0,325539348	0,043761239
Q9NVJ2	0,799	0,761093027	-0,323732592	0,118562257
Q8IXB1	0,799	0,851372148	-0,323732592	0,069880562
P42785	0,799	0,847713266	-0,323732592	0,07175102
Q9Y4E8	0,8	0,78498644	-0,321928095	0,105137845
O14787	0,8	0,827687407	-0,321928095	0,082133652
Q9BZK7	0,8	0,813831176	-0,321928095	0,089465677
Q8TC12	0,8	0,790944349	-0,321928095	0,101854072
Q8ND24	0,8	0,895526137	-0,321928095	0,047921734
Q8TED0	0,8	0,870954348	-0,321928095	0,060004608
Q9BWW4	0,8	0,870450752	-0,321928095	0,060255795
B8ZZA8	0,801	0,78820403	-0,320125852	0,103361349
O95218	0,801	0,81309528	-0,320125852	0,08985856
K7EPH3	0,801	0,870450752	-0,320125852	0,060255795
A0A0A0MS70	0,801	0,919602635	-0,320125852	0,036399793
H7BZW1	0,801	0,86516518	-0,320125852	0,062900968
Q14566	0,802	0,606334486	-0,318325858	0,21728773
P23378	0,802	0,606334486	-0,318325858	0,21728773
O75531	0,802	0,80059691	-0,318325858	0,09658609
Q9NPI6	0,802	0,919602635	-0,318325858	0,036399793
P26358	0,803	0,754079617	-0,316528107	0,122582798
Q9GZT3	0,803	0,606334486	-0,316528107	0,21728773
Q9Y6G9	0,803	0,775479754	-0,316528107	0,110429536
Q8NBQ5	0,803	0,794899527	-0,316528107	0,099687761
A0A1B0GTB0	0,803	0,864155777	-0,316528107	0,063407962
Q9NQ50	0,803	0,872209582	-0,316528107	0,059379147
Q9Y4C2	0,804	0,686202957	-0,314732593	0,163547415
Q14683	0,804	0,676632308	-0,314732593	0,169647269
P11766	0,804	0,610213782	-0,314732593	0,214517988
P42345	0,804	0,802620114	-0,314732593	0,095489961
J3KMZ8	0,804	0,945821355	-0,314732593	0,024190885
O95373	0,805	0,606334486	-0,312939312	0,21728773
P45954	0,805	0,839615238	-0,312939312	0,075919688
P08263	0,805	0,762070805	-0,312939312	0,118004676
P78347	0,806	0,606334486	-0,311148256	0,21728773
B1AK88	0,806	0,813831176	-0,311148256	0,089465677
P20020	0,806	0,81309528	-0,311148256	0,08985856
Q07021	0,806	0,606334486	-0,311148256	0,21728773
Q8TCJ2	0,806	0,705316544	-0,311148256	0,151615929
Q86TX2	0,806	0,825436313	-0,311148256	0,083316429
O43929	0,806	0,889945084	-0,311148256	0,050636792
A0A024R442	0,806	0,87465753	-0,311148256	0,058161961
Q9BRP4	0,806	0,917032482	-0,311148256	0,037615281
B3KY94	0,806	0,873120408	-0,311148256	0,058925861

P00338	0,807	0,606334486	-0,309359421	0,21728773
P07355	0,807	0,606334486	-0,309359421	0,21728773
O00625	0,807	0,791129771	-0,309359421	0,101752272
B8ZWD1	0,807	0,813831176	-0,309359421	0,089465677
J3KS15	0,807	0,885081154	-0,309359421	0,053016907
P51970	0,807	0,889945084	-0,309359421	0,050636792
P68371	0,808	0,610213782	-0,307572802	0,214517988
Q13283	0,808	0,610733581	-0,307572802	0,2141482
P22234	0,808	0,60853908	-0,307572802	0,215711526
Q5JWF2	0,808	0,703930733	-0,307572802	0,152470073
B9ZVN9	0,808	0,828294114	-0,307572802	0,081815425
Q5T6V5	0,808	0,823365464	-0,307572802	0,084407353
O95232	0,808	0,761642334	-0,307572802	0,118248925
Q01650	0,808	0,902607717	-0,307572802	0,044500958
A0A494C0R8	0,809	0,863661049	-0,305788392	0,063656667
Q5T4S7	0,809	0,674764721	-0,305788392	0,170847632
P39687	0,809	0,680132877	-0,305788392	0,167406231
O60884	0,809	0,813831176	-0,305788392	0,089465677
F5H8H2	0,809	0,872895766	-0,305788392	0,059037613
Q7Z3C6	0,809	0,88880466	-0,305788392	0,051193677
J3KR97	0,81	0,819888202	-0,304006187	0,086245363
Q5EBL8	0,81	0,902607717	-0,304006187	0,044500958
O43852	0,811	0,634837578	-0,30222618	0,197337374
O00116	0,811	0,650518823	-0,30222618	0,186740132
Q9NZT2	0,811	0,847463733	-0,30222618	0,071878878
Q13308	0,811	0,688256122	-0,30222618	0,162249917
P04183	0,811	0,718886374	-0,30222618	0,143339748
Q8WU90	0,811	0,847463733	-0,30222618	0,071878878
I3L4G0	0,811	0,919464224	-0,30222618	0,036465164
Q86YS3	0,811	0,903381422	-0,30222618	0,044128845
Q14204	0,812	0,634837578	-0,300448367	0,197337374
P46013	0,812	0,831045342	-0,300448367	0,08037528
Q8N5K1	0,812	0,813831176	-0,300448367	0,089465677
Q99856	0,812	0,875570822	-0,300448367	0,05770872
P40818	0,812	0,888435404	-0,300448367	0,051374143
P61978	0,813	0,64567869	-0,298672743	0,189983547
Q9UQE7	0,813	0,681762212	-0,298672743	0,166367074
P60510	0,813	0,843852953	-0,298672743	0,073733225
P61964	0,813	0,933155011	-0,298672743	0,030046207
Q9BXP2	0,813	0,902712393	-0,298672743	0,044450595
O43865	0,814	0,821635909	-0,2968993	0,085320588
Q92769	0,814	0,889009183	-0,2968993	0,051093753
O14497	0,814	0,895526137	-0,2968993	0,047921734
Q9BZE1	0,815	0,937471816	-0,295128036	0,02804178
Q12789	0,815	0,921636345	-0,295128036	0,035440407
Q9Y3U8	0,815	0,715191446	-0,295128036	0,145577689
Q14254	0,815	0,918007555	-0,295128036	0,037153745

Q6YHU6	0,815	0,92780715	-0,295128036	0,032542285
O75323	0,815	0,942082081	-0,295128036	0,025911257
P04424	0,816	0,705258775	-0,293358943	0,151651502
Q8TF05	0,816	0,857696166	-0,293358943	0,066666531
Q00796	0,816	0,845104541	-0,293358943	0,073089565
Q6UXN9	0,816	0,856710214	-0,293358943	0,067166055
P10606	0,816	0,747413415	-0,293358943	0,126439111
Q9GZT8	0,816	0,914210182	-0,293358943	0,038953946
A0A2R8Y4I8	0,816	0,969792804	-0,293358943	0,013321043
Q92667	0,817	0,931911376	-0,291592017	0,030625387
E9PKP7	0,817	0,857696166	-0,291592017	0,066666531
C9JG97	0,817	0,859244314	-0,291592017	0,065883333
Q99832	0,818	0,675411197	-0,289827252	0,170431744
Q14444	0,818	0,719208808	-0,289827252	0,143145003
P09110	0,818	0,756167518	-0,289827252	0,121381982
Q9NVI1	0,818	0,859244314	-0,289827252	0,065883333
A0A087WZ13	0,818	0,918007555	-0,289827252	0,037153745
O95155	0,818	0,864155777	-0,289827252	0,063407962
P56182	0,818	0,944957739	-0,289827252	0,024587614
Q6UX07	0,818	0,980484318	-0,289827252	0,008559348
Q99569	0,818	0,980823302	-0,289827252	0,008409225
P50990	0,819	0,683750405	-0,288064643	0,165102403
Q92922	0,819	0,890608245	-0,288064643	0,050313289
Q9NP72	0,819	0,737681128	-0,288064643	0,132131327
P37198	0,819	0,86516518	-0,288064643	0,062900968
Q9H479	0,819	0,923277657	-0,288064643	0,034667674
C9JXQ9	0,819	0,919602635	-0,288064643	0,036399793
Q5T7U1	0,819	0,875844843	-0,288064643	0,057572823
Q6PL24	0,819	0,924143221	-0,288064643	0,034260718
P53985	0,82	0,731105322	-0,286304185	0,136020055
P84090	0,82	0,753973257	-0,286304185	0,122644058
Q9UH62	0,82	0,926560663	-0,286304185	0,033126142
A0A3B3IRL5	0,82	0,979469477	-0,286304185	0,009009093
P52756	0,82	0,895526137	-0,286304185	0,047921734
P35613	0,821	0,694743921	-0,284545873	0,158175245
Q9UHB6	0,821	0,953001598	-0,284545873	0,020906371
I3L2C7	0,821	0,953001598	-0,284545873	0,020906371
Q8N5N7	0,821	0,953001598	-0,284545873	0,020906371
Q8TCE6	0,821	0,953001598	-0,284545873	0,020906371
Q9C0D9	0,821	0,846484044	-0,284545873	0,072381224
Q14807	0,821	0,968506441	-0,284545873	0,013897487
Q8WWC4	0,821	0,947665183	-0,284545873	0,023345075
P48643	0,822	0,703930733	-0,282789701	0,152470073
Q9UMX0	0,822	0,924143221	-0,282789701	0,034260718
P19525	0,822	0,763013206	-0,282789701	0,117467945
P30086	0,823	0,709993813	-0,281035664	0,148745436
P52888	0,823	0,863678098	-0,281035664	0,063648094

Q14011	0,823	0,872895766	-0,281035664	0,059037613
P28799	0,823	0,926035667	-0,281035664	0,033372286
Q96PU5	0,823	0,921750842	-0,281035664	0,035386457
B4DDK6	0,823	0,932509755	-0,281035664	0,030346616
Q9NYU1	0,823	0,99975746	-0,281035664	0,000105347
P62330	0,824	0,763013206	-0,279283757	0,117467945
A0A0G2JHJ2	0,824	0,932509755	-0,279283757	0,030346616
Q86VP6	0,825	0,721102673	-0,277533976	0,142002895
P33993	0,825	0,718886374	-0,277533976	0,143339748
I3L0H8	0,825	0,773685257	-0,277533976	0,111435679
Q99442	0,825	0,883605373	-0,277533976	0,053741652
Q13148	0,826	0,766965493	-0,275786313	0,115224175
P20339	0,826	0,87148065	-0,275786313	0,059742251
Q13867	0,826	0,785356908	-0,275786313	0,104932932
Q00059	0,827	0,788153261	-0,274040765	0,103389323
Q9BYD6	0,827	0,968506441	-0,274040765	0,013897487
P50579	0,828	0,888435404	-0,272297327	0,051374143
Q9Y383	0,828	0,864155777	-0,272297327	0,063407962
Q8WUA4	0,828	0,917032482	-0,272297327	0,037615281
O95197	0,828	0,781915825	-0,272297327	0,106839997
Q9NQZ5	0,828	0,966576772	-0,272297327	0,014763646
P49736	0,829	0,747318232	-0,270555993	0,126494422
Q13838	0,829	0,782333832	-0,270555993	0,106607888
Q9UKD2	0,829	0,783958713	-0,270555993	0,105706809
Q96I25	0,829	0,813831176	-0,270555993	0,089465677
Q9NXV6	0,829	0,980965309	-0,270555993	0,008346351
Q9UHI6	0,829	0,953453474	-0,270555993	0,020700495
E7EMN6	0,829	0,941555886	-0,270555993	0,026153897
Q8TBC4	0,829	0,969792804	-0,270555993	0,013321043
E9PKU7	0,83	0,944898897	-0,268816758	0,024614658
Q6PJT7	0,83	0,870450752	-0,268816758	0,060255795
C9JYN0	0,83	0,877985444	-0,268816758	0,056512684
Q96I99	0,831	0,790944349	-0,267079618	0,101854072
Q8TDD1	0,831	0,87148065	-0,267079618	0,059742251
Q29RF7	0,832	0,791082358	-0,265344567	0,101778301
Q14534	0,832	0,970616678	-0,265344567	0,012952251
Q9NYV4	0,832	0,98107407	-0,265344567	0,008298203
O95801	0,832	0,975028964	-0,265344567	0,010982483
Q9NUQ3	0,833	0,967942813	-0,263611599	0,0141503
Q9Y6B6	0,833	0,961351224	-0,263611599	0,017117916
Q6UWP2	0,833	0,932855428	-0,263611599	0,030185657
Q9Y3E7	0,833	0,944898897	-0,263611599	0,024614658
Q01581	0,834	0,788153261	-0,261880711	0,103389323
P28288	0,834	0,81309528	-0,261880711	0,08985856
E9PC52	0,834	0,865619703	-0,261880711	0,062672867
O15119	0,834	0,98107407	-0,261880711	0,008298203
Q9H9Q2	0,834	0,965629244	-0,261880711	0,01518959

Q96CT7	0,834	0,94745997	-0,261880711	0,02343913
P25205	0,835	0,768168568	-0,260151897	0,114543467
P16422	0,835	0,766965493	-0,260151897	0,115224175
Q9BY77	0,835	0,890901044	-0,260151897	0,050170532
P03915	0,835	0,971123946	-0,260151897	0,012725337
P60201	0,835	0,895814623	-0,260151897	0,047781853
Q9GZN8	0,836	0,895526137	-0,258425153	0,047921734
Q04323	0,836	0,953001598	-0,258425153	0,020906371
Q16537	0,836	0,975348173	-0,258425153	0,010840325
Q9BWJ5	0,836	0,965062406	-0,258425153	0,015444602
C9JQ41	0,836	0,87148065	-0,258425153	0,059742251
A5YKK6	0,837	0,810040646	-0,256700472	0,091493189
Q01081	0,837	0,875844843	-0,256700472	0,057572823
O14965	0,837	0,897434928	-0,256700472	0,046997032
P46108	0,837	0,944466098	-0,256700472	0,024813627
P40227	0,838	0,788111773	-0,254977851	0,103412185
P23919	0,838	0,797115843	-0,254977851	0,098478559
A0A087WY55	0,838	0,877985444	-0,254977851	0,056512684
Q16795	0,838	0,827982635	-0,254977851	0,081978771
Q9H0U3	0,838	0,831159928	-0,254977851	0,080315403
Q9UGV2	0,839	0,895814623	-0,253257284	0,047781853
E7EMP6	0,839	0,958719392	-0,253257284	0,018308488
Q9BVS5	0,839	0,965306589	-0,253257284	0,015334729
Q5BJH2	0,839	0,978941397	-0,253257284	0,009243306
Q9GZL7	0,839	0,979086916	-0,253257284	0,009178753
Q5UIP0	0,839	0,98107407	-0,253257284	0,008298203
Q5T9A4	0,84	0,848155016	-0,251538767	0,071524765
Q15021	0,84	0,823365464	-0,251538767	0,084407353
Q14498	0,84	0,810325137	-0,251538767	0,091340689
P49642	0,84	0,932855428	-0,251538767	0,030185657
A0A0A0MR74	0,84	0,966576772	-0,251538767	0,014763646
Q15554	0,84	0,99576809	-0,251538767	0,001841795
P17987	0,841	0,805868537	-0,249822294	0,0937358
P07954	0,841	0,802714927	-0,249822294	0,095438661
Q13409	0,841	0,819888202	-0,249822294	0,086245363
Q9H6T3	0,841	0,915703263	-0,249822294	0,038245238
F8W9U3	0,841	0,914210182	-0,249822294	0,038953946
Q9UBV8	0,841	0,902607717	-0,249822294	0,044500958
E9PR44	0,841	0,975028964	-0,249822294	0,010982483
Q9BQC3	0,841	0,974159657	-0,249822294	0,01136986
P04004	0,842	0,840088882	-0,248107862	0,075674763
Q8WW59	0,842	0,953001598	-0,248107862	0,020906371
Q86VS8	0,842	0,970616678	-0,248107862	0,012952251
P09211	0,843	0,813831176	-0,246395464	0,089465677
Q96F86	0,843	0,966576772	-0,246395464	0,014763646
Q96EY8	0,843	0,979212921	-0,246395464	0,009122865
O75157	0,843	0,98107407	-0,246395464	0,008298203

P52732	0,844	0,846484044	-0,244685096	0,072381224
Q9NR30	0,844	0,846484044	-0,244685096	0,072381224
O60784	0,844	0,954250728	-0,244685096	0,0203375
Q9HAD4	0,844	0,975028964	-0,244685096	0,010982483
P24386	0,844	0,98107407	-0,244685096	0,008298203
Q14697	0,845	0,819615116	-0,242976753	0,086390041
Q9UHX1	0,845	0,813786834	-0,242976753	0,089489341
O15042	0,845	0,846484044	-0,242976753	0,072381224
P37268	0,845	0,916387527	-0,242976753	0,037920831
Q96DV4	0,845	0,966576772	-0,242976753	0,014763646
P49458	0,845	0,931717935	-0,242976753	0,030715545
A0A3B3ITS5	0,845	0,969193506	-0,242976753	0,013589504
Q3SXM5	0,845	0,98107407	-0,242976753	0,008298203
Q92499	0,846	0,827332236	-0,241270432	0,082320054
Q9H307	0,846	0,937471816	-0,241270432	0,02804178
Q12872	0,846	0,98107407	-0,241270432	0,008298203
Q93009	0,847	0,827866594	-0,239566125	0,082039642
Q96P11	0,847	0,928238472	-0,239566125	0,032340436
Q9H6R7	0,847	0,979469477	-0,239566125	0,009009093
A0A2U3TZM0	0,848	0,859244314	-0,23786383	0,065883333
Q9Y2U8	0,848	0,970616678	-0,23786383	0,012952251
Q96T88	0,849	0,926719353	-0,236163541	0,033051767
Q9BZE4	0,849	0,927921611	-0,236163541	0,032488711
E7ESY4	0,849	0,967942813	-0,236163541	0,0141503
K7EJE1	0,849	0,953001598	-0,236163541	0,020906371
A0A3F2YNY7	0,849	0,965086305	-0,236163541	0,015433847
Q14790	0,849	0,976570387	-0,236163541	0,010296449
Q9UHD8	0,85	0,846484044	-0,234465254	0,072381224
Q99570	0,85	0,966576772	-0,234465254	0,014763646
P78316	0,85	0,979086916	-0,234465254	0,009178753
Q9NZL9	0,85	0,98107407	-0,234465254	0,008298203
A0A0A0MR02	0,851	0,847463733	-0,232768963	0,071878878
A0A087WXK8	0,851	0,98107407	-0,232768963	0,008298203
H0Y6Y8	0,851	0,952427044	-0,232768963	0,021168281
Q9C0C2	0,851	0,993217131	-0,232768963	0,002955798
Q14966	0,851	0,975732543	-0,232768963	0,01066921
Q96AE4	0,852	0,852676787	-0,231074664	0,06921556
P30041	0,852	0,852680663	-0,231074664	0,069213586
A0A0D9SF70	0,852	0,937948827	-0,231074664	0,027820855
O95299	0,852	0,953859691	-0,231074664	0,020515504
O14880	0,852	0,944882686	-0,231074664	0,024622109
A0A087WYS3	0,852	0,975732543	-0,231074664	0,01066921
Q9H6H4	0,852	0,975732543	-0,231074664	0,01066921
Q9NPF0	0,852	0,970616678	-0,231074664	0,012952251
Q9NTK5	0,853	0,857696166	-0,229382353	0,066666531
Q9Y6A5	0,853	0,865093987	-0,229382353	0,062936707
Q8TEX9	0,853	0,939376074	-0,229382353	0,027160506

Q5VV41	0,853	0,87148065	-0,229382353	0,059742251
O95292	0,853	0,857696166	-0,229382353	0,066666531
Q8IYB3	0,853	0,971417048	-0,229382353	0,012594279
Q96P48	0,853	0,98107407	-0,229382353	0,008298203
A0A087WVM4	0,854	0,933918313	-0,227692025	0,029691109
Q15386	0,854	0,975348173	-0,227692025	0,010840325
Q9HCM4	0,854	0,98107407	-0,227692025	0,008298203
Q9BVW5	0,854	0,98107407	-0,227692025	0,008298203
P54577	0,855	0,859244314	-0,226003675	0,065883333
Q15637	0,855	0,87148065	-0,226003675	0,059742251
Q9GZR7	0,855	0,944898897	-0,226003675	0,024614658
P62879	0,855	0,98107407	-0,226003675	0,008298203
Q12972	0,855	0,953001598	-0,226003675	0,020906371
Q86V21	0,855	0,94057116	-0,226003675	0,026608342
Q9Y316	0,855	0,96473541	-0,226003675	0,015591781
Q15393	0,856	0,862697367	-0,224317298	0,064141527
K7EMM8	0,856	0,944898897	-0,224317298	0,024614658
Q96DI7	0,856	0,952590825	-0,224317298	0,021093606
Q8WWK9	0,856	0,979469477	-0,224317298	0,009009093
P07195	0,857	0,864155777	-0,222632891	0,063407962
O15355	0,857	0,864155777	-0,222632891	0,063407962
Q02818	0,857	0,872895766	-0,222632891	0,059037613
Q92797	0,857	0,872895766	-0,222632891	0,059037613
Q86V81	0,857	0,952590825	-0,222632891	0,021093606
A6NDG6	0,857	0,959941659	-0,222632891	0,017755161
H0YEH2	0,857	0,979469477	-0,222632891	0,009009093
Q9NRZ9	0,858	0,953417031	-0,220950447	0,020717094
P30405	0,858	0,976211495	-0,220950447	0,010456083
Q9BTE6	0,858	0,98262426	-0,220950447	0,007612518
A0A0C4DGQ8	0,858	0,98107407	-0,220950447	0,008298203
J3KT75	0,858	0,962189362	-0,220950447	0,016739449
Q96EH3	0,858	0,99975746	-0,220950447	0,000105347
O75911	0,858	0,98107407	-0,220950447	0,008298203
B0QZ18	0,859	0,866546918	-0,219269964	0,062207918
P67870	0,859	0,959860926	-0,219269964	0,017791687
Q9H3K6	0,859	0,966576772	-0,219269964	0,014763646
F2Z2T0	0,859	0,976922891	-0,219269964	0,010139714
Q9P258	0,86	0,872895766	-0,217591435	0,059037613
Q9BQP7	0,86	0,883161756	-0,217591435	0,053959746
Q14244	0,86	0,965629244	-0,217591435	0,01518959
Q13618	0,86	0,966576772	-0,217591435	0,014763646
A0A0B4J2D5	0,86	0,966576772	-0,217591435	0,014763646
Q5VWZ2	0,86	0,986983696	-0,217591435	0,005690021
E9PKN0	0,86	0,98107407	-0,217591435	0,008298203
P55209	0,861	0,972482109	-0,215914857	0,01211838
P40938	0,861	0,966576772	-0,215914857	0,014763646
P52735	0,861	0,975028964	-0,215914857	0,010982483

Q96ST2	0,861	0,98107407	-0,215914857	0,008298203
G5E975	0,861	0,985132519	-0,215914857	0,006505345
A0A087X0X3	0,862	0,875844843	-0,214240226	0,057572823
P27144	0,862	0,877228668	-0,214240226	0,056887184
Q96KP4	0,862	0,877985444	-0,214240226	0,056512684
Q9Y2W1	0,862	0,895526137	-0,214240226	0,047921734
Q9H9B4	0,862	0,895526137	-0,214240226	0,047921734
Q9NZJ9	0,862	0,883605373	-0,214240226	0,053741652
A0A2R8Y5A6	0,862	0,976922891	-0,214240226	0,010139714
H3BUV4	0,862	0,985132519	-0,214240226	0,006505345
Q9BRX5	0,863	0,98107407	-0,212567535	0,008298203
Q9Y3Q0	0,863	0,98107407	-0,212567535	0,008298203
Q14012	0,863	0,98107407	-0,212567535	0,008298203
B4E3L3	0,863	0,98107407	-0,212567535	0,008298203
P11172	0,864	0,889608762	-0,210896782	0,050800948
Q02978	0,864	0,895526137	-0,210896782	0,047921734
Q9BZZ5	0,864	0,891676267	-0,210896782	0,049792792
Q9UGP8	0,864	0,979414442	-0,210896782	0,009033496
Q5T0D9	0,864	0,965622091	-0,210896782	0,015192807
Q5RKV6	0,864	0,98107407	-0,210896782	0,008298203
Q9Y3D9	0,864	0,898816814	-0,210896782	0,046328812
Q2TAL8	0,864	0,975529197	-0,210896782	0,010759728
O94776	0,864	0,984617738	-0,210896782	0,006732345
P82921	0,864	0,982903546	-0,210896782	0,007489098
Q14240	0,865	0,896310571	-0,209227962	0,047541482
P33992	0,865	0,893390764	-0,209227962	0,048958542
P09874	0,865	0,895526137	-0,209227962	0,047921734
Q8NCW5	0,865	0,967041651	-0,209227962	0,01455482
P55212	0,865	0,977418427	-0,209227962	0,009919478
P05362	0,865	0,985132519	-0,209227962	0,006505345
O00170	0,865	0,987126334	-0,209227962	0,005627262
F8VWW8	0,865	0,988206852	-0,209227962	0,005152139
B8ZZQ6	0,865	0,983972189	-0,209227962	0,007017176
Q9UHG3	0,866	0,911053568	-0,20756107	0,040456087
H0YB11	0,866	0,998739524	-0,20756107	0,000547763
G3V599	0,866	0,997728988	-0,20756107	0,00098741
A0A3B3IT92	0,867	0,906120619	-0,205896101	0,042813987
O75436	0,867	0,902662281	-0,205896101	0,044474705
Q9P0L0	0,867	0,970944785	-0,205896101	0,012805467
Q9NWT6	0,867	0,970616678	-0,205896101	0,012952251
Q9BRQ8	0,867	0,906781701	-0,205896101	0,042497253
A0A2R8Y653	0,867	0,98107407	-0,205896101	0,008298203
P32969	0,868	0,915750086	-0,204233052	0,038223032
O00487	0,868	0,911797393	-0,204233052	0,040101654
P61086	0,868	0,911797393	-0,204233052	0,040101654
Q9BYT8	0,868	0,953001598	-0,204233052	0,020906371
A1L0T0	0,868	0,98107407	-0,204233052	0,008298203

Q969S3	0,868	0,98107407	-0,204233052	0,008298203
Q96PU8	0,868	0,975028964	-0,204233052	0,010982483
P17858	0,869	0,971417048	-0,202571918	0,012594279
A0MZ66	0,869	0,915487461	-0,202571918	0,0383476
Q9HB71	0,869	0,915909139	-0,202571918	0,038147608
P28482	0,869	0,924143221	-0,202571918	0,034260718
Q8NDI1	0,869	0,98107407	-0,202571918	0,008298203
A0A2R8Y855	0,869	0,997663787	-0,202571918	0,001015791
Q15154	0,869	0,997663787	-0,202571918	0,001015791
Q13416	0,869	0,988813095	-0,202571918	0,004885791
Q05655	0,869	0,98107407	-0,202571918	0,008298203
P48728	0,869	0,98107407	-0,202571918	0,008298203
F8VYK3	0,869	0,99576809	-0,202571918	0,001841795
J3KTA4	0,87	0,917032482	-0,200912694	0,037615281
E7EX73	0,87	0,916387527	-0,200912694	0,037920831
P26639	0,87	0,916387527	-0,200912694	0,037920831
P62081	0,87	0,916623473	-0,200912694	0,037809025
I3L1R7	0,87	0,921636345	-0,200912694	0,035440407
Q15738	0,87	0,915548466	-0,200912694	0,038318661
Q86TU7	0,87	0,979469477	-0,200912694	0,009009093
P62277	0,87	0,918554614	-0,200912694	0,036895017
F5H5D3	0,871	0,914210182	-0,199255376	0,038953946
Q14558	0,871	0,98107407	-0,199255376	0,008298203
Q15125	0,871	0,975348173	-0,199255376	0,010840325
Q5F1R6	0,871	0,98107407	-0,199255376	0,008298203
Q92979	0,871	0,98107407	-0,199255376	0,008298203
O75843	0,871	0,99576809	-0,199255376	0,001841795
P11586	0,872	0,921750842	-0,19759996	0,035386457
P78344	0,872	0,923277657	-0,19759996	0,034667674
P0DP25	0,872	0,925286688	-0,19759996	0,033723686
Q9BXS5	0,872	0,919602635	-0,19759996	0,036399793
P46783	0,872	0,923277657	-0,19759996	0,034667674
Q9C0C9	0,872	0,997663787	-0,19759996	0,001015791
P60983	0,872	0,919464224	-0,19759996	0,036465164
P53611	0,872	0,98107407	-0,19759996	0,008298203
Q8N4Q1	0,872	0,98107407	-0,19759996	0,008298203
H0YBZ2	0,872	0,99576809	-0,19759996	0,001841795
P60174	0,873	0,930140724	-0,195946441	0,031451341
P62753	0,873	0,926719353	-0,195946441	0,033051767
O94905	0,873	0,976211495	-0,195946441	0,010456083
Q5VTR2	0,873	0,984617738	-0,195946441	0,006732345
P10909	0,873	0,983972189	-0,195946441	0,007017176
P11142	0,874	0,932509755	-0,194294815	0,030346616
O75390	0,874	0,932509755	-0,194294815	0,030346616
D6RER5	0,874	0,942717719	-0,194294815	0,02561833
Q9UHY7	0,874	0,98107407	-0,194294815	0,008298203
Q9H0A8	0,874	0,99576809	-0,194294815	0,001841795

Q9UNN5	0,874	0,99576809	-0,194294815	0,001841795
P51809	0,874	0,983655849	-0,194294815	0,007156821
P06748	0,875	0,937471816	-0,192645078	0,02804178
A0A0D9SEM4	0,875	0,984617738	-0,192645078	0,006732345
Q6PI48	0,875	0,98107407	-0,192645078	0,008298203
Q9UKA9	0,875	0,98107407	-0,192645078	0,008298203
Q69YN2	0,875	0,98677502	-0,192645078	0,005781853
Q16401	0,875	0,981750145	-0,192645078	0,007999026
P17900	0,875	0,98107407	-0,192645078	0,008298203
P19784	0,875	0,997663787	-0,192645078	0,001015791
Q562R1	0,876	0,942246997	-0,190997225	0,025835238
A0A2R8YFE2	0,876	0,933155011	-0,190997225	0,030046207
Q8NBS9	0,876	0,944466098	-0,190997225	0,024813627
P51398	0,876	0,943354337	-0,190997225	0,02532515
P51649	0,876	0,98107407	-0,190997225	0,008298203
Q96EV2	0,876	0,99576809	-0,190997225	0,001841795
P62253	0,876	0,99975746	-0,190997225	0,000105347
O00217	0,877	0,989353654	-0,189351252	0,004648438
C9J164	0,877	0,99576809	-0,189351252	0,001841795
Q8WZA0	0,877	0,989308676	-0,189351252	0,004668182
Q00765	0,877	0,98107407	-0,189351252	0,008298203
A0A2R8Y761	0,877	0,98107407	-0,189351252	0,008298203
Q9BUH6	0,877	0,99975746	-0,189351252	0,000105347
O75533	0,878	0,950250893	-0,187707155	0,022161714
F8VZX2	0,878	0,950116606	-0,187707155	0,022223091
A0A087X2I1	0,878	0,949920468	-0,187707155	0,022312754
P08574	0,878	0,98107407	-0,187707155	0,008298203
O75027	0,878	0,98677502	-0,187707155	0,005781853
Q9NVV4	0,878	0,98107407	-0,187707155	0,008298203
A0A1W2PQ77	0,878	0,966576772	-0,187707155	0,014763646
Q7Z4H8	0,878	0,984617738	-0,187707155	0,006732345
X6RM59	0,878	0,99975746	-0,187707155	0,000105347
Q16181	0,879	0,953001598	-0,18606493	0,020906371
P12235	0,879	0,977418427	-0,18606493	0,009919478
P31930	0,88	0,953001598	-0,184424571	0,020906371
P25788	0,88	0,958719392	-0,184424571	0,018308488
P37108	0,88	0,950116606	-0,184424571	0,022223091
O60264	0,88	0,986146592	-0,184424571	0,006058522
P04179	0,88	0,98107407	-0,184424571	0,008298203
O95071	0,88	0,997728988	-0,184424571	0,00098741
Q9UBV2	0,88	0,987126334	-0,184424571	0,005627262
P50748	0,88	0,997728988	-0,184424571	0,00098741
F8WCA5	0,88	0,99975746	-0,184424571	0,000105347
Q5H909	0,881	0,953455327	-0,182786076	0,02069965
P39656	0,881	0,959980293	-0,182786076	0,017737682
Q9UBE0	0,881	0,959860926	-0,182786076	0,017791687
B4DLN1	0,881	0,98107407	-0,182786076	0,008298203

Q8NF37	0,881	0,98107407	-0,182786076	0,008298203
Q6NXE6	0,881	0,98107407	-0,182786076	0,008298203
Q96B26	0,881	0,999610105	-0,182786076	0,000169362
D3YTB1	0,881	0,944898897	-0,182786076	0,024614658
Q9NW08	0,881	0,998739524	-0,182786076	0,000547763
Q03701	0,882	0,953001598	-0,181149439	0,020906371
Q5SY16	0,882	0,988206852	-0,181149439	0,005152139
Q8IY37	0,882	0,984617738	-0,181149439	0,006732345
Q8N8A6	0,882	0,994994976	-0,181149439	0,002179112
B4DJV5	0,882	0,99975746	-0,181149439	0,000105347
A0A0A0MTS2	0,883	0,966576772	-0,179514657	0,014763646
Q02878	0,883	0,966576772	-0,179514657	0,014763646
Q99733	0,883	0,965306589	-0,179514657	0,015334729
P14324	0,883	0,964001518	-0,179514657	0,015922282
Q9Y5K6	0,883	0,98107407	-0,179514657	0,008298203
Q8NHQ9	0,883	0,988584312	-0,179514657	0,004986286
O95159	0,883	0,99975746	-0,179514657	0,000105347
O00410	0,884	0,966576772	-0,177881725	0,014763646
P27824	0,884	0,966576772	-0,177881725	0,014763646
P30040	0,884	0,966576772	-0,177881725	0,014763646
Q92820	0,884	0,967041651	-0,177881725	0,01455482
P06730	0,884	0,966576772	-0,177881725	0,014763646
Q8TC07	0,884	0,983972189	-0,177881725	0,007017176
E9PFR3	0,884	0,999610105	-0,177881725	0,000169362
Q12834	0,884	0,989796432	-0,177881725	0,004454116
A0A182DWF2	0,884	0,99975746	-0,177881725	0,000105347
Q15007	0,884	0,99975746	-0,177881725	0,000105347
P43487	0,885	0,969647552	-0,17625064	0,013386095
A0A494C1L5	0,885	0,98107407	-0,17625064	0,008298203
A0A494C1S7	0,885	0,993648498	-0,17625064	0,00276722
Q86W42	0,885	0,99975746	-0,17625064	0,000105347
A0A3B3ITT5	0,885	0,966576772	-0,17625064	0,014763646
Q14CX7	0,885	0,993686847	-0,17625064	0,002750459
Q8NCD3	0,885	0,997663787	-0,17625064	0,001015791
F5H5I6	0,886	0,98107407	-0,174621396	0,008298203
P24928	0,886	0,99576809	-0,174621396	0,001841795
Q13601	0,886	0,98107407	-0,174621396	0,008298203
Q9Y2S7	0,886	0,98107407	-0,174621396	0,008298203
Q96JM3	0,886	0,99975746	-0,174621396	0,000105347
P04406	0,887	0,975028964	-0,17299399	0,010982483
P05455	0,887	0,974159657	-0,17299399	0,01136986
P53597	0,887	0,966576772	-0,17299399	0,014763646
Q9Y3D8	0,887	0,982903546	-0,17299399	0,007489098
O60832	0,887	0,983972189	-0,17299399	0,007017176
Q9NX20	0,887	0,99975746	-0,17299399	0,000105347
Q14692	0,887	0,99975746	-0,17299399	0,000105347
P13807	0,887	0,997529861	-0,17299399	0,001074095

Q92536	0,887	0,99975746	-0,17299399	0,000105347
F8WDS9	0,887	0,99975746	-0,17299399	0,000105347
P78371	0,888	0,975732543	-0,171368418	0,01066921
Q9NPH2	0,888	0,972640591	-0,171368418	0,01204761
P40429	0,888	0,975732543	-0,171368418	0,01066921
Q9Y2X3	0,888	0,988206852	-0,171368418	0,005152139
Q9UKX7	0,888	0,98107407	-0,171368418	0,008298203
Q8NEZ5	0,888	0,983972189	-0,171368418	0,007017176
O96008	0,888	0,985132519	-0,171368418	0,006505345
B7ZC38	0,888	0,997663787	-0,171368418	0,001015791
O14980	0,889	0,976570387	-0,169744676	0,010296449
R4GNH3	0,889	0,976211495	-0,169744676	0,010456083
P07858	0,889	0,975348173	-0,169744676	0,010840325
P82650	0,889	0,983972189	-0,169744676	0,007017176
P49790	0,889	0,99975746	-0,169744676	0,000105347
Q6GMV2	0,889	0,98107407	-0,169744676	0,008298203
Q68E01	0,889	0,99576809	-0,169744676	0,001841795
Q6UWW7	0,889	0,99975746	-0,169744676	0,000105347
Q9Y6D9	0,889	0,99576809	-0,169744676	0,001841795
P50991	0,89	0,979469477	-0,168122759	0,009009093
Q15181	0,89	0,979469477	-0,168122759	0,009009093
P61313	0,89	0,979469477	-0,168122759	0,009009093
H7C2W9	0,89	0,977418427	-0,168122759	0,009919478
J3KT73	0,89	0,968506441	-0,168122759	0,013897487
O95716	0,89	0,998739524	-0,168122759	0,000547763
Q96S94	0,89	0,99975746	-0,168122759	0,000105347
A0A1W2PPA3	0,89	0,983972189	-0,168122759	0,007017176
Q9Y289	0,89	0,99975746	-0,168122759	0,000105347
O60870	0,89	0,989308676	-0,168122759	0,004668182
P35580	0,891	0,98107407	-0,166502663	0,008298203
P49368	0,891	0,980589461	-0,166502663	0,008512779
P60900	0,891	0,979717271	-0,166502663	0,008899236
H0YHA7	0,891	0,98107407	-0,166502663	0,008298203
O95347	0,891	0,971417048	-0,166502663	0,012594279
C9J236	0,891	0,997663787	-0,166502663	0,001015791
Q8IX12	0,891	0,984617738	-0,166502663	0,006732345
P82673	0,891	0,984617738	-0,166502663	0,006732345
Q96DZ1	0,891	0,997728988	-0,166502663	0,00098741
Q15428	0,891	0,99975746	-0,166502663	0,000105347
Q86XP3	0,892	0,971549458	-0,164884385	0,012535086
O00567	0,892	0,976211495	-0,164884385	0,010456083
P08559	0,892	0,975028964	-0,164884385	0,010982483
Q8WXF1	0,892	0,975028964	-0,164884385	0,010982483
P36222	0,892	0,983972189	-0,164884385	0,007017176
Q16740	0,892	0,998739524	-0,164884385	0,000547763
P47813	0,892	0,985132519	-0,164884385	0,006505345
Q9HAU0	0,892	0,99576809	-0,164884385	0,001841795

Q9BV20	0,892	0,99975746	-0,164884385	0,000105347
Q16775	0,892	0,997663787	-0,164884385	0,001015791
I3L397	0,893	0,98107407	-0,16326792	0,008298203
Q15645	0,893	0,975028964	-0,16326792	0,010982483
Q96TA2	0,893	0,991871097	-0,16326792	0,003544765
P43034	0,893	0,989499924	-0,16326792	0,004584235
Q13144	0,893	0,99975746	-0,16326792	0,000105347
P08579	0,893	0,99975746	-0,16326792	0,000105347
Q13614	0,893	0,99975746	-0,16326792	0,000105347
P42704	0,894	0,98107407	-0,161653263	0,008298203
P46779	0,894	0,98107407	-0,161653263	0,008298203
Q13547	0,894	0,991871097	-0,161653263	0,003544765
P55263	0,894	0,976211495	-0,161653263	0,010456083
P53602	0,894	0,99975746	-0,161653263	0,000105347
Q9NQT5	0,894	0,99975746	-0,161653263	0,000105347
A0A0A0MSH9	0,894	0,997663787	-0,161653263	0,001015791
P42166	0,895	0,98107407	-0,160040413	0,008298203
P39023	0,895	0,98107407	-0,160040413	0,008298203
P28838	0,895	0,98107407	-0,160040413	0,008298203
P26373	0,895	0,98107407	-0,160040413	0,008298203
Q92734	0,895	0,977060543	-0,160040413	0,010078525
A0AVT1	0,895	0,987872649	-0,160040413	0,005299039
Q9Y676	0,895	0,99975746	-0,160040413	0,000105347
F6XZQ7	0,895	0,99975746	-0,160040413	0,000105347
Q99808	0,895	0,999014607	-0,160040413	0,000428162
Q9BV23	0,895	0,99975746	-0,160040413	0,000105347
P52292	0,896	0,98107407	-0,158429363	0,008298203
F5GZS6	0,896	0,98107407	-0,158429363	0,008298203
F8WJN3	0,896	0,972482109	-0,158429363	0,01211838
Q9Y3B4	0,896	0,979469477	-0,158429363	0,009009093
G3V5V3	0,896	0,99975746	-0,158429363	0,000105347
A0A0A0MT83	0,896	0,993217131	-0,158429363	0,002955798
Q16658	0,897	0,98107407	-0,15682011	0,008298203
P15880	0,897	0,98107407	-0,15682011	0,008298203
Q12874	0,897	0,98107407	-0,15682011	0,008298203
Q7L1Q6	0,897	0,98107407	-0,15682011	0,008298203
Q7L0Y3	0,897	0,993217131	-0,15682011	0,002955798
Q13247	0,897	0,98107407	-0,15682011	0,008298203
O43747	0,897	0,99576809	-0,15682011	0,001841795
Q15370	0,897	0,98107407	-0,15682011	0,008298203
Q9BTT6	0,897	0,99975746	-0,15682011	0,000105347
H7C3P9	0,897	0,99975746	-0,15682011	0,000105347
P51687	0,897	0,99975746	-0,15682011	0,000105347
Q8WVJ2	0,897	0,99975746	-0,15682011	0,000105347
H3BTP8	0,897	0,99975746	-0,15682011	0,000105347
Q8TBX8	0,897	0,994356943	-0,15682011	0,002457689
O00186	0,897	0,997728988	-0,15682011	0,00098741

O00291	0,897	0,99975746	-0,15682011	0,000105347
P12270	0,898	0,98107407	-0,15521265	0,008298203
Q15459	0,898	0,98107407	-0,15521265	0,008298203
P54727	0,898	0,98107407	-0,15521265	0,008298203
Q14694	0,898	0,98107407	-0,15521265	0,008298203
Q6UN15	0,898	0,993648498	-0,15521265	0,00276722
Q13243	0,898	0,994356943	-0,15521265	0,002457689
Q99575	0,898	0,99975746	-0,15521265	0,000105347
O15091	0,898	0,99975746	-0,15521265	0,000105347
Q8IWV8	0,898	0,995433657	-0,15521265	0,001987679
P04075	0,899	0,98107407	-0,153606979	0,008298203
P31939	0,899	0,98107407	-0,153606979	0,008298203
Q9Y265	0,899	0,98107407	-0,153606979	0,008298203
Q99459	0,899	0,98107407	-0,153606979	0,008298203
P51665	0,899	0,98107407	-0,153606979	0,008298203
Q9Y312	0,899	0,99975746	-0,153606979	0,000105347
Q9Y237	0,899	0,99975746	-0,153606979	0,000105347
Q5SW96	0,899	0,99975746	-0,153606979	0,000105347
Q9UJX4	0,899	0,99975746	-0,153606979	0,000105347
Q9Y3I1	0,899	0,99975746	-0,153606979	0,000105347
Q96S59	0,899	0,99975746	-0,153606979	0,000105347
A0A0C4DGX7	0,899	0,99975746	-0,153606979	0,000105347
P61981	0,9	0,982541782	-0,152003093	0,007648972
Q15691	0,9	0,982029419	-0,152003093	0,007875502
O00232	0,9	0,983532445	-0,152003093	0,007211309
F8VZJ2	0,9	0,983972189	-0,152003093	0,007017176
Q9NR28	0,9	0,98107407	-0,152003093	0,008298203
Q8ND56	0,9	0,99975746	-0,152003093	0,000105347
Q13868	0,9	0,997728988	-0,152003093	0,00098741
Q9BTE7	0,9	0,99975746	-0,152003093	0,000105347
P50583	0,9	0,99975746	-0,152003093	0,000105347
O94826	0,901	0,98107407	-0,150400989	0,008298203
Q9Y5B9	0,901	0,98107407	-0,150400989	0,008298203
Q16186	0,901	0,98107407	-0,150400989	0,008298203
Q08945	0,901	0,98107407	-0,150400989	0,008298203
O43823	0,901	0,99975746	-0,150400989	0,000105347
Q96K76	0,901	0,99975746	-0,150400989	0,000105347
Q9ULX6	0,901	0,997728988	-0,150400989	0,00098741
P15311	0,902	0,984617738	-0,148800661	0,006732345
Q9Y230	0,902	0,984617738	-0,148800661	0,006732345
Q8N2K0	0,902	0,98107407	-0,148800661	0,008298203
O60568	0,902	0,99975746	-0,148800661	0,000105347
D6RIY6	0,902	0,99975746	-0,148800661	0,000105347
P21964	0,902	0,99975746	-0,148800661	0,000105347
O14777	0,902	0,99975746	-0,148800661	0,000105347
Q96HE7	0,903	0,982541782	-0,147202107	0,007648972
Q9NUQ9	0,903	0,98107407	-0,147202107	0,008298203

M0R3D6	0,903	0,986146592	-0,147202107	0,006058522
Q15274	0,903	0,98107407	-0,147202107	0,008298203
Q9Y4P1	0,903	0,99975746	-0,147202107	0,000105347
Q9NXH9	0,903	0,99975746	-0,147202107	0,000105347
Q7Z7H5	0,903	0,99975746	-0,147202107	0,000105347
Q15477	0,903	0,997728988	-0,147202107	0,00098741
P07437	0,904	0,989308676	-0,145605322	0,004668182
E9PLK3	0,904	0,989308676	-0,145605322	0,004668182
P09429	0,904	0,989308676	-0,145605322	0,004668182
P36873	0,904	0,998739524	-0,145605322	0,000547763
P46939	0,904	0,998739524	-0,145605322	0,000547763
B4DKY1	0,904	0,997663787	-0,145605322	0,001015791
Q9BTT0	0,904	0,999610105	-0,145605322	0,000169362
K7EKI8	0,904	0,99975746	-0,145605322	0,000105347
P04844	0,905	0,991871097	-0,144010303	0,003544765
P08865	0,905	0,989308676	-0,144010303	0,004668182
P26368	0,905	0,989308676	-0,144010303	0,004668182
P24534	0,905	0,989353654	-0,144010303	0,004648438
A0A2R8Y7S2	0,905	0,997663787	-0,144010303	0,001015791
P49721	0,905	0,989308676	-0,144010303	0,004668182
P49750	0,905	0,99975746	-0,144010303	0,000105347
P46736	0,905	0,99975746	-0,144010303	0,000105347
Q96I51	0,905	0,99975746	-0,144010303	0,000105347
Q13405	0,905	0,99975746	-0,144010303	0,000105347
P84098	0,906	0,993374613	-0,142417045	0,002886943
Q92688	0,906	0,983972189	-0,142417045	0,007017176
H7BXH2	0,906	0,99576809	-0,142417045	0,001841795
O14776	0,906	0,98107407	-0,142417045	0,008298203
Q3LXA3	0,906	0,99975746	-0,142417045	0,000105347
P62854	0,906	0,993648498	-0,142417045	0,00276722
P30626	0,906	0,98107407	-0,142417045	0,008298203
Q9BVL2	0,906	0,99975746	-0,142417045	0,000105347
P52948	0,907	0,98107407	-0,140825544	0,008298203
Q9UQ35	0,907	0,98107407	-0,140825544	0,008298203
Q9Y2Q3	0,907	0,981573982	-0,140825544	0,008076962
B1AKJ5	0,907	0,98107407	-0,140825544	0,008298203
P83731	0,907	0,994994976	-0,140825544	0,002179112
O75223	0,907	0,98107407	-0,140825544	0,008298203
E7ETY2	0,907	0,998739524	-0,140825544	0,000547763
Q10570	0,907	0,99975746	-0,140825544	0,000105347
Q9BXW9	0,907	0,99975746	-0,140825544	0,000105347
Q9P0Z9	0,907	0,99975746	-0,140825544	0,000105347
O75909	0,907	0,99975746	-0,140825544	0,000105347
P11274	0,907	0,99975746	-0,140825544	0,000105347
Q9Y291	0,907	0,99975746	-0,140825544	0,000105347
Q9Y617	0,908	0,99576809	-0,139235797	0,001841795
Q9UNM6	0,908	0,99576809	-0,139235797	0,001841795

M0R0F0	0,908	0,99576809	-0,139235797	0,001841795
Q9H0S4	0,908	0,99975746	-0,139235797	0,000105347
Q9HCD5	0,908	0,99975746	-0,139235797	0,000105347
Q15056	0,908	0,990867031	-0,139235797	0,003984622
Q08623	0,908	0,99975746	-0,139235797	0,000105347
P16435	0,909	0,99576809	-0,1376478	0,001841795
Q9BUQ8	0,909	0,98107407	-0,1376478	0,008298203
Q9UBQ7	0,909	0,99975746	-0,1376478	0,000105347
Q9NPJ3	0,909	0,99975746	-0,1376478	0,000105347
P82933	0,91	0,993217131	-0,13606155	0,002955798
Q9BQ52	0,91	0,99975746	-0,13606155	0,000105347
Q9H7Z7	0,91	0,99975746	-0,13606155	0,000105347
Q9Y697	0,91	0,99975746	-0,13606155	0,000105347
Q96EY1	0,91	0,99975746	-0,13606155	0,000105347
P24390	0,91	0,99975746	-0,13606155	0,000105347
Q8TAF3	0,91	0,99975746	-0,13606155	0,000105347
P63151	0,91	0,99975746	-0,13606155	0,000105347
Q13136	0,91	0,99975746	-0,13606155	0,000105347
Q7RTV0	0,91	0,99975746	-0,13606155	0,000105347
Q86U42	0,911	0,99975746	-0,134477041	0,000105347
O60256	0,911	0,99975746	-0,134477041	0,000105347
Q9BVP2	0,911	0,99975746	-0,134477041	0,000105347
Q9UKN8	0,911	0,99975746	-0,134477041	0,000105347
Q8TB61	0,911	0,99975746	-0,134477041	0,000105347
Q5BJD5	0,911	0,99975746	-0,134477041	0,000105347
Q9UBQ5	0,911	0,99975746	-0,134477041	0,000105347
P34897	0,912	0,997728988	-0,13289427	0,00098741
Q9H4A4	0,912	0,99576809	-0,13289427	0,001841795
O94906	0,912	0,988813095	-0,13289427	0,004885791
Q8TEM1	0,912	0,992702761	-0,13289427	0,00318077
P08243	0,912	0,984617738	-0,13289427	0,006732345
Q9NS69	0,912	0,999610105	-0,13289427	0,000169362
P25490	0,912	0,99975746	-0,13289427	0,000105347
Q8NHH9	0,912	0,99975746	-0,13289427	0,000105347
Q9H0R4	0,912	0,99975746	-0,13289427	0,000105347
E9PGT6	0,912	0,99975746	-0,13289427	0,000105347
Q5SRE5	0,912	0,99975746	-0,13289427	0,000105347
Q86UE8	0,912	0,99975746	-0,13289427	0,000105347
P67809	0,913	0,99975746	-0,131313235	0,000105347
P23396	0,913	0,998739524	-0,131313235	0,000547763
Q9BXP5	0,913	0,99975746	-0,131313235	0,000105347
K7EQJ5	0,913	0,993217131	-0,131313235	0,002955798
O95359	0,913	0,99975746	-0,131313235	0,000105347
C9JIZ6	0,913	0,99975746	-0,131313235	0,000105347
Q9P0V9	0,913	0,99975746	-0,131313235	0,000105347
B7WP74	0,913	0,99975746	-0,131313235	0,000105347
Q06587	0,913	0,99975746	-0,131313235	0,000105347

O75934	0,913	0,99975746	-0,131313235	0,000105347
Q99496	0,913	0,99975746	-0,131313235	0,000105347
P10809	0,914	0,99975746	-0,12973393	0,000105347
A0A024R4E5	0,914	0,99975746	-0,12973393	0,000105347
O75694	0,914	0,994356943	-0,12973393	0,002457689
P49792	0,914	0,993217131	-0,12973393	0,002955798
O95573	0,914	0,987848359	-0,12973393	0,005309717
Q13616	0,914	0,993648498	-0,12973393	0,00276722
Q12959	0,914	0,99975746	-0,12973393	0,000105347
Q13185	0,914	0,994927333	-0,12973393	0,002208638
Q9Y4W2	0,914	0,99975746	-0,12973393	0,000105347
Q92530	0,914	0,99975746	-0,12973393	0,000105347
Q00577	0,914	0,99975746	-0,12973393	0,000105347
Q2TB90	0,914	0,99975746	-0,12973393	0,000105347
J3KN16	0,915	0,993217131	-0,128156351	0,002955798
Q5JR04	0,915	0,99975746	-0,128156351	0,000105347
P35250	0,915	0,99576809	-0,128156351	0,001841795
P40937	0,915	0,99576809	-0,128156351	0,001841795
Q9H4L4	0,915	0,99975746	-0,128156351	0,000105347
Q53EP0	0,915	0,99975746	-0,128156351	0,000105347
Q9H1B7	0,915	0,99975746	-0,128156351	0,000105347
P09661	0,916	0,99576809	-0,126580497	0,001841795
H0Y5J4	0,916	0,99975746	-0,126580497	0,000105347
Q9UHY1	0,916	0,993374613	-0,126580497	0,002886943
P62266	0,916	0,997728988	-0,126580497	0,00098741
Q16222	0,916	0,99975746	-0,126580497	0,000105347
H3BMM9	0,916	0,99975746	-0,126580497	0,000105347
P08572	0,916	0,99975746	-0,126580497	0,000105347
A0A024R4M0	0,917	0,99975746	-0,125006361	0,000105347
Q9GZS3	0,917	0,99975746	-0,125006361	0,000105347
Q9NVX2	0,917	0,99975746	-0,125006361	0,000105347
O96013	0,917	0,99975746	-0,125006361	0,000105347
P62258	0,918	0,99975746	-0,123433941	0,000105347
P10515	0,918	0,99975746	-0,123433941	0,000105347
P19623	0,918	0,99975746	-0,123433941	0,000105347
Q06203	0,918	0,99576809	-0,123433941	0,001841795
Q6PCE3	0,918	0,99975746	-0,123433941	0,000105347
P35232	0,919	0,99975746	-0,121863233	0,000105347
P43686	0,919	0,99975746	-0,121863233	0,000105347
P18077	0,919	0,99975746	-0,121863233	0,000105347
Q13155	0,919	0,99576809	-0,121863233	0,001841795
Q16643	0,919	0,99975746	-0,121863233	0,000105347
P19174	0,919	0,99975746	-0,121863233	0,000105347
P53367	0,919	0,99975746	-0,121863233	0,000105347
C9JJ19	0,919	0,99975746	-0,121863233	0,000105347
P52306	0,919	0,99975746	-0,121863233	0,000105347
H0Y6U5	0,919	0,99975746	-0,121863233	0,000105347

P78345	0,919	0,99975746	-0,121863233	0,000105347
Q96GC5	0,919	0,99975746	-0,121863233	0,000105347
E7ESJ7	0,919	0,99975746	-0,121863233	0,000105347
Q9UMS4	0,92	0,99975746	-0,120294234	0,000105347
P62701	0,92	0,99975746	-0,120294234	0,000105347
P57740	0,92	0,99576809	-0,120294234	0,001841795
Q99567	0,92	0,99975746	-0,120294234	0,000105347
Q9Y2L1	0,92	0,99975746	-0,120294234	0,000105347
A0A087WYR0	0,92	0,99975746	-0,120294234	0,000105347
Q6UXH1	0,92	0,99975746	-0,120294234	0,000105347
Q12931	0,921	0,99975746	-0,118726939	0,000105347
P55884	0,921	0,99975746	-0,118726939	0,000105347
Q5JTH9	0,921	0,99975746	-0,118726939	0,000105347
G3V180	0,921	0,997663787	-0,118726939	0,001015791
C9J2Y9	0,921	0,99975746	-0,118726939	0,000105347
P11498	0,921	0,99975746	-0,118726939	0,000105347
Q9NPF4	0,921	0,99975746	-0,118726939	0,000105347
P0CG08	0,921	0,99975746	-0,118726939	0,000105347
P51452	0,921	0,99975746	-0,118726939	0,000105347
Q9UMX5	0,921	0,99975746	-0,118726939	0,000105347
P62241	0,922	0,99975746	-0,117161344	0,000105347
P30084	0,922	0,99975746	-0,117161344	0,000105347
E7EU96	0,922	0,99975746	-0,117161344	0,000105347
P62714	0,922	0,99975746	-0,117161344	0,000105347
F5H7Y6	0,922	0,99975746	-0,117161344	0,000105347
Q15369	0,922	0,998739524	-0,117161344	0,000547763
E9PQ57	0,922	0,99975746	-0,117161344	0,000105347
E7EWK3	0,922	0,99975746	-0,117161344	0,000105347
J3QKW2	0,922	0,99975746	-0,117161344	0,000105347
Q9BU23	0,922	0,99975746	-0,117161344	0,000105347
P05091	0,923	0,99975746	-0,115597447	0,000105347
P28066	0,923	0,99975746	-0,115597447	0,000105347
O43301	0,923	0,99975746	-0,115597447	0,000105347
B4E0K5	0,923	0,99975746	-0,115597447	0,000105347
F8VUA2	0,923	0,99975746	-0,115597447	0,000105347
P62633	0,923	0,99975746	-0,115597447	0,000105347
A0A087WX58	0,923	0,99975746	-0,115597447	0,000105347
Q9NUT2	0,923	0,99975746	-0,115597447	0,000105347
A0A0G2JLB3	0,923	0,99975746	-0,115597447	0,000105347
Q6ZNB6	0,923	0,99975746	-0,115597447	0,000105347
P05783	0,924	0,99975746	-0,114035243	0,000105347
P34932	0,924	0,99975746	-0,114035243	0,000105347
Q9NZI8	0,924	0,99975746	-0,114035243	0,000105347
P43490	0,924	0,99975746	-0,114035243	0,000105347
O75400	0,924	0,99975746	-0,114035243	0,000105347
Q99747	0,924	0,99975746	-0,114035243	0,000105347
Q99598	0,924	0,99975746	-0,114035243	0,000105347

Q9UFC0	0,924	0,99975746	-0,114035243	0,000105347
Q6P4A7	0,924	0,99975746	-0,114035243	0,000105347
P49848	0,924	0,99975746	-0,114035243	0,000105347
Q6Y1H2	0,924	0,99975746	-0,114035243	0,000105347
P08236	0,924	0,99975746	-0,114035243	0,000105347
P28074	0,925	0,99975746	-0,112474729	0,000105347
Q7L8L6	0,925	0,99975746	-0,112474729	0,000105347
P53701	0,925	0,99975746	-0,112474729	0,000105347
Q96CW5	0,925	0,99975746	-0,112474729	0,000105347
Q9UKF6	0,925	0,99975746	-0,112474729	0,000105347
A0A3B3IUD2	0,925	0,99975746	-0,112474729	0,000105347
Q8TB37	0,925	0,99975746	-0,112474729	0,000105347
P12956	0,926	0,99975746	-0,110915901	0,000105347
P63244	0,926	0,99975746	-0,110915901	0,000105347
O43143	0,926	0,99975746	-0,110915901	0,000105347
E9PKZ0	0,926	0,99975746	-0,110915901	0,000105347
O43488	0,926	0,99975746	-0,110915901	0,000105347
Q9H3N1	0,926	0,99975746	-0,110915901	0,000105347
V9GZ56	0,926	0,99975746	-0,110915901	0,000105347
O75718	0,926	0,99975746	-0,110915901	0,000105347
P98175	0,926	0,99975746	-0,110915901	0,000105347
O43324	0,926	0,99975746	-0,110915901	0,000105347
Q9Y5L4	0,926	0,99975746	-0,110915901	0,000105347
Q9BQG0	0,927	0,99975746	-0,109358756	0,000105347
H3BNC9	0,927	0,99975746	-0,109358756	0,000105347
P49720	0,927	0,99975746	-0,109358756	0,000105347
Q9NT62	0,927	0,99975746	-0,109358756	0,000105347
Q9BPW8	0,927	0,99975746	-0,109358756	0,000105347
Q9BTX1	0,927	0,99975746	-0,109358756	0,000105347
Q8N511	0,927	0,99975746	-0,109358756	0,000105347
Q96M27	0,927	0,99975746	-0,109358756	0,000105347
P26641	0,928	0,99975746	-0,10780329	0,000105347
P12004	0,928	0,99975746	-0,10780329	0,000105347
P36578	0,928	0,99975746	-0,10780329	0,000105347
Q9H0D6	0,928	0,99975746	-0,10780329	0,000105347
P11177	0,928	0,99975746	-0,10780329	0,000105347
F6T1Q0	0,928	0,99975746	-0,10780329	0,000105347
Q9NS86	0,928	0,99975746	-0,10780329	0,000105347
Q96HS1	0,928	0,99975746	-0,10780329	0,000105347
Q15750	0,928	0,99975746	-0,10780329	0,000105347
P36404	0,928	0,99975746	-0,10780329	0,000105347
O60783	0,928	0,99975746	-0,10780329	0,000105347
P82675	0,928	0,99975746	-0,10780329	0,000105347
Q16762	0,928	0,99975746	-0,10780329	0,000105347
A0A0J9YWM3	0,928	0,99975746	-0,10780329	0,000105347
Q6ZXV5	0,928	0,99975746	-0,10780329	0,000105347
Q92609	0,928	0,99975746	-0,10780329	0,000105347

Q9NS87	0,928	0,99975746	-0,10780329	0,000105347
Q9BRZ2	0,928	0,99975746	-0,10780329	0,000105347
P41252	0,929	0,99975746	-0,106249498	0,000105347
Q9Y6C9	0,929	0,99975746	-0,106249498	0,000105347
M0QWZ7	0,929	0,99975746	-0,106249498	0,000105347
H3BPJ9	0,929	0,99975746	-0,106249498	0,000105347
E9PRK2	0,929	0,99975746	-0,106249498	0,000105347
Q6UX04	0,929	0,99975746	-0,106249498	0,000105347
O60678	0,929	0,99975746	-0,106249498	0,000105347
A0A087WVQ6	0,93	0,99975746	-0,104697379	0,000105347
P14625	0,93	0,99975746	-0,104697379	0,000105347
P17174	0,93	0,99975746	-0,104697379	0,000105347
Q9BPX3	0,93	0,99975746	-0,104697379	0,000105347
Q9Y5X1	0,93	0,99975746	-0,104697379	0,000105347
P62834	0,93	0,99975746	-0,104697379	0,000105347
Q9P2I0	0,93	0,99975746	-0,104697379	0,000105347
Q00535	0,93	0,99975746	-0,104697379	0,000105347
O75348	0,93	0,99975746	-0,104697379	0,000105347
P13667	0,931	0,99975746	-0,103146927	0,000105347
Q9Y4L1	0,931	0,99975746	-0,103146927	0,000105347
Q9Y266	0,931	0,99975746	-0,103146927	0,000105347
Q99623	0,931	0,99975746	-0,103146927	0,000105347
O15371	0,931	0,99975746	-0,103146927	0,000105347
P56537	0,931	0,99975746	-0,103146927	0,000105347
J3KRX5	0,931	0,99975746	-0,103146927	0,000105347
H0Y993	0,931	0,99975746	-0,103146927	0,000105347
P39748	0,931	0,99975746	-0,103146927	0,000105347
F8W733	0,931	0,99975746	-0,103146927	0,000105347
P60520	0,931	0,99975746	-0,103146927	0,000105347
A4D1E9	0,931	0,99975746	-0,103146927	0,000105347
Q9Y2P8	0,931	0,99975746	-0,103146927	0,000105347
Q15631	0,932	0,99975746	-0,10159814	0,000105347
P51610	0,932	0,99975746	-0,10159814	0,000105347
Q66K74	0,932	0,99975746	-0,10159814	0,000105347
P82664	0,932	0,99975746	-0,10159814	0,000105347
E9PM35	0,932	0,99975746	-0,10159814	0,000105347
P22102	0,933	0,99975746	-0,100051014	0,000105347
P28070	0,933	0,99975746	-0,100051014	0,000105347
G5EA06	0,933	0,99975746	-0,100051014	0,000105347
P09543	0,933	0,99975746	-0,100051014	0,000105347
O43719	0,933	0,99975746	-0,100051014	0,000105347
Q9BRP8	0,933	0,99975746	-0,100051014	0,000105347
Q7KZN9	0,933	0,99975746	-0,100051014	0,000105347
O60934	0,933	0,99975746	-0,100051014	0,000105347
Q9UEU0	0,933	0,99975746	-0,100051014	0,000105347
B3KNS4	0,933	0,99975746	-0,100051014	0,000105347
Q07065	0,934	0,99975746	-0,098505545	0,000105347

O43592	0,934	0,99975746	-0,098505545	0,000105347
P51784	0,934	0,99975746	-0,098505545	0,000105347
P25787	0,934	0,99975746	-0,098505545	0,000105347
E7EVJ5	0,934	0,99975746	-0,098505545	0,000105347
P46734	0,934	0,99975746	-0,098505545	0,000105347
Q9H2P9	0,934	0,99975746	-0,098505545	0,000105347
P56385	0,934	0,99975746	-0,098505545	0,000105347
P60468	0,934	0,99975746	-0,098505545	0,000105347
Q15075	0,935	0,99975746	-0,09696173	0,000105347
Q9NSE4	0,935	0,99975746	-0,09696173	0,000105347
P16949	0,935	0,99975746	-0,09696173	0,000105347
Q13557	0,935	0,99975746	-0,09696173	0,000105347
P20618	0,935	0,99975746	-0,09696173	0,000105347
Q9Y2D5	0,935	0,99975746	-0,09696173	0,000105347
Q13564	0,935	0,99975746	-0,09696173	0,000105347
P60866	0,935	0,99975746	-0,09696173	0,000105347
P08240	0,935	0,99975746	-0,09696173	0,000105347
Q9BTC0	0,935	0,99975746	-0,09696173	0,000105347
Q9H0E2	0,935	0,99975746	-0,09696173	0,000105347
P60842	0,936	0,99975746	-0,095419565	0,000105347
P60228	0,936	0,99975746	-0,095419565	0,000105347
J3KP15	0,936	0,99975746	-0,095419565	0,000105347
E7ENY0	0,936	0,99975746	-0,095419565	0,000105347
Q8IZL8	0,936	0,99975746	-0,095419565	0,000105347
P42285	0,936	0,99975746	-0,095419565	0,000105347
P41567	0,936	0,99975746	-0,095419565	0,000105347
Q9BRK5	0,936	0,99975746	-0,095419565	0,000105347
Q9Y3C6	0,936	0,99975746	-0,095419565	0,000105347
O00139	0,936	0,99975746	-0,095419565	0,000105347
P49207	0,936	0,99975746	-0,095419565	0,000105347
Q9H2P0	0,936	0,99975746	-0,095419565	0,000105347
Q7Z2T5	0,936	0,99975746	-0,095419565	0,000105347
A0A2R8Y4Z8	0,936	0,99975746	-0,095419565	0,000105347
Q9UK61	0,936	0,99975746	-0,095419565	0,000105347
P41743	0,936	0,99975746	-0,095419565	0,000105347
G3V4K3	0,936	0,99975746	-0,095419565	0,000105347
P05023	0,937	0,99975746	-0,093879047	0,000105347
O75534	0,937	0,99975746	-0,093879047	0,000105347
O95239	0,937	0,99975746	-0,093879047	0,000105347
P13489	0,937	0,99975746	-0,093879047	0,000105347
P48163	0,937	0,99975746	-0,093879047	0,000105347
H7C0E5	0,937	0,99975746	-0,093879047	0,000105347
P04899	0,937	0,99975746	-0,093879047	0,000105347
Q6RW13	0,937	0,99975746	-0,093879047	0,000105347
Q6UW02	0,937	0,99975746	-0,093879047	0,000105347
Q8WYP5	0,937	0,99975746	-0,093879047	0,000105347
Q9ULC3	0,937	0,99975746	-0,093879047	0,000105347

Q9NZZ3	0,937	0,99975746	-0,093879047	0,000105347
Q71U36	0,938	0,99975746	-0,092340172	0,000105347
P07237	0,938	0,99975746	-0,092340172	0,000105347
Q14974	0,938	0,99975746	-0,092340172	0,000105347
P29692	0,938	0,99975746	-0,092340172	0,000105347
A0A494C1B8	0,938	0,99975746	-0,092340172	0,000105347
O15067	0,938	0,99975746	-0,092340172	0,000105347
Q09028	0,938	0,99975746	-0,092340172	0,000105347
Q969V3	0,938	0,99975746	-0,092340172	0,000105347
P63220	0,938	0,99975746	-0,092340172	0,000105347
Q9ULX3	0,938	0,99975746	-0,092340172	0,000105347
A0A494C1B1	0,938	0,99975746	-0,092340172	0,000105347
H3BQQ2	0,938	0,99975746	-0,092340172	0,000105347
P78527	0,939	0,99975746	-0,090802937	0,000105347
P13639	0,939	0,99975746	-0,090802937	0,000105347
O95831	0,939	0,99975746	-0,090802937	0,000105347
Q5T4U5	0,939	0,99975746	-0,090802937	0,000105347
Q7Z434	0,939	0,99975746	-0,090802937	0,000105347
Q0VDF9	0,939	0,99975746	-0,090802937	0,000105347
P50914	0,939	0,99975746	-0,090802937	0,000105347
P23786	0,939	0,99975746	-0,090802937	0,000105347
Q9NTJ5	0,939	0,99975746	-0,090802937	0,000105347
Q52LJ0	0,939	0,99975746	-0,090802937	0,000105347
Q9H9T3	0,939	0,99975746	-0,090802937	0,000105347
A0A024RAC6	0,939	0,99975746	-0,090802937	0,000105347
P07900	0,94	0,99975746	-0,089267338	0,000105347
Q96QK1	0,94	0,99975746	-0,089267338	0,000105347
O43615	0,94	0,99975746	-0,089267338	0,000105347
Q9Y277	0,94	0,99975746	-0,089267338	0,000105347
O95163	0,94	0,99975746	-0,089267338	0,000105347
A0A494C0X0	0,94	0,99975746	-0,089267338	0,000105347
Q8IYQ7	0,94	0,99975746	-0,089267338	0,000105347
O43264	0,94	0,99975746	-0,089267338	0,000105347
Q96ME7	0,94	0,99975746	-0,089267338	0,000105347
Q13042	0,94	0,99975746	-0,089267338	0,000105347
Q9Y2Z4	0,94	0,99975746	-0,089267338	0,000105347
Q8NCF5	0,94	0,99975746	-0,089267338	0,000105347
Q7Z6Z7	0,941	0,99975746	-0,087733372	0,000105347
P11169	0,941	0,99975746	-0,087733372	0,000105347
O43809	0,941	0,99975746	-0,087733372	0,000105347
P35249	0,941	0,99975746	-0,087733372	0,000105347
Q13153	0,941	0,99975746	-0,087733372	0,000105347
Q9UHD9	0,941	0,99975746	-0,087733372	0,000105347
Q96HY6	0,941	0,99975746	-0,087733372	0,000105347
Q96BP3	0,941	0,99975746	-0,087733372	0,000105347
P23526	0,942	0,99975746	-0,086201035	0,000105347
P04792	0,942	0,99975746	-0,086201035	0,000105347

P62195	0,942	0,99975746	-0,086201035	0,000105347
Q92621	0,942	0,99975746	-0,086201035	0,000105347
Q01105	0,942	0,99975746	-0,086201035	0,000105347
Q9UDY2	0,942	0,99975746	-0,086201035	0,000105347
Q8IVD9	0,942	0,99975746	-0,086201035	0,000105347
Q9Y512	0,942	0,99975746	-0,086201035	0,000105347
Q13190	0,942	0,99975746	-0,086201035	0,000105347
Q8WZA9	0,942	0,99975746	-0,086201035	0,000105347
M0R1B0	0,942	0,99975746	-0,086201035	0,000105347
P45973	0,942	0,99975746	-0,086201035	0,000105347
P27348	0,943	0,99975746	-0,084670324	0,000105347
Q13347	0,943	0,99975746	-0,084670324	0,000105347
O95433	0,943	0,99975746	-0,084670324	0,000105347
Q9Y6E2	0,943	0,99975746	-0,084670324	0,000105347
Q99436	0,943	0,99975746	-0,084670324	0,000105347
Q13098	0,943	0,99975746	-0,084670324	0,000105347
Q99543	0,943	0,99975746	-0,084670324	0,000105347
Q9UBW8	0,943	0,99975746	-0,084670324	0,000105347
Q16718	0,943	0,99975746	-0,084670324	0,000105347
Q9NUL7	0,943	0,99975746	-0,084670324	0,000105347
Q14331	0,943	0,99975746	-0,084670324	0,000105347
Q9H5X1	0,943	0,99975746	-0,084670324	0,000105347
P51151	0,943	0,99975746	-0,084670324	0,000105347
Q99460	0,944	0,99975746	-0,083141235	0,000105347
P22695	0,944	0,99975746	-0,083141235	0,000105347
P39019	0,944	0,99975746	-0,083141235	0,000105347
A0A087WYT3	0,944	0,99975746	-0,083141235	0,000105347
P62851	0,944	0,99975746	-0,083141235	0,000105347
Q96EE3	0,944	0,99975746	-0,083141235	0,000105347
Q9Y639	0,944	0,99975746	-0,083141235	0,000105347
O15126	0,944	0,99975746	-0,083141235	0,000105347
Q5TA50	0,944	0,99975746	-0,083141235	0,000105347
F8WAN9	0,944	0,99975746	-0,083141235	0,000105347
O15231	0,944	0,99975746	-0,083141235	0,000105347
Q6P2Q9	0,945	0,99975746	-0,081613766	0,000105347
P45974	0,945	0,99975746	-0,081613766	0,000105347
Q14914	0,945	0,99975746	-0,081613766	0,000105347
P68036	0,945	0,99975746	-0,081613766	0,000105347
O60826	0,945	0,99975746	-0,081613766	0,000105347
Q9NY27	0,945	0,99975746	-0,081613766	0,000105347
P42858	0,945	0,99975746	-0,081613766	0,000105347
O14818	0,946	0,99975746	-0,080087911	0,000105347
Q9UPU5	0,946	0,99975746	-0,080087911	0,000105347
O14530	0,946	0,99975746	-0,080087911	0,000105347
Q14232	0,946	0,99975746	-0,080087911	0,000105347
Q92600	0,946	0,99975746	-0,080087911	0,000105347
Q92947	0,946	0,99975746	-0,080087911	0,000105347

Q8IYS2	0,946	0,99975746	-0,080087911	0,000105347
P52597	0,947	0,99975746	-0,078563669	0,000105347
P54578	0,947	0,99975746	-0,078563669	0,000105347
P06493	0,947	0,99975746	-0,078563669	0,000105347
P18583	0,947	0,99975746	-0,078563669	0,000105347
Q8WYA6	0,947	0,99975746	-0,078563669	0,000105347
P00441	0,947	0,99975746	-0,078563669	0,000105347
Q9BRJ6	0,947	0,99975746	-0,078563669	0,000105347
O00743	0,947	0,99975746	-0,078563669	0,000105347
P46199	0,947	0,99975746	-0,078563669	0,000105347
Q96ER3	0,947	0,99975746	-0,078563669	0,000105347
E7EUN9	0,947	0,99975746	-0,078563669	0,000105347
P25705	0,948	0,99975746	-0,077041036	0,000105347
Q10567	0,948	0,99975746	-0,077041036	0,000105347
O14979	0,948	0,99975746	-0,077041036	0,000105347
Q12907	0,948	0,99975746	-0,077041036	0,000105347
Q13573	0,948	0,99975746	-0,077041036	0,000105347
C9JLU1	0,948	0,99975746	-0,077041036	0,000105347
F5GXX5	0,948	0,99975746	-0,077041036	0,000105347
P25325	0,948	0,99975746	-0,077041036	0,000105347
Q9NPD3	0,948	0,99975746	-0,077041036	0,000105347
P13010	0,949	0,99975746	-0,075520008	0,000105347
P30050	0,949	0,99975746	-0,075520008	0,000105347
Q9NYF8	0,949	0,99975746	-0,075520008	0,000105347
J3KR44	0,949	0,99975746	-0,075520008	0,000105347
P22087	0,949	0,99975746	-0,075520008	0,000105347
P31937	0,949	0,99975746	-0,075520008	0,000105347
Q14166	0,949	0,99975746	-0,075520008	0,000105347
C9JQD4	0,949	0,99975746	-0,075520008	0,000105347
P68106	0,949	0,99975746	-0,075520008	0,000105347
Q8N1G4	0,95	0,99975746	-0,074000581	0,000105347
Q13162	0,95	0,99975746	-0,074000581	0,000105347
P62269	0,95	0,99975746	-0,074000581	0,000105347
P25786	0,95	0,99975746	-0,074000581	0,000105347
Q6IN85	0,95	0,99975746	-0,074000581	0,000105347
P61254	0,95	0,99975746	-0,074000581	0,000105347
O75569	0,95	0,99975746	-0,074000581	0,000105347
B5MCF9	0,95	0,99975746	-0,074000581	0,000105347
Q9BW27	0,95	0,99975746	-0,074000581	0,000105347
O75381	0,95	0,99975746	-0,074000581	0,000105347
Q9UBD5	0,95	0,99975746	-0,074000581	0,000105347
Q14696	0,95	0,99975746	-0,074000581	0,000105347
H0Y547	0,95	0,99975746	-0,074000581	0,000105347
Q13123	0,95	0,99975746	-0,074000581	0,000105347
A0A024QZ42	0,95	0,99975746	-0,074000581	0,000105347
Q96S55	0,95	0,99975746	-0,074000581	0,000105347
P06576	0,951	0,99975746	-0,072482754	0,000105347

Q8TCS8	0,951	0,99975746	-0,072482754	0,000105347
P36871	0,951	0,99975746	-0,072482754	0,000105347
Q9Y262	0,951	0,99975746	-0,072482754	0,000105347
P08779	0,951	0,99975746	-0,072482754	0,000105347
Q9NTX5	0,951	0,99975746	-0,072482754	0,000105347
P42677	0,951	0,99975746	-0,072482754	0,000105347
Q96GM8	0,951	0,99975746	-0,072482754	0,000105347
P61081	0,951	0,99975746	-0,072482754	0,000105347
P05026	0,951	0,99975746	-0,072482754	0,000105347
C9J406	0,952	0,99975746	-0,070966521	0,000105347
Q9Y285	0,952	0,99975746	-0,070966521	0,000105347
Q9NTJ3	0,952	0,99975746	-0,070966521	0,000105347
P61604	0,952	0,99975746	-0,070966521	0,000105347
Q6YN16	0,952	0,99975746	-0,070966521	0,000105347
Q7Z3B4	0,952	0,99975746	-0,070966521	0,000105347
Q96GA3	0,952	0,99975746	-0,070966521	0,000105347
J3KNR0	0,952	0,99975746	-0,070966521	0,000105347
P62424	0,953	0,99975746	-0,069451881	0,000105347
P13798	0,953	0,99975746	-0,069451881	0,000105347
A0A2R8Y811	0,953	0,99975746	-0,069451881	0,000105347
Q96PZ0	0,953	0,99975746	-0,069451881	0,000105347
Q9NZL4	0,953	0,99975746	-0,069451881	0,000105347
P61088	0,953	0,99975746	-0,069451881	0,000105347
Q96EY7	0,953	0,99975746	-0,069451881	0,000105347
A0A087WUC6	0,953	0,99975746	-0,069451881	0,000105347
Q96CN7	0,953	0,99975746	-0,069451881	0,000105347
Q9NW13	0,953	0,99975746	-0,069451881	0,000105347
Q9P0T7	0,953	0,99975746	-0,069451881	0,000105347
Q9NX14	0,953	0,99975746	-0,069451881	0,000105347
C9J014	0,953	0,99975746	-0,069451881	0,000105347
Q13509	0,954	0,99975746	-0,067938829	0,000105347
P50395	0,954	0,99975746	-0,067938829	0,000105347
P38919	0,954	0,99975746	-0,067938829	0,000105347
Q9BWD1	0,954	0,99975746	-0,067938829	0,000105347
Q9UJA5	0,954	0,99975746	-0,067938829	0,000105347
Q2TAY7	0,954	0,99975746	-0,067938829	0,000105347
Q9H0C8	0,954	0,99975746	-0,067938829	0,000105347
Q9H477	0,954	0,99975746	-0,067938829	0,000105347
K7ERI7	0,954	0,99975746	-0,067938829	0,000105347
Q9UKR5	0,954	0,99975746	-0,067938829	0,000105347
P06733	0,955	0,99975746	-0,066427362	0,000105347
O75643	0,955	0,99975746	-0,066427362	0,000105347
P19338	0,955	0,99975746	-0,066427362	0,000105347
P41091	0,955	0,99975746	-0,066427362	0,000105347
O76094	0,955	0,99975746	-0,066427362	0,000105347
S4R3H4	0,955	0,99975746	-0,066427362	0,000105347
A0A1B0GTJ7	0,955	0,99975746	-0,066427362	0,000105347

P80723	0,955	0,99975746	-0,066427362	0,000105347
O15270	0,955	0,99975746	-0,066427362	0,000105347
P10619	0,955	0,99975746	-0,066427362	0,000105347
Q9HCC0	0,955	0,99975746	-0,066427362	0,000105347
E9PLL6	0,955	0,99975746	-0,066427362	0,000105347
Q9BXR0	0,955	0,99975746	-0,066427362	0,000105347
Q6IA86	0,955	0,99975746	-0,066427362	0,000105347
Q96E29	0,955	0,99975746	-0,066427362	0,000105347
Q13435	0,956	0,99975746	-0,064917477	0,000105347
P13929	0,956	0,99975746	-0,064917477	0,000105347
Q6DKJ4	0,956	0,99975746	-0,064917477	0,000105347
P54619	0,956	0,99975746	-0,064917477	0,000105347
P35658	0,956	0,99975746	-0,064917477	0,000105347
Q96RE7	0,956	0,99975746	-0,064917477	0,000105347
Q15036	0,956	0,99975746	-0,064917477	0,000105347
Q15018	0,956	0,99975746	-0,064917477	0,000105347
F5GX71	0,956	0,99975746	-0,064917477	0,000105347
P21281	0,957	0,99975746	-0,06340917	0,000105347
Q9HDC9	0,957	0,99975746	-0,06340917	0,000105347
O75940	0,957	0,99975746	-0,06340917	0,000105347
F8WCT1	0,957	0,99975746	-0,06340917	0,000105347
Q9BUJ2	0,958	0,99975746	-0,061902439	0,000105347
B1ALA9	0,958	0,99975746	-0,061902439	0,000105347
E5RJR5	0,958	0,99975746	-0,061902439	0,000105347
O14936	0,958	0,99975746	-0,061902439	0,000105347
P53999	0,958	0,99975746	-0,061902439	0,000105347
P42126	0,958	0,99975746	-0,061902439	0,000105347
Q9H3Z4	0,958	0,99975746	-0,061902439	0,000105347
Q9NXW2	0,958	0,99975746	-0,061902439	0,000105347
P01111	0,958	0,99975746	-0,061902439	0,000105347
J3KMY0	0,958	0,99975746	-0,061902439	0,000105347
A0A0A0MR04	0,958	0,99975746	-0,061902439	0,000105347
P49411	0,959	0,99975746	-0,06039728	0,000105347
A0A1B0GTG2	0,959	0,99975746	-0,06039728	0,000105347
Q15418	0,959	0,99975746	-0,06039728	0,000105347
Q12904	0,959	0,99975746	-0,06039728	0,000105347
Q9Y3T9	0,959	0,99975746	-0,06039728	0,000105347
A0A2Q2TH77	0,959	0,99975746	-0,06039728	0,000105347
A0A0A0MRM9	0,959	0,99975746	-0,06039728	0,000105347
P28072	0,959	0,99975746	-0,06039728	0,000105347
P10620	0,959	0,99975746	-0,06039728	0,000105347
Q96ER9	0,959	0,99975746	-0,06039728	0,000105347
Q9P0V3	0,959	0,99975746	-0,06039728	0,000105347
P14927	0,959	0,99975746	-0,06039728	0,000105347
Q15031	0,959	0,99975746	-0,06039728	0,000105347
Q8N201	0,959	0,99975746	-0,06039728	0,000105347
Q8N999	0,959	0,99975746	-0,06039728	0,000105347

P30101	0,96	0,99975746	-0,058893689	0,000105347
P33176	0,96	0,99975746	-0,058893689	0,000105347
Q92900	0,96	0,99975746	-0,058893689	0,000105347
P30740	0,96	0,99975746	-0,058893689	0,000105347
P62244	0,96	0,99975746	-0,058893689	0,000105347
Q9H2W6	0,96	0,99975746	-0,058893689	0,000105347
Q5JPH6	0,96	0,99975746	-0,058893689	0,000105347
A0A1W2PNW1	0,96	0,99975746	-0,058893689	0,000105347
P61289	0,961	0,99975746	-0,057391664	0,000105347
P62140	0,961	0,99975746	-0,057391664	0,000105347
Q15435	0,961	0,99975746	-0,057391664	0,000105347
A0A087X0M4	0,961	0,99975746	-0,057391664	0,000105347
E7EM64	0,961	0,99975746	-0,057391664	0,000105347
O75150	0,961	0,99975746	-0,057391664	0,000105347
P41223	0,961	0,99975746	-0,057391664	0,000105347
Q02127	0,961	0,99975746	-0,057391664	0,000105347
P11413	0,962	0,99975746	-0,055891201	0,000105347
P30048	0,962	0,99975746	-0,055891201	0,000105347
Q15084	0,962	0,99975746	-0,055891201	0,000105347
Q9NTZ6	0,962	0,99975746	-0,055891201	0,000105347
O43396	0,962	0,99975746	-0,055891201	0,000105347
Q96A33	0,962	0,99975746	-0,055891201	0,000105347
Q9P2M7	0,962	0,99975746	-0,055891201	0,000105347
E7ERK9	0,962	0,99975746	-0,055891201	0,000105347
Q16204	0,962	0,99975746	-0,055891201	0,000105347
P46937	0,962	0,99975746	-0,055891201	0,000105347
O15226	0,962	0,99975746	-0,055891201	0,000105347
Q92615	0,962	0,99975746	-0,055891201	0,000105347
O00231	0,963	0,99975746	-0,054392297	0,000105347
Q9Y520	0,963	0,99975746	-0,054392297	0,000105347
Q03252	0,963	0,99975746	-0,054392297	0,000105347
P30419	0,963	0,99975746	-0,054392297	0,000105347
P36542	0,963	0,99975746	-0,054392297	0,000105347
P48506	0,963	0,99975746	-0,054392297	0,000105347
A0A1W2PS43	0,963	0,99975746	-0,054392297	0,000105347
Q8TEA8	0,963	0,99975746	-0,054392297	0,000105347
Q969X5	0,963	0,99975746	-0,054392297	0,000105347
P07737	0,964	0,99975746	-0,052894948	0,000105347
Q01469	0,964	0,99975746	-0,052894948	0,000105347
E9PKG1	0,964	0,99975746	-0,052894948	0,000105347
Q8N6T3	0,964	0,99975746	-0,052894948	0,000105347
Q9Y2Z0	0,964	0,99975746	-0,052894948	0,000105347
Q9BT78	0,964	0,99975746	-0,052894948	0,000105347
P62913	0,964	0,99975746	-0,052894948	0,000105347
K7ELG9	0,964	0,99975746	-0,052894948	0,000105347
P17050	0,964	0,99975746	-0,052894948	0,000105347
Q9UKK6	0,964	0,99975746	-0,052894948	0,000105347

O60306	0,964	0,99975746	-0,052894948	0,000105347
P53396	0,965	0,99975746	-0,051399153	0,000105347
O96005	0,965	0,99975746	-0,051399153	0,000105347
O14841	0,965	0,99975746	-0,051399153	0,000105347
Q86YV9	0,965	0,99975746	-0,051399153	0,000105347
Q92945	0,966	0,99975746	-0,049904906	0,000105347
A0A2R8Y6J3	0,966	0,99975746	-0,049904906	0,000105347
P51648	0,966	0,99975746	-0,049904906	0,000105347
E9PRJ8	0,966	0,99975746	-0,049904906	0,000105347
J3KQ48	0,966	0,99975746	-0,049904906	0,000105347
P07741	0,966	0,99975746	-0,049904906	0,000105347
O15269	0,966	0,99975746	-0,049904906	0,000105347
Q5QP56	0,966	0,99975746	-0,049904906	0,000105347
Q96N67	0,966	0,99975746	-0,049904906	0,000105347
Q6NUQ1	0,966	0,99975746	-0,049904906	0,000105347
P18669	0,967	0,99975746	-0,048412205	0,000105347
A0A087WWU8	0,967	0,99975746	-0,048412205	0,000105347
Q9P0I2	0,967	0,99975746	-0,048412205	0,000105347
O14656	0,967	0,99975746	-0,048412205	0,000105347
P05198	0,968	0,99975746	-0,046921047	0,000105347
Q00325	0,968	0,99975746	-0,046921047	0,000105347
E7EPM6	0,968	0,99975746	-0,046921047	0,000105347
B3KS98	0,968	0,99975746	-0,046921047	0,000105347
P55265	0,968	0,99975746	-0,046921047	0,000105347
E9PDF2	0,968	0,99975746	-0,046921047	0,000105347
Q14789	0,968	0,99975746	-0,046921047	0,000105347
A0A2R8Y7U1	0,968	0,99975746	-0,046921047	0,000105347
J3KS22	0,968	0,99975746	-0,046921047	0,000105347
O95456	0,968	0,99975746	-0,046921047	0,000105347
P53990	0,968	0,99975746	-0,046921047	0,000105347
Q6P1M0	0,968	0,99975746	-0,046921047	0,000105347
Q96JG6	0,968	0,99975746	-0,046921047	0,000105347
P61247	0,969	0,99975746	-0,045431429	0,000105347
Q9BXJ9	0,969	0,99975746	-0,045431429	0,000105347
P56192	0,969	0,99975746	-0,045431429	0,000105347
Q8WUM0	0,969	0,99975746	-0,045431429	0,000105347
R4GMR5	0,969	0,99975746	-0,045431429	0,000105347
A8MZF9	0,969	0,99975746	-0,045431429	0,000105347
P61970	0,969	0,99975746	-0,045431429	0,000105347
Q9BRX2	0,969	0,99975746	-0,045431429	0,000105347
Q8N1G2	0,969	0,99975746	-0,045431429	0,000105347
Q13813	0,97	0,99975746	-0,043943348	0,000105347
P30153	0,97	0,99975746	-0,043943348	0,000105347
Q7L2H7	0,97	0,99975746	-0,043943348	0,000105347
Q9UI26	0,97	0,99975746	-0,043943348	0,000105347
Q86Y07	0,97	0,99975746	-0,043943348	0,000105347
J3QRV5	0,97	0,99975746	-0,043943348	0,000105347

Q8NBN7	0,97	0,99975746	-0,043943348	0,000105347
Q9H9F9	0,97	0,99975746	-0,043943348	0,000105347
O43242	0,971	0,99975746	-0,042456799	0,000105347
Q02790	0,971	0,99975746	-0,042456799	0,000105347
P41250	0,971	0,99975746	-0,042456799	0,000105347
P50502	0,971	0,99975746	-0,042456799	0,000105347
P55010	0,971	0,99975746	-0,042456799	0,000105347
P82979	0,971	0,99975746	-0,042456799	0,000105347
P36915	0,971	0,99975746	-0,042456799	0,000105347
C9JDU0	0,971	0,99975746	-0,042456799	0,000105347
P00558	0,972	0,99975746	-0,040971781	0,000105347
Q8NC51	0,972	0,99975746	-0,040971781	0,000105347
Q15907	0,972	0,99975746	-0,040971781	0,000105347
Q9Y5S9	0,972	0,99975746	-0,040971781	0,000105347
P61020	0,972	0,99975746	-0,040971781	0,000105347
O00507	0,972	0,99975746	-0,040971781	0,000105347
B2WTI3	0,972	0,99975746	-0,040971781	0,000105347
P50542	0,972	0,99975746	-0,040971781	0,000105347
Q86U44	0,972	0,99975746	-0,040971781	0,000105347
B7Z7P8	0,973	0,99975746	-0,03948829	0,000105347
P18124	0,973	0,99975746	-0,03948829	0,000105347
P23381	0,973	0,99975746	-0,03948829	0,000105347
Q9NSD9	0,973	0,99975746	-0,03948829	0,000105347
Q9Y263	0,973	0,99975746	-0,03948829	0,000105347
P61221	0,973	0,99975746	-0,03948829	0,000105347
O15260	0,973	0,99975746	-0,03948829	0,000105347
A1X283	0,973	0,99975746	-0,03948829	0,000105347
Q9NRL3	0,973	0,99975746	-0,03948829	0,000105347
Q15118	0,973	0,99975746	-0,03948829	0,000105347
P55072	0,974	0,99975746	-0,038006323	0,000105347
O60701	0,974	0,99975746	-0,038006323	0,000105347
P62750	0,974	0,99975746	-0,038006323	0,000105347
Q8N766	0,974	0,99975746	-0,038006323	0,000105347
A0A2R8Y849	0,974	0,99975746	-0,038006323	0,000105347
C9JRZ6	0,974	0,99975746	-0,038006323	0,000105347
P06756	0,974	0,99975746	-0,038006323	0,000105347
Q92620	0,974	0,99975746	-0,038006323	0,000105347
P11021	0,975	0,99975746	-0,036525876	0,000105347
P62937	0,975	0,99975746	-0,036525876	0,000105347
O00303	0,975	0,99975746	-0,036525876	0,000105347
Q8TEQ6	0,975	0,99975746	-0,036525876	0,000105347
P00387	0,975	0,99975746	-0,036525876	0,000105347
Q9NUQ8	0,975	0,99975746	-0,036525876	0,000105347
O95400	0,975	0,99975746	-0,036525876	0,000105347
Q8IZ83	0,975	0,99975746	-0,036525876	0,000105347
O00571	0,976	0,99975746	-0,035046947	0,000105347
P04843	0,976	0,99975746	-0,035046947	0,000105347

P42765	0,976	0,99975746	-0,035046947	0,000105347
Q9Y2B0	0,976	0,99975746	-0,035046947	0,000105347
P61513	0,976	0,99975746	-0,035046947	0,000105347
P23258	0,976	0,99975746	-0,035046947	0,000105347
E7EVX8	0,976	0,99975746	-0,035046947	0,000105347
Q15006	0,976	0,99975746	-0,035046947	0,000105347
P27487	0,976	0,99975746	-0,035046947	0,000105347
Q9BTA9	0,976	0,99975746	-0,035046947	0,000105347
Q9BVA1	0,977	0,99975746	-0,033569533	0,000105347
Q16531	0,977	0,99975746	-0,033569533	0,000105347
Q96PK6	0,977	0,99975746	-0,033569533	0,000105347
Q9BY44	0,977	0,99975746	-0,033569533	0,000105347
Q9BWF3	0,977	0,99975746	-0,033569533	0,000105347
Q92905	0,977	0,99975746	-0,033569533	0,000105347
Q15833	0,977	0,99975746	-0,033569533	0,000105347
Q96GK7	0,977	0,99975746	-0,033569533	0,000105347
P27797	0,978	0,99975746	-0,03209363	0,000105347
Q15029	0,978	0,99975746	-0,03209363	0,000105347
P08237	0,978	0,99975746	-0,03209363	0,000105347
Q9BUL8	0,978	0,99975746	-0,03209363	0,000105347
Q9Y295	0,978	0,99975746	-0,03209363	0,000105347
P14174	0,978	0,99975746	-0,03209363	0,000105347
Q7Z4S6	0,978	0,99975746	-0,03209363	0,000105347
P30046	0,978	0,99975746	-0,03209363	0,000105347
P49773	0,978	0,99975746	-0,03209363	0,000105347
Q15427	0,978	0,99975746	-0,03209363	0,000105347
P62191	0,979	0,99975746	-0,030619235	0,000105347
P38117	0,979	0,99975746	-0,030619235	0,000105347
P61011	0,979	0,99975746	-0,030619235	0,000105347
Q9H488	0,979	0,99975746	-0,030619235	0,000105347
P09668	0,979	0,99975746	-0,030619235	0,000105347
R4GMX3	0,979	0,99975746	-0,030619235	0,000105347
Q8NE86	0,979	0,99975746	-0,030619235	0,000105347
P32929	0,979	0,99975746	-0,030619235	0,000105347
Q15365	0,98	0,99975746	-0,029146346	0,000105347
P55036	0,98	0,99975746	-0,029146346	0,000105347
P40222	0,98	0,99975746	-0,029146346	0,000105347
P62829	0,98	0,99975746	-0,029146346	0,000105347
P14550	0,98	0,99975746	-0,029146346	0,000105347
P24539	0,98	0,99975746	-0,029146346	0,000105347
P62249	0,98	0,99975746	-0,029146346	0,000105347
Q5T5P2	0,98	0,99975746	-0,029146346	0,000105347
E9PQW4	0,98	0,99975746	-0,029146346	0,000105347
Q9UBM7	0,98	0,99975746	-0,029146346	0,000105347
O95777	0,98	0,99975746	-0,029146346	0,000105347
O75369	0,981	0,99975746	-0,027674958	0,000105347
P32119	0,981	0,99975746	-0,027674958	0,000105347

Q07666	0,981	0,99975746	-0,027674958	0,000105347
O60502	0,981	0,99975746	-0,027674958	0,000105347
Q9BRA2	0,981	0,99975746	-0,027674958	0,000105347
Q9H078	0,981	0,99975746	-0,027674958	0,000105347
Q96EK9	0,981	0,99975746	-0,027674958	0,000105347
Q96BP2	0,981	0,99975746	-0,027674958	0,000105347
Q9HAV4	0,982	0,99975746	-0,02620507	0,000105347
Q14157	0,982	0,99975746	-0,02620507	0,000105347
O95793	0,982	0,99975746	-0,02620507	0,000105347
Q32MZ4	0,982	0,99975746	-0,02620507	0,000105347
Q9HB07	0,982	0,99975746	-0,02620507	0,000105347
Q9NRG9	0,982	0,99975746	-0,02620507	0,000105347
P41227	0,982	0,99975746	-0,02620507	0,000105347
O60231	0,982	0,99975746	-0,02620507	0,000105347
P51948	0,982	0,99975746	-0,02620507	0,000105347
P61201	0,982	0,99975746	-0,02620507	0,000105347
P08238	0,983	0,99975746	-0,024736678	0,000105347
Q9UJZ1	0,983	0,99975746	-0,024736678	0,000105347
P51572	0,983	0,99975746	-0,024736678	0,000105347
Q9P287	0,983	0,99975746	-0,024736678	0,000105347
P36551	0,983	0,99975746	-0,024736678	0,000105347
O95219	0,983	0,99975746	-0,024736678	0,000105347
M0QXB5	0,983	0,99975746	-0,024736678	0,000105347
J3KRF8	0,983	0,99975746	-0,024736678	0,000105347
Q9H6S0	0,983	0,99975746	-0,024736678	0,000105347
Q8TAA9	0,983	0,99975746	-0,024736678	0,000105347
Q9UHB9	0,984	0,99975746	-0,023269779	0,000105347
Q16543	0,984	0,99975746	-0,023269779	0,000105347
Q12769	0,984	0,99975746	-0,023269779	0,000105347
H0YNH8	0,984	0,99975746	-0,023269779	0,000105347
Q9NW64	0,984	0,99975746	-0,023269779	0,000105347
O94832	0,984	0,99975746	-0,023269779	0,000105347
Q4G0J3	0,984	0,99975746	-0,023269779	0,000105347
A0A0A0MTH9	0,984	0,99975746	-0,023269779	0,000105347
H0YKT5	0,985	0,99975746	-0,02180437	0,000105347
O75663	0,985	0,99975746	-0,02180437	0,000105347
Q8N392	0,985	0,99975746	-0,02180437	0,000105347
P13716	0,985	0,99975746	-0,02180437	0,000105347
Q9UHW5	0,985	0,99975746	-0,02180437	0,000105347
P29401	0,986	0,99975746	-0,020340448	0,000105347
Q01813	0,986	0,99975746	-0,020340448	0,000105347
Q14152	0,986	0,99975746	-0,020340448	0,000105347
P62888	0,986	0,99975746	-0,020340448	0,000105347
Q8NFH4	0,986	0,99975746	-0,020340448	0,000105347
H3BV90	0,986	0,99975746	-0,020340448	0,000105347
O60232	0,986	0,99975746	-0,020340448	0,000105347
F6WIT2	0,986	0,99975746	-0,020340448	0,000105347

E9PF05	0,986	0,99975746	-0,020340448	0,000105347
Q9H490	0,986	0,99975746	-0,020340448	0,000105347
P54136	0,987	0,99975746	-0,01887801	0,000105347
P23921	0,987	0,99975746	-0,01887801	0,000105347
O95336	0,987	0,99975746	-0,01887801	0,000105347
Q9NX58	0,987	0,99975746	-0,01887801	0,000105347
Q969Z0	0,987	0,99975746	-0,01887801	0,000105347
Q86X29	0,987	0,99975746	-0,01887801	0,000105347
Q9Y3D6	0,987	0,99975746	-0,01887801	0,000105347
Q66K14	0,987	0,99975746	-0,01887801	0,000105347
Q9Y696	0,988	0,99975746	-0,017417053	0,000105347
Q9UBT2	0,988	0,99975746	-0,017417053	0,000105347
Q04837	0,988	0,99975746	-0,017417053	0,000105347
P49959	0,988	0,99975746	-0,017417053	0,000105347
P10768	0,988	0,99975746	-0,017417053	0,000105347
G5EA36	0,988	0,99975746	-0,017417053	0,000105347
O14737	0,988	0,99975746	-0,017417053	0,000105347
O14828	0,988	0,99975746	-0,017417053	0,000105347
Q96C23	0,988	0,99975746	-0,017417053	0,000105347
O43665	0,988	0,99975746	-0,017417053	0,000105347
A0A0C4DG33	0,988	0,99975746	-0,017417053	0,000105347
Q9NU22	0,989	0,99975746	-0,015957574	0,000105347
P49903	0,989	0,99975746	-0,015957574	0,000105347
P14678	0,989	0,99975746	-0,015957574	0,000105347
P09234	0,989	0,99975746	-0,015957574	0,000105347
P18075	0,989	0,99975746	-0,015957574	0,000105347
A0A2R8Y4J4	0,989	0,99975746	-0,015957574	0,000105347
Q15008	0,99	0,99975746	-0,01449957	0,000105347
P05388	0,99	0,99975746	-0,01449957	0,000105347
P49006	0,99	0,99975746	-0,01449957	0,000105347
O14562	0,99	0,99975746	-0,01449957	0,000105347
G3V158	0,99	0,99975746	-0,01449957	0,000105347
K7ELC7	0,99	0,99975746	-0,01449957	0,000105347
Q9Y2W2	0,99	0,99975746	-0,01449957	0,000105347
Q9NPA0	0,99	0,99975746	-0,01449957	0,000105347
O60573	0,99	0,99975746	-0,01449957	0,000105347
Q9H4A6	0,99	0,99975746	-0,01449957	0,000105347
Q96CP2	0,99	0,99975746	-0,01449957	0,000105347
Q96GQ7	0,991	0,99975746	-0,013043037	0,000105347
Q01085	0,991	0,99975746	-0,013043037	0,000105347
A0A494C1J1	0,991	0,99975746	-0,013043037	0,000105347
O43815	0,991	0,99975746	-0,013043037	0,000105347
O75937	0,991	0,99975746	-0,013043037	0,000105347
F8WF69	0,991	0,99975746	-0,013043037	0,000105347
Q9Y3A6	0,991	0,99975746	-0,013043037	0,000105347
H3BRS3	0,991	0,99975746	-0,013043037	0,000105347
Q8IZV5	0,991	0,99975746	-0,013043037	0,000105347

O43913	0,991	0,99975746	-0,013043037	0,000105347
Q9UQ80	0,992	0,99975746	-0,011587974	0,000105347
Q99613	0,992	0,99975746	-0,011587974	0,000105347
P52294	0,992	0,99975746	-0,011587974	0,000105347
H0YDU8	0,992	0,99975746	-0,011587974	0,000105347
P43897	0,992	0,99975746	-0,011587974	0,000105347
O95881	0,992	0,99975746	-0,011587974	0,000105347
C9JVN9	0,992	0,99975746	-0,011587974	0,000105347
Q8TD19	0,992	0,99975746	-0,011587974	0,000105347
O75165	0,992	0,99975746	-0,011587974	0,000105347
Q9UHR5	0,992	0,99975746	-0,011587974	0,000105347
Q8IZP0	0,992	0,99975746	-0,011587974	0,000105347
Q9BT09	0,992	0,99975746	-0,011587974	0,000105347
P16615	0,993	0,99975746	-0,010134377	0,000105347
P15170	0,993	0,99975746	-0,010134377	0,000105347
O14744	0,993	0,99975746	-0,010134377	0,000105347
A0A0A6YYL4	0,993	0,99975746	-0,010134377	0,000105347
Q9H173	0,993	0,99975746	-0,010134377	0,000105347
A3KN83	0,993	0,99975746	-0,010134377	0,000105347
Q5BJF2	0,993	0,99975746	-0,010134377	0,000105347
Q9BSJ8	0,994	0,99975746	-0,008682243	0,000105347
Q96C19	0,994	0,99975746	-0,008682243	0,000105347
Q9NXF1	0,994	0,99975746	-0,008682243	0,000105347
O43684	0,994	0,99975746	-0,008682243	0,000105347
P25398	0,994	0,99975746	-0,008682243	0,000105347
P30043	0,994	0,99975746	-0,008682243	0,000105347
P62857	0,994	0,99975746	-0,008682243	0,000105347
P42766	0,994	0,99975746	-0,008682243	0,000105347
Q9NVG8	0,994	0,99975746	-0,008682243	0,000105347
Q14126	0,995	0,99975746	-0,007231569	0,000105347
Q53GQ0	0,995	0,99975746	-0,007231569	0,000105347
Q5T760	0,995	0,99975746	-0,007231569	0,000105347
Q9UJX2	0,995	0,99975746	-0,007231569	0,000105347
G3V3E8	0,996	0,99975746	-0,005782353	0,000105347
Q9Y4P3	0,996	0,99975746	-0,005782353	0,000105347
Q9BT22	0,996	0,99975746	-0,005782353	0,000105347
P49585	0,996	0,99975746	-0,005782353	0,000105347
P35573	0,996	0,99975746	-0,005782353	0,000105347
Q13442	0,996	0,99975746	-0,005782353	0,000105347
Q9BVC6	0,996	0,99975746	-0,005782353	0,000105347
Q05513	0,996	0,99975746	-0,005782353	0,000105347
O60610	0,997	0,99975746	-0,00433459	0,000105347
Q9Y5P6	0,997	0,99975746	-0,00433459	0,000105347
P20340	0,997	0,99975746	-0,00433459	0,000105347
Q9BTD8	0,997	0,99975746	-0,00433459	0,000105347
B4E3T4	0,997	0,99975746	-0,00433459	0,000105347
I3L3X1	0,997	0,99975746	-0,00433459	0,000105347

P02751	0,998	0,998739524	-0,002888279	0,000547763
B4DDD6	0,998	0,99975746	-0,002888279	0,000105347
Q8NBJ5	0,998	0,99975746	-0,002888279	0,000105347
P52788	0,998	0,99975746	-0,002888279	0,000105347
P16152	0,998	0,99975746	-0,002888279	0,000105347
P33240	0,998	0,99975746	-0,002888279	0,000105347
B0QYK0	0,998	0,99975746	-0,002888279	0,000105347
Q9BVK6	0,998	0,99975746	-0,002888279	0,000105347
P49005	0,998	0,99975746	-0,002888279	0,000105347
Q9BQ67	0,998	0,99975746	-0,002888279	0,000105347
Q9UBT7	0,998	0,99975746	-0,002888279	0,000105347
Q96NB2	0,998	0,99975746	-0,002888279	0,000105347
A0A2R8Y5S7	0,999	0,997728988	-0,001443417	0,00098741
P35998	0,999	0,997728988	-0,001443417	0,00098741
O43776	0,999	0,999610105	-0,001443417	0,000169362
H0YN18	0,999	0,997728988	-0,001443417	0,00098741
Q9BRF8	0,999	0,99975746	-0,001443417	0,000105347
Q9NX46	0,999	0,99975746	-0,001443417	0,000105347
P53007	0,999	0,99975746	-0,001443417	0,000105347
K7EIN2	0,999	0,99975746	-0,001443417	0,000105347
Q15291	0,999	0,99975746	-0,001443417	0,000105347
Q6P6C2	0,999	0,99975746	-0,001443417	0,000105347
A0A087WUX8	0,999	0,99975746	-0,001443417	0,000105347
P05141	1	0,997663787	0	0,001015791
Q12792	1	0,99975746	0	0,000105347
Q92879	1	0,99975746	0	0,000105347
E7ESC6	1	0,99975746	0	0,000105347
P42892	1	0,99975746	0	0,000105347
A0A1W2PPS8	1	0,99975746	0	0,000105347
E7EN73	1	0,99975746	0	0,000105347
Q08J23	1,001	0,99975746	0,001441974	0,000105347
Q8WWM7	1,001	0,99975746	0,001441974	0,000105347
Q96P70	1,001	0,99975746	0,001441974	0,000105347
P60981	1,001	0,99576809	0,001441974	0,001841795
P48047	1,001	0,997663787	0,001441974	0,001015791
Q93052	1,001	0,99975746	0,001441974	0,000105347
Q08752	1,001	0,99975746	0,001441974	0,000105347
Q8TAQ2	1,001	0,99975746	0,001441974	0,000105347
O75616	1,001	0,99975746	0,001441974	0,000105347
A0A140T9R1	1,001	0,99975746	0,001441974	0,000105347
Q9NWU2	1,001	0,99975746	0,001441974	0,000105347
Q92890	1,001	0,99975746	0,001441974	0,000105347
P40926	1,002	0,99576809	0,002882509	0,001841795
Q15758	1,002	0,99975746	0,002882509	0,000105347
P49189	1,002	0,99975746	0,002882509	0,000105347
Q9Y450	1,002	0,99975746	0,002882509	0,000105347
Q8NBY1	1,002	0,99975746	0,002882509	0,000105347

Q53GS9	1,002	0,99975746	0,002882509	0,000105347
P30049	1,002	0,99975746	0,002882509	0,000105347
P18827	1,002	0,99975746	0,002882509	0,000105347
Q8WWV3	1,002	0,99975746	0,002882509	0,000105347
P50570	1,003	0,99975746	0,004321606	0,000105347
Q8N4C8	1,003	0,99975746	0,004321606	0,000105347
P50213	1,003	0,99975746	0,004321606	0,000105347
Q13505	1,003	0,99975746	0,004321606	0,000105347
Q15042	1,003	0,99975746	0,004321606	0,000105347
P62312	1,003	0,99975746	0,004321606	0,000105347
Q04917	1,004	0,994994976	0,005759269	0,002179112
P20042	1,004	0,994796716	0,005759269	0,002265657
Q8N163	1,004	0,99975746	0,005759269	0,000105347
P62280	1,004	0,995050555	0,005759269	0,002154854
O76031	1,004	0,99975746	0,005759269	0,000105347
Q9Y4W6	1,004	0,99975746	0,005759269	0,000105347
C9JBI3	1,004	0,99975746	0,005759269	0,000105347
Q96C86	1,004	0,99975746	0,005759269	0,000105347
A0A3B3ITX4	1,004	0,99975746	0,005759269	0,000105347
Q9BSJ2	1,004	0,99975746	0,005759269	0,000105347
Q06546	1,004	0,99975746	0,005759269	0,000105347
Q5VW32	1,004	0,99975746	0,005759269	0,000105347
Q9NRV9	1,005	0,99975746	0,007195501	0,000105347
A0A499FJY3	1,005	0,99975746	0,007195501	0,000105347
F8W7Q4	1,005	0,99975746	0,007195501	0,000105347
Q8TAT6	1,005	0,99975746	0,007195501	0,000105347
Q9NQA3	1,005	0,99975746	0,007195501	0,000105347
Q9NY64	1,005	0,99975746	0,007195501	0,000105347
E9PJH7	1,005	0,99975746	0,007195501	0,000105347
P26599	1,006	0,990890817	0,008630305	0,003974196
Q9NP58	1,006	0,99975746	0,008630305	0,000105347
Q9BTV4	1,006	0,99975746	0,008630305	0,000105347
Q9BVI4	1,006	0,99975746	0,008630305	0,000105347
A0A087WTU3	1,006	0,99975746	0,008630305	0,000105347
Q96FV9	1,006	0,99975746	0,008630305	0,000105347
Q5VWU8	1,006	0,99975746	0,008630305	0,000105347
Q9UJX5	1,006	0,99975746	0,008630305	0,000105347
K7ELL7	1,007	0,988584312	0,010063683	0,004986286
O95202	1,007	0,99975746	0,010063683	0,000105347
P62826	1,007	0,989308676	0,010063683	0,004668182
Q93008	1,007	0,99975746	0,010063683	0,000105347
Q96G03	1,007	0,99975746	0,010063683	0,000105347
Q9UIJ7	1,007	0,99975746	0,010063683	0,000105347
Q96G46	1,007	0,99975746	0,010063683	0,000105347
E7EW05	1,007	0,99975746	0,010063683	0,000105347
G5EA31	1,008	0,99975746	0,011495639	0,000105347
O14976	1,008	0,99975746	0,011495639	0,000105347

Q9UPN9	1,008	0,99975746	0,011495639	0,000105347
P16219	1,008	0,99975746	0,011495639	0,000105347
Q8NHP8	1,008	0,99975746	0,011495639	0,000105347
Q9NWS0	1,008	0,99975746	0,011495639	0,000105347
Q96A35	1,008	0,99975746	0,011495639	0,000105347
E9PHA2	1,009	0,99975746	0,012926174	0,000105347
Q5SZE1	1,01	0,99975746	0,014355293	0,000105347
C9JAZ1	1,01	0,99975746	0,014355293	0,000105347
Q9H061	1,01	0,99975746	0,014355293	0,000105347
Q13526	1,011	0,99975746	0,015782997	0,000105347
P78417	1,012	0,98118266	0,01720929	0,008250135
E9PDF6	1,012	0,99975746	0,01720929	0,000105347
O75347	1,012	0,98107407	0,01720929	0,008298203
P62906	1,012	0,982001767	0,01720929	0,007887731
M0R165	1,012	0,99975746	0,01720929	0,000105347
P54709	1,012	0,982454523	0,01720929	0,007687544
Q92599	1,012	0,99975746	0,01720929	0,000105347
Q96A49	1,012	0,99975746	0,01720929	0,000105347
O43290	1,012	0,99975746	0,01720929	0,000105347
Q9NZM3	1,012	0,99975746	0,01720929	0,000105347
A0A087WYN9	1,012	0,99975746	0,01720929	0,000105347
Q9BUB7	1,012	0,99975746	0,01720929	0,000105347
Q99523	1,012	0,99975746	0,01720929	0,000105347
Q86X76	1,012	0,99975746	0,01720929	0,000105347
C9JQS9	1,012	0,99975746	0,01720929	0,000105347
Q9UH99	1,012	0,99975746	0,01720929	0,000105347
Q99661	1,013	0,99975746	0,018634174	0,000105347
H3BVG0	1,013	0,99975746	0,018634174	0,000105347
P21291	1,013	0,99975746	0,018634174	0,000105347
P35269	1,013	0,99975746	0,018634174	0,000105347
Q9BXJ8	1,013	0,99975746	0,018634174	0,000105347
Q9NR19	1,013	0,99975746	0,018634174	0,000105347
Q8IYB7	1,013	0,99975746	0,018634174	0,000105347
P27695	1,014	0,98107407	0,020057652	0,008298203
A8MQ02	1,014	0,99975746	0,020057652	0,000105347
P50402	1,014	0,99975746	0,020057652	0,000105347
P36405	1,014	0,99975746	0,020057652	0,000105347
Q9BVJ6	1,014	0,99975746	0,020057652	0,000105347
P47985	1,014	0,99975746	0,020057652	0,000105347
E5RGR0	1,014	0,99975746	0,020057652	0,000105347
P35270	1,014	0,99975746	0,020057652	0,000105347
P20073	1,015	0,997728988	0,021479727	0,00098741
G3V0E4	1,015	0,99975746	0,021479727	0,000105347
O43148	1,015	0,99975746	0,021479727	0,000105347
Q9ULC4	1,015	0,99975746	0,021479727	0,000105347
Q05048	1,015	0,99975746	0,021479727	0,000105347
H0Y2W2	1,016	0,997663787	0,022900402	0,001015791

P46087	1,016	0,99975746	0,022900402	0,000105347
Q9BZG8	1,016	0,99975746	0,022900402	0,000105347
H0YLH3	1,016	0,99975746	0,022900402	0,000105347
A0A1P0AYU5	1,016	0,99975746	0,022900402	0,000105347
Q86X83	1,016	0,99975746	0,022900402	0,000105347
Q15046	1,017	0,98107407	0,024319679	0,008298203
P22061	1,017	0,99975746	0,024319679	0,000105347
P00492	1,017	0,98107407	0,024319679	0,008298203
Q10713	1,017	0,99975746	0,024319679	0,000105347
Q8NI27	1,017	0,99975746	0,024319679	0,000105347
Q7L5D6	1,017	0,99975746	0,024319679	0,000105347
Q5C9Z4	1,017	0,99975746	0,024319679	0,000105347
H3BUD2	1,017	0,99975746	0,024319679	0,000105347
Q9Y3D3	1,017	0,99975746	0,024319679	0,000105347
H0YBD2	1,017	0,99975746	0,024319679	0,000105347
D6REX3	1,018	0,98107407	0,025737561	0,008298203
Q6PKG0	1,018	0,99975746	0,025737561	0,000105347
E7EX17	1,018	0,98107407	0,025737561	0,008298203
P46778	1,018	0,98107407	0,025737561	0,008298203
P55145	1,018	0,99975746	0,025737561	0,000105347
Q86UE4	1,018	0,99975746	0,025737561	0,000105347
P62070	1,018	0,99975746	0,025737561	0,000105347
Q9NUJ1	1,018	0,99975746	0,025737561	0,000105347
Q9NUI1	1,018	0,99975746	0,025737561	0,000105347
O60841	1,019	0,98107407	0,027154052	0,008298203
P17812	1,019	0,98107407	0,027154052	0,008298203
Q8WW12	1,019	0,99975746	0,027154052	0,000105347
O75976	1,019	0,99975746	0,027154052	0,000105347
Q8WUA2	1,019	0,99975746	0,027154052	0,000105347
Q96H20	1,019	0,99975746	0,027154052	0,000105347
P27708	1,02	0,98107407	0,028569152	0,008298203
Q9Y5L0	1,02	0,99975746	0,028569152	0,000105347
Q13492	1,02	0,99975746	0,028569152	0,000105347
O76021	1,02	0,99975746	0,028569152	0,000105347
Q9BU89	1,02	0,99975746	0,028569152	0,000105347
Q8WXA9	1,02	0,99975746	0,028569152	0,000105347
P23528	1,021	0,98107407	0,029982866	0,008298203
Q9UNZ2	1,021	0,98107407	0,029982866	0,008298203
P31689	1,021	0,99975746	0,029982866	0,000105347
G3V3H3	1,021	0,99975746	0,029982866	0,000105347
Q14997	1,021	0,99975746	0,029982866	0,000105347
Q9NQR4	1,021	0,99975746	0,029982866	0,000105347
Q9Y5A9	1,021	0,99975746	0,029982866	0,000105347
Q6ZMI0	1,021	0,99975746	0,029982866	0,000105347
Q7Z5L9	1,021	0,99975746	0,029982866	0,000105347
P53384	1,021	0,99975746	0,029982866	0,000105347
P50613	1,021	0,99975746	0,029982866	0,000105347

Q86Y82	1,021	0,99975746	0,029982866	0,000105347
Q00534	1,022	0,98107407	0,031395196	0,008298203
P49916	1,022	0,99975746	0,031395196	0,000105347
Q9BTY2	1,022	0,99975746	0,031395196	0,000105347
P05386	1,022	0,98107407	0,031395196	0,008298203
Q9BUN8	1,022	0,99975746	0,031395196	0,000105347
Q9P2J5	1,023	0,978941397	0,032806145	0,009243306
Q07960	1,023	0,98107407	0,032806145	0,008298203
P06865	1,023	0,99975746	0,032806145	0,000105347
Q7Z460	1,023	0,99975746	0,032806145	0,000105347
P29353	1,023	0,99975746	0,032806145	0,000105347
Q9BZD4	1,023	0,99975746	0,032806145	0,000105347
P12236	1,024	0,99975746	0,034215715	0,000105347
Q8NE71	1,024	0,984721449	0,034215715	0,006686602
J3KS05	1,024	0,99975746	0,034215715	0,000105347
O75844	1,024	0,99975746	0,034215715	0,000105347
U3KPZ7	1,024	0,99975746	0,034215715	0,000105347
P54105	1,024	0,99975746	0,034215715	0,000105347
Q9Y6V7	1,024	0,99975746	0,034215715	0,000105347
Q9Y5S2	1,024	0,99975746	0,034215715	0,000105347
Q86W56	1,024	0,99975746	0,034215715	0,000105347
A0A499FIJ6	1,024	0,99975746	0,034215715	0,000105347
O43169	1,025	0,99975746	0,03562391	0,000105347
Q9BXW7	1,025	0,99975746	0,03562391	0,000105347
Q9NZJ7	1,025	0,99975746	0,03562391	0,000105347
Q15120	1,025	0,99975746	0,03562391	0,000105347
P54802	1,025	0,99975746	0,03562391	0,000105347
Q9BVL4	1,025	0,99975746	0,03562391	0,000105347
Q9HC35	1,026	0,975028964	0,037030731	0,010982483
Q9NR45	1,026	0,99975746	0,037030731	0,000105347
H0Y368	1,026	0,99975746	0,037030731	0,000105347
Q9UPN7	1,026	0,99975746	0,037030731	0,000105347
Q9BZI7	1,026	0,99975746	0,037030731	0,000105347
Q15650	1,026	0,99975746	0,037030731	0,000105347
O95251	1,026	0,99975746	0,037030731	0,000105347
P04040	1,027	0,998739524	0,038436182	0,000547763
Q16836	1,027	0,99975746	0,038436182	0,000105347
Q06124	1,027	0,99975746	0,038436182	0,000105347
M0R2B7	1,027	0,99975746	0,038436182	0,000105347
P49257	1,027	0,997728988	0,038436182	0,00098741
Q9BY43	1,027	0,99975746	0,038436182	0,000105347
K7ELS8	1,027	0,99975746	0,038436182	0,000105347
Q0VF96	1,027	0,99975746	0,038436182	0,000105347
P51795	1,027	0,998739524	0,038436182	0,000547763
Q96GX2	1,027	0,99975746	0,038436182	0,000105347
P07814	1,028	0,970944785	0,039840265	0,012805467
P62820	1,028	0,997728988	0,039840265	0,00098741

Q9NYU2	1,028	0,99975746	0,039840265	0,000105347
P22059	1,028	0,99975746	0,039840265	0,000105347
O14929	1,028	0,99975746	0,039840265	0,000105347
P16930	1,028	0,99975746	0,039840265	0,000105347
Q8N8N7	1,028	0,99975746	0,039840265	0,000105347
Q08211	1,029	0,9701579	0,041242982	0,013157576
Q15233	1,029	0,969647552	0,041242982	0,013386095
P12268	1,029	0,968506441	0,041242982	0,013897487
P84095	1,029	0,99975746	0,041242982	0,000105347
P78540	1,029	0,99576809	0,041242982	0,001841795
Q5JTY5	1,029	0,99975746	0,041242982	0,000105347
O60825	1,029	0,99975746	0,041242982	0,000105347
Q8IXI1	1,029	0,99975746	0,041242982	0,000105347
Q8WVP7	1,029	0,99975746	0,041242982	0,000105347
Q5T6F2	1,03	0,99975746	0,042644337	0,000105347
Q8N5M9	1,03	0,993217131	0,042644337	0,002955798
Q86U90	1,03	0,99975746	0,042644337	0,000105347
Q9H0U4	1,031	0,99975746	0,044044333	0,000105347
Q9NR50	1,031	0,99975746	0,044044333	0,000105347
Q8WX92	1,031	0,997663787	0,044044333	0,001015791
Q86YP4	1,031	0,994994976	0,044044333	0,002179112
O43709	1,031	0,99975746	0,044044333	0,000105347
P10253	1,031	0,99975746	0,044044333	0,000105347
P23246	1,032	0,966576772	0,045442971	0,014763646
Q9Y3I0	1,032	0,966576772	0,045442971	0,014763646
A2A274	1,032	0,966576772	0,045442971	0,014763646
P62314	1,032	0,98107407	0,045442971	0,008298203
P16278	1,032	0,997529861	0,045442971	0,001074095
Q3ZCQ8	1,032	0,997663787	0,045442971	0,001015791
I1E4Y6	1,032	0,99975746	0,045442971	0,000105347
Q32P28	1,032	0,99975746	0,045442971	0,000105347
Q14684	1,032	0,997663787	0,045442971	0,001015791
Q6P158	1,032	0,997663787	0,045442971	0,001015791
Q9HD15	1,032	0,997728988	0,045442971	0,00098741
Q8NG11	1,032	0,997663787	0,045442971	0,001015791
Q86Y39	1,032	0,99975746	0,045442971	0,000105347
X6R390	1,032	0,997728988	0,045442971	0,00098741
B8ZZW5	1,032	0,99975746	0,045442971	0,000105347
Q9H270	1,032	0,99975746	0,045442971	0,000105347
Q92598	1,033	0,965062406	0,046840254	0,015444602
P00491	1,033	0,99576809	0,046840254	0,001841795
Q9Y5X3	1,033	0,963520913	0,046840254	0,016138855
A0A494C169	1,033	0,99576809	0,046840254	0,001841795
Q8NFH5	1,033	0,99975746	0,046840254	0,000105347
Q5VU11	1,033	0,995050555	0,046840254	0,002154854
Q92692	1,033	0,993217131	0,046840254	0,002955798
Q9Y287	1,033	0,99975746	0,046840254	0,000105347

Q6P9B9	1,033	0,99975746	0,046840254	0,000105347
P38646	1,034	0,956904336	0,048236186	0,019131478
P14618	1,034	0,959874451	0,048236186	0,017785568
Q96IJ6	1,034	0,99975746	0,048236186	0,000105347
P30154	1,034	0,99975746	0,048236186	0,000105347
I3L0M4	1,034	0,99975746	0,048236186	0,000105347
P49459	1,034	0,99975746	0,048236186	0,000105347
Q9Y490	1,035	0,953455327	0,049630768	0,02069965
A0A0G2JN29	1,035	0,99975746	0,049630768	0,000105347
P61204	1,035	0,953455327	0,049630768	0,02069965
Q9UNF0	1,035	0,972482109	0,049630768	0,01211838
Q5SSJ5	1,035	0,99576809	0,049630768	0,001841795
Q9NQH7	1,035	0,99975746	0,049630768	0,000105347
Q9Y376	1,035	0,997728988	0,049630768	0,00098741
X6RLT1	1,035	0,998739524	0,049630768	0,000547763
Q8IWT0	1,035	0,99975746	0,049630768	0,000105347
X6R2S6	1,035	0,987126334	0,049630768	0,005627262
Q9NR09	1,036	0,99576809	0,051024003	0,001841795
O00193	1,036	0,99975746	0,051024003	0,000105347
O15347	1,036	0,99975746	0,051024003	0,000105347
Q96QU8	1,036	0,99975746	0,051024003	0,000105347
P68104	1,037	0,950648349	0,052415894	0,021980102
Q15717	1,037	0,952590825	0,052415894	0,021093606
P04632	1,037	0,993686847	0,052415894	0,002750459
C9JFR7	1,037	0,980310814	0,052415894	0,008636207
O15397	1,037	0,985306407	0,052415894	0,006428693
H0YNG3	1,037	0,999610105	0,052415894	0,000169362
O15511	1,037	0,997728988	0,052415894	0,00098741
Q8TB52	1,037	0,99975746	0,052415894	0,000105347
A0A1B0GUE3	1,037	0,99576809	0,052415894	0,001841795
Q9Y224	1,038	0,98107407	0,053806444	0,008298203
A0A0B4J1R4	1,038	0,997663787	0,053806444	0,001015791
P62310	1,038	0,997728988	0,053806444	0,00098741
Q92614	1,038	0,99975746	0,053806444	0,000105347
Q93050	1,038	0,99975746	0,053806444	0,000105347
Q9NQW7	1,039	0,944466098	0,055195654	0,024813627
P11908	1,039	0,99576809	0,055195654	0,001841795
Q96T76	1,039	0,99576809	0,055195654	0,001841795
Q9Y478	1,039	0,99975746	0,055195654	0,000105347
Q9UGR2	1,039	0,99975746	0,055195654	0,000105347
P26196	1,04	0,98107407	0,056583528	0,008298203
Q92930	1,04	0,99975746	0,056583528	0,000105347
F6Y5H0	1,04	0,99975746	0,056583528	0,000105347
P49757	1,04	0,99975746	0,056583528	0,000105347
Q9Y679	1,04	0,997728988	0,056583528	0,00098741
P49756	1,041	0,99576809	0,057970069	0,001841795
P48147	1,041	0,986673427	0,057970069	0,005826568

O75330	1,041	0,997728988	0,057970069	0,00098741
U3KQS2	1,041	0,99975746	0,057970069	0,000105347
Q9H974	1,041	0,99975746	0,057970069	0,000105347
Q5W0H4	1,042	0,931826238	0,059355278	0,030665065
P09417	1,042	0,989308676	0,059355278	0,004668182
Q96S44	1,042	0,997663787	0,059355278	0,001015791
P68402	1,042	0,997663787	0,059355278	0,001015791
P36543	1,042	0,99576809	0,059355278	0,001841795
Q99614	1,042	0,997663787	0,059355278	0,001015791
P14868	1,043	0,927921611	0,060739158	0,032488711
P21127	1,043	0,99576809	0,060739158	0,001841795
Q9H6Z4	1,043	0,994994976	0,060739158	0,002179112
A8MXP9	1,044	0,924143221	0,062121712	0,034260718
P62316	1,044	0,923139408	0,062121712	0,034732709
Q9H3G5	1,044	0,98107407	0,062121712	0,008298203
P49247	1,044	0,99576809	0,062121712	0,001841795
Q99797	1,044	0,994356943	0,062121712	0,002457689
P53350	1,044	0,99576809	0,062121712	0,001841795
Q9NP97	1,044	0,994994976	0,062121712	0,002179112
Q9H857	1,044	0,99975746	0,062121712	0,000105347
Q7Z3K3	1,044	0,99975746	0,062121712	0,000105347
Q9BRS2	1,044	0,99975746	0,062121712	0,000105347
Q13200	1,045	0,921636345	0,063502942	0,035440407
O00264	1,045	0,99576809	0,063502942	0,001841795
P40616	1,045	0,99576809	0,063502942	0,001841795
Q5T440	1,045	0,997663787	0,063502942	0,001015791
P37837	1,046	0,919602635	0,064882852	0,036399793
A0A286YFF7	1,046	0,991871097	0,064882852	0,003544765
P58546	1,046	0,994994976	0,064882852	0,002179112
Q13011	1,046	0,991996939	0,064882852	0,003489668
O43837	1,046	0,984617738	0,064882852	0,006732345
Q9UN81	1,046	0,993217131	0,064882852	0,002955798
Q6UWE0	1,046	0,98107407	0,064882852	0,008298203
P53365	1,046	0,99975746	0,064882852	0,000105347
A0A0G2JIW1	1,047	0,98107407	0,066261442	0,008298203
Q16891	1,047	0,99576809	0,066261442	0,001841795
Q13404	1,047	0,994994976	0,066261442	0,002179112
A0A024RCR6	1,047	0,976922891	0,066261442	0,010139714
O00443	1,047	0,989308676	0,066261442	0,004668182
O60684	1,047	0,983972189	0,066261442	0,007017176
Q9GZP4	1,047	0,983259031	0,066261442	0,007332056
Q9BSH5	1,047	0,99975746	0,066261442	0,000105347
O14495	1,047	0,99975746	0,066261442	0,000105347
P23284	1,048	0,914210182	0,067638717	0,038953946
O75717	1,048	0,987848359	0,067638717	0,005309717
A6NHX0	1,048	0,991871097	0,067638717	0,003544765
Q96Q11	1,048	0,98107407	0,067638717	0,008298203

F5GYQ1	1,048	0,98107407	0,067638717	0,008298203
M0R1T5	1,048	0,984617738	0,067638717	0,006732345
Q92616	1,049	0,910887098	0,069014678	0,040535449
Q9UPN3	1,049	0,999667339	0,069014678	0,000144497
K7EKE6	1,049	0,910567896	0,069014678	0,040687666
Q13363	1,049	0,983972189	0,069014678	0,007017176
P69905	1,049	0,98107407	0,069014678	0,008298203
P06132	1,049	0,993374613	0,069014678	0,002886943
Q86VM9	1,049	0,99975746	0,069014678	0,000105347
P22314	1,05	0,902607717	0,070389328	0,044500958
Q8WXX5	1,05	0,994356943	0,070389328	0,002457689
Q53H82	1,05	0,983532445	0,070389328	0,007211309
P31946	1,051	0,902383185	0,071762669	0,044609006
P98082	1,051	0,985132519	0,071762669	0,006505345
A0A087WXI5	1,051	0,990890817	0,071762669	0,003974196
F5H658	1,051	0,99975746	0,071762669	0,000105347
Q9H089	1,051	0,989308676	0,071762669	0,004668182
P09104	1,052	0,983972189	0,073134705	0,007017176
M0R2N5	1,052	0,979469477	0,073134705	0,009009093
Q9UI30	1,052	0,983972189	0,073134705	0,007017176
Q9GZM5	1,052	0,99975746	0,073134705	0,000105347
Q9BTU6	1,052	0,99975746	0,073134705	0,000105347
P26038	1,053	0,891757309	0,074505436	0,049753322
Q7LBC6	1,053	0,988813095	0,074505436	0,004885791
O43583	1,053	0,983972189	0,074505436	0,007017176
Q9NXE4	1,053	0,98107407	0,074505436	0,008298203
O95295	1,053	0,99975746	0,074505436	0,000105347
Q9BRG1	1,053	0,988907502	0,074505436	0,004844328
Q86UP2	1,054	0,887423404	0,075874867	0,051869122
Q99714	1,054	0,886368398	0,075874867	0,052385736
Q99497	1,054	0,888435404	0,075874867	0,051374143
P55735	1,054	0,98107407	0,075874867	0,008298203
O14579	1,054	0,984617738	0,075874867	0,006732345
O96019	1,054	0,983972189	0,075874867	0,007017176
Q9Y570	1,054	0,985132519	0,075874867	0,006505345
Q9BSR8	1,054	0,993217131	0,075874867	0,002955798
P11940	1,055	0,884483355	0,077242999	0,053310336
Q13310	1,055	0,882352456	0,077242999	0,054357901
E7EVA0	1,055	0,884573246	0,077242999	0,0532662
P11216	1,055	0,976922891	0,077242999	0,010139714
Q96RS6	1,055	0,984617738	0,077242999	0,006732345
P31948	1,056	0,879153259	0,078609835	0,05593541
P26640	1,056	0,953417031	0,078609835	0,020717094
Q14739	1,056	0,98107407	0,078609835	0,008298203
O76071	1,056	0,98107407	0,078609835	0,008298203
Q96SQ9	1,056	0,98107407	0,078609835	0,008298203
Q10471	1,056	0,984702991	0,078609835	0,006694743

Q8N2G8	1,056	0,997663787	0,078609835	0,001015791
A0A0D9SGE8	1,056	0,99975746	0,078609835	0,000105347
Q16851	1,057	0,875844843	0,079975377	0,057572823
Q14008	1,057	0,875839436	0,079975377	0,057575504
O00154	1,057	0,874220393	0,079975377	0,058379067
Q5TFE4	1,057	0,98107407	0,079975377	0,008298203
Q13619	1,057	0,981573982	0,079975377	0,008076962
Q53EL6	1,057	0,99576809	0,079975377	0,001841795
M0QYV0	1,057	0,997663787	0,079975377	0,001015791
A0A0C4DG89	1,058	0,966576772	0,081339627	0,014763646
Q9Y2T2	1,058	0,983972189	0,081339627	0,007017176
Q9H1Y0	1,058	0,989308676	0,081339627	0,004668182
P11441	1,058	0,98262426	0,081339627	0,007612518
P15291	1,059	0,98107407	0,082702589	0,008298203
O95376	1,059	0,997663787	0,082702589	0,001015791
P09972	1,06	0,99576809	0,084064265	0,001841795
Q9HCU5	1,06	0,98107407	0,084064265	0,008298203
C9JVE2	1,06	0,98107407	0,084064265	0,008298203
Q86TB9	1,06	0,98107407	0,084064265	0,008298203
Q96T51	1,06	0,99975746	0,084064265	0,000105347
H7C488	1,06	0,99975746	0,084064265	0,000105347
O14672	1,061	0,979469477	0,085424656	0,009009093
Q13151	1,062	0,953417031	0,086783766	0,020717094
G3V325	1,062	0,979469477	0,086783766	0,009009093
O75691	1,062	0,982029419	0,086783766	0,007875502
P61009	1,062	0,98107407	0,086783766	0,008298203
Q9NSI2	1,062	0,98107407	0,086783766	0,008298203
Q9H7D7	1,062	0,99975746	0,086783766	0,000105347
P20936	1,062	0,99975746	0,086783766	0,000105347
Q9UG63	1,063	0,98107407	0,088141597	0,008298203
Q9HAV7	1,063	0,979469477	0,088141597	0,009009093
P61006	1,063	0,98107407	0,088141597	0,008298203
Q9BQA1	1,063	0,98107407	0,088141597	0,008298203
Q9Y320	1,063	0,98107407	0,088141597	0,008298203
Q5HYK3	1,063	0,998739524	0,088141597	0,000547763
O75494	1,063	0,98107407	0,088141597	0,008298203
P02538	1,064	0,98107407	0,089498151	0,008298203
P31943	1,064	0,863158491	0,089498151	0,063909453
O00151	1,064	0,863661049	0,089498151	0,063656667
Q12996	1,064	0,98107407	0,089498151	0,008298203
Q15819	1,064	0,98107407	0,089498151	0,008298203
G8JLB3	1,064	0,98107407	0,089498151	0,008298203
H3BN98	1,064	0,98107407	0,089498151	0,008298203
Q8N5A5	1,064	0,989308676	0,089498151	0,004668182
A0A0C4DFX9	1,064	0,98107407	0,089498151	0,008298203
P48729	1,064	0,98107407	0,089498151	0,008298203
P14859	1,064	0,99975746	0,089498151	0,000105347

O43598	1,064	0,98107407	0,089498151	0,008298203
F8WBJ6	1,064	0,99975746	0,089498151	0,000105347
P53618	1,065	0,859244314	0,09085343	0,065883333
Q92973	1,065	0,86675967	0,09085343	0,062101304
P50995	1,065	0,859244314	0,09085343	0,065883333
P49755	1,065	0,859244314	0,09085343	0,065883333
P15121	1,065	0,864155777	0,09085343	0,063407962
C9J931	1,065	0,98107407	0,09085343	0,008298203
Q1RLN5	1,065	0,993648498	0,09085343	0,00276722
Q9P000	1,065	0,99576809	0,09085343	0,001841795
Q9H3S7	1,066	0,979469477	0,092207438	0,009009093
O00159	1,066	0,85777103	0,092207438	0,066628626
O14617	1,066	0,98107407	0,092207438	0,008298203
E9PC15	1,066	0,98107407	0,092207438	0,008298203
P46977	1,067	0,857696166	0,093560176	0,066666531
F6TLX2	1,067	0,970020736	0,093560176	0,013218982
O15498	1,067	0,98107407	0,093560176	0,008298203
E7EWW0	1,067	0,993217131	0,093560176	0,002955798
D6RGZ2	1,067	0,98107407	0,093560176	0,008298203
Q9BU76	1,067	0,99975746	0,093560176	0,000105347
P31751	1,067	0,99975746	0,093560176	0,000105347
Q13045	1,068	0,98107407	0,094911647	0,008298203
Q9H845	1,068	0,958500914	0,094911647	0,018407469
F8W7U3	1,068	0,975028964	0,094911647	0,010982483
Q9HCN8	1,068	0,988206852	0,094911647	0,005152139
Q9H269	1,068	0,99576809	0,094911647	0,001841795
Q3ZCW2	1,068	0,98107407	0,094911647	0,008298203
F8W9X7	1,068	0,99975746	0,094911647	0,000105347
P35221	1,069	0,848455014	0,096261853	0,071371179
O60506	1,069	0,847463733	0,096261853	0,071878878
O60493	1,069	0,98107407	0,096261853	0,008298203
Q9NX40	1,069	0,98107407	0,096261853	0,008298203
A0AV96	1,07	0,98107407	0,097610797	0,008298203
Q9UBU9	1,07	0,98107407	0,097610797	0,008298203
Q92522	1,07	0,98107407	0,097610797	0,008298203
Q14847	1,071	0,849558593	0,09895848	0,070806663
P48634	1,071	0,999610105	0,09895848	0,000169362
A0A0A0MS54	1,071	0,975028964	0,09895848	0,010982483
Q96C36	1,072	0,976429171	0,100304906	0,010359254
O00764	1,072	0,97920882	0,100304906	0,009124683
D6RF35	1,072	0,99576809	0,100304906	0,001841795
Q9UBB6	1,072	0,99975746	0,100304906	0,000105347
A0A0C4DFM1	1,073	0,975028964	0,101650076	0,010982483
O75530	1,073	0,98107407	0,101650076	0,008298203
P08621	1,074	0,836490328	0,102993993	0,077539076
O00203	1,074	0,976625944	0,102993993	0,010271743
Q14376	1,074	0,977366442	0,102993993	0,009942577

A0A494C0W0	1,074	0,98107407	0,102993993	0,008298203
Q5QNY5	1,074	0,997663787	0,102993993	0,001015791
P12955	1,075	0,882352871	0,10433666	0,054357697
Q9Y446	1,075	0,975028964	0,10433666	0,010982483
P60953	1,075	0,96473541	0,10433666	0,015591781
Q9H501	1,075	0,99576809	0,10433666	0,001841795
Q9Y5A7	1,075	0,997663787	0,10433666	0,001015791
E7EQ64	1,076	0,840972356	0,105678078	0,07521828
F5H157	1,076	0,979469477	0,105678078	0,009009093
Q9BXB5	1,076	0,98107407	0,105678078	0,008298203
Q9UKM7	1,076	0,998739524	0,105678078	0,000547763
P51148	1,077	0,932509755	0,10701825	0,030346616
Q14677	1,077	0,976211495	0,10701825	0,010456083
F8VXU5	1,077	0,970944785	0,10701825	0,012805467
A0A087X1X9	1,077	0,99576809	0,10701825	0,001841795
J3KTL2	1,078	0,818779003	0,108357178	0,086833303
Q9NTI5	1,078	0,971417048	0,108357178	0,012594279
Q9UJW0	1,078	0,979469477	0,108357178	0,009009093
G5E994	1,078	0,993648498	0,108357178	0,00276722
P60709	1,079	0,813831176	0,109694865	0,089465677
P48444	1,079	0,814186997	0,109694865	0,089275838
Q9UL25	1,079	0,971417048	0,109694865	0,012594279
Q08431	1,079	0,971417048	0,109694865	0,012594279
Q9BXB4	1,079	0,99576809	0,109694865	0,001841795
A0A087X0L7	1,079	0,981750145	0,109694865	0,007999026
Q8WUM4	1,08	0,813831176	0,111031312	0,089465677
Q86V48	1,08	0,970994089	0,111031312	0,012783414
Q6P996	1,08	0,966576772	0,111031312	0,014763646
Q96RP9	1,08	0,942246997	0,111031312	0,025835238
Q9BV38	1,08	0,966576772	0,111031312	0,014763646
Q5JTZ9	1,08	0,998954077	0,111031312	0,000454476
A0A0D9SGD3	1,08	0,983972189	0,111031312	0,007017176
Q9BSE5	1,081	0,918007555	0,112366523	0,037153745
Q92520	1,081	0,972482109	0,112366523	0,01211838
P98179	1,081	0,966576772	0,112366523	0,014763646
Q92917	1,081	0,98107407	0,112366523	0,008298203
G3V3R7	1,081	0,982541782	0,112366523	0,007648972
P46459	1,082	0,918007555	0,113700499	0,037153745
O14964	1,082	0,953455327	0,113700499	0,02069965
O75152	1,082	0,98107407	0,113700499	0,008298203
A0A0B4J2A0	1,082	0,98107407	0,113700499	0,008298203
Q9BXK5	1,082	0,974552177	0,113700499	0,011194904
P52298	1,082	0,975348173	0,113700499	0,010840325
J3QL56	1,082	0,971417048	0,113700499	0,012594279
Q9Y678	1,083	0,797716633	0,115033243	0,098151352
P40925	1,083	0,797716633	0,115033243	0,098151352
Q9H8Y8	1,083	0,958719392	0,115033243	0,018308488

Q99805	1,083	0,956511327	0,115033243	0,019309883
Q13724	1,083	0,967942813	0,115033243	0,0141503
Q8WVV9	1,083	0,98107407	0,115033243	0,008298203
Q13895	1,083	0,959860926	0,115033243	0,017791687
B5MDQ0	1,083	0,967041651	0,115033243	0,01455482
P48735	1,084	0,795911883	0,116364757	0,099135011
O60749	1,084	0,872895766	0,116364757	0,059037613
Q6P2E9	1,084	0,971417048	0,116364757	0,012594279
O14681	1,084	0,966576772	0,116364757	0,014763646
A0A075B797	1,084	0,99576809	0,116364757	0,001841795
Q9NUD5	1,084	0,99576809	0,116364757	0,001841795
P47755	1,085	0,846484044	0,117695043	0,072381224
D6REB4	1,085	0,971417048	0,117695043	0,012594279
Q13523	1,085	0,985538678	0,117695043	0,006326327
P05534	1,085	0,98107407	0,117695043	0,008298203
O43617	1,085	0,953001598	0,117695043	0,020906371
P52209	1,086	0,790944349	0,119024103	0,101854072
P30622	1,086	0,956255699	0,119024103	0,019425964
O95747	1,086	0,966576772	0,119024103	0,014763646
Q13501	1,086	0,942246997	0,119024103	0,025835238
Q8IYB8	1,086	0,944898897	0,119024103	0,024614658
Q16555	1,087	0,784848556	0,12035194	0,105214136
P30519	1,087	0,827876692	0,12035194	0,082034344
P08754	1,087	0,868154207	0,12035194	0,061403126
Q9ULT8	1,087	0,98107407	0,12035194	0,008298203
P36507	1,087	0,98107407	0,12035194	0,008298203
C9J1S9	1,087	0,98107407	0,12035194	0,008298203
O75794	1,087	0,98107407	0,12035194	0,008298203
Q92665	1,088	0,953001598	0,121678557	0,020906371
Q16629	1,089	0,944882686	0,123003954	0,024622109
P14635	1,089	0,961351224	0,123003954	0,017117916
A0A0A0MQR2	1,089	0,966576772	0,123003954	0,014763646
Q9BV86	1,089	0,966576772	0,123003954	0,014763646
P62304	1,089	0,950116606	0,123003954	0,022223091
A0A3B3IUA2	1,089	0,966576772	0,123003954	0,014763646
A0A087WY61	1,09	0,953001598	0,124328135	0,020906371
Q96K17	1,09	0,942246997	0,124328135	0,025835238
P53582	1,09	0,98107407	0,124328135	0,008298203
H0Y9B0	1,09	0,98107407	0,124328135	0,008298203
P57772	1,09	0,989353654	0,124328135	0,004648438
A8MPP1	1,09	0,985132519	0,124328135	0,006505345
A0A2U3TZL8	1,091	0,954250728	0,125651102	0,0203375
G5E9X5	1,091	0,973042274	0,125651102	0,011868291
Q9BZG1	1,091	0,98107407	0,125651102	0,008298203
O95396	1,091	0,986146592	0,125651102	0,006058522
P54886	1,092	0,763013206	0,126972856	0,117467945
P06737	1,092	0,910784479	0,126972856	0,040584379

P48637	1,092	0,819615116	0,126972856	0,086390041
Q6P3X3	1,092	0,965062406	0,126972856	0,015444602
Q9H9A6	1,092	0,937940047	0,126972856	0,027824921
Q92896	1,092	0,966576772	0,126972856	0,014763646
A0A0A0MTC6	1,092	0,997663787	0,126972856	0,001015791
Q92878	1,093	0,947591131	0,128293401	0,023379013
P49770	1,093	0,864155777	0,128293401	0,063407962
Q9BY32	1,093	0,937471816	0,128293401	0,02804178
Q9NVH1	1,093	0,956255699	0,128293401	0,019425964
O60427	1,093	0,988206852	0,128293401	0,005152139
Q86X55	1,094	0,945821355	0,129612738	0,024190885
A0A2R8YHB3	1,094	0,927921611	0,129612738	0,032488711
P04066	1,094	0,98107407	0,129612738	0,008298203
F8VXI9	1,094	0,990891563	0,129612738	0,003973869
P13804	1,095	0,756167518	0,13093087	0,121381982
Q96AG4	1,095	0,758435098	0,13093087	0,120081577
O75947	1,095	0,909973092	0,13093087	0,04097145
Q9BV44	1,095	0,947061197	0,13093087	0,023621957
J3KNK1	1,095	0,98107407	0,13093087	0,008298203
H0YFD6	1,096	0,753973257	0,132247798	0,122644058
O75822	1,096	0,933155011	0,132247798	0,030046207
Q8IWJ2	1,096	0,98107407	0,132247798	0,008298203
Q13630	1,096	0,952590825	0,132247798	0,021093606
Q9H3K2	1,096	0,953859691	0,132247798	0,020515504
Q8NFW8	1,096	0,969792804	0,132247798	0,013321043
Q8WUF5	1,096	0,986673427	0,132247798	0,005826568
Q96S66	1,097	0,980965309	0,133563526	0,008346351
Q5JVF3	1,097	0,938847409	0,133563526	0,027404988
Q9NWT1	1,097	0,98107407	0,133563526	0,008298203
Q9P2E9	1,098	0,748449948	0,134878054	0,125837237
P51114	1,098	0,863661049	0,134878054	0,063656667
Q5JRX3	1,098	0,864155777	0,134878054	0,063407962
Q9P035	1,098	0,827921683	0,134878054	0,082010743
O43172	1,098	0,953859691	0,134878054	0,020515504
Q15629	1,098	0,953001598	0,134878054	0,020906371
P11717	1,099	0,857696166	0,136191386	0,066666531
Q9Y5Z4	1,099	0,950250893	0,136191386	0,022161714
Q8N684	1,099	0,921750842	0,136191386	0,035386457
Q96DG6	1,1	0,86458142	0,137503524	0,063194102
P31942	1,1	0,928986771	0,137503524	0,03199047
Q6UW68	1,1	0,928012054	0,137503524	0,032446383
Q92597	1,1	0,984617738	0,137503524	0,006732345
Q5T5C7	1,101	0,731105322	0,138814469	0,136020055
Q9Y624	1,101	0,791129771	0,138814469	0,101752272
O95394	1,101	0,859244314	0,138814469	0,065883333
A0A087X0R6	1,101	0,917460894	0,138814469	0,037412438
P18615	1,101	0,976922891	0,138814469	0,010139714

O43293	1,101	0,967041651	0,138814469	0,01455482
P48059	1,101	0,953001598	0,138814469	0,020906371
O94782	1,101	0,984617738	0,138814469	0,006732345
P38159	1,102	0,921636345	0,140124224	0,035440407
Q15363	1,102	0,919602635	0,140124224	0,036399793
F1T0I1	1,102	0,962859605	0,140124224	0,016437033
Q14139	1,102	0,944898897	0,140124224	0,024614658
O75886	1,102	0,966576772	0,140124224	0,014763646
Q14674	1,102	0,988779196	0,140124224	0,00490068
Q9Y6M1	1,103	0,928012054	0,141432791	0,032446383
A0A0C4DFL7	1,103	0,917032482	0,141432791	0,037615281
C9JWV9	1,103	0,98107407	0,141432791	0,008298203
Q9BRX8	1,104	0,864105506	0,142740172	0,063433228
Q14C86	1,104	0,944996516	0,142740172	0,024569793
H3BQZ7	1,104	0,944466098	0,142740172	0,024813627
A0A3B3ITW1	1,104	0,979862581	0,142740172	0,008834827
Q9ULV0	1,104	0,976211495	0,142740172	0,010456083
Q12765	1,105	0,931826238	0,14404637	0,030665065
Q8IWB7	1,105	0,937940047	0,14404637	0,027824921
M0QY97	1,105	0,985132519	0,14404637	0,006505345
O43663	1,105	0,98107407	0,14404637	0,008298203
Q14320	1,106	0,766992332	0,145351386	0,115208978
P21399	1,106	0,924917367	0,145351386	0,033897066
P51571	1,106	0,87148065	0,145351386	0,059742251
P46060	1,107	0,703930733	0,146655222	0,152470073
P61158	1,107	0,729672938	0,146655222	0,13687176
P49902	1,107	0,885291459	0,146655222	0,052913726
P84103	1,107	0,76669659	0,146655222	0,115376468
Q9ULR3	1,107	0,921636345	0,146655222	0,035440407
P35637	1,108	0,703930733	0,147957881	0,152470073
P61923	1,108	0,895526137	0,147957881	0,047921734
O60547	1,108	0,932509755	0,147957881	0,030346616
Q9P2Y4	1,108	0,98107407	0,147957881	0,008298203
P53621	1,109	0,693859784	0,149259365	0,158728283
Q6IBS0	1,109	0,857696166	0,149259365	0,066666531
A6NFX8	1,109	0,812115006	0,149259365	0,090382465
Q01082	1,11	0,688593093	0,150559677	0,162037338
P61106	1,11	0,819984844	0,150559677	0,086194175
Q7KZ85	1,11	0,917032482	0,150559677	0,037615281
Q96N66	1,11	0,910784479	0,150559677	0,040584379
P13984	1,11	0,970994089	0,150559677	0,012783414
P24752	1,111	0,806368779	0,151858817	0,093466296
Q13617	1,111	0,895526137	0,151858817	0,047921734
H0Y517	1,111	0,685520056	0,151858817	0,163979835
Q53F19	1,111	0,9822835	0,151858817	0,007763151
Q712K3	1,111	0,968506441	0,151858817	0,013897487
O76003	1,112	0,683260797	0,153156788	0,165413497

P35610	1,112	0,970616678	0,153156788	0,012952251
A0A2R8YE10	1,112	0,945821355	0,153156788	0,024190885
Q13769	1,112	0,967041651	0,153156788	0,01455482
P55084	1,113	0,676632308	0,154453593	0,169647269
P27694	1,113	0,846484044	0,154453593	0,072381224
P84085	1,113	0,883605373	0,154453593	0,053741652
Q9NRP0	1,113	0,889009183	0,154453593	0,051093753
E9PPY3	1,113	0,983972189	0,154453593	0,007017176
Q9HBM0	1,113	0,98107407	0,154453593	0,008298203
V9GYM8	1,113	0,98107407	0,154453593	0,008298203
P14923	1,114	0,795613989	0,155749233	0,099297589
E9PEX6	1,114	0,674764721	0,155749233	0,170847632
B1AHL2	1,114	0,944466098	0,155749233	0,024813627
Q15102	1,114	0,911166882	0,155749233	0,040402074
P22626	1,115	0,669966949	0,15704371	0,173946622
O00429	1,115	0,966576772	0,15704371	0,014763646
Q9H0A0	1,115	0,898394174	0,15704371	0,046533073
Q9Y5V3	1,115	0,938663608	0,15704371	0,027490019
Q9Y6D6	1,115	0,984617738	0,15704371	0,006732345
A0A0A6YYA0	1,115	0,872895766	0,15704371	0,059037613
Q9NZW5	1,115	0,909810317	0,15704371	0,041049143
L0R6Q1	1,115	0,98107407	0,15704371	0,008298203
P25391	1,115	0,98107407	0,15704371	0,008298203
O00273	1,116	0,883082383	0,158337027	0,053998779
Q9BZE9	1,116	0,971417048	0,158337027	0,012594279
O43896	1,117	0,98107407	0,159629186	0,008298203
Q9Y3C8	1,117	0,902662281	0,159629186	0,044474705
P47897	1,118	0,654316423	0,160920188	0,184212179
P42224	1,118	0,857696166	0,160920188	0,066666531
Q09161	1,118	0,869231354	0,160920188	0,060864617
Q16706	1,118	0,895526137	0,160920188	0,047921734
Q9H993	1,118	0,883605373	0,160920188	0,053741652
P16157	1,118	0,976570387	0,160920188	0,010296449
Q5JRA6	1,118	0,945821355	0,160920188	0,024190885
O60763	1,119	0,762148929	0,162210036	0,117960156
Q86TI2	1,119	0,918276617	0,162210036	0,037026474
Q96I24	1,12	0,872895766	0,163498732	0,059037613
Q9BR76	1,12	0,864155777	0,163498732	0,063407962
Q9UBB4	1,12	0,872895766	0,163498732	0,059037613
P04080	1,12	0,64116843	0,163498732	0,19302787
Q9ULW0	1,12	0,88330367	0,163498732	0,053889965
B1AJY5	1,12	0,872895766	0,163498732	0,059037613
Q14344	1,12	0,911797393	0,163498732	0,040101654
O14975	1,12	0,885081154	0,163498732	0,053016907
H7BYY1	1,121	0,916623473	0,164786278	0,037809025
Q9H2J4	1,121	0,87148065	0,164786278	0,059742251
Q9Y6G5	1,121	0,965062406	0,164786278	0,015444602

Q6VY07	1,121	0,962189362	0,164786278	0,016739449
J3KNI1	1,121	0,98107407	0,164786278	0,008298203
P24666	1,122	0,895526137	0,166072676	0,047921734
P13674	1,123	0,902069139	0,167357928	0,044760175
Q15014	1,123	0,87148065	0,167357928	0,059742251
Q9NYL9	1,123	0,902231393	0,167357928	0,044682066
V9GYU0	1,123	0,98107407	0,167357928	0,008298203
B4DY09	1,124	0,623636183	0,168642036	0,205068695
Q14554	1,124	0,869906802	0,168642036	0,060527273
Q96AY3	1,124	0,847463733	0,168642036	0,071878878
Q32P41	1,124	0,974363993	0,168642036	0,011278773
E7EVC7	1,124	0,954250728	0,168642036	0,0203375
O43765	1,125	0,895526137	0,169925001	0,047921734
B4DEE8	1,125	0,864155777	0,169925001	0,063407962
A0A3B3IS71	1,125	0,919602635	0,169925001	0,036399793
Q9BQ95	1,125	0,98107407	0,169925001	0,008298203
A0A2R8Y880	1,125	0,98107407	0,169925001	0,008298203
P63104	1,126	0,612038481	0,171206827	0,213221271
Q8N3C0	1,126	0,879471571	0,171206827	0,055778195
P05186	1,126	0,98107407	0,171206827	0,008298203
Q6NSJ2	1,126	0,98107407	0,171206827	0,008298203
P35579	1,127	0,610213782	0,172487516	0,214517988
P00505	1,127	0,610387164	0,172487516	0,214394608
P62873	1,127	0,864155777	0,172487516	0,063407962
E9PP16	1,127	0,864155777	0,172487516	0,063407962
Q6GMV3	1,127	0,863661049	0,172487516	0,063656667
Q8NBX0	1,127	0,864155777	0,172487516	0,063407962
P19823	1,127	0,900668307	0,172487516	0,045435119
Q15126	1,127	0,98107407	0,172487516	0,008298203
P09651	1,128	0,606334486	0,173767068	0,21728773
O14950	1,128	0,606334486	0,173767068	0,21728773
O15173	1,128	0,863661049	0,173767068	0,063656667
Q86UV5	1,128	0,926035667	0,173767068	0,033372286
O75190	1,128	0,865306643	0,173767068	0,062829962
A0A1W2PQ51	1,129	0,675667732	0,175045486	0,170266821
O43390	1,129	0,606334486	0,175045486	0,21728773
J3KN67	1,129	0,85777103	0,175045486	0,066628626
P0DMN0	1,129	0,864105506	0,175045486	0,063433228
P18754	1,129	0,87148065	0,175045486	0,059742251
Q16512	1,129	0,864105506	0,175045486	0,063433228
O95070	1,129	0,895814623	0,175045486	0,047781853
P35914	1,13	0,885813353	0,176322773	0,052657777
P38432	1,13	0,921254131	0,176322773	0,035620552
P49356	1,13	0,966817445	0,176322773	0,014655522
Q9Y333	1,131	0,875544508	0,177598929	0,057721772
P62995	1,131	0,865619703	0,177598929	0,062672867
P00734	1,131	0,888435404	0,177598929	0,051374143

Q9P265	1,131	0,979469477	0,177598929	0,009009093
Q9BS26	1,132	0,72766436	0,178873958	0,138068896
P30520	1,132	0,848155016	0,178873958	0,071524765
P62993	1,132	0,860311565	0,178873958	0,065344239
Q9P0S9	1,132	0,883605373	0,178873958	0,053741652
A0A1W2PPZ5	1,133	0,813831176	0,180147861	0,089465677
P02765	1,133	0,857696166	0,180147861	0,066666531
Q86UU1	1,133	0,983972189	0,180147861	0,007017176
P35606	1,134	0,606334486	0,18142064	0,21728773
Q13561	1,134	0,843020453	0,18142064	0,074161889
P09012	1,135	0,833089571	0,182692298	0,079308302
Q13057	1,135	0,87148065	0,182692298	0,059742251
O43395	1,135	0,865619703	0,182692298	0,062672867
A0A1B0GUA3	1,135	0,866509779	0,182692298	0,062226532
Q92747	1,135	0,87148065	0,182692298	0,059742251
Q00839	1,136	0,606334486	0,183962835	0,21728773
Q9UJS0	1,136	0,606334486	0,183962835	0,21728773
Q96A65	1,136	0,979469477	0,183962835	0,009009093
Q9NP61	1,136	0,976211495	0,183962835	0,010456083
Q99615	1,137	0,711077095	0,185232254	0,14808331
Q9NZB2	1,137	0,867357332	0,185232254	0,061801946
Q9NYZ3	1,137	0,976922891	0,185232254	0,010139714
P51149	1,138	0,606334486	0,186500558	0,21728773
Q9H583	1,138	0,872895766	0,186500558	0,059037613
P16401	1,138	0,674764721	0,186500558	0,170847632
Q9UH65	1,138	0,857696166	0,186500558	0,066666531
Q13217	1,138	0,910784479	0,186500558	0,040584379
P10644	1,139	0,791129771	0,187767747	0,101752272
P12931	1,139	0,847463733	0,187767747	0,071878878
P15374	1,139	0,857696166	0,187767747	0,066666531
P21283	1,139	0,847463733	0,187767747	0,071878878
Q13620	1,14	0,864155777	0,189033824	0,063407962
P61163	1,14	0,85777103	0,189033824	0,066628626
E9PC69	1,14	0,925286688	0,189033824	0,033723686
P61160	1,141	0,697847902	0,190298792	0,156239223
H0Y8G5	1,141	0,601055126	0,190298792	0,221085695
P48739	1,141	0,826643042	0,190298792	0,082681985
Q96SU4	1,141	0,838069546	0,190298792	0,076719941
Q9P0K7	1,141	0,971417048	0,190298792	0,012594279
A0A0C4DGB5	1,142	0,829965842	0,191562651	0,080939781
O75477	1,142	0,827876692	0,191562651	0,082034344
Q5ZPR3	1,142	0,919602635	0,191562651	0,036399793
P68032	1,143	0,586904903	0,192825404	0,231432262
A0A3B3IUB5	1,143	0,591605764	0,192825404	0,227967603
P62318	1,143	0,606334486	0,192825404	0,21728773
Q9BW19	1,143	0,838069546	0,192825404	0,076719941
A0A0A0MRW6	1,143	0,864155777	0,192825404	0,063407962

Q8N9N2	1,143	0,889009183	0,192825404	0,051093753
Q14669	1,144	0,857696166	0,194087052	0,066666531
Q9H0H5	1,144	0,885081154	0,194087052	0,053016907
Q5TDF0	1,145	0,847463733	0,195347598	0,071878878
Q92990	1,145	0,857696166	0,195347598	0,066666531
A0A087WWW9	1,145	0,857696166	0,195347598	0,066666531
Q9BPX5	1,145	0,84572796	0,195347598	0,072769311
O15145	1,146	0,707526686	0,196607044	0,150257175
Q9H2M9	1,146	0,863262904	0,196607044	0,063856921
A0A087WXS7	1,146	0,857696166	0,196607044	0,066666531
E7EQ69	1,147	0,81309528	0,197865391	0,08985856
Q8N3V7	1,147	0,955352062	0,197865391	0,019836555
Q06210	1,148	0,565088711	0,199122642	0,247883369
B5MDU6	1,148	0,944466098	0,199122642	0,024813627
Q5VZE5	1,148	0,917460894	0,199122642	0,037412438
Q9UHR4	1,149	0,85777103	0,200378798	0,066628626
Q9H910	1,149	0,857696166	0,200378798	0,066666531
P45877	1,149	0,847463733	0,200378798	0,071878878
A0A0C4DFV2	1,15	0,859244314	0,201633861	0,065883333
O95807	1,15	0,924143221	0,201633861	0,034260718
P43121	1,15	0,911053568	0,201633861	0,040456087
P17655	1,151	0,813831176	0,202887833	0,089465677
P30533	1,151	0,606334486	0,202887833	0,21728773
P59998	1,151	0,647676373	0,202887833	0,188641946
O43432	1,151	0,976922891	0,202887833	0,010139714
Q9ULK4	1,151	0,953001598	0,202887833	0,020906371
A0A286YFF8	1,151	0,837451954	0,202887833	0,0770401
P08047	1,151	0,973472988	0,202887833	0,011676095
P05787	1,152	0,543556738	0,204140717	0,264755116
P63010	1,152	0,795613989	0,204140717	0,099297589
P45985	1,152	0,904779382	0,204140717	0,043457305
P11279	1,153	0,540751826	0,205392513	0,267002005
Q15643	1,153	0,939300148	0,205392513	0,027195609
P07686	1,154	0,606334486	0,206643224	0,21728773
P32322	1,154	0,805913757	0,206643224	0,093711431
Q15424	1,154	0,839739348	0,206643224	0,075855496
Q99961	1,154	0,823445672	0,206643224	0,084365049
Q9UM00	1,154	0,813831176	0,206643224	0,089465677
G3V1D1	1,154	0,810040646	0,206643224	0,091493189
P42695	1,154	0,966576772	0,206643224	0,014763646
Q9UHD1	1,155	0,547947097	0,207892852	0,26126137
Q9Y5M8	1,155	0,791129771	0,207892852	0,101752272
P62805	1,155	0,531599401	0,207892852	0,274415517
P80303	1,155	0,840038112	0,207892852	0,07570101
O43464	1,155	0,857696166	0,207892852	0,066666531
B8ZZS4	1,155	0,953417031	0,207892852	0,020717094
Q07812	1,156	0,733569488	0,209141398	0,134558741

Q9BVT8	1,156	0,861053521	0,209141398	0,064969853
P41240	1,157	0,847463733	0,210388864	0,071878878
Q9ULR0	1,157	0,945510726	0,210388864	0,02433354
Q14203	1,158	0,783958713	0,211635253	0,105706809
P56747	1,158	0,757253624	0,211635253	0,12075864
Q9H2V7	1,158	0,856541214	0,211635253	0,067251735
Q9NRX4	1,158	0,976211495	0,211635253	0,010456083
Q9Y6K9	1,158	0,953001598	0,211635253	0,020906371
Q6UWP7	1,158	0,842454936	0,211635253	0,074453321
Q15004	1,158	0,976211495	0,211635253	0,010456083
Q15417	1,159	0,516259313	0,212880566	0,287132101
P57081	1,159	0,97034212	0,212880566	0,013075117
P55795	1,16	0,782972176	0,214124805	0,106253671
Q5QNW6	1,16	0,50772856	0,214124805	0,294368407
P15586	1,16	0,524503266	0,214124805	0,280251803
O94973	1,16	0,813831176	0,214124805	0,089465677
P22307	1,16	0,770435684	0,214124805	0,11326361
P00568	1,16	0,819888202	0,214124805	0,086245363
P18887	1,16	0,902662281	0,214124805	0,044474705
Q01518	1,161	0,506547252	0,215367972	0,295380036
E9PB90	1,161	0,801940493	0,215367972	0,095857857
Q5TB53	1,161	0,832102331	0,215367972	0,079823261
P61026	1,162	0,499339274	0,216610069	0,301604275
P63000	1,162	0,520871325	0,216610069	0,283269551
P31150	1,163	0,812510837	0,217851097	0,090170838
O15144	1,163	0,6067422	0,217851097	0,216995798
P53004	1,163	0,769840926	0,217851097	0,113599005
Q6P1N0	1,163	0,807303403	0,217851097	0,092963217
P30038	1,163	0,818779003	0,217851097	0,086833303
Q9Y371	1,164	0,775479754	0,219091058	0,110429536
Q9NXG2	1,164	0,764140515	0,219091058	0,116826773
Q96TC7	1,164	0,917148654	0,219091058	0,037560267
H0YB16	1,164	0,606334486	0,219091058	0,21728773
P14866	1,165	0,48940339	0,220329955	0,310333027
Q14165	1,165	0,718392509	0,220329955	0,143638205
P38606	1,165	0,74224127	0,220329955	0,129454902
Q96CW1	1,165	0,758336557	0,220329955	0,120138008
O00629	1,165	0,813831176	0,220329955	0,089465677
Q99986	1,165	0,813831176	0,220329955	0,089465677
Q9H1A4	1,165	0,966576772	0,220329955	0,014763646
P02545	1,166	0,731105322	0,221567789	0,136020055
A0A087X0K9	1,166	0,813831176	0,221567789	0,089465677
Q96DB5	1,166	0,872895766	0,221567789	0,059037613
P61764	1,167	0,942717719	0,222804561	0,02561833
Q9Y2I8	1,167	0,873701792	0,222804561	0,058636774
E9PHY5	1,168	0,770435684	0,224040274	0,11326361
Q96JB5	1,168	0,875544508	0,224040274	0,057721772

B4DR12	1,168	0,975028964	0,224040274	0,010982483
A0A024R571	1,169	0,762148929	0,22527493	0,117960156
O43252	1,169	0,766965493	0,22527493	0,115224175
Q9NZ45	1,169	0,813831176	0,22527493	0,089465677
P30837	1,17	0,463727289	0,22650853	0,333737346
P78318	1,17	0,81556978	0,22650853	0,088538875
Q12906	1,171	0,45755886	0,227741076	0,339553031
Q9H1I8	1,171	0,78820403	0,227741076	0,103361349
Q5SQP8	1,171	0,932509755	0,227741076	0,030346616
K7ERE1	1,171	0,836646331	0,227741076	0,077458089
P21333	1,172	0,454273111	0,22897257	0,342682969
Q92575	1,172	0,872458986	0,22897257	0,05925498
Q5T123	1,172	0,753220885	0,22897257	0,123077647
B5MC59	1,172	0,785625184	0,22897257	0,104784603
Q9BQL6	1,172	0,952590825	0,22897257	0,021093606
P12429	1,173	0,452275637	0,230203013	0,344596806
Q6IAA8	1,173	0,783958713	0,230203013	0,105706809
P49768	1,173	0,865306643	0,230203013	0,062829962
Q8N7H5	1,173	0,775904522	0,230203013	0,110191717
P11233	1,173	0,74224127	0,230203013	0,129454902
O75351	1,173	0,795911883	0,230203013	0,099135011
Q9NXR1	1,174	0,805670533	0,231432408	0,09384252
Q96B13	1,174	0,907961986	0,231432408	0,041932334
Q5T091	1,175	0,626350729	0,232660757	0,203182413
Q9H832	1,175	0,783958713	0,232660757	0,105706809
Q7LBR1	1,175	0,768929797	0,232660757	0,114113309
P51991	1,176	0,435681575	0,23388806	0,360830806
Q8IVW6	1,176	0,768168568	0,23388806	0,114543467
E7EVE9	1,176	0,872895766	0,23388806	0,059037613
O75396	1,177	0,449483215	0,23511432	0,347286521
Q9UGI8	1,177	0,606334486	0,23511432	0,21728773
P48449	1,177	0,753220885	0,23511432	0,123077647
Q9BQ70	1,177	0,954250728	0,23511432	0,0203375
O95994	1,178	0,606334486	0,236339539	0,21728773
O94874	1,178	0,791129771	0,236339539	0,101752272
Q96SK2	1,178	0,872209582	0,236339539	0,059379147
Q4R9M9	1,179	0,864105506	0,237563718	0,063433228
O60869	1,179	0,737151234	0,237563718	0,132443403
Q9NP77	1,179	0,838069546	0,237563718	0,076719941
Q9NRK6	1,179	0,961156109	0,237563718	0,017206069
P07942	1,18	0,625783328	0,23878686	0,203576011
P16989	1,18	0,805670533	0,23878686	0,09384252
Q969H8	1,18	0,641957475	0,23878686	0,19249374
Q96F63	1,18	0,953417031	0,23878686	0,020717094
Q9Y6D5	1,181	0,756167518	0,240008965	0,121381982
Q9UJX3	1,181	0,861432517	0,240008965	0,064778739
Q68EM7	1,181	0,813831176	0,240008965	0,089465677

Q13576	1,182	0,408401443	0,241230036	0,388912732
P08758	1,183	0,399213039	0,242450074	0,398795282
O43278	1,183	0,773670578	0,242450074	0,111443919
C9JJV6	1,183	0,76669659	0,242450074	0,115376468
Q96KG9	1,184	0,813831176	0,243669081	0,089465677
Q9UI12	1,184	0,763013206	0,243669081	0,117467945
Q02809	1,184	0,766547583	0,243669081	0,115460881
E9PNP3	1,184	0,791129771	0,243669081	0,101752272
Q9UG56	1,185	0,766965493	0,244887059	0,115224175
Q9Y4X5	1,185	0,846484044	0,244887059	0,072381224
P51858	1,186	0,497563406	0,24610401	0,303151568
Q14318	1,186	0,753220885	0,24610401	0,123077647
Q6PD62	1,186	0,857696166	0,24610401	0,066666531
A0A0C4DFT8	1,186	0,731105322	0,24610401	0,136020055
Q92783	1,187	0,74203161	0,247319935	0,129577594
Q9UK22	1,187	0,762070805	0,247319935	0,118004676
P61586	1,188	0,703930733	0,248534836	0,152470073
P18031	1,188	0,756167518	0,248534836	0,121381982
O15143	1,188	0,693859784	0,248534836	0,158728283
H0Y7W6	1,188	0,910784479	0,248534836	0,040584379
P62942	1,189	0,53969056	0,249748715	0,267855178
P31641	1,189	0,932509755	0,249748715	0,030346616
Q96KC8	1,189	0,921752413	0,249748715	0,035385717
O15127	1,19	0,763013206	0,250961574	0,117467945
P07384	1,191	0,423955057	0,252173413	0,37268018
P14735	1,191	0,601055126	0,252173413	0,221085695
Q9NWV4	1,191	0,788111773	0,252173413	0,103412185
Q13426	1,191	0,737086202	0,252173413	0,132481719
Q6PGP7	1,191	0,734349677	0,252173413	0,134097092
P13861	1,192	0,634837578	0,253384236	0,197337374
O75935	1,192	0,756167518	0,253384236	0,121381982
Q6NVY1	1,193	0,767120911	0,254594043	0,115136179
P82914	1,193	0,813831176	0,254594043	0,089465677
Q9UKZ1	1,193	0,895526137	0,254594043	0,047921734
Q5T447	1,194	0,87148065	0,255802837	0,059742251
P36957	1,196	0,360029608	0,25821739	0,443661782
Q5TBP9	1,196	0,74224127	0,25821739	0,129454902
Q96AQ6	1,196	0,840198133	0,25821739	0,075618288
P38435	1,196	0,846484044	0,25821739	0,072381224
Q14894	1,197	0,939169239	0,259423152	0,02725614
P09327	1,198	0,331419882	0,260627908	0,479621442
Q8WTT2	1,198	0,714279371	0,260627908	0,146131892
P16333	1,198	0,841149343	0,260627908	0,07512689
D6RBZ0	1,2	0,321331633	0,263034406	0,493046519
A0A0G2JPP5	1,2	0,731105322	0,263034406	0,136020055
Q00169	1,2	0,709993813	0,263034406	0,148745436
Q9HCN4	1,2	0,77296498	0,263034406	0,111840182

A0A494C1T9	1,201	0,844181675	0,264236151	0,073564079
Q96AC1	1,202	0,656631039	0,265436896	0,182678592
O95372	1,202	0,714279371	0,265436896	0,146131892
Q96D71	1,202	0,944466098	0,265436896	0,024813627
O95721	1,203	0,735492771	0,266636643	0,133421592
Q16698	1,203	0,74224127	0,266636643	0,129454902
O95758	1,204	0,82575134	0,267835392	0,083150713
O75607	1,205	0,722923095	0,269033146	0,140907901
O15460	1,206	0,606334486	0,270229907	0,21728773
F2Z2E2	1,206	0,87148065	0,270229907	0,059742251
Q13451	1,206	0,877699002	0,270229907	0,056654395
Q96EP5	1,207	0,76669659	0,271425676	0,115376468
I3L4X2	1,207	0,911797393	0,271425676	0,040101654
Q9Y3P9	1,208	0,857696166	0,272620455	0,066666531
O00267	1,21	0,738272729	0,275007047	0,131783174
P25685	1,21	0,610733581	0,275007047	0,2141482
Q9UBF2	1,21	0,819615116	0,275007047	0,086390041
A0A2R8Y6Y7	1,21	0,671033509	0,275007047	0,173255792
Q9H8H2	1,21	0,87148065	0,275007047	0,059742251
Q15334	1,211	0,864155777	0,276198865	0,063407962
P50453	1,212	0,56030391	0,277389699	0,251576347
P61019	1,212	0,566821591	0,277389699	0,246553615
B4DR61	1,212	0,289708242	0,277389699	0,538039149
Q9BW60	1,212	0,683527451	0,277389699	0,165244039
A0A1W2PNX8	1,213	0,635517488	0,27857955	0,196872494
E9PDM8	1,214	0,753220885	0,279768422	0,123077647
P05556	1,215	0,523121838	0,280956314	0,28139715
Q147X3	1,215	0,76669659	0,280956314	0,115376468
Q8N357	1,215	0,686202957	0,280956314	0,163547415
Q96J02	1,218	0,692284543	0,284514133	0,159715365
Q96AX1	1,218	0,823238104	0,284514133	0,084474536
Q9HCS7	1,218	0,874220393	0,284514133	0,058379067
P09960	1,219	0,249871571	0,285698126	0,602283153
P22033	1,22	0,723695662	0,286881148	0,140444031
X6RLX0	1,22	0,683260797	0,286881148	0,165413497
Q9BRR6	1,22	0,859244314	0,286881148	0,065883333
C9JZR2	1,221	0,405847124	0,2880632	0,391637527
Q13596	1,221	0,613949788	0,2880632	0,211867146
O95782	1,222	0,606334486	0,289244285	0,21728773
Q12797	1,222	0,688256122	0,289244285	0,162249917
H0YGR4	1,222	0,606334486	0,289244285	0,21728773
Q3KQV9	1,222	0,792796735	0,289244285	0,100838147
Q14690	1,223	0,659047877	0,290424404	0,181083035
E9PGM4	1,224	0,763013206	0,291603558	0,117467945
O43399	1,225	0,224635205	0,292781749	0,64852218
P05413	1,225	0,497563406	0,292781749	0,303151568
Q7KZF4	1,226	0,22155245	0,293958979	0,654523443

Q14019	1,226	0,34346339	0,293958979	0,464119548
A0A087WT45	1,226	0,762070805	0,293958979	0,118004676
Q96QR8	1,226	0,846484044	0,293958979	0,072381224
J3KNQ4	1,227	0,693859784	0,295135249	0,158728283
Q9HD20	1,227	0,753220885	0,295135249	0,123077647
Q9NPQ8	1,229	0,606334486	0,297484916	0,21728773
H7BZW6	1,229	0,64116843	0,297484916	0,19302787
Q9NVZ3	1,23	0,718886374	0,298658316	0,143339748
P13473	1,23	0,606334486	0,298658316	0,21728773
Q6P1A2	1,231	0,659047877	0,299830762	0,181083035
Q7Z2W4	1,232	0,606334486	0,301002256	0,21728773
Q9Y4G6	1,232	0,649860965	0,301002256	0,187179549
Q7Z2K6	1,233	0,606334486	0,3021728	0,21728773
Q96KR1	1,234	0,772492172	0,303342394	0,112105913
Q92572	1,234	0,707526686	0,303342394	0,150257175
Q9Y530	1,234	0,634837578	0,303342394	0,197337374
P16070	1,236	0,630699104	0,305678743	0,200177786
Q86VR2	1,237	0,731105322	0,3068455	0,136020055
E9PQR7	1,237	0,733750958	0,3068455	0,134451319
Q96D46	1,238	0,76178659	0,308011315	0,118166677
Q92542	1,239	0,606334486	0,309176187	0,21728773
P58107	1,24	0,518117089	0,310340121	0,285572083
J3QQZ9	1,24	0,606334486	0,310340121	0,21728773
P09497	1,241	0,610213782	0,311503115	0,214517988
Q8NEW0	1,241	0,648060354	0,311503115	0,188384546
Q9NZD8	1,241	0,773685257	0,311503115	0,111435679
O96011	1,242	0,882018844	0,312665174	0,054522136
Q9Y2H0	1,243	0,783958713	0,313826296	0,105706809
P46063	1,244	0,56030391	0,314986485	0,251576347
Q9P2D3	1,244	0,758336557	0,314986485	0,120138008
P15927	1,245	0,610213782	0,316145742	0,214517988
O14682	1,246	0,911797393	0,317304068	0,040101654
Q9Y508	1,249	0,610213782	0,320773477	0,214517988
Q13393	1,249	0,813831176	0,320773477	0,089465677
C9JME2	1,249	0,753220885	0,320773477	0,123077647
Q9Y2D4	1,249	0,81309528	0,320773477	0,08985856
F5GZ78	1,25	0,606334486	0,321928095	0,21728773
B7Z385	1,252	0,745527526	0,324234562	0,127536317
P49748	1,253	0,299166486	0,325386415	0,52408706
Q9NZ32	1,253	0,786281324	0,325386415	0,10442204
P30044	1,254	0,152218989	0,326537348	0,817531167
B4DDF4	1,254	0,531981206	0,326537348	0,27410371
P42025	1,254	0,81309528	0,326537348	0,08985856
M0R3F6	1,254	0,847463733	0,326537348	0,071878878
P54920	1,255	0,292209485	0,327687364	0,534305691
Q8NCA5	1,255	0,567270199	0,327687364	0,246210031
Q5RI15	1,255	0,624190559	0,327687364	0,204682804

Q8TF74	1,255	0,864155777	0,327687364	0,063407962
Q969T9	1,256	0,791129771	0,328836464	0,101752272
Q460N5	1,257	0,64273553	0,32998465	0,191967692
P10599	1,258	0,146861601	0,331131922	0,833091742
Q5SRE7	1,258	0,780396648	0,331131922	0,107684605
Q9BZJ0	1,259	0,585538736	0,332278283	0,232444369
Q9Y4C8	1,259	0,790944349	0,332278283	0,101854072
B1AM21	1,259	0,816819656	0,332278283	0,08787382
Q6P9B6	1,26	0,729672938	0,333423734	0,13687176
Q9NUY8	1,261	0,813831176	0,334568276	0,089465677
P37802	1,263	0,121045127	0,336854639	0,91705269
P12814	1,264	0,120550723	0,337996464	0,918830181
Q9H4L7	1,264	0,766992332	0,337996464	0,115208978
A0A3B3ITH3	1,264	0,807127549	0,337996464	0,093057829
P46940	1,265	0,118191044	0,339137385	0,927415431
A0A0A0MRM8	1,268	0,551808205	0,342554745	0,258211846
Q9BUF5	1,27	0,492479671	0,344828497	0,307611692
Q00653	1,27	0,632298819	0,344828497	0,199077629
Q14746	1,27	0,847463733	0,344828497	0,071878878
Q9BTX7	1,271	0,753220885	0,34596403	0,123077647
Q9C075	1,272	0,472136253	0,347098671	0,325932651
O15264	1,272	0,791129771	0,347098671	0,101752272
Q14195	1,273	0,5083632	0,348232419	0,293825895
A0A0A0MSK5	1,277	0,606334486	0,352758525	0,21728773
P62306	1,278	0,558977452	0,353887836	0,25260571
O94851	1,279	0,811775856	0,355016264	0,09056387
A0A0A0MRF6	1,28	0,51361169	0,35614381	0,2893651
P67936	1,281	0,142457686	0,357270476	0,846314114
G5EA48	1,281	0,903381422	0,357270476	0,044128845
O94760	1,282	0,088265878	0,358396262	1,054207154
P04264	1,283	0,088074641	0,35952117	1,055149118
Q96BJ3	1,283	0,838069546	0,35952117	0,076719941
O00515	1,284	0,535570201	0,360645202	0,271183595
Q96KP1	1,284	0,775697546	0,360645202	0,110307582
P51153	1,285	0,606334486	0,361768359	0,21728773
A0A0A0MSB8	1,285	0,649532438	0,361768359	0,187399155
Q9H6Y2	1,285	0,535570201	0,361768359	0,271183595
Q6YP21	1,286	0,509124601	0,362890643	0,293175917
Q6VN20	1,286	0,606334486	0,362890643	0,21728773
Q9BWH2	1,287	0,569293328	0,364012054	0,244663906
P29966	1,288	0,181742137	0,365132593	0,74054437
Q96SI9	1,288	0,660147783	0,365132593	0,180358831
O00233	1,288	0,606334486	0,365132593	0,21728773
O14974	1,289	0,392123116	0,366252264	0,406577555
Q9HAU5	1,289	0,567270199	0,366252264	0,246210031
O15400	1,29	0,51895783	0,367371066	0,284867931
A0A0C4DGS9	1,29	0,612218264	0,367371066	0,213093718

Q6NUQ4	1,291	0,360205272	0,368489001	0,443449935
O75083	1,293	0,072983293	0,370722275	1,136776545
Q9HB90	1,294	0,558977452	0,371837617	0,25260571
Q96TA1	1,294	0,640357929	0,371837617	0,193577209
H3BPE1	1,295	0,709993813	0,372952098	0,148745436
P57088	1,297	0,107390647	0,37517848	0,969033541
Q5BKZ1	1,297	0,606334486	0,37517848	0,21728773
O00178	1,298	0,606334486	0,376290383	0,21728773
Q9H1E5	1,301	0,676632308	0,379620962	0,169647269
Q08257	1,302	0,098449588	0,380729449	1,006786097
Q9UNH7	1,303	0,495913527	0,381837084	0,304594045
Q9UN86	1,303	0,453875972	0,381837084	0,343062808
A8CG34	1,303	0,768622523	0,381837084	0,114286894
P20645	1,304	0,507194832	0,38294387	0,29482518
A0A494C1E9	1,304	0,718993681	0,38294387	0,143274926
P52565	1,305	0,059359723	0,384049807	1,226508134
G3V150	1,305	0,634837578	0,384049807	0,197337374
Q9NQG5	1,307	0,216174191	0,386259141	0,665196158
A0A0A0MTH3	1,308	0,447815076	0,387362541	0,34890129
Q9BVS4	1,309	0,674100915	0,388465097	0,171275083
O00425	1,311	0,091902001	0,390667686	1,036675033
C9JK83	1,312	0,722923095	0,39176772	0,140907901
F5H442	1,313	0,442560996	0,392866916	0,354026864
Q96MW1	1,313	0,763013206	0,392866916	0,117467945
O60271	1,314	0,346299299	0,393965276	0,460548388
O43707	1,315	0,049066044	0,3950628	1,309218956
P11387	1,315	0,32315083	0,3950628	0,490594724
Q13423	1,315	0,550269182	0,3950628	0,259424809
P68366	1,316	0,047479315	0,396159489	1,323495555
Q9UKM9	1,316	0,047717857	0,396159489	1,321319069
O60664	1,317	0,047353608	0,397255346	1,324646925
O14578	1,317	0,547947097	0,397255346	0,26126137
Q14247	1,319	0,045720652	0,399444565	1,339887585
H0YEF3	1,319	0,838069546	0,399444565	0,076719941
Q7L523	1,32	0,560326951	0,40053793	0,251558488
C9J3L8	1,321	0,321331633	0,401630467	0,493046519
Q9ULV4	1,322	0,252219768	0,402722177	0,598220878
O95486	1,322	0,451397499	0,402722177	0,345440851
Q14573	1,322	0,606334486	0,402722177	0,21728773
Q00403	1,322	0,606334486	0,402722177	0,21728773
P02533	1,326	0,480729835	0,407080775	0,318098924
Q86SF2	1,328	0,606334486	0,409255147	0,21728773
E7EVI4	1,329	0,557089308	0,410341105	0,254075177
O15031	1,33	0,483203372	0,411426246	0,315870044
Q8TBA6	1,33	0,606334486	0,411426246	0,21728773
F8W809	1,331	0,100194149	0,412510571	0,999157639
A0A0A0MS50	1,333	0,606334486	0,41467678	0,21728773

P48681	1,334	0,407626794	0,415758667	0,389737277
Q9NP90	1,334	0,859244314	0,415758667	0,065883333
Q15404	1,335	0,264214568	0,416839742	0,57804324
Q2M2I8	1,336	0,686202957	0,417920008	0,163547415
H0YME5	1,337	0,606334486	0,418999465	0,21728773
Q9H444	1,338	0,256860336	0,420078116	0,590302954
O43657	1,338	0,431483907	0,420078116	0,365035397
P46926	1,339	0,031418351	0,421155961	1,502816613
O00754	1,339	0,556076711	0,421155961	0,254865293
Q9H6S3	1,34	0,228106188	0,422233001	0,641862933
A4D1P6	1,34	0,668800867	0,422233001	0,174703173
Q9HBI1	1,341	0,660147783	0,423309237	0,180358831
M0R0I0	1,341	0,606334486	0,423309237	0,21728773
P24941	1,342	0,499339274	0,424384672	0,301604275
A0A494C0A8	1,343	0,585538736	0,425459305	0,232444369
Q9Y3A5	1,344	0,132109884	0,426533138	0,879064689
Q15785	1,345	0,028260771	0,427606173	1,548815994
Q9UKY7	1,346	0,308739552	0,42867841	0,51040773
O95674	1,347	0,531599401	0,429749851	0,274415517
O94903	1,348	0,346299299	0,430820497	0,460548388
A8MQB8	1,35	0,753973257	0,432959407	0,122644058
Q16513	1,351	0,360914133	0,434027675	0,442596111
Q9UP95	1,354	0,6255212	0,437227739	0,203757967
P02792	1,356	0,340149036	0,439357178	0,468330756
Q3KQU3	1,356	0,791129771	0,439357178	0,101752272
Q99707	1,358	0,689490376	0,44148348	0,161471791
Q9H223	1,359	0,36620324	0,442545456	0,436277818
Q99536	1,36	0,020504873	0,443606651	1,688142916
Q9NR12	1,36	0,621506574	0,443606651	0,206554273
Q9HC07	1,363	0,472136253	0,446785562	0,325932651
Q92882	1,364	0,516280989	0,447843644	0,287113867
Q9P0U3	1,368	0,752195167	0,45206823	0,123669461
P50552	1,373	0,415785974	0,457331625	0,381130165
Q9Y305	1,375	0,322661832	0,459431619	0,491252405
Q14315	1,376	0,014803104	0,46048047	1,82964721
Q13242	1,376	0,250341354	0,46048047	0,601467403
Q5JXR6	1,376	0,766028473	0,46048047	0,115755087
Q15149	1,377	0,083674379	0,461528559	1,077407502
P48507	1,381	0,216174191	0,46571332	0,665196158
A0A499FIX8	1,383	0,132109884	0,467801156	0,879064689
Q8IUF8	1,383	0,506585983	0,467801156	0,295346831
Q96JB2	1,383	0,621618822	0,467801156	0,206475844
0A2R8YCH5 (Catenin beta-1	1,385	0,012278803	0,469885976	1,910843968
O00299	1,387	0,011943224	0,471967788	1,922878422
O15118	1,387	0,473528413	0,471967788	0,324653957
F5H5N2	1,387	0,681762212	0,471967788	0,166367074
Q9H939	1,387	0,531981206	0,471967788	0,27410371

Q13043	1,389	0,287338075	0,474046599	0,541606822
A0A0A0MSZ4	1,39	0,011060563	0,475084883	1,956222766
Q86UU0	1,39	0,64116843	0,475084883	0,19302787
E9PGZ1	1,392	0,01052153	0,477159211	1,977921102
H0YIB2	1,395	0,201792332	0,480265122	0,695095341
Q5VZK9	1,395	0,606334486	0,480265122	0,21728773
Q5KU26	1,395	0,606334486	0,480265122	0,21728773
Q92466	1,397	0,288636985	0,482332021	0,539648021
P13796	1,401	0,640357929	0,486456956	0,193577209
Q15654	1,404	0,585538736	0,489542936	0,232444369
F8VV59	1,407	0,718886374	0,492622329	0,143339748
Q92888	1,408	0,408493365	0,493647334	0,388814993
O95817	1,41	0,34346339	0,495695163	0,464119548
P32780	1,411	0,813831176	0,496717988	0,089465677
O60566	1,412	0,653548529	0,497740089	0,184722159
P23142	1,413	0,509629882	0,498761466	0,292745115
Q9NVH0	1,413	0,633574926	0,498761466	0,198202018
Q15208	1,413	0,674764721	0,498761466	0,170847632
Q99653	1,415	0,186400275	0,500802053	0,729553451
Q9Y4B6	1,415	0,674764721	0,500802053	0,170847632
O00161	1,418	0,153090159	0,503857533	0,815052726
F8VWK8	1,42	0,099596703	0,50589093	1,001755038
A0A3B3ISU0	1,42	0,620737345	0,50589093	0,207092126
H0Y2Y8	1,422	0,007783535	0,507921465	2,108823117
P42566	1,425	0,318385603	0,510961919	0,497046579
Q8IWE2	1,427	0,410873446	0,512985335	0,386291925
Q9NX62	1,428	0,304723578	0,513995979	0,516093941
A0A0A0MRL6	1,431	0,252219768	0,517023672	0,598220878
P15924	1,432	0,004426505	0,518031493	2,353939041
K7EPQ7	1,433	0,195432271	0,51903861	0,709003721
O75146	1,435	0,572119435	0,521050737	0,242513299
P00533	1,439	0,070363822	0,525066592	1,152650579
Q9UHG0	1,444	0,494016689	0,530070742	0,306258379
Q5TD07	1,453	0,363464403	0,539034703	0,439538117
Q9NUP9	1,455	0,10048577	0,541019153	0,997895435
Q9UNY4	1,455	0,590129247	0,541019153	0,229052861
Q08378	1,457	0,148236576	0,543000877	0,829044625
Q9Y6N9	1,457	0,762122768	0,543000877	0,117975064
O95470	1,459	0,172874725	0,544979883	0,762268498
Q13033	1,459	0,225723953	0,544979883	0,646422353
P50454	1,462	0,002248796	0,547943311	2,64804994
Q5T9L3	1,462	0,168630694	0,547943311	0,773063373
O14908	1,464	0,08613642	0,549915554	1,064813182
Q8IWZ8	1,466	0,555514924	0,551885103	0,255304269
Q16527	1,469	0,048268274	0,554834396	1,316338231
Q15121	1,469	0,11441159	0,554834396	0,941529979
P18206	1,472	0,001775389	0,557777671	2,750706475

A0A0B4J1R2	1,474	0,316595358	0,559736524	0,499495457
Q15811	1,475	0,596214744	0,560714954	0,224597288
Q96LR5	1,477	0,610213782	0,562669826	0,214517988
B1AKC9	1,477	0,417027876	0,562669826	0,379834914
Q07820	1,478	0,606334486	0,563646269	0,21728773
Q12965	1,48	0,135047935	0,565597176	0,869512052
P13647	1,483	0,037097936	0,568518598	1,430650252
Q6NUM9	1,486	0,12350189	0,571434116	0,908326396
O00401	1,488	0,304925845	0,573374526	0,515805764
P98194	1,491	0,427825157	0,576280258	0,368733682
P07996	1,491	0,194645563	0,576280258	0,710755492
Q9BYN0	1,495	0,135018151	0,580145484	0,869607844
Q8IXU6	1,495	0,606334486	0,580145484	0,21728773
Q9UL15	1,496	0,366759981	0,581110175	0,435618058
Q96IZ0	1,501	0,20070629	0,585923977	0,697439017
A6NN50	1,507	0,31792431	0,591679417	0,497676263
D6R9W2	1,511	0,452275637	0,595503661	0,344596806
P50749	1,513	0,451397499	0,597411988	0,345440851
O95861	1,514	0,152614333	0,598365205	0,816404677
Q9NQW6	1,516	0,079484364	0,600269754	1,099718296
P13645	1,518	0,000542558	0,602171791	3,265553828
Q4G148	1,52	0,241423546	0,604071324	0,617220376
O94919	1,521	0,366759981	0,605020153	0,435618058
P35237	1,525	0,044267657	0,608809243	1,353913464
Q9NZD2	1,527	0,070510602	0,610700062	1,151745577
Q9H0B6	1,539	0,507291095	0,621993232	0,294742761
Q9H9H4	1,539	0,323404314	0,621993232	0,490254191
Q9NZM1	1,547	0,000267546	0,629473197	3,572601538
O95757	1,547	0,275129929	0,629473197	0,560462164
Q9UJ70	1,558	0,054084186	0,639695233	1,266929702
Q709C8	1,565	0,383227609	0,646162657	0,41654321
Q96CG8	1,565	0,066143779	0,646162657	1,179510996
Q15555	1,572	0,185654327	0,652601218	0,731294924
Q969S9	1,582	0,455813974	0,6617496	0,341212365
P31947	1,583	0,000101267	0,662661255	3,994532056
P08727	1,59	8,29245E-05	0,669026766	4,081317138
P29373	1,592	7,98429E-05	0,670840336	4,097763697
P40763	1,596	0,007113854	0,674460652	2,147895052
Q9UBP6	1,597	0,436331913	0,675364313	0,360183022
Q9UQN3	1,599	0,19494449	0,677169939	0,710089035
P05161	1,601	0,001674099	0,678973308	2,776218863
E9PNF5	1,601	0,318045608	0,678973308	0,497510597
Q96HC4	1,606	0,008825314	0,683471893	2,054269834
P58004	1,607	0,363081534	0,684369929	0,439995838
B1AMN7	1,62	0,234797127	0,695993813	0,629307221
Q15437	1,627	0,004426505	0,702214251	2,353939041
P40121	1,634	0,005643124	0,708407983	2,248480407

Q9NQC3	1,635	0,019970662	0,709290636	1,699607539
C9JW51	1,636	0,088074641	0,710172748	1,055149118
Q9NQ88	1,641	0,006636143	0,714575239	2,178084264
A0A087WXX9	1,642	0,172279775	0,715454127	0,763765704
Q9NUQ6	1,647	0,004704853	0,719840555	2,327453941
P11047	1,658	0,006277616	0,729444007	2,202205253
**42771 (p16-INK4a/CDKN2A**	1,66	0,060478416	0,731183242	1,218399592
Q6DT37	1,661	0,606334486	0,732052073	0,21728773
P23229	1,669	0,009323666	0,738983955	2,030413293
Q5JSH3	1,671	0,137910639	0,740711733	0,860402229
E9PMT2	1,674	0,006701423	0,743299528	2,173832968
Q96EK6	1,687	0,015482893	0,754459974	1,810147888
O95684	1,69	0,482518602	0,757023247	0,316485939
A6NKZ2	1,696	0,130239867	0,76213617	0,885256056
P11166	1,715	2,55025E-06	0,778208576	5,593417244
Q93034	1,723	0,055079846	0,784922702	1,259007283
Q9UK76	1,758	0,042022246	0,81393507	1,376520739
Q7LG56	1,765	0,042215118	0,819668183	1,374531992
B7Z6U4	1,765	0,019532331	0,819668183	1,709245925
Q9H3P7	1,769	0,024793822	0,822934048	1,605656521
Q9NRN7	1,775	0,006605767	0,827819025	2,180076749
Q8NHU6	1,776	0,067229627	0,828631582	1,172439298
Q8NBM4	1,782	0,11799662	0,833497337	0,928130433
Q8NHV4	1,783	0,238182753	0,834306703	0,623089689
Q96DH6	1,786	0,00238089	0,83673208	2,623260669
Q9H5Q4	1,804	0,074476028	0,851199339	1,127983493
Q9UJG1	1,807	0,031250989	0,853596506	1,505136234
P49454	1,815	0,389158151	0,859969548	0,409873869
A6NHR9	1,818	0,004489811	0,8623522	2,34777194
Q15796	1,822	0,070003966	0,865522959	1,154877355
Q9NRW3	1,823	0,023401873	0,866314561	1,630749382
P55085	1,832	0,141368936	0,873419503	0,849646011
P35908	1,839	6,95892E-08	0,87892148	7,157458156
Q9Y666	1,862	0,173377218	0,896853073	0,76100797
Q9H330	1,875	0,019660797	0,906890596	1,706398881
Q53FA7	1,882	1,46285E-06	0,912266628	5,834800204
Q96LJ7	1,921	0,034372816	0,941857519	1,463784887
Q14651	1,922	0,004788762	0,942608336	2,319776747
O95084	1,922	0,032262769	0,942608336	1,491298361
Q8NFC6	1,955	0,110097154	0,967168608	0,958223907
O00560	1,965	0,018888051	0,974529312	1,723812853
Q6DN03	1,978	0,006528812	0,984042426	2,185165837
O15230	1,98	0,006921839	0,98550043	2,159778506
A5PLL7	1,986	0,005309851	0,989865623	2,274917666
O95425	2,004	0,002932372	1,002882509	2,532780936
Q9UJX6	2,027	0,016345948	1,019346089	1,786589887
Q15678	2,032	0,033373929	1,022900402	1,476592662

Q9UJC5	2,051	0,075890933	1,036327492	1,119810108
Q99541	2,067	1,26857E-07	1,047538389	6,896685563
Q9H8V3	2,092	0,002530819	1,064882852	2,596738914
O75915	2,094	0,001006108	1,066261442	2,997355398
P29083	2,13	0,002075384	1,09085343	2,682901536
G3XAH0	2,132	0,014287912	1,092207438	1,845031233
P98172	2,158	0,043705225	1,109694865	1,35946664
Q9UK59	2,196	0,000355809	1,134878054	3,448783071
P29317	2,222	0,000506843	1,151858817	3,295126547
Q01968	2,229	0,000178083	1,156396617	3,749377537
Q8WX93	2,23	0,002173739	1,15704371	2,662792603
Q92733	2,231	0,152667384	1,157690514	0,816253736
Q14192	2,232	1,33206E-05	1,158337027	4,875476213
E7ENN3	2,266	0,018587423	1,180147861	1,730780818
Q5JUW8	2,306	0,003576813	1,205392513	2,446503765
Q9P253	2,309	0,004704853	1,207268173	2,327453941
Q14517	2,331	0,046215567	1,220949004	1,335211714
Q8WUP2	2,406	0,000223272	1,266636643	3,651165737
P36952	2,493	4,37181E-05	1,317882883	4,359338721
P25208	2,533	4,38355E-05	1,340847077	4,358174035
Q8TC05	2,54	0,000298594	1,344828497	3,524918923
E9PI99	2,556	2,30685E-06	1,353887836	5,636980644
P17301	2,64	1,24807E-10	1,40053793	9,903761056
P31431	2,692	0,000119327	1,42867841	3,923261278
P09382	2,748	2,49209E-15	1,458382004	14,60343628
Q96QD9	2,921	0,000249824	1,546462358	3,602365842
Q9P2W9	2,993	0,028345924	1,581592279	1,547509382
Q9H4L5	3,148	0,000138887	1,654435541	3,857338403
Q01995	3,173	2,49209E-15	1,665847521	14,60343628
P07305	3,227	4,58233E-08	1,690193578	7,338913638
Q9BYJ9	3,362	1,82223E-06	1,749319725	5,739396808
P20337	3,431	7,22236E-13	1,778629126	12,14132087
Q02952	3,451	5,07874E-14	1,787014474	13,29424402
Q15124	3,596	1,03076E-07	1,846393021	6,986842443
P04083	3,714	2,49209E-15	1,892973815	14,60343628
Q9NZU0	4,044	0,000145835	2,015782997	3,836138234
Q9Y3E0	4,162	6,82439E-10	2,057276965	9,165936162
Q13203	4,399	1,23578E-07	2,137175601	6,908058838
Q96Q15	5,153	1,03096E-10	2,365412592	9,986758184
Q4G0F5	19,404	2,49209E-15	4,27828218	14,60343628

Supplementary Table S4: Correlative analysis between DUSP9, DUSP5, HMGCR and EZH2 in HB.

	**Ikeda et al**	**Carillo-Reixach et al**	**Lopez-Terrada et al**	**Karns et al**	**Buendia et al**	**Kappler et al**	**Raymond et al**
**DUSP9 vs EZH2**	R=0.14	R=0.66	R=0.65	R=0.76	R=0.51	R=0.78	R=0.62
	p=0.29	**p<0.0001**	**p<0.0001**	**p<0.0001**	**p<0.01**	**p<0.01**	**p<0.001**
**DUSP5 vs EZH2**	R=-0.46	R=-0.64	R=-0.45	R=-0.57	R=-0.31	R=-0.78	R=0.64
	**p<0.001**	**p<0.0001**	**p<0.001**	**p<0.01**	p=0.12	**p<0.01**	**p<0.001**
**DUSP5 vs DUSP9**	R=-0.28	R=-0.67	R=-0.44	R=-0.68	R=-0.4	R=-0.93	R=-0.37
	**p<0.05**	**p<0.0001**	**p<0.001**	**p<0.001**	**p<0.05**	**p<0.0001**	**p<0.05**
**HMGCR vs EZH2**	R=0.34	R=0.48	R=0.49	R=0.57	R=0.31	R=0.53	R=0.35
	**P<0.05**	**p<0.01**	**p<0.001**	**p<0.01**	p=0.12	p=0.08	p=0.055

Supplementary Table S5: Cell lines used in this study.

Name	Cancer type	Citations	Supplier	Cat no.	Authentication
Huh6	Hepatoblastoma	PMID:29152775 PMID:9671767	JCRB Cell bank	JCRB0401	STR profiling
HepG2	Hepatoblastoma	PMID:29152775 PMID:9671767	LGC Promochem	ATCC n° HB-8065	STR profiling
PDX HB282	Hepatoblastoma	PMID: 34239847	Xentech company	/	STR profiling
PDX HB303	Hepatoblastoma	PMID: 36776327	Xentech company	/	STR profiling
Huh7	Hepatocellular carcinoma	PMID: 35839778 PMID: 35634422		JCRB0403	STR profiling
NCI-H23	Lung carcinoma	PMID: 35839778 PMID: 31978347	ATCC	ATCC n° CRL-5800	STR profiling
WT-CLS1	Rhabdoid tumor of the kidney	PMID: 20106868 PMID: 34384283	CLS	300379	STR profiling
HOS-MNNG	Osteosarcoma	PMID: 25138053 PMID: 26320182	ATCC	CRL-1547	STR profiling
U2-OS	Osteosarcoma	PMID: 25138053 PMID: 24646477	ATCC	HTB-96	STR profiling

Supplementary Table S6: Sequences of siRNAs and primers.

Name	Sequence (5' → 3')	Supplier
Small interfering RNAs		
siEZH2-1	GAG GGA AAG UGU AUG AUA A (TT)	Eurofins
siEZH2-2	UUU GGC UUC AUC UUU AUU G (TT)	Eurofins
siDUSP9-1	CCA UUG AGU UCA UUG AUG A (dT) (dT)	Sigma
siDUSP9-2	UUA GGU UCG GCC UUC GCU U (TT)	Sigma
Primers for cloning and quantitative PCR		
EZH2 Fw Plasmid	GCG CGC TAG C*AC C***AT G**GG CCA GAC TGG GAA	Eurofins
EZH2 Rv Plasmid	GCG CAC GCG T**TC A**AG GGA TTT CCA TTT CTC	Eurofins
DUSP9 Fw Plasmid	GCG CGC TAG C*AC C***AT G**GA GGG TCT GGG CC	Eurofins
DUSP9 Rv Plasmid	GCG CAC GCG T**CT A**GG TGG GGG CCA GC	Eurofins
DUSP5 Fw Plasmid	GCG CGC TAG C*AC C***AT G**AA GGT CAC GTC GCT	Eurofins
DUSP5 Rv Plasmid	GCG CAC GCG T**TC A**GC AGG ATG TGG CCG TTG	Eurofins
EZH2 H689A Fw	GTTTGGATTTACCGAAGCATTTGCAAAACGAATTTTGTTACCCTTGCG	Eurofins
EZH2 H689A Rv	CGCAAGGGTAACAAAATTCGTTTTGCAAATGCTTCGGTAAATCCAAAC	Eurofins
EZH2 Fw sequencing	ATG TGC TGG AAT CAA AGG ATA CAG AC	Eurofins
EZH2 Rv sequencing	GAA TTT GCT TCA GAG GAG CTC GAA GT	Eurofins
DUSP9 Fw sequencing	ATC CGA GGC TGA CCG CGA CT	Eurofins
DUSP9 Rv sequencing	AGA TAG AGG TTG GGC AGG ATC T	Eurofins
DUSP5 Fw sequencing	GAA AAC CAG TGG TAA ATG TCA	Eurofins
DUSP5 Rv sequencing	TGA CAT TTA CCA CTG GTT TTC	Eurofins
EZH2 Fw q-PCR	AAT CAG AGT ACA TGC GAC TGA GA	Eurofins
EZH2 Rv q-PCR	GCT GTA TCC TTC GCT GTT TCC	Eurofins
DUSP9 Fw q-PCR	CAG CCG TTC TGT CAC CGT C	Eurofins
DUSP9 Rv q-PCR	CAA GCT GCG CTC AAA GTC C	Eurofins

GAPDH Fw<br>q-PCR	CAA GGA GTA AGA CCC CTG GA	Eurofins
GAPDH Rv<br>q-PCR	AGG GGA GAT TCA GTG TGG TG	Eurofins
Primers for ChIP and quantitative PCR		
AR Fw<br>q-PCR	AAG GGC TAG AAG GCG AGA	Eurofins
AR Rv<br>q-PCR	GAC TTG TAG AGA GAC AGG GTA GA	Eurofins
CDKN2A Fw<br>q-PCR	CCA TGG CCA GCT AAT TTT TG	Eurofins
CDKN2A Rv<br>q-PCR	GAA AAT GCT TTG GAG CAG GA	Eurofins
DUSP5 +0.83 Fw<br>q-PCR	GTG GAG TTG GTT ATG CTG CG	Eurofins
DUSP5 +0.83 Rv<br>q-PCR	CAC CAA GGG AGA GTG GAT CG	Eurofins
DUSP5 +1.04 Fw<br>q-PCR	CCT AGC CAG CTG TGG TCT TC	Eurofins
DUSP5 +1.04 Rv<br>q-PCR	CAG GAT GGG TCC CAG GGA TA	Eurofins
DUSP5 +1.3 Fw<br>q-PCR	TGG TAT GGA GAT TCT GCG GC	Eurofins
DUSP5 +1.3 Rv<br>q-PCR	ACA CAA AGC AGG AGC AGT CA	Eurofins
DUSP5 +1.67 Fw<br>q-PCR	TGA GTT TCA CAC AGT CCG GG	Eurofins
DUSP5 +1.67 Rv<br>q-PCR	AAA TCA GGA GGC ACG CTG AA	Eurofins
DUSP5 +2.0 Fw<br>q-PCR	GGC CCT GCT GAC AGT TTA GA	Eurofins
DUSP5 +2.0 Rv<br>q-PCR	CAT CAC AGG GCC TTC ATG CT	Eurofins
DUSP5 +2.37 Fw<br>q-PCR	TGA CCA TTA GGT TGC GTC CC	Eurofins
DUSP5 +2.37 Rv<br>q-PCR	ACG CAA GAG CAC ATG GAG AA	Eurofins

Underlined: restriction site. Italic: Kozak sequence. Bold: start or stop codon.

Supplementary Table S7: Antibodies used in this study.

Target protein	Supplier	Cat no.	Dilution
EZH2	Cell signaling	5246S	1/1000
P16	Abcam	Ab108349	1/1000
P21	Cell Signaling	2947	1/1000
DUSP3	GeneTex	GTX109149	1/1000
DUSP5	Abcam	ab200708	1/1000
DUSP6	Ozyme	39441S	1/1000
DUSP9	Abcam	ab194355	1/2500
DUSP16	GeneTex	GTX66001	1/1000
P44/42 MAPK(ERK1/2)	Cell Signaling	9102	1/1000
Phospho-p44/42 (p-ERK1/2)	Cell Signaling	9106	1/1000
β-catenin	BD Bioscience	610154	1/5000
CTSV	Abcam	ab24508	1/1000
H3K27me3	Ozyme	9733S	1/1000
H3K27me3	Cell Signaling	9733T	1/100
Histone H3	Santa-Cruz	517576	1/500
GAPDH	Ozyme	BLE649203	1/15000
anti mouse IgG-HRP	Biorad	170-6516	1/3000
anti-rabbit IgG-HRP	Sigma Aldrich	A0545	1/5000
Isotype control IgG	Cell Signaling	3900S	1/100

Supplementary Table S8: Reagents used in the ChIP assay.

Reagent	***Reference***
cOmplete ULTRA Tablets, EDTA free	*Sigma 5892791001*
16% Paraformaldehyde	*Thermo Fisher 28908*
Glycine	*Euromedex 26-128-6405-C*
EDTA	*Sigma E6758*
Igepal	*Euromedex UN3500-A*
MgCl2	*Euromedex 2189*
NaOH	*Sigma S5881*
NaCl	*Euromedex 1112-A*
LiCl	*Sigma L7026*
Sodium deoxycholate	*Euromedex BI-DD0150*
Tris-HCl	*Euromedex EU0011-C*
SDS	*Invitrogen I5525*
Sodium bicarbonate	*Euromedex 6885-1-A*
Nuclease-free water	*Thermo Fisher 10977*
Triton X-100	*Sigma 93426*
Proteinase K	*Qiagen 1133759*
RNase A	*Qiagen 1126483*
CHIP-grade Protein A/G Magnetic Beads	*Thermo Fisher 26162*
Bovine serum albumin	*Pan Biotech P06-B91500*
PBS	*Gibco 14190*
Agarose	*Euromedex D5-E*
Nucleospin Gel and PCR clean up	*Macherey-Nagel 740609*
H3K27me3 antibody	*Cell Signaling 9733T*
IgG antibody	*Cell Signaling 3900S*

Supplementary Table S9: Clinical and biological data of HB samples

Patient	Samples	16G sig	4G sig	Gender	Age (months)	TV	VI	S/MN	M	SC	CH	P	CHIC-HS	Risk	AFP (ng/mL)	ß-catenin RNAseq
P01	T	C1	C1	F	NA	74	N	S	N	Y	N	II	B	NA	NA	G34R
P02	T/NT	C1	C1	F	11	60	N	S	N	N	Y	II	B	SR	153840	exon3 del
P03_met	T	C2	C2A	M	53	NA	Y	S	Y	N	Y	III	D	HR	342000	NA
P04_a	T/NT	C1	C1	F	14	197	N	S	N	N	N	II	B	SR	300	exon3 del
P04_b	T	C1	C1	F	14	197	N	S	N	N	Y	II	B	SR	300	del>200
P04_c	T/NT	C1	C1	F	14	197	N	S	N	N	Y	II	B	SR	300	exon3 del
P05	T/NT	C2 / C1	C1	M	NA	1 573	Y	M	N	N	Y	IV	D	HR	485000	D32T
P06	T/NT	C2	C2B	M	6	485	N	S	N	Y	Y	II	B	SR	849499	WT
P07	T	C1	C1	M	7	1 428	N	M	N	Y	Y	II	C	SR	246867	del<200
P07_bio	T	C1	C1	M	7	1 428	N	M	N	N	N	II	C	SR	246867	del<200
P08	T	C1	C1	M	NA	NA	Y	M	NA	Y	Y	II	C	SR	1400	NA
P09	T/NT	C1	C2B	F	6	160	N	S	N	N	Y	III	B	NA	401807	exon3 del
P10	T/NT	C1	C1	F	7	888	N	S	N	Y	NA	III	B	SR	960000	in frame deletion aa5-42 (86 nt)
P11	T/NT	C1	C1	M	35	854	Y	M	N	N	Y	IV	D	HR	1285000	G34V
P12	T/NT	C2	C1	F	155	859	Y	M	Y	N	Y	I	D	SR	154382	NA
P13	T/NT	C2	C2A	M	13	1 328	Y	S	Y	N	NA	III	D	HR	337000	del 210
P14	T/NT	C1	C1	M	15	NA	Y	M	N	N	Y	IV	C	SR	14514	NA
P15	T/NT	C1	C1	M	5	760	N	S	N	N	Y	II	B	SR	56613	exon3 del
P16	T/NT	C1	C1	M	36	800; 500	Y	M	N	N	Y	IV	D	HR	54982	WT
P17	T/NT	C2	C2A	M	12	520	N	S	N	Y	Y	II	B	SR	837000	exon3 del
P18	T	C1	C1	M	NA	NA		M	N	Y	Y		D	NA	200000	NA
P19	T/NT	NA	C1	M	9	2	N	S	N	Y	Y	I	B	HR	88000	in frame deletion aa15-79 (195 [illegible]
P20	T/NT	C1	C1	F	12	41	N	S	N	N	Y	II	B	SR	318800	D32V
P21	T	C1	C1	M	17	1 997	N	M	Y	N	Y	III	D	HR	123000	WT (FAP)
P22	T/NT	C1	C1	F	NA	2	Y	M	Y	Y	Y	III	D	HR	2000000	G34V
P23	T/NT	C2	C2A	M	50	4	Y	M	Y	Y	Y	II	D	HR	350000	in frame deletion aa25-36 (36 nt)
P24	T/NT	C1	C1	M	4	5	N	M	N	Y	Y	III	C	SR	82000	S37A
P25	T	C2	C2B	M	11	173	N	M	N	Y	Y	II	C	SR	18599	del < 200
P26_a	T	C2	C2A	M	28	NA	NA	NA	NA	NA	Y	II	D	HR	1500000	NA
P26_b	T/NT	C2	C2B	M	28	2 805	Y	M	Y	Y	Y	II	D	HR	1500000	in frame deletion aa37-61 (75) nt, [illegible]
P27	T/NT	C2	C2A	F	NA	598	N	S	N	N	Y	II	B	SR	2355000	G34V
P28	T/NT	C1	C1	F	11	1 540	Y	M	N	Y	Y	IV	C	HR	360000	exon3 del
P29	T/NT	C1	C1	F	NA	NA	N	S	N	N	N	II	A	NA	300	in frame deletion aa19-79 (180 [illegible]
P30	T	C1	C1	M	25	1 373	N	S	N	N	Y	II	B	SR	3714	NA
P31	T	C2	C2A	F	NA	NA	NA	NA	NA	N	N		NA	NA	NA	NA
P32	T/NT	C2	C2B	F	4	1	N	S	N	N	Y	II	B	SR	934371	WT
P33	T/NT	C1	C1	M	10	241	N	M	N	Y	Y	III	C	SR	300000-500000	in frame deletion aa28-61 (102 [illegible]
P34	T/NT	C2 / C1	C1	M	4	520	N	S	N	N	Y	III	B	SR	518000	T41A
P35	T/NT	C1	C1	F	24	912	Y	M	N	Y	Y	II	C	HR	1000000	G34E
P36	T/NT	C2 / C1	C2B	M	NA	NA	N	S	N	N	Y	IV	D	HR	NA	WT
P37_a	T	C1	C1	F	24	402	N	S	N	N	Y	I	B	SR	66810	NA
P37_b	T	C1	C1	F	24	402	N	S	N	N	Y	I	B	SR	66810	D32V
P38	T/NT	C1	C1	M	11	1 584	N	S	N	N	Y	II	B	NA	155385	exon3 del
P39	T/NT	C1	C1	M	14	220	N	S	N	N	Y	II	B	SR	19096	WT
P40	T	NA	NA	M	72	NA	NA	NA	O	N	Y	IV	NA	NA	1000000	NA
P97	T/NT	NA	NA	F	12	58	NA	S	N	N	Y	II	NA	NA	111343	NA
P191	T/NT	NA	NA	M	20	678	Y	M	N	N	Y	IV	NA	NA	1855	NA
P200	T/NT	NA	NA	M	13	NA	NA	NA	Y	N	Y	II	NA	NA	1 286 980	NA

4G sig: 4-gene signature
16G sig: 16-gene signature
CH: chemotherapy
HR: high risk
NA: non available
M: metastasis

P: PRETEXT stage
SC: Small Cells presence
S/MN: solitary/multiple nodules
SR: standard risk
TV: Tumor volume (cm3)
VI: vascular invasion

Cession N°: 2018-ACP-BCT-01R
Ref : B-CRB-DE-PSAD13-003-01
